# Base‐Mediated Radical Borylation of Alkyl Sulfones

**DOI:** 10.1002/chem.202103866

**Published:** 2021-11-29

**Authors:** Mingming Huang, Jiefeng Hu, Ivo Krummenacher, Alexandra Friedrich, Holger Braunschweig, Stephen A. Westcott, Udo Radius, Todd B. Marder

**Affiliations:** ^1^ Institute of Inorganic Chemistry and Institute for Sustainable Chemistry & Catalysis with Boron Julius-Maximilians-Universität Würzburg Am Hubland 97074 Würzburg Germany; ^2^ Department of Chemistry & Biochemistry Mount Allison University Sackville NB E4L 1G8 Canada

**Keywords:** boron, boronate, boronic acid, metal-free, radical

## Abstract

A practical and direct method was developed for the production of versatile alkyl boronate esters via transition metal‐free borylation of primary and secondary alkyl sulfones. The key to the success of the strategy is the use of bis(neopentyl glycolato) diboron (B_2_neop_2_), with a stoichiometric amount of base as a promoter. The practicality and industrial potential of this protocol are highlighted by its wide functional group tolerance, the late‐stage modification of complex compounds, no need for further transesterification, and operational simplicity. Radical clock, radical trap experiments, and EPR studies were conducted which show that the borylation process involves radical intermediates.

## Graphical abstract

**The utilization** of inactivated alkyl sulfones as alkyl radical precursors in a base‐mediated borylation reaction with B_2_neop_2_ is reported, allowing direct access to valuable alkyl boronate esters without further transesterification. This approach is scalable and is tolerant to a variety of functional groups and substrates including complex molecules.

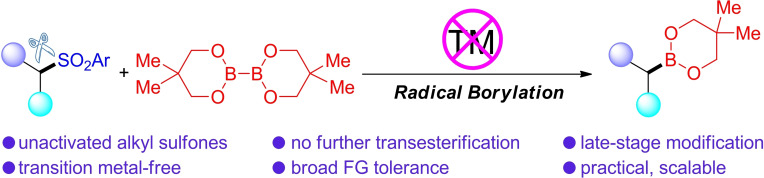

## Introduction

**Scheme 1:**
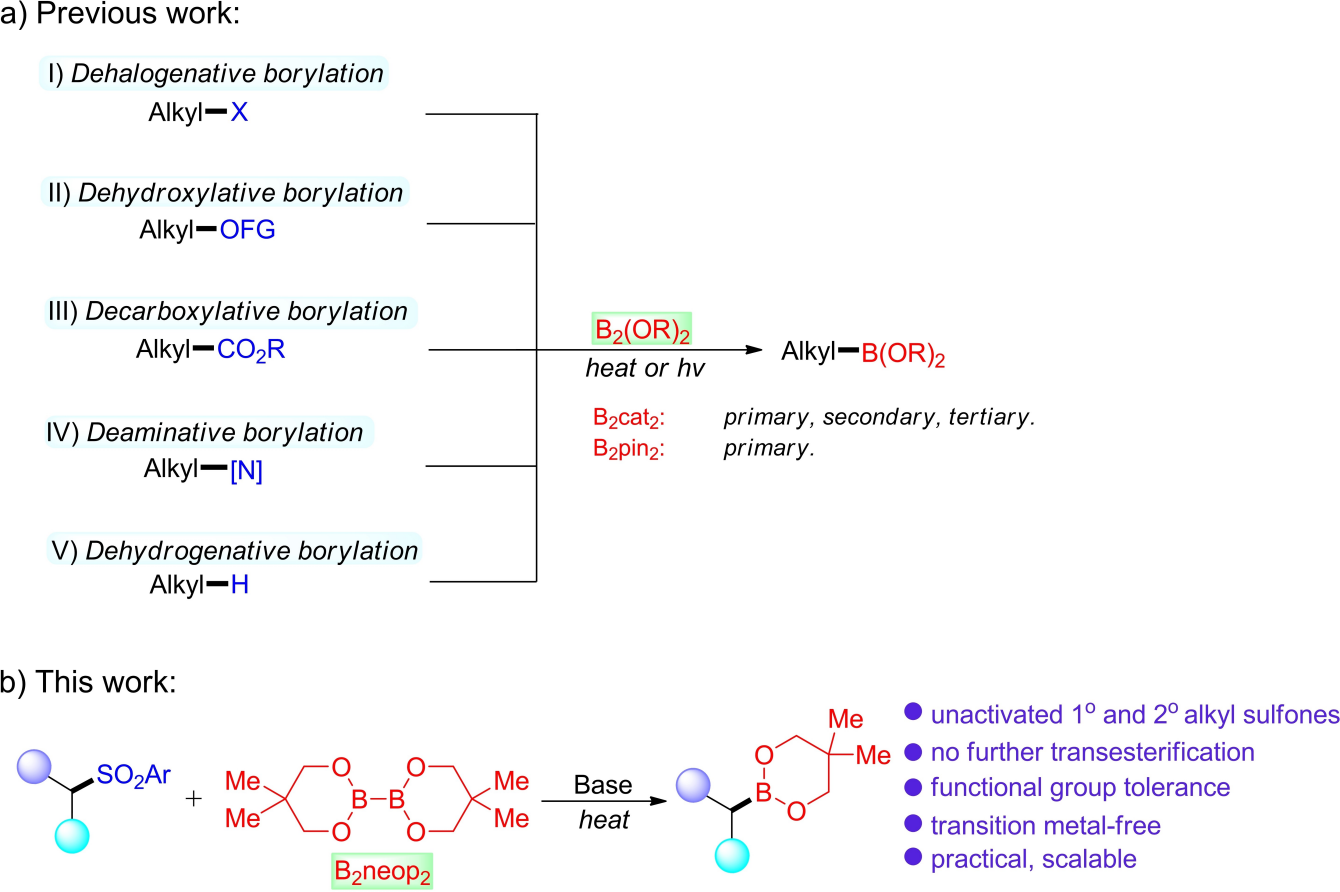
Transition metal‐free radical borylation reactions.

The preparation of alkyl boronates is an important and valuable process in organic synthesis because these compounds play an essential role in synthetic chemistry, drug discovery, and materials science.^[1]^ Early research typically focused on transmetalation using organolithium or Grignard reagents,^[2]^ and the metal‐catalyzed hydroboration^[3]^ or diboration of alkenes.^[4]^ More recently, transition metal‐catalyzed cross‐coupling strategies for the direct borylation of alkyl halides have been well‐developed by Marder, Steel and Liu,^[5]^ Fu,^[6]^ Ito,^[7]^ Cook,^[8]^ and others.^[9]^ This Miyaura‐type borylation has now been widely applied using sustainable chemical feedstocks, such as alcohols,^[10]^ carboxylic acids^[11]^ and amine derivatives.^[12]^ With increasing attention to sustainable chemistry, transition metal‐free radical borylation protocols have emerged as an important tool to access alkyl boronate esters (Scheme 1 I–V).^[13]^ Bis(catecholato)diboron (B_2_cat_2_)^[14]^ is an efficient diboron(4) compound for the borylation of redox‐active esters in the presence of amide‐based solvents, as first reported by Aggarwal and co‐workers.^[15]^ As the catechol boronate esters are sensitive to hydrolysis, transesterification with pinacol in the presence of Et_3_N is employed. Then, Studer^[16]^ and Jiao^[17]^ independently reported the transition metal‐free radical borylation of primary and secondary alkyl bromides and iodides employing B_2_cat_2_ under blue LED irradiation, providing a broad range of alkylboronate esters. In addition, visible light‐mediated approaches for deoxygenative borylation^[18]^ and decarboxylative borylation[^15^, ^19^] using B_2_cat_2_ as the boron source in *N,N*‐dimethylformamide (DMF) or *N,N*‐dimethylacetamide (DMA) were introduced by Studer and Aggarwal. Interestingly, Shi,^[20]^ Aggarwal,^[21]^ and Glorius^[22]^ independently reported metal‐free deaminative borylations of pyridinium salt‐activated alkylamines with a proposed B_2_cat_2_‐DMA sp^2^‐sp^3^ adduct^[23]^ under visible light or with heating. In 2020, a photochemical method for the dehydrogenative borylation of non‐activated alkanes using a chloride source as a hydrogen atom transfer (HAT) catalyst was described.^[24]^ The trapping of alkyl radicals with B_2_pin_2_ ^[25]^ without transesterification was also reported; however, the substrates for this method are limited to primary alkyl halides.^[26]^ The sustained expansion of transition metal‐free direct radical borylation without transesterification is extremely desirable but challenging.

Sulfone functionalities are important and fundamental structural motifs in chemistry.^[27]^ In general transition metal‐catalyzed cross‐coupling processes, they are commonly used as electrophiles to construct C−C and C−heteroatom bonds. Generally, studies in this area have mainly concentrated on aryl,^[28]^ vinyl,^[29]^ allylic^[30]^ and benzylic sulfones.^[31]^ In contrast, the desulfonative transformation of inactivated alkyl sulfones is scarce and challenging because of the inherent low reactivity of the C−SO_2_ bond.^[32]^ Notably, alkyl sulfones present several advantages over alkylhalides, including the tolerance of α‐functionalization before coupling, as well as their stability and crystallinity, which allow for convenient manipulation. Pragmatically speaking, alkyl sulfones can be easily synthesized from alkyl alcohols or halides with inexpensive and odorless sodium arenesulfinate or diphenyl disulphide to produce highly crystalline products. In 2018, Scheidt et al. developed a Cu‐catalyzed approach for the hydroboration of aldimines with B_2_pin_2_ using *N*‐benzoyl‐protected *α*‐tosylamines as starting materials, providing enantioenriched *α*‐amidoboronates.^[33]^ In the same year, Baran and co‐workers reported a breakthrough in the desulfonative radical process of alkyl sulfones in the presence of aryl zinc reagents using nickel(II) acetylacetonate and a bipyridine‐type ligand as catalyst precursor.^[34]^ Recently, transition metal‐free, pyridine‐catalyzed borylation of benzyl sulfones with B_2_pin_2_ was reported by the Crudden group;^[31d]^ however, their study mainly focused on benzylic substrates. In view of recent progress and our interest in borylation reactions,^[35]^ we postulated that if a transient alkyl radical could be generated from an alkyl sulfone using light or heat, this species might undergo radical borylation with an appropriate diboron reagent for the construction of C(sp^3^)‐B bonds. Herein, we report our initial results on the utilization of alkyl sulfones as alkyl radical precursors in a base‐mediated borylation reaction with B_2_neop_2_, thus allowing for direct access to valuable alkyl boronate esters without transesterification.

## Results and Discussion

**Scheme 2:**
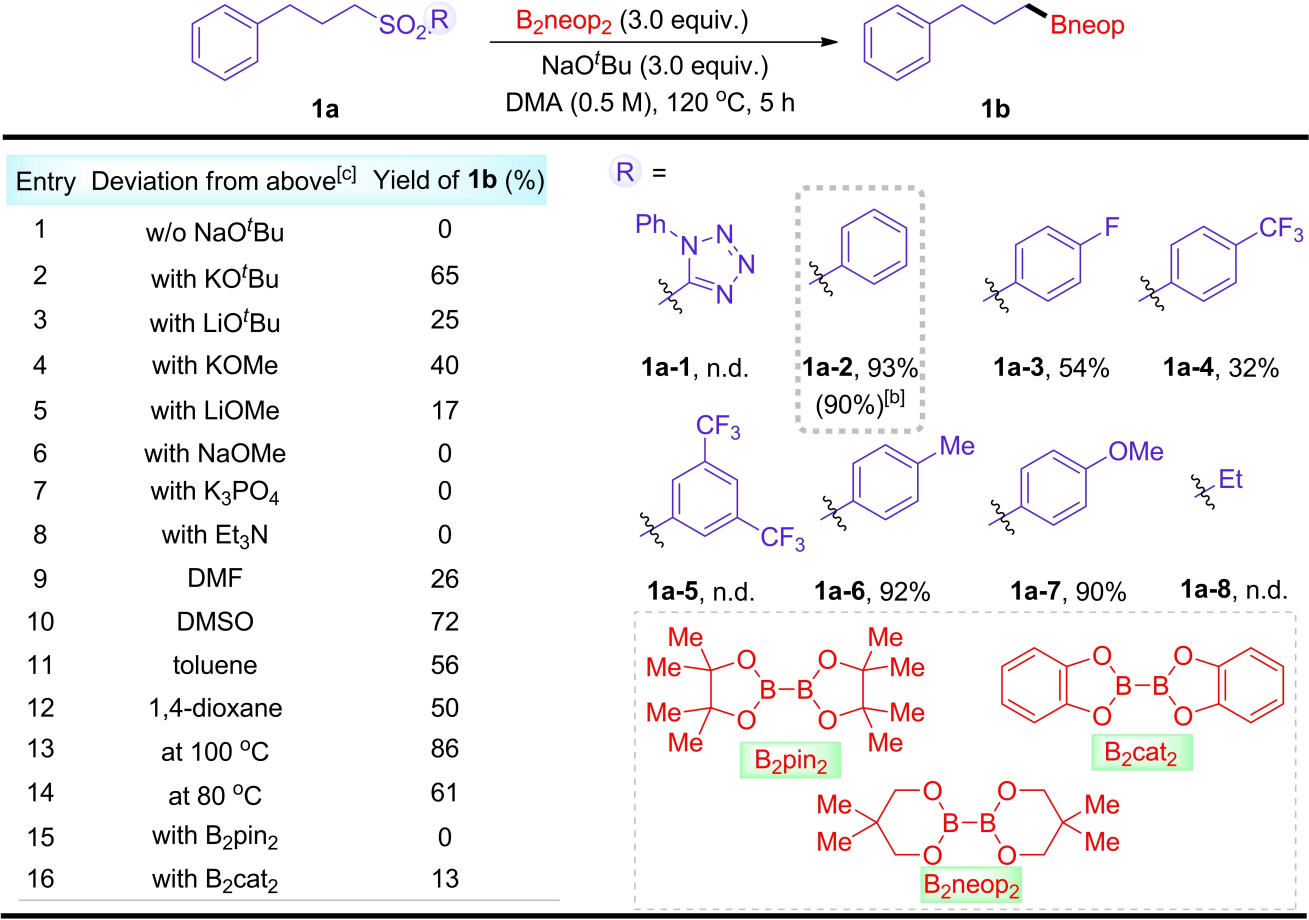
Optimization of reaction conditions for the borylation of alkyl sulfones^[a]^ [a] Reaction conditions: a mixture of alkyl sulfone **1 a** (0.5 mmol, 1.0 equiv.), B_2_neop_2_ (3.0 equiv.), and NaO^*t*^Bu (3.0 equiv.), DMA (1.0 mL), 120 °C, 5 h, under Ar. The yields were determined by GC‐MS analysis using an internal standard and are averages of two runs. [b] Yield of Isolated product after chromatographic workup. [c] Using **1 a**–**2** as starting material. n.d.=not detected.

We initiated this study by investigating a sulfone bearing a phenyl‐tetrazole moiety, **1 a**–**1**, previously reported by Baran, for potential borylation using B_2_neop_2_,^[36]^ but the target product **1 b** was not detected by GC‐MS in the presence of NaO^*t*^Bu in DMA at 120 °C. However, alkyl aryl sulfone **1 a**–**2** was effectively converted to alkylboronic ester **1 b** under these conditions with an isolated yield of 90 %. Other sulfones (**1 a**–**3**‐**1 a**–**7**) with different electronic properties and sizes were also explored in this system, and we found that substrates with electron‐withdrawing groups gave low yields and reaction with **1 a**–**8** was unsuccessful (Scheme 2). Subsequently, we began to study the influence of other parameters on this borylation reaction by using ((3‐phenylpropyl)sulfonyl)benzene **1 a**–**2** as the model substrate. Upon removal of the base from this system, alkylboronates were not formed, leaving only unreacted starting materials (entry 1). Other alkoxides, such as KO^*t*^Bu, LiO^*t*^Bu, KOMe and LiOMe, gave lower yields (entries 2–5), and no reaction took place with NaOMe, K_3_PO_4_ and Et_3_N under these conditions (entries 6–8). In the presence of 3.0 equivalents of NaO^*t*^Bu, we observed moderate reactivity in other organic solvents, for example DMF, DMSO, toluene, and 1,4‐dioxane (entries 9–12). Lowering the reaction temperature to 100 °C resulted in a slightly diminished yield of **1 b** under the otherwise optimal conditions (entry 13). At a lower temperature (80 °C), the results were inferior (entry 14). B_2_pin_2_ and B_2_cat_2_ are commonly used sources of boron in radical borylation reactions. However, B_2_pin_2_ failed to afford any product under our conditions (entry 15). The reaction also resulted in poor conversion using B_2_cat_2_ instead of B_2_neop_2_ (entry 16).

**Scheme 3:**
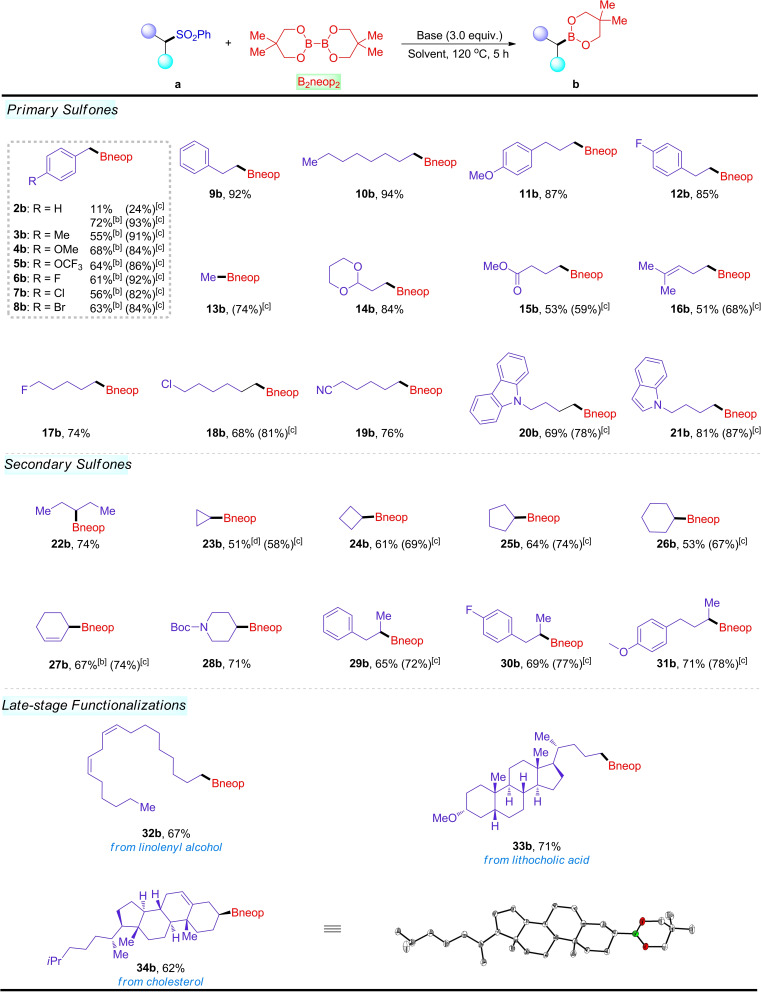
Alkyl sulfone substrate scope.^[a]^ [a] Reaction conditions: A mixture of alkyl aryl sulfone **a** (0.5 mmol, 1.0 equiv.), B_2_neop_2_ (3.0 equiv.), and NaO^*t*^Bu (3.0 equiv.) in DMA (1 mL) was stirred for 5 h at 120 °C under Ar; isolated yields after chromatography. [b] Using KOMe (1.2 equiv.) and B_2_neop_2_ (1.2 equiv.) in 1,4‐dioxane (2.0 mL) at 110 °C for 2 h under Ar. [c] Yields in parentheses were determined by ^1^H NMR analysis. [d] From sulfone substrate 1‐(cyclopropylsulfonyl)‐4‐methylbenzene.

Under optimized reaction conditions, we proceeded to investigate the scope of alkyl aryl sulfones using B_2_neop_2_ as a coupling reagent (Scheme 3). Initially, (benzylsulfonyl)benzene **2 a** was examined in reactions with B_2_neop_2_ under standard conditions, affording the corresponding product **2 b** in 24 % NMR yield. To improve the conversion efficiency of the transformation, modified conditions were applied to the desulfonative borylation of benzyl sulfones (see Table S2 in the Supporting Information for a survey of the reaction conditions). The borylation reaction is tolerant to OCF_3_ (**5 b**) and F, Cl, and Br groups (**6 b**, **7 b**, **8 b**). Aryl halide substrates worked well in the process, exhibiting selective cleavage of the C(sp^3^)‐SO_2_ bonds over the aryl C−X bonds (**6 b**–**8 b**). Sulfone substrates with different carbon chains were successfully borylated using NaO^*t*^Bu as the base (**9 b**–**12 b**, 85 %–94 %). In particular, methyl boronate ester **13 b**, which decomposes readily on silica gel, was formed from methyl phenyl sulfone in 74 % NMR yield under our conditions. In addition, under these conditions, other functionalities, such as ether, ester, olefin, F and Cl, were well‐tolerated and proceeded to generate the desired alkylboronates in moderate to high isolated yields (**14 b**–**18 b**). The sulfone bearing a cyano motif also worked well in this system, giving **19 b** in 76 % yield. Heterocycles, including carbazole and indole, exhibited high levels of reactivity, delivering **20 b** and **21 b**, respectively. A range of secondary alkyl sulfones were also efficiently transformed into the corresponding alkyl boronates **22 b**–**31 b** in good yields (51 %–74 %). Interestingly, this method could be used for the late‐stage derivatization of complex compounds. Linolenyl alcohol derivative **32 a**, bearing two *cis*‐alkene groups, reacted with B_2_neop_2_ under our conditions, providing product **32 b** in 67 % yield. The lithocholic acid derivative **33 a** afforded the borylation product **33 b** in 71 % yield, and the complex sulfone derivative **34 a** was also readily converted into alkyl boronate ester **34 b** without observation of other isomers. The molecular structure of **34 b** was determined by single‐crystal X‐ray diffraction. Tertiary sulfone substrates were also examined, but no desired borylated products were observed (see the Supporting Information).

**Scheme 4:**
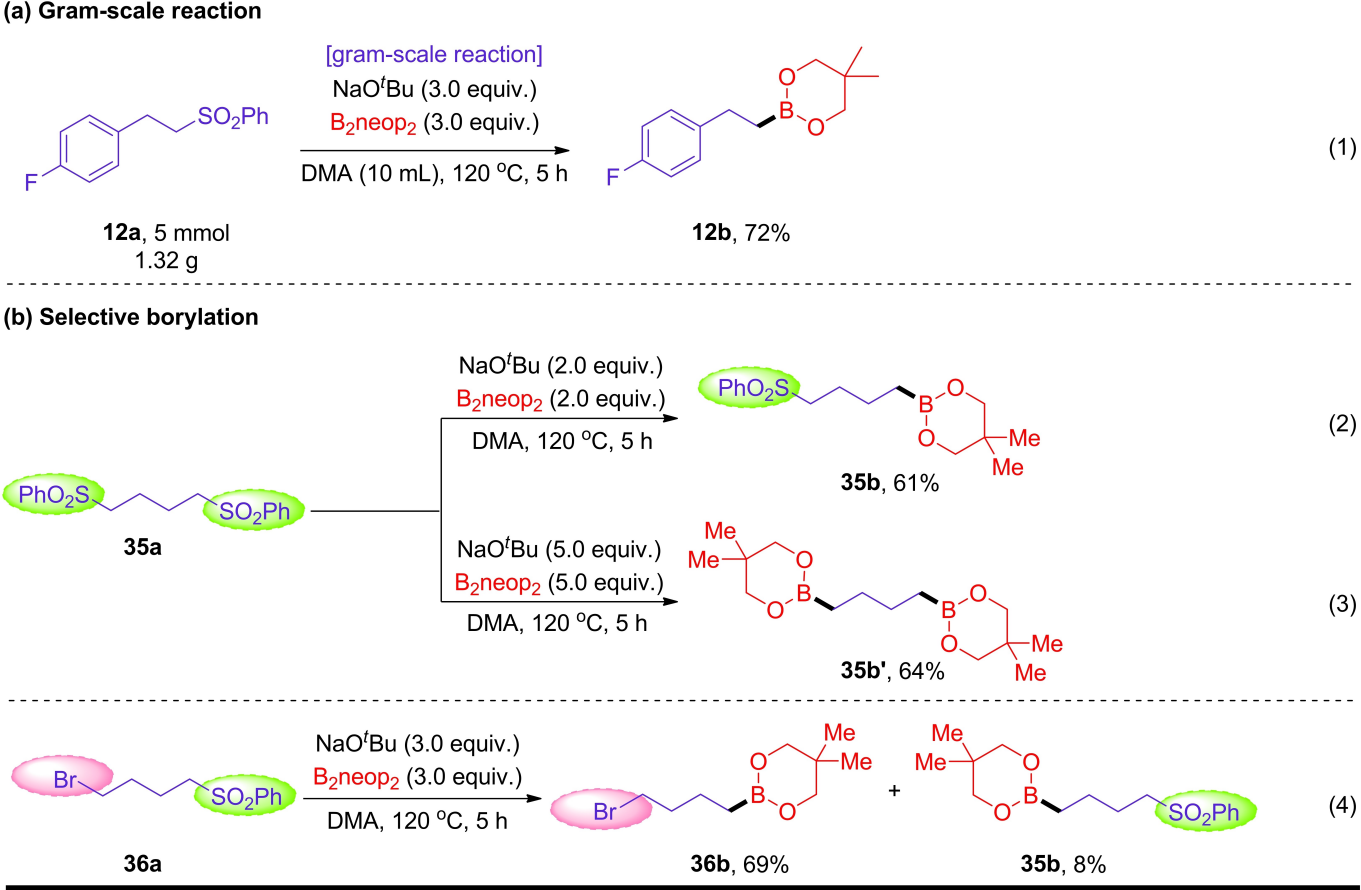
Gram‐scale reaction and selective borylation.

To showcase the applicability of the process, a 1 g scale reaction was performed with **12 a** giving a 72 % isolated yield of borylated product **12 b**, indicating the viability of this strategy for the large‐scale production of alkyl boronate esters [(Scheme 4a, Eq. (1)]. The borylation of 1,4‐bis(phenylsulfonyl)butane **35 a** selectively generated the mono‐borylated product **35 b** or di‐borylated product **35 b’** under different reaction conditions [Scheme 4b, Eqs. (2) and (3)]. Different from substrate **18 a**, **36 a** bears both alkyl bromide and alkyl aryl sulfone sites, afforded **36 b** as the main product, accompanied by only 8 % yield of the debromination product **35 b** [Scheme 4b, Eq. (4)].

**Scheme 5:**
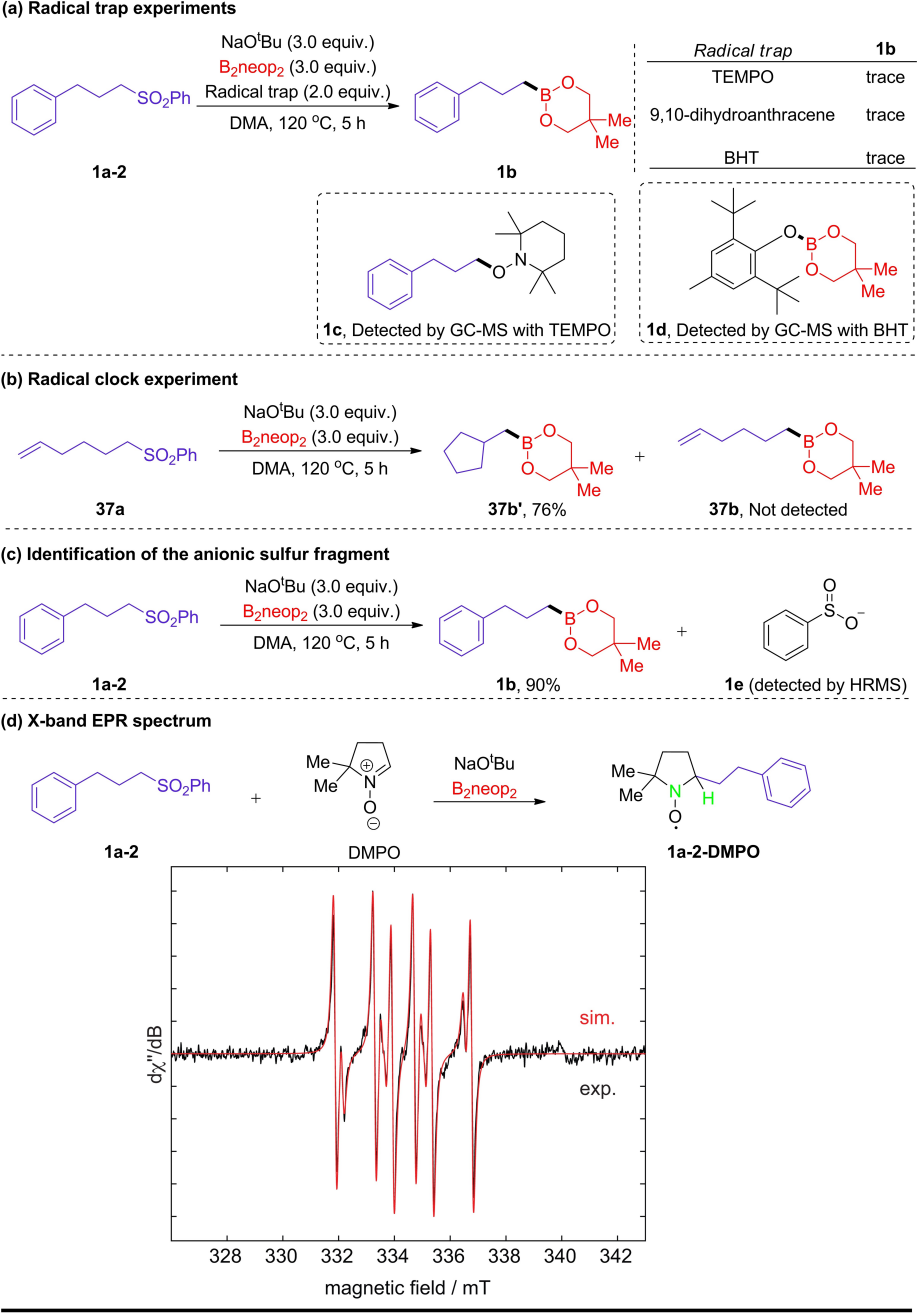
Mechanistic studies. a) Radical trap experiments. b) Radical clock experiment. c) Identification of the anionic sulfur fragment. d) Experimental (black) and simulated (red) continuous‐wave (CW) X‐band EPR spectra of the DMPO spin trapping experiment. Best‐fit simulation parameters: *g*_iso_=2.0053, *a*(^14^N)=40 MHz (14.3 G) and *a*(^1^H)=58 MHz (20.6 G, major species); *g*_iso_=2.0053, *a*(^14^N)=40 MHz (14.3 G) and *a*(^1^H)=43 MHz (15.4 G, minor species).

We next conducted some control experiments to explore this borylation process. Firstly, when 2,2,6,6‐tetramethyl‐1‐piperidinyloxyl (TEMPO), 9,10‐dihydroanthracene, or butylated hydroxytoluene (BHT) were added as radical traps, **1 b** was barely detected by GC‐MS (Scheme 5a). Additionally, TEMPO‐trapped product **1 c** and BHT‐Bneop adduct **1 d** were observed by GC‐MS and HRMS analysis. The formation of a boron radical is suggested during the reaction based on the observation of adduct **1 d**. Secondly, a radical clock experiment was carried out with hex‐5‐en‐1‐ylsulfonylbenzene **37 a** as the substrate, and the cyclic boronate ester **37 b’** was isolated exclusively in 76 % yield (Scheme 5b). These results support a radical borylation mechanism. In addition, after the reaction completed, we detected phenylsulfinate by HRMS, which was generated by cleavage of the C−S bond (Scheme 5c). We also used electron paramagnetic resonance (EPR) spectroscopy to investigate the nature of the radicals in the process (Scheme 5d). 5,5‐Dimethyl‐1‐pyrroline *N*‐oxide (DMPO) was added to act as a spin‐trap, which reacts with short‐lived alkyl radicals to produce the more stable and observable DMPO‐trapped radical. The EPR signal depicted in Scheme 5(d) shows a *g*_iso_ value of 2.0053 with a hyperfine splitting which is in accordance with coupling to the adjacent hydrogen and nitrogen atoms (highlighted in green in Scheme 5d). The major alkyl‐DMPO adduct is accompanied by a second minor species of unknown composition. A sulfonyl radical seems reasonable based on the *g* factor and observed couplings; we can definitely exclude a phenyl radical, which could also be generated by extrusion of sulfur dioxide from a putative sulfonyl radical. The trapping experiment was also conducted under the same conditions without the addition of B_2_neop_2_, and no EPR signal was observed (see the Supporting Information for more details).

**Scheme 6:**
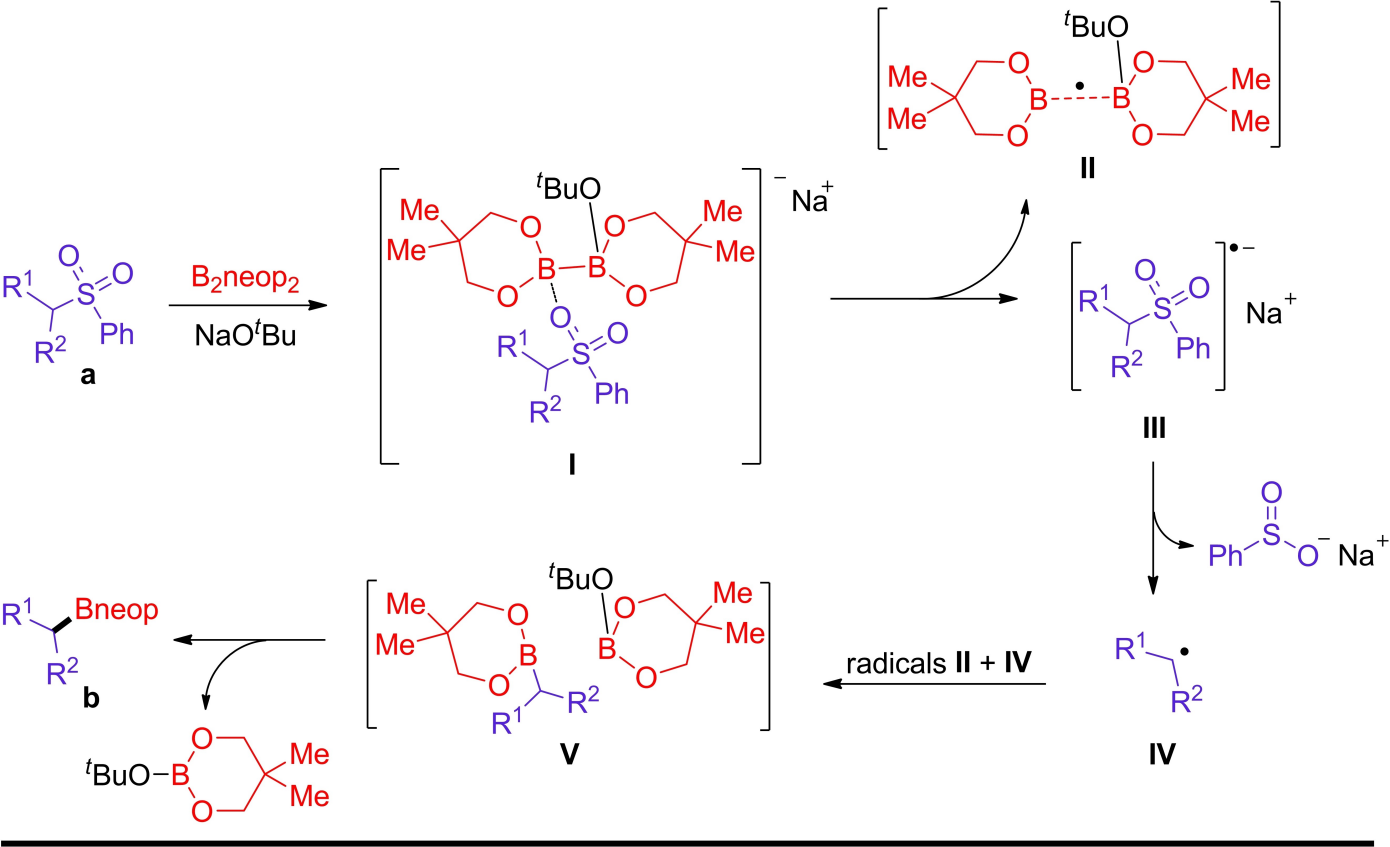
Proposed mechanism.

Based on the above observations and previous work on radical borylations, a possible mechanism is shown in Scheme 6. We propose a possible ate complex **I**, which is generated from **a**, B_2_neop_2_, and alkoxide.[^23^, ^37^] This intermediate undergoes intramolecular electron transfer to release radical **II** and sulfone radical anion **III**. Then, alkyl radical **IV** is formed by elimination of phenylsulfinate from sulfone radical anion **III**. Subsequently, alkyl radicals **II** and **IV** combine to produce species **V**, leading to the borylated product and ^*t*^BuOBneop.^[38]^ While we have previously explored the electronic properties of B_2_neop_2_, B_2_cat_2_, and B_2_pin_2_,[^36b^, ^39a^] or their monoboron counterparts,^[39b]^ in various contexts, the reaction mixtures in the present work are likely more complex than depicted in our proposed mechanism, and it is not clear to us why B_2_neop_2_ ^[36a]^ shows such unique reactivity among the diboron compounds investigated in our newly developed process. Thus, while our proposal is consistent with our observations, further experimental and theoretical studies will be required to gain a complete understanding of the nature of all species present during the reaction.

## Conclusions

In summary, we have successfully achieved the base‐promoted radical borylation of alkyl sulfones using B_2_neop_2_, which displayed enhanced reactivity compared with the diboron reagents B_2_cat_2_ and B_2_pin_2_. This approach is tolerant to a variety of functional groups and substrates, including complex molecules. Preliminary mechanistic studies revealed a plausible reaction pathway involving the formation of alkyl radicals. It appears that optimization of both steric and electronic effects may be required to achieve both the formation of the proposed adduct **I**, which involves both neutral and anionic Lewis base donors, and the stability of critical reaction intermediates, although we cannot currently provide a more detailed rationale. Additional studies are required to achieve a more detailed understanding of the intimate mechanism of the process and the reason that B_2_neop_2_ shows optimum reactivity among the diboron(4) reagents examined. We anticipate that these findings will prompt further development of desulfonative radical cross‐coupling reactions.

## Crystal structure

Deposition Numbers 2079501 (for **34 b**) contains the supplementary crystallographic data for this paper. These data are provided free of charge by the joint Cambridge Crystallographic Data Centre and Fachinformationszentrum Karlsruhe Access Structures service.

## Conflict of interest

There are no conflicts to declare.

1

## Supporting information

As a service to our authors and readers, this journal provides supporting information supplied by the authors. Such materials are peer reviewed and may be re‐organized for online delivery, but are not copy‐edited or typeset. Technical support issues arising from supporting information (other than missing files) should be addressed to the authors.

Supporting Information

## Data Availability

The data that support the findings of this study are available from the corresponding author upon reasonable request.
