# Supplementary material for: Base‐Mediated Radical Borylation of Alkyl Sulfones
Source: Chemistry. 2021 Nov 29;28(3):e202103866. doi: 10.1002/chem.202103866 (PMC9299846; doi:10.1002/chem.202103866)
Supplement: Supplementary file 1 — Supporting Information [file CHEM-28-0-s001.pdf]

Chemistry–A European Journal

Supporting Information

Base-Mediated Radical Borylation of Alkyl Sulfones

Mingming Huang, Jiefeng Hu,* Ivo Krummenacher, Alexandra Friedrich, Holger Braunschweig, Stephen A. Westcott, Udo Radius,* and Todd B. Marder*

Table of Contents

1 Experimental Section 1

1.1 General Considerations 1

1.2 Optimization of the Reaction Conditions 2

1.3 Synthesis of Sulfone Substrates 6

1.4 Details of the Borylation of Alkyl Sulfones 26

1.5 Unsuccessful Tertiary Sulfone Substrates 40

2 Gram-scale Reaction 41

3 Selective Borylation 42

4 Preliminary Mechanistic Investigations 44

4.1 Radical Trap Experiments 44

4.2 Radical Clock Experiment 46

4.3 EPR Spectroscopic Study 47

5 Single-Crystal X-ray Diffraction Analysis 49

6 References 52

7 NMR Spectra 54

1 Experimental Section

1.1 General Considerations

All reactions and subsequent manipulations were performed under an argon atmosphere using standard Schlenk techniques or in a glovebox (Innovative Technology Inc. and Braun Uni Lab). All reactions were carried out in oven-dried glassware. Reagent grade solvents (Fisher Scientific and J.T. Baker) were nitrogen saturated and were dried and deoxygenated using an Innovative Technology Inc. Pure-Solv 400 Solvent Purification System, and further deoxygenated using the freeze-pump-thaw method. $CDCl_3$ was purchased from Sigma-Aldrich. The diboron reagents B_2neop_2, B_2cat_2 and B_2pin_2 were a generous gift from AllyChem Co. Ltd. All other reagents were purchased from Alfa-Aesar, Sigma-Aldrich or ABCR, and were checked for purity by GC-MS and/or ^{1}H NMR spectroscopy and used as received.

NMR spectra were recorded at 298 K using Bruker Avance 300 (^{1}H, 300 MHz; ^{13}C, 75 MHz, ^{11}B, 96 MHz), Bruker DPX-400 (^{1}H, 400 MHz; ^{13}C, 100 MHz, ^{11}B, 128 MHz; ^{19}F, 376 MHz), or Bruker Avance 500 (^{1}H, 500 MHz; ^{13}C, 125 MHz, ^{11}B, 160 MHz; ^{19}F, 470 MHz) spectrometers. ^{1}H NMR chemical shifts are reported relative to TMS and were referenced *via* residual proton resonances of the corresponding deuterated solvent ($CDCl_3$: 7.26 ppm) whereas ^{13}C{^{1}H} NMR spectra are reported relative to TMS using the natural-abundance carbon resonances ($CDCl_3$: 77.16 ppm). However, signals for the carbon attach to boron, C-B, are usually too broad to observe in the ^{13}C{^{1}H} NMR spectra. ^{11}B and ^{19}F NMR chemical shifts are reported relative to external BF_3•OEt_3 or $CFCl_3$, respectively. Coupling constants are given in Hertz. Elemental analyses were performed in the microanalytical laboratory of the Institute of Inorganic Chemistry, Universität Würzburg, using an Elementar vario micro cube instrument. Automated flash chromatography was performed using a Biotage® Isolera Four system, on silica gel (Biotage SNAP cartridge KP-Sil 10 g and KP-Sil 25 g). Commercially available, precoated TLC plates (Polygram® Sil G/UV254) were purchased from Machery-Nagel. The removal of solvent was performed on a rotary evaporator *in vacuo* at a maximum temperature of 30 °C. GC-MS analyses were performed using a Thermo Fisher Scientific Trace 1310 gas chromatograph (column: TG-SQC 5% phenyl methyl siloxane, 15 m, ø 0.25 mm, film 0.25 µm; injector: 250 °C; oven: 40 °C (2 min), 40 °C to 280 °C; carrier gas: He (1.2 mL min^{-1}) or an Agilent 7890A gas chromatograph (column: HP-5MS 5% phenyl methyl siloxane, 30 m, Ø 0.25 mm, film 0.25 µm; injector: 250 °C; oven: 40 °C (2 min), 40 °C to 280 °C (20 °C min^{-1}); carrier gas: He (1.2 mL min^{-1}) equipped with an Agilent 5975C inert MSD with triple-axis detector operating in EI mode and an Agilent 7693A series auto sampler/injector. High-resolution mass spectra were obtained using a Thermo Scientific Exactive Plus spectrometer equipped with an Orbitrap Mass Analyzer. Measurements were accomplished using an ASAP/APCI source with a corona needle, and a carrier-gas (N_2) temperature of 250 °C.

1.2 Optimization of the Reaction Conditions

General procedure of optimization for Table S1. In an argon-filled glovebox, the alkyl sulfone **1a-2** (0.5 mmol, 1.0 equiv.), dissolved in solvent (1 mL), was added to a 10 mL thick-walled reaction tube equipped with a magnetic stirring bar. Base and the boron source were added. The reaction mixture was stirred at indicated temperature for 5 h, then diluted with Et_2O (2 mL) and filtered through a pad of Celite (Ø 3 mm x 8 mm). The solvent was evaporated under reduced pressure and dodecane was added as an internal standard and the crude reaction mixture was analyzed by GC-MS.

Table S1: Optimization of reaction conditions for the borylation of ((3-phenylpropyl)sulfonyl)benzene **1a-2**.

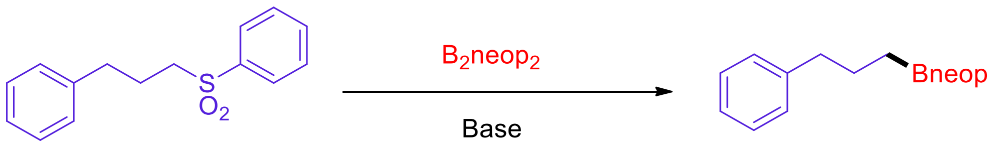

Entry	Base (eq.)	Solvent	B_2neop_2 (eq.)	T (°C)	Yield of **1b** (%)[a]
1	KOMe (3.0)	DMA	3.0	120	40
2	KO*t*Bu (3.0)	DMA	3.0	120	65
3	NaO*t*Bu (3.0)	DMA	3.0	120	93 (90)[b]
4	NaOMe (3.0)	DMA	3.0	120	0
5	CsF (3.0)	DMA	3.0	120	28
6	K_3PO_4 (3.0)	DMA	3.0	120	0
7	LiOMe (3.0)	DMA	3.0	120	17
8	LiO*t*Bu (3.0)	DMA	3.0	120	25
9	NaO*t*Bu (3.0)	DMF	3.0	120	26
10	NaO*t*Bu (3.0)	DMSO	3.0	120	72
11	NaO*t*Bu (3.0)	Me-Cy	3.0	120	68
12	NaO*t*Bu (3.0)	MTBE	3.0	120	69
13	NaO*t*Bu (3.0)	toluene	3.0	120	56
14	NaO*t*Bu (3.0)	1,4-dioxane	3.0	120	50
15	NaO*t*Bu (1.5)	DMA	1.5	120	47
16	NaO*t*Bu (2.0)	DMA	2.0	120	67
17	NaO*t*Bu (2.5)	DMA	2.5	120	76
18	NaO*t*Bu (3.5)	DMA	3.5	120	83
19	NaO*t*Bu (3.0)	DMA	3.0	80	61
20	NaO*t*Bu (3.0)	DMA	3.0	90	81
21	NaO*t*Bu (3.0)	DMA	3.0	100	86

22	NaO^tBu (3.0)	DMA	3.0	110	89
23[c]	NaO^tBu (3.0)	DMA	3.0	120	83
24[d]	NaO^tBu (3.0)	DMA	3.0	120	0
25[e]	NaO^tBu (3.0)	DMA	3.0	120	13

[a]Reaction conditions: **1a-2** (0.5 mmol, 1.0 equiv.) in solvent (0.5 M) for 5 h unless otherwise stated. The yields were determined by GC-MS analysis vs. a calibrated internal standard and are averages of two runs. [b]Isolated yield. [c]3 h. [d]B_2pin_2 instead of B_2neop_2. [e]B_2cat_2 instead of B_2neop_2. DMA = *N,N*-dimethylacetamide.

General procedure of optimization for Table S2. In an argon-filled glovebox, the alkyl aryl sulfone **2a** (0.5 mmol, 1.0 equiv.), dissolved in solvent (1 mL), was added to a 10 mL thick-walled reaction tube equipped with a magnetic stirring bar. Base and the boron source were added. The reaction mixture was stirred at indicated temperature for 2 h, then diluted with Et_2O (2 mL) and filtered through a pad of Celite (Ø 3 mm x 8 mm). *n*-Dodecane was added as an internal standard and crude reaction mixture was analysed by GC-MS.

Table S2: Optimization of reaction conditions for the borylation of (benzylsulfonyl)benzene **2a**.

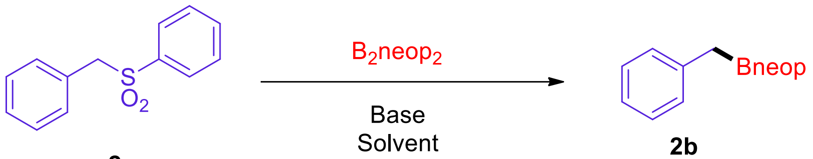

Entry	Base (eq.)	Solvent	B_2neop_2 (eq.)	T (°C)	Yield of **2b** (%)[a]
1	NaO^{t}Bu (3.0)	DMA	3.0	120	24
2	NaO^{t}Bu (1.5)	toluene	1.5	110	57
3	NaO^{t}Bu (1.5)	THF	1.5	110	66
4	NaO^{t}Bu (1.5)	MTBE	1.5	110	49
5	NaO^{t}Bu (1.5)	Me-Cy	1.5	110	53
6	NaO^{t}Bu (1.5)	1,4-dioxane	1.5	110	71
7	NaO^{t}Bu (1.5)	hexane	1.5	110	52
8	KO^{t}Bu (1.5)	1,4-dioxane	1.5	110	70
9	LiO^{t}Bu (1.5)	1,4-dioxane	1.5	110	67
10	LiOMe (1.5)	1,4-dioxane	1.5	110	36
11	KOMe (1.5)	1,4-dioxane	1.5	110	93
12	NaOMe (1.5)	1,4-dioxane	1.5	110	0
13	NaOAc (1.5)	1,4-dioxane	1.5	110	0
14	K_3PO_4 (1.5)	1,4-dioxane	1.5	110	0
15	KF (1.5)	1,4-dioxane	1.5	110	0
16	CsF (1.5)	1,4-dioxane	1.5	100	62
17	KOMe (1.5)	1,4-dioxane	1.5	60	10
18	KOMe (1.5)	1,4-dioxane	1.5	80	78
19	KOMe (1.5)	1,4-dioxane	1.5	100	90
21[b]	KOMe (1.1)	1,4-dioxane	1.1	110	73
22[c]	KOMe (1.2)	1,4-dioxane	1.2	110	93 (72)[e]
23[d]	KOMe (1.2)	1,4-dioxane	-	110	0

[a]Reaction conditions: **2a** (0.5 mmol, 1.0 equiv.) in solvent (1.0 M) for 2 h unless otherwise stated. The yields were determined by GC-MS analysis vs. a calibrated internal standard and are averages of two runs. [b]1.1 equiv. KOMe and 1.1 equiv. B_2neop_2 were added. [c]1.2 equiv. KOMe and 1.2 equiv. B_2neop_2 were added. [d]1.2 equiv. B_2pin_2 was added. [e]isolated yield.

1.3 Synthesis of Sulfone Substrates

1-Phenyl-5-({3-phenylpropyl}sulfonyl)-1*H*-tetrazole 1a-1

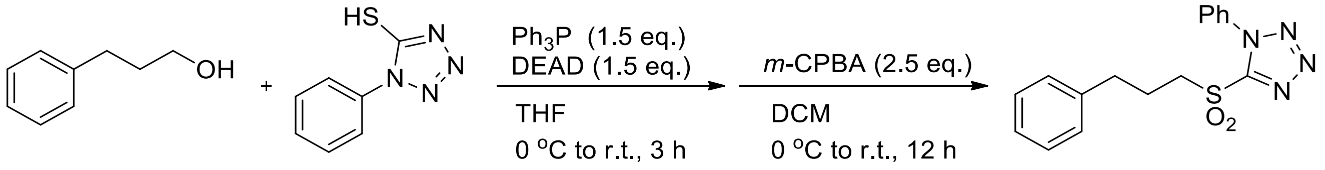

1a-1

Compound **1a-1** was synthesized according to reported literature.[1] A glass tube was charged with alcohol (0.545 g, 4.0 mmol), PPh_3 (1.574 g, 6.0 mmol) and 1-phenyl-1*H*-tetrazole-5-thiol (1.069 g, 6.0 mmol) in THF (15 mL). DEAD (1.10 mL, 6.0 mmol) was slowly added at 0 °C to the solution, and mixture was stirred at room temperature for 3 h before the solvent was evaporated. The crude aryl sulfide was dissolved in DCM (12 mL) in an ice-water bath before *m*-CPBA (contains *ca*. 23 wt%, 10.0 mmol, 2.24 g) was added in portions. The mixture was allowed to warm to room temperature. After 12 h, saturated aqueous Na_2CO_3 was added, and the resulting solution was extracted with EtOAc (3 x 30 mL). The combined organic layer was dried over Na_2SO_4 and filtered through a pad of Celite (Ø 3 mm x 8 mm). The product was isolated by flash column chromatography (hexane/ethyl acetate: 10/1) to afford compound **1a-1**.

Yield: 960 mg (2.92 mmol, 73%) of a white solid. **^{1}H NMR** (400 MHz, $CDCl_3$): δ = 7.68 – 7.57 (m, 5H), 7.32 (t, *J* = 7 Hz, 2H), 7.26 – 7.22 (m, 1H), 7.20 – 7.18 (m, 2H), 3.73 – 3.69 (m, 2H), 2.84 (t, *J* = 7 Hz, 2H), 2.35 – 2.27 (m, 2H). **^{13}C{^{1}H} NMR** (100 MHz, $CDCl_3$): δ = 153.5, 139.3, 133.1, 131.6, 129.9, 128.9, 128.6, 126.9, 125.2, 55.3, 34.0, 23.7. **HRMS-ASAP** (m/z): Calculated (found) for $C_{16}H_{17}N_4O_2S$ $[M+H]^+$ 329.1067 (329.1063).

The spectroscopic data for **1a-1** match those reported in the literature.[1]

General procedure 1:

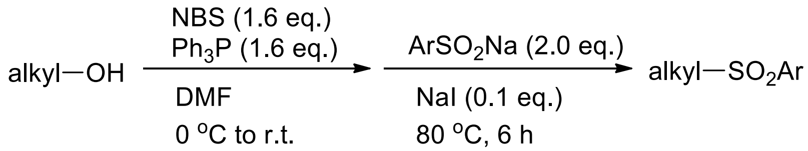

This method was based on the literature.[2] The alkyl alcohol (3.0 mmol), Ph_3P (4.8 mmol) and anhydrous DMF (10 mL) were added to a Schlenk flask equipped with a magnetic stirring bar at 0 °C under Ar. NBS (4.8 mmol) was added in small portions over 15 min. The reaction mixture was stirred while worming from 0 °C to r.t. over 30 min. To this solution was added a mixture of $PhSO_2Na$ (6.0 mmol) and NaI (0.3 mmol) in 3 portions over 10 min. The mixture was stirred for 6 h at 80 °C, then

diluted with EtOAc (20 mL) and 3% aq $Na_2S_2O_3$ (20 mL). The layers were separated and the aqueous phase was extracted with EtOAc (2 x 30 mL). The organic layer was washed with water and brine, and then dried over anhydrous Na_2SO_4, filtered and the solvent removed under vacuum. The crude product was purified by recrystallization or flash column chromatography on silica gel with hexane/EtOAc to afford the sulfone.

({3-Phenylpropyl}sulfonyl)benzene 1a-2

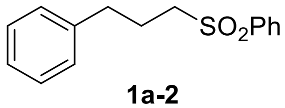

1a-2

According to **General procedure 1** with 3-phenyl-1-propanol (409 mg, 3.0 mmol, 1.0 equiv.) and $PhSO_2Na$ (985 mg, 6.0 mmol, 2.0 equiv.), the reaction mixture was purified by recrystallization (hexane/EtOAc) to yield the product **1a-2** as a white solid (633 mg, 2.43 mmol, 81% yield). **^{1}H NMR** (400 MHz, $CDCl_3$): δ = 7.90 – 7.87 (m, 2H), 7.68 – 7.64 (m, 1H), 7.58 – 7.54 (m, 2H), 7.29 – 7.25 (m, 2H), 7.22 – 7.18 (m, 1H), 7.11 – 7.09 (m, 2H), 3.10 – 3.06 (m, 2H), 2.70 (t, *J* = 7 Hz, 2H), 2.09 – 2.01 (m, 2H). **^{13}C{^{1}H} NMR** (100 MHz, $CDCl_3$): δ = 139.9, 139.0, 133.8, 129.4, 128.7, 128.5, 128.1, 126.5, 55.5, 34.2, 24.3. **HRMS-ASAP** (m/z): Calculated (found) for $C_{15}H_{17}O_2S$ $[M+H]^+$ 261.0944 (261.0943).

The spectroscopic data for **1a-2** match those reported in the literature.[4]

1-Fluoro-4-({3-phenylpropyl}sulfonyl)benzene 1a-3

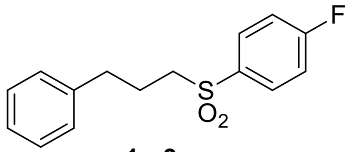

1a-3

According to **General procedure 1** with 3-phenyl-1-propanol (409 mg, 3.0 mmol, 1.0 equiv.) and 4-$FPhSO_2Na$ (1.093 g, 6.0 mmol, 2.0 equiv.), the reaction mixture was purified by recrystallization (hexane/EtOAc) to yield the product **1a-3** as a white solid (559 mg, 2.01 mmol, 67% yield). **^{1}H NMR** (400 MHz, $CDCl_3$): δ = 7.93 – 7.88 (m, 2H), 7.31 – 7.19 (m, 5H), 7.12 – 7.10 (m, 2H), 3.10 – 3.06 (m, 2H), 2.71 (t, *J* = 7 Hz, 2H), 2.09 – 2.01 (m, 2H). **^{13}C{^{1}H} NMR** (100 MHz, $CDCl_3$): δ = 165.9 (d, *J* = 254 Hz), 139.8, 135.1 (d, *J* = 3 Hz), 131.0 (d, *J* = 9 Hz), 128.7, 128.5, 126.6, 116.7 (d, *J* = 23 Hz), 55.6, 34.1, 24.4. **^{19}F{^{1}H} NMR** (376 MHz, $CDCl_3$): δ = -103.3 (s). **HRMS-ASAP** (m/z): Calculated (found) for $C_{15}H_{16}FO_2S$ $[M+H]^+$ 279.0850 (279.0848).

1-Methyl-4-({3-phenylpropyl}sulfonyl)benzene 1a-6

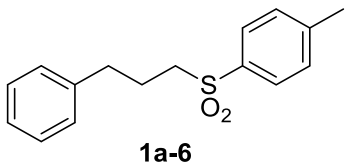

1a-6

According to **General procedure 1** with 3-phenyl-1-propanol (409 mg, 3.0 mmol, 1.0 equiv.) and 4-MePhSO_2Na (1.069 g, 6.0 mmol, 2.0 equiv.), the reaction mixture was purified by recrystallization (hexane/EtOAc) to yield the product **1a-6** as a white solid (633 mg, 2.31 mmol, 77% yield). **^{1}H NMR** (400 MHz, $CDCl_3$): δ = 7.76 (d, J = 8 Hz, 2H), 7.34 (d, J = 8 Hz, 2H), 7.27 (dd, J = 6, 9 Hz, 2H), 7.20 (dd, J = 6, 9 Hz, 1H), 7.10 (dd, J = 7 Hz, 2H), 3.08 – 3.04 (m, 2H), 2.69 (t, J = 7 Hz, 2H), 2.45 (s, 3H), 2.07 – 1.99 (m, 2H). **^{13}C{^{1}H} NMR** (100 MHz, $CDCl_3$): δ = 144.8, 140.0, 136.1, 130.0, 128.7, 128.5, 128.2, 126.5, 55.6, 34.2, 24.4, 21.8. **HRMS-ASAP** (m/z): Calculated (found) for $C_{16}H_{19}O_2S$ $[M+H]^+$ 275.1100 (275.1098).

The spectroscopic data for **1a-6** match those reported in the literature.[3]

General procedure 2:

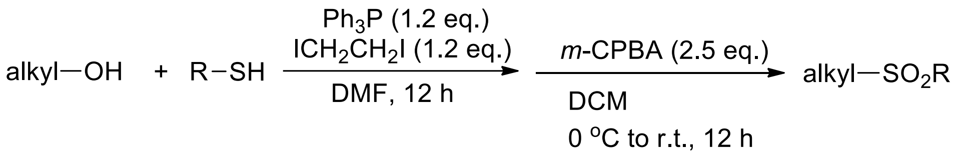

This method was based on the literature.[5] The alkyl alcohol (3.0 mmol), Ph_3P (3.6 mmol) and anhydrous DMF (10 mL) were added to a Schlenk flask equipped with a magnetic stirring bar under Ar. 1,2-Diiodoethane (3.6 mmol) was then added and the mixture was stirred for 2 min until the 1,2-diiodoethane was completely dissolved. Thiol (9.0 mmol) was added subsequently and the mixture was stirred at room temperature for 12 h, then diluted with DCM (20 mL). The mixture was washed with water (3 x 20 mL), the combined organic phases were dried over anhydrous Na_2SO_4, filtered and the solvent was removed under vacuum.

The crude aryl sulfide was dissolved in DCM (10 mL) in an ice-water bath before *m*-CPBA (contains *ca*. 23 wt%, 7.5 mmol, 1.68 g) was added in portions. The mixture was allowed to warm to room temperature. After 12 h, saturated aqueous Na_2CO_3 was added and the resulting solution was extracted with EtOAc (3 x 20 mL). The combined organic layer was dried over Na_2SO_4, and filtered through a pad of Celite (Ø 3 mm x 8 mm). The product was purified by flash column chromatography (hexane/ethyl acetate: 10/1).

1-({3-Phenylpropyl}sulfonyl)-4-(trifluoromethyl)benzene 1a-4

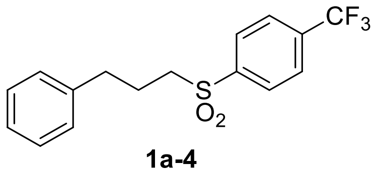

1a-4

According to **General procedure 2** with 3-phenyl-1-propanol (409 mg, 3.0 mmol, 1.0 equiv.) and 4-CF_3PhSH (1.603 g, 9.0 mmol, 3.0 equiv.), the reaction mixture was purified by column chromatography on silica gel (hexane/EtOAc = 10/1) to yield the product **1a-4** as a white solid (630 mg, 1.92 mmol, 64% yield). **^{1}H NMR** (500 MHz, $CDCl_3$): δ = 8.02 (d, J = 8 Hz, 2H), 7.83 (d, J = 8 Hz, 2H), 7.27 (t, J = 7 Hz, 2H), 7.21 (dd, J = 6, 9 Hz, 1H), 7.10 (d, J = 7 Hz, 2H), 3.11 – 3.08 (m, 2H), 2.72 (t, J = 7 Hz, 2H), 2.10 – 2.03 (m, 2H). **^{13}C{^{1}H} NMR** (125 MHz, $CDCl_3$): δ = 142.7, 139.7, 135.6 (q, J = 33 Hz), 128.9, 128.8, 128.5, 126.7, 126.6 (q, J = 4 Hz), 123.2 (q, J = 273 Hz), 55.4, 34.2, 24.2. **^{19}F{^{1}H} NMR** (376 MHz, $CDCl_3$): δ = -63.2 (s). **HRMS-ASAP** (m/z): Calculated (found) for $C_{16}H_{16}F_3O_2S$ $[M+H]^+$ 329.0818 (329.0814).

The spectroscopic data for **1a-4** match those reported in the literature.[3]

1-({3-Phenylpropyl}sulfonyl)-3,5-bis(trifluoromethyl)benzene 1a-5

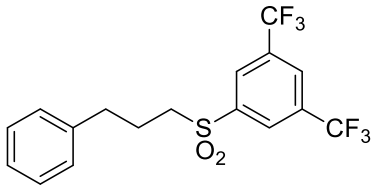

1a-5

According to **General procedure 2** with 3-phenyl-1-propanol (409 mg, 3.0 mmol, 1.0 equiv.) and 3,5-CF_3PhSH (2.215 g, 9.0 mmol, 3.0 equiv.), the reaction mixture was purified by column chromatography on silica gel (hexane/EtOAc = 10/1) to yield the product **1a-5** as a white solid (654 mg, 1.65 mmol, 55% yield). **^{1}H NMR** (500 MHz, $CDCl_3$): δ = 8.33 (s, 2H), 8.15 (s, 1H), 7.30 – 7.27 (m, 2H), 7.24 – 7.20 (m, 1H), 7.11 – 7.09 (m, 2H), 3.15 – 3.12 (m, 2H), 2.76 (t, J = 8 Hz, 2H), 2.15 – 2.09 (m, 2H). **^{13}C{^{1}H} NMR** (120 MHz, $CDCl_3$): δ = 142.2, 139.3, 133.5 (q, J = 34 Hz), 128.9, 128.6 (q, J = 4 Hz), 128.5, 127.6, 126.9, 122.5 (q, J = 273 Hz), 55.4, 34.0, 23.9. **^{19}F{^{1}H} NMR** (470 MHz, $CDCl_3$): δ = -62.9 (s). **HRMS-ASAP** (m/z): Calculated (found) for $C_{17}H_{15}F_6O_2S$ $[M+H]^+$ 397.0691 (397.0687).

1-Methoxy-4-({3-phenylpropyl}sulfonyl)benzene 1a-7

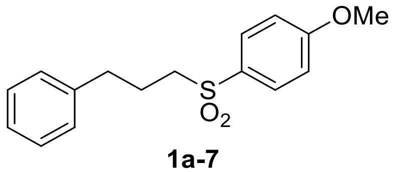

1a-7

According to **General procedure 2** with 3-phenyl-1-propanol (409 mg, 3.0 mmol, 1.0 equiv.) and 4-OMePhSH (1.262 g, 9.0 mmol, 3.0 equiv.), the reaction mixture was purified by column chromatography on silica gel (hexane/EtOAc = 10/1) to yield the product **1a-7** as a white solid (633 mg, 2.31 mmol, 77% yield). **^{1}H NMR** (400 MHz, $CDCl_3$): δ = 7.80 (d, J = 9 Hz, 2H), 7.27 (dd, J = 6, 9 Hz, 2H), 7.20 (dd, J = 6, 9 Hz, 1H), 7.12 – 7.10 (m, 2H), 7.00 (d, J = 9 Hz, 2H), 3.87 (s, 3H), 3.08 – 3.04 (m, 2H), 2.69 (t, J = 7 Hz, 2H), 2.07 – 1.99 (m, 2H). **^{13}C{^{1}H} NMR** (100 MHz, $CDCl_3$): δ = 163.7, 140.0, 130.5, 130.2, 128.6, 128.4, 126.4, 114.5, 55.77, 55.75, 34.1, 24.5. **HRMS-ASAP** (m/z): Calculated (found) for $C_{16}H_{19}O_3S$ $[M+H]^+$ 291.1049 (291.1044).

The spectroscopic data for **1a-7** match those reported in the literature.[3]

(3-{Ethylsulfonyl}propyl)benzene 1a-8

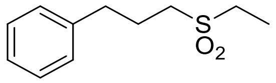

1a-8

According to **General procedure 2** with 3-phenyl-1-propanol (409 mg, 3.0 mmol, 1.0 equiv.) and ethanethiol (559 mg, 9.0 mmol, 3.0 equiv.), the reaction mixture was purified by column chromatography on silica gel (hexane/EtOAc = 10/1) to yield the product **1a-8** as a white solid (535 mg, 2.52 mmol, 84%). **^{1}H NMR** (400 MHz, $CDCl_3$): δ = 7.32 – 7.29 (m, 2H), 7.24 – 7.17 (m, 3H), 2.98 – 2.90 (m, 4H), 2.78 (t, J = 7 Hz, 2H), 2.21 – 2.13 (m, 2H), 1.34 (t, J = 7 Hz, 3H). **^{13}C{^{1}H} NMR** (100 MHz, $CDCl_3$): δ = 139.9, 128.7, 128.5, 126.6, 50.9, 47.1, 34.3, 23.4, 6.62. **HRMS-ASAP** (m/z): Calculated (found) for $C_{11}H_{17}O_2S$ $[M+H]^+$ 213.0944 (213.0938).

The spectroscopic data for **1a-8** match those reported in the literature.[4]

General procedure 3:

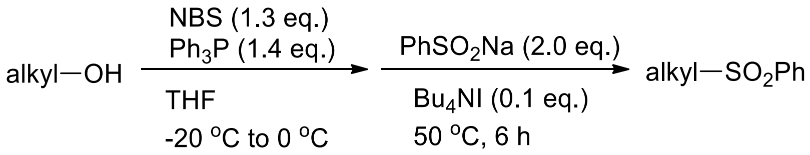

This method was according to reported literature.[2] Alkyl alcohol (3.0 mmol), Ph_3P (4.2 mmol) and THF (10 mL) were added to a Schlenk flask equipped with a magnetic stirring bar at –20 °C under Ar. NBS (3.9 mmol) was added in small portions over 15 min. The reaction mixture was stirred while warming from –20 °C to 0 °C for 30 min. A mixture of $PhSO_2Na$ (6.0 mmol) and Bu_4NI (0.3 mmol) was added in 3 portions over 10 min to this solution. The mixture was stirred for 6 h at 50 °C, then diluted with EtOAc (20 mL) and 3% aqueous $Na_2S_2O_3$ (20 mL). The layers were separated and the aqueous phase was extracted with EtOAc (2 x 30 mL). The organic layer was washed with water and brine, and then dried over anhydrous Na_2SO_4, filtered and the solvent was removed under vacuum. The crude product was purified by recrystallization or flash column chromatography on silica gel with hexane/EtOAc to afford the sulfone.

1-Methyl-4-({phenylsulfonyl}methyl)benzene 3a

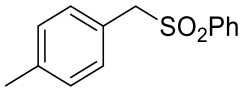

3a

According to **General procedure 3** with *p*-tolylmethanol (366 mg, 3.0 mmol, 1.0 equiv.), the reaction mixture was purified by recrystallization (hexane/EtOAc) to yield the product **3a** as a white solid (672 mg, 2.73 mmol, 91% yield). **^{1}H NMR** (400 MHz, $CDCl_3$): δ = 7.63 (d, J = 8 Hz, 2H), 7.60 (t, J = 8 Hz, 1H), 7.44 (t, J = 8 Hz, 2H), 7.06 (d, J = 8 Hz, 2H), 6.95 (d, J = 8 Hz, 2H), 4.27 (s, 2H), 2.31 (s, 3H). **^{13}C{^{1}H} NMR** (100 MHz, $CDCl_3$): δ = 138.8, 137.9, 133.8, 130.7, 129.3, 128.9, 128.7, 124.9, 62.6, 21.3. **HRMS-ASAP** (m/z): Calculated (found) for $C_{14}H_{15}O_2S$ $[M+H]^+$ 247.0787 (247.0785).

The spectroscopic data for **3a** match those reported in the literature.[6]

1-Methoxy-4-({phenylsulfonyl}methyl)benzene 4a

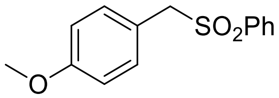

4a

According to **General procedure 3** with (4-methoxyphenyl)methanol (414 mg, 3.0 mmol, 1.0 equiv.), the reaction mixture was purified by recrystallization (hexane/EtOAc) to yield the product **4a** as a white solid (684 mg, 2.61 mmol, 87% yield). **^{1}H NMR** (400 MHz, $CDCl_3$): δ = 7.63 (d, J = 7 Hz, 2H), 7.60 (t, J = 8 Hz, 1H), 7.45 (t, J = 8 Hz, 2H), 6.99 (d, J = 9 Hz, 2H), 6.78 (d, J = 9 Hz, 2H), 4.25 (s, 2H), 3.78 (s, 3H). **^{13}C{^{1}H} NMR** (100 MHz, $CDCl_3$): δ = 160.0, 137.9, 133.8, 132.1, 129.0, 128.7, 120.0, 114.1, 62.3, 55.4. **HRMS-ASAP** (m/z): Calculated (found) for $C_{14}H_{15}O_3S$ $[M+H]^+$ 263.0736 (263.0728).

The spectroscopic data for **4a** match those reported in the literature.[6]

1-({Phenylsulfonyl}methyl)-4-(trifluoromethoxy)benzene 5a

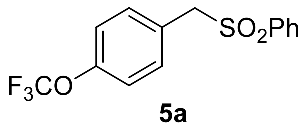

5a

According to **General procedure 3** with (4-(trifluoromethoxy)phenyl)methanol (576 mg, 3.0 mmol, 1.0 equiv.), the reaction mixture was purified by recrystallization (hexane/EtOAc) to yield the product **5a** as a white solid (692 mg, 2.19 mmol, 73% yield). ^{1}**H NMR** (500 MHz, $CDCl_3$): δ = 7.66 – 7.61 (m, 3H), 7.47 (t, J = 8 Hz, 2H), 7.14 – 7.10 (m, 4H), 4.30 (s, 2H). ^{13}**C{^{1}H} NMR** (125 MHz, $CDCl_3$): δ = 149.8 (q, J = 3 Hz), 137.8, 134.1, 132.5, 129.2, 128.7, 127.0, 121.1, 120.5 (q, J = 256 Hz), 62.2. ^{19}**F{^{1}H} NMR** (470 MHz, $CDCl_3$): δ = -57.9 (s). **HRMS-ASAP** (m/z): Calculated (found) for $C_{14}H_{12}F_3O_3S$ $[M+H]^+$ 317.0454 (317.0449).

The spectroscopic data for **5a** match those reported in the literature.[7]

1-Fluoro-4-({phenylsulfonyl}methyl)benzene 6a

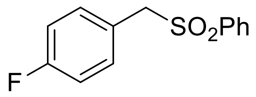

6a

According to **General procedure 3** with (4-fluorophenyl)methanol (378 mg, 3.0 mmol, 1.0 equiv.), the reaction mixture was purified by recrystallization (hexane/EtOAc) to yield the product **6a** as a white solid (630 mg, 2.52 mmol, 84% yield). ^{1}**H NMR** (400 MHz, $CDCl_3$): δ = 7.65 – 7.60 (m, 3H), 7.47 (t, J = 8 Hz, 2H), 7.08 – 7.03 (m, 2H), 6.95 (t, J = 8 Hz, 2H), 4.28 (s, 2H). ^{13}**C{^{1}H} NMR** (100 MHz, $CDCl_3$): δ = 163.1 (d, J = 247 Hz), 137.6, 134.0, 132.7 (d, J = 8 Hz), 129.1, 128.7, 124.0 (d, J = 3 Hz), 115.8 (d, J = 22 Hz), 62.1. ^{19}**F{^{1}H} NMR** (376 MHz, $CDCl_3$): δ = -112.3 (s). **HRMS-ASAP** (m/z): Calculated (found) for $C_{13}H_{12}FO_2S$ $[M+H]^+$ 251.0537 (251.0534).

The spectroscopic data for **6a** match those reported in the literature.[6]

1-Chloro-4-({phenylsulfonyl}methyl)benzene 7a

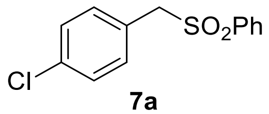

7a

According to **General procedure 3** with (4-chlorophenyl)methanol (428 mg, 3.0 mmol, 1.0 equiv.), the reaction mixture was purified by recrystallization (hexane/EtOAc) to yield the product **7a** as a white

solid (664 mg, 2.49 mmol, 83% yield). **^{1}H NMR** (400 MHz, $CDCl_3$): δ = 7.66 – 7.60 (m, 3H), 7.47 (t, J = 8 Hz, 2H), 7.23 (d, J = 8 Hz, 2H), 7.01 (d, J = 8 Hz, 2H), 4.27 (s, 2H). **^{13}C{^{1}H} NMR** (100 MHz, $CDCl_3$): δ = 137.7, 135.1, 134.1, 132.2, 129.2, 128.9, 128.7, 126.7, 62.2. **HRMS-ASAP** (m/z): Calculated (found) for $C_{13}H_{12}ClO_2S$ $[M+H]^+$ 267.0241 (267.0235).

The spectroscopic data for **7a** match those reported in the literature.[7]

1-Bromo-4-({phenylsulfonyl}methyl)benzene 8a

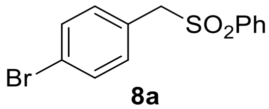

8a

According to **General procedure 3** with (4-bromophenyl)methanol (561 mg, 3.0 mmol, 1.0 equiv.), the reaction mixture was purified by recrystallization (hexane/EtOAc) to yield the product **8a** as a white solid (644 mg, 2.07 mmol, 69% yield). **^{1}H NMR** (400 MHz, $CDCl_3$): δ = 7.66 – 7.61 (m, 3H), 7.48 (t, J = 8 Hz, 2H), 7.39 (d, J = 8 Hz, 2H), 6.95 (d, J = 8 Hz, 2H), 4.26 (s, 2H). **^{13}C{^{1}H} NMR** (100 MHz, $CDCl_3$): δ = 137.6, 134.1, 132.5, 131.9, 129.2, 128.7, 127.2, 123.4, 62.3. **HRMS-ASAP** (m/z): Calculated (found) for $C_{13}H_{12}BrO_2S$ $[M+H]^+$ 310.9736 (310.9730).

The spectroscopic data for **8a** match those reported in the literature.[7]

({2-Phenylethyl}sulfonyl)benzene 9a

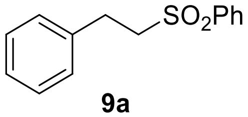

9a

According to **General procedure 1** with 2-phenylethanol (366 mg, 3.0 mmol, 1.0 equiv.), the reaction mixture was purified by recrystallization (hexane/EtOAc) to yield the product **9a** as a white solid (554 mg, 2.25 mmol, 75% yield). **^{1}H NMR** (400 MHz, $CDCl_3$): δ = 7.95 – 7.92 (m, 2H), 7.69 – 7.64 (m, 1H), 7.60 – 7.55 (m, 2H), 7.28 – 7.23 (m, 2H), 7.21 – 7.17 (m, 1H), 7.12 – 7.09 (m, 2H), 3.37 – 3.33 (m, 2H), 3.06 – 3.02 (m, 2H). **^{13}C{^{1}H} NMR** (100 MHz, $CDCl_3$): δ = 139.0, 137.5, 134.0, 129.5, 128.9, 128.4, 128.2, 127.0, 57.6, 28.8. **HRMS-ASAP** (m/z): Calculated (found) for $C_{14}H_{15}O_2S$ $[M+H]^+$ 247.0787 (247.0782).

The spectroscopic data for **9a** match those reported in the literature.[8]

(Octylsulfonyl)benzene 10a

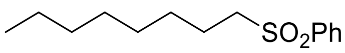

10a

According to **General procedure 1** with octan-1-ol (390 mg, 3.0 mmol, 1.0 equiv.), the reaction mixture was purified by column chromatography on silica gel (hexane/EtOAc = 10/1) to yield the product **10a** as a white solid (587 mg, 2.31 mmol, 77% yield). **^{1}H NMR** (300 MHz, $CDCl_3$): δ = 7.93 – 7.89 (m, 2H), 7.68 – 7.63 (m, 1H), 7.60 – 7.54 (m, 2H), 3.10 – 3.05 (m, 2H), 1.75 – 1.65 (m, 2H), 1.36 – 1.22 (m, 10H), 0.85 (t, J = 7 Hz, 3H). **^{13}C{^{1}H} NMR** (75 MHz, $CDCl_3$): δ = 139.4, 133.8, 129.4, 128.2, 56.5, 31.8, 29.1, 29.0, 28.4, 22.8, 22.7, 14.2. **HRMS-ASAP** (m/z): Calculated (found) for $C_{14}H_{23}O_2S$ $[M+H]^+$ 255.1413 (255.1409).

The spectroscopic data for **10a** match those reported in the literature.[9]

1-Methoxy-4-(3-{phenylsulfonyl}propyl)benzene 11a

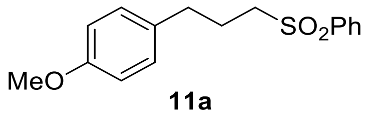

11a

According to **General procedure 1** with 3-(4-methoxyphenyl)propan-1-ol (499 mg, 3.0 mmol, 1.0 equiv.), the reaction mixture was purified by recrystallization (hexane/EtOAc) to yield the product **11a** as a white solid (653 mg, 2.25 mmol, 75% yield). **^{1}H NMR** (300 MHz, $CDCl_3$): δ = 7.91 – 7.87 (m, 2H), 7.68 – 7.62 (m, 1H), 7.59 – 7.53 (m, 2H), 7.02 (d, J = 9 Hz, 2H), 6.81 (d, J = 9 Hz, 2H), 3.78 (s, 3H), 3.08 – 3.03 (m, 2H), 2.64 (t, J = 6 Hz, 2H), 2.06 – 1.96 (m, 2H). **^{13}C{^{1}H} NMR** (75 MHz, $CDCl_3$): δ = 158.3, 139.3, 133.8, 132.0, 129.5, 129.4, 128.2, 114.1, 55.6, 55.4, 33.3, 24.5. **HRMS-ASAP** (m/z): Calculated (found) for $C_{16}H_{19}O_3S$ $[M+H]^+$ 291.1049 (291.1045).

The spectroscopic data for **11a** match those reported in the literature.[10]

1-Fluoro-4-(2-{phenylsulfonyl}ethyl)benzene 12a

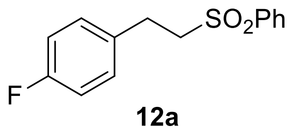

12a

According to **General procedure 1** with 2-(4-fluorophenyl)ethanol (420 mg, 3.0 mmol, 1.0 equiv.), the reaction mixture was purified by recrystallization (hexane/EtOAc) to yield the product **12a** as a white solid (554 mg, 2.13 mmol, 71% yield). **^{1}H NMR** (400 MHz, $CDCl_3$): δ = 7.94 – 7.91 (m, 2H), 7.69 – 7.65 (m, 1H), 7.60 – 7.55 (m, 2H), 7.10 – 7.05 (m, 2H), 6.97 – 6.91 (m, 2H), 3.35 – 3.31 (m, 2H), 3.05 – 3.01 (m, 2H). **^{13}C{^{1}H} NMR** (100 MHz, $CDCl_3$): δ = 161.9 (d, J = 244 Hz), 139.0, 134.0, 133.2 (d, J = 3 Hz), 129.9 (d, J = 8 Hz), 129.5, 128.2, 115.8 (d, J = 22 Hz), 57.6, 28.0. **^{19}F{^{1}H} NMR** (376 MHz, $CDCl_3$): δ = -115.6 (s). **HRMS-ASAP** (m/z): Calculated (found) for $C_{14}H_{14}FO_2S$ $[M+H]^+$ 265.0693 (265.0691).

The spectroscopic data for **12a** match those reported in the literature.[8]

2-(2-{Phenylsulfonyl}ethyl)-1,3-dioxane 14a

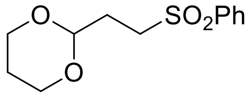

14a

According to **General procedure 1** with 2-(1,3-dioxan-2-yl)ethanol (396 mg, 3.0 mmol, 1.0 equiv.), the reaction mixture was purified by recrystallization (hexane/EtOAc) to yield the product **14a** as a white solid (599 mg, 2.34 mmol, 78% yield). **^{1}H NMR** (400 MHz, $CDCl_3$): *δ* = 7.91 – 7.88 (m, 2H), 7.67 – 7.62 (m, 1H), 7.58 – 7.53 (m, 2H), 4.62 (t, *J* = 4 Hz, 1H), 4.03 (dd, *J* = 5, 11 Hz, 2H), 3.70 (td, *J* = 2, 12 Hz, 2H), 3.26 – 3.22 (m, 2H), 2.02 – 1.91 (m, 3H), 1.33 – 1.28 (m, 1H). **^{13}C{^{1}H} NMR** (100 MHz, $CDCl_3$): *δ* = 138.9, 133.8, 129.4, 128.2, 99.3, 66.9, 51.1, 28.5, 25.6. **HRMS-ASAP** (m/z): Calculated (found) for $C_{12}H_{17}O_4S$ $[M+H]^+$ 257.0842 (257.0838).

The spectroscopic data for **14a** match those reported in the literature.[11]

Methyl 4-(phenylsulfonyl)butanoate 15a

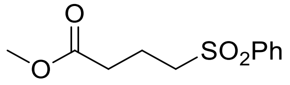

15a

According to **General procedure 1** with methyl 4-hydroxybutanoate (354 mg, 3.0 mmol, 1.0 equiv.), the reaction mixture was purified by column chromatography on silica gel (hexane/EtOAc = 5/1) to yield the product **15a** as a white solid (378 mg, 1.56 mmol, 52% yield). **^{1}H NMR** (300 MHz, $CDCl_3$): *δ* = 7.93 – 7.89 (m, 2H), 7.69 – 7.63 (m, 1H), 7.60 – 7.54 (m, 2H), 3.64 (s, 3H), 3.20 – 3.15 (m, 2H), 2.46 (t, *J* = 7 Hz, 2H), 2.07 – 1.98 (m, 2H). **^{13}C{^{1}H} NMR** (75 MHz, $CDCl_3$): *δ* = 172.6, 139.0, 133.9, 129.5, 128.2, 55.2, 51.9, 32.1, 18.4. **HRMS-ASAP** (m/z): Calculated (found) for $C_{11}H_{15}O_4S$ $[M+H]^+$ 243.0686 (243.0681).

The spectroscopic data for **14a** match those reported in the literature.[12]

({4-Methylpent-3-en-1-yl)sulfonyl)benzene 16a

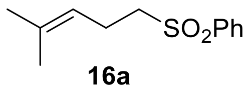

16a

According to **General procedure 3** with 4-methylpent-3-en-1-ol (300 mg, 3.0 mmol, 1.0 equiv.), the reaction mixture was purified by column chromatography on silica gel (hexane/EtOAc = 10/1) to yield the product **16a** as a white solid (430 mg, 1.92 mmol, 64% yield). **^{1}H NMR** (300 MHz, $CDCl_3$): *δ* = 7.94

– 7.90 (m, 2H), 7.69 – 7.63 (m, 1H), 7.60 – 7.54 (m, 2H), 5.00 – 4.93 (m, 1H), 3.11 – 3.05 (m, 2H), 2.40 (dd, *J* = 8, 16 Hz, 2H), 1.63 (d, *J* = 1 Hz, 3H), 1.54 (s, 3H). **^{13}C{^{1}H} NMR** (75 MHz, $CDCl_3$): δ = 139.4, 135.2, 133.8, 129.4, 128.2, 119.4, 56.1, 25.7, 21.8, 17.8. **HRMS-ASAP** (m/z): Calculated (found) for $C_{12}H_{17}O_2S$ $[M+H]^+$ 225.0944 (225.0939).

The spectroscopic data for **16a** match those reported in the literature.[13]

({5-fluoropentyl}sulfonyl)benzene 17a

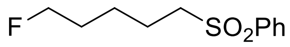

17a

According to **General procedure 1** with 5-fluoropentan-1-ol (318 mg, 3.0 mmol, 1.0 equiv.), the reaction mixture was purified by column chromatography on silica gel (hexane/EtOAc = 10/1) to yield the product **17a** as a white solid (497 mg, 2.16 mmol, 72% yield). **^{1}H NMR** (400 MHz, $CDCl_3$): δ = 7.92 – 7.89 (m, 2H), 7.69 – 7.64 (m, 1H), 7.60 – 7.55 (m, 2H), 4.46 (t, *J* = 6 Hz, 1H), 4.34 (t, *J* = 6 Hz, 1H), 3.12 – 3.08 (m, 2H), 1.80 – 1.60 (m, 4H), 1.53 – 1.45 (m, 2H). **^{13}C{^{1}H} NMR** (100 MHz, $CDCl_3$): δ = 139.1, 133.9, 129.4, 128.1, 83.5 (d, *J* = 164 Hz), 55.2, 29.9 (d, *J* = 19 Hz), 24.3 (d, *J* = 5 Hz), 22.4. **^{19}F{^{1}H} NMR** (376 MHz, $CDCl_3$): δ = -218.8 (s). **HRMS-ASAP** (m/z): Calculated (found) for $C_{11}H_{16}FO_2S$ $[M+H]^+$ 231.0850 (231.0844).

({6-Chlorohexyl}sulfonyl)benzene 18a

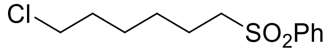

18a

According to **General procedure 1** with 6-chlorohexan-1-ol (410 mg, 3.0 mmol, 1.0 equiv.), the reaction mixture was purified by column chromatography on silica gel (hexane/EtOAc = 10/1) to yield the product **18a** as a white solid (594 mg, 2.28 mmol, 76% yield). **^{1}H NMR** (300 MHz, $CDCl_3$): δ = 7.93 – 7.89 (m, 2H), 7.69 – 7.63 (m, 1H), 7.60 – 7.54 (m, 2H), 3.52 – 3.34 (m, 2H), 3.11 – 3.06 (m, 2H), 1.86 – 1.68 (m, 4H), 1.48 – 1.33 (m, 4H). **^{13}C{^{1}H} NMR** (75 MHz, $CDCl_3$): δ = 139.3, 133.8, 129.4, 128.2, 56.2, 44.9, 32.2, 27.7, 26.4, 22.7. **HRMS-ASAP** (m/z): Calculated (found) for $C_{12}H_{18}ClO_2S$ $[M+H]^+$ 261.0711 (261.0708).

The spectroscopic data for **18a** match those reported in the literature.[14]

6-(Phenylsulfonyl)hexanenitrile 19a

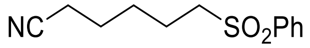

19a

According to **General procedure 1** with 6-hydroxyhexanenitrile (339 mg, 3.0 mmol, 1.0 equiv.), the reaction mixture was purified by column chromatography on silica gel (hexane/EtOAc = 10/1) to yield the product **19a** as a white solid (470 mg, 1.98 mmol, 66% yield). **^{1}H NMR** (300 MHz, $CDCl_3$): δ = 7.93 – 7.89 (m, 2H), 7.79 – 7.65 (m, 1H), 7.61 – 7.55 (m, 2H), 3.12 – 3.07 (m, 2H), 2.33 (t, J = 7 Hz, 2H), 1.81 – 1.51 (m, 6H). **^{13}C{^{1}H} NMR** (75 MHz, $CDCl_3$): δ = 139.2, 134.0, 129.5, 128.1, 119.3, 55.9, 27.4, 25.0, 22.1, 17.1. **HRMS-ASAP** (m/z): Calculated (found) for $C_{12}H_{16}NO_2S$ $[M+H]^+$ 238.0896 (238.0890).

9-(4-{Phenylsulfonyl}butyl)-9*H*-carbazole 20a

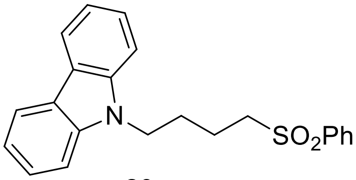

20a

According to **General procedure 1** with 4-(9*H*-carbazol-9-yl)butan-1-ol (718 mg, 3.0 mmol, 1.0 equiv.), the reaction mixture was purified by column chromatography on silica gel (hexane/EtOAc = 10/1) to yield the product **20a** as a white solid (806 mg, 2.22 mmol, 74% yield). **^{1}H NMR** (300 MHz, $CDCl_3$): δ = 8.12 – 8.08 (m, 2H), 7.67 – 7.63 (m, 2H), 7.52 – 7.43 (m, 5H), 7.38 – 7.35 (m, 2H), 7.26 – 7.21 (m, 2H), 4.32 (t, J = 7 Hz, 2H), 4.07 – 3.99 (m, 1H), 3.67 – 3.54 (m, 1H), 2.00 – 1.91 (m, 2H), 1.72 – 1.63 (m, 2H). **^{13}C{^{1}H} NMR** (75 MHz, $CDCl_3$): δ = 144.5, 140.4, 132.3, 129.2, 125.8, 125.3, 123.0, 120.5, 119.0, 108.7, 64.0, 42.5, 27.5, 25.6. **HRMS-ASAP** (m/z): Calculated (found) for $C_{22}H_{22}NO_2S$ $[M+H]^+$ 364.1366 (364.1362).

1-(4-{Phenylsulfonyl}butyl)-1*H*-indole 21a

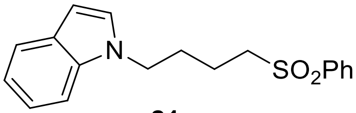

21a

According to **General procedure 1** with 4-(1*H*-indol-1-yl)butan-1-ol (568 mg, 3.0 mmol, 1.0 equiv.), the reaction mixture was purified by column chromatography on silica gel (hexane/EtOAc = 10/1) to yield the product **21a** as a white solid (667 mg, 2.13 mmol, 71% yield). **^{1}H NMR** (300 MHz, $CDCl_3$): δ = 7.82 – 7.78 (m, 2H), 7.66 – 7.60 (m, 1H), 7.54 – 7.48 (m, 2H), 7.28 – 7.25 (m, 1H), 7.22 – 7.16 (m, 1H), 7.13 – 7.07 (m, 1H), 7.00 (d, J = 3 Hz, 1H), 6.46 (dd, J = 3, 1 Hz, 1H), 4.12 (t, J = 7 Hz, 2H), 3.03 – 2.98 (m, 2H), 2.00 – 1.90 (m, 2H), 1.78 – 1.67 (m, 2H). **^{13}C{^{1}H} NMR** (75 MHz, $CDCl_3$): δ = 139.0, 135.9, 133.9, 129.4, 128.8, 128.1, 127.7, 121.7, 121.3, 119.6, 109.3, 101.6, 55.7, 45.8, 28.9, 20.6. **HRMS-ASAP** (m/z): Calculated (found) for $C_{18}H_{20}NO_2S$ $[M+H]^+$ 314.1209 (314.1207).

General procedure 4:

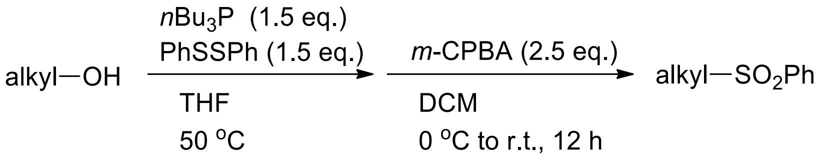

A glass tube was charged with alkyl alcohol (3.0 mmol, 1.0 equiv.), diphenyl disulfide (983 mg, 4.5 mmol, 1.5 equiv.) and *n*Bu$_3$P (1.12 mL, 4.5 mmol, 1.5 equiv.) in dry THF (10 mL) at room temperature, and the mixture was heated at 50 °C for 12 h. The mixture was quenched with 2N NaOH and extracted with EtOAc three times. The combined organic phase was washed with brine and concentrated under reduced pressure.

The crude aryl sulfide was dissolved in DCM (9 mL) in an ice-water bath before *m*-CPBA (*ca*. 23 wt%, 7.5 mmol, 1.68 g) was added in portions. The mixture was allowed to warm to room temperature. After 12 h, saturated aqueous Na_2CO_3 was added, and the resulting solution was extracted with EtOAc (3 x 30 mL). The combined organic layer was dried over Na_2SO_4 and filtered through a pad of Celite (Ø 3 mm x 8 mm). The crude product was purified by flash column chromatography on silica gel with hexane/EtOAc to afford the sulfone.

(Pentan-3-ylsulfonyl)benzene 22a

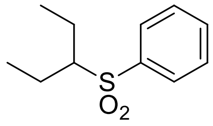

22a

According to **General procedure 4** with pentan-3-ol (264 mg, 3.0 mmol, 1.0 equiv.), the reaction mixture was purified by column chromatography on silica gel (hexane/EtOAc = 10/1) to yield the product **22a** as a white solid (350 mg, 1.65 mmol, 55% yield). **^{1}H NMR** (400 MHz, $CDCl_3$): δ = 7.88 – 7.86 (m, 2H), 7.65 – 7.61 (m, 1H), 7.56 – 7.52 (m, 2H), 2.82 – 2.76 (m, 1H), 1.91 – 1.81 (m, 2H), 1.72 – 1.61 (m, 2H), 0.98 (t, *J* = 8 Hz, 6H). **^{13}C{^{1}H} NMR** (100 MHz, $CDCl_3$): δ = 138.5, 133.6, 129.2, 128.8, 67.1, 20.3, 11.2. **HRMS-ASAP** (m/z): Calculated (found) for $C_{11}H_{17}O_2S$ $[M+H]^+$ 213.0944 (213.0939).

The spectroscopic data for **22a** match those reported in the literature.[15]

1-(Cyclopropylsulfonyl)-4-methylbenzene 23a

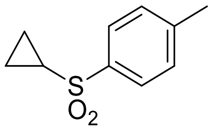

23a

According to **General procedure 4** with cyclopropanol (174 mg, 3.0 mmol, 1.0 equiv.) and 4-MePhSO$_2$Na (3.3 mmol, 1.1 equiv.), the reaction mixture was purified by column chromatography on silica gel (hexane/EtOAc = 10/1) to yield the product **23a** as a white solid (312 mg, 1.59 mmol, 53% yield). **^{1}H NMR** (400 MHz, CDCl$_3$): δ = 7.75 (d, J = 8 Hz, 2H), 7.33 (d, J = 8 Hz, 2H), 2.46 – 2.39 (m, 1H), 2.42 (s, 3H), 1.32 – 1.27 (m, 2H), 1.02 – 0.96 (m, 2H). **^{13}C{^{1}H} NMR** (100 MHz, CDCl$_3$): δ = 144.3, 137.7, 129.9, 127.6, 33.1, 21.7, 6.0. **HRMS-ASAP** (m/z): Calculated (found) for $C_{10}H_{13}O_2S$ [M+H]$^+$ 197.0631 (197.0627).

The spectroscopic data for **23a** match those reported in the literature.[16]

(Cyclobutylsulfonyl)benzene 24a

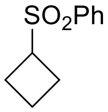

24a

According to **General procedure 4** with cyclobutanol (216 mg, 3.0 mmol, 1.0 equiv.), the reaction mixture was purified by column chromatography on silica gel (hexane/EtOAc = 10/1) to yield the product **24a** as a white solid (283 mg, 1.44 mmol, 48% yield). **^{1}H NMR** (400 MHz, CDCl$_3$): δ = 7.87 – 7.85 (m, 2H), 7.65 – 7.61 (m, 1H), 7.56 – 7.52 (m, 2H), 3.84 – 3.76 (m, 1H), 2.61 – 2.51 (m, 2H), 2.21 – 2.13 (m, 2H), 2.01 – 1.95 (m, 2H). **^{13}C{^{1}H} NMR** (100 MHz, CDCl$_3$): δ = 138.3, 133.7, 129.3, 128.4, 57.0, 22.9, 16.9. **HRMS-ASAP** (m/z): Calculated (found) for $C_{10}H_{13}O_2S$ [M+H]$^+$ 197.0631 (197.0627).

The spectroscopic data for **24a** match those reported in the literature.[17]

(Cyclopentylsulfonyl)benzene 25a

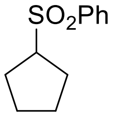

25a

According to **General procedure 4** with cyclopentanol (258 mg, 3.0 mmol, 1.0 equiv.), the reaction mixture was purified by column chromatography on silica gel (hexane/EtOAc = 10/1) to yield the product **25a** as a white solid (309 mg (1.47 mmol, 49% yield). **^{1}H NMR** (400 MHz, CDCl$_3$): δ = 7.91 – 7.89 (m, 2H), 7.66 – 7.62 (m, 1H), 7.58 – 7.53 (m, 1H), 3.53 – 3.45 (m, 1H), 2.11 – 2.02 (m, 2H), 1.91 – 1.82 (m, 2H), 1.81 – 1.71 (m, 2H), 1.64 – 1.54 (m, 2H). **^{13}C{^{1}H} NMR** (100 MHz, CDCl$_3$): δ = 139.0,

133.6, 129.3, 128.5, 64.2, 27.3, 26.0. **HRMS-ASAP** (m/z): Calculated (found) for $C_{11}H_{15}O_2S$ $[M+H]^+$ 211.0787 (211.0781).

The spectroscopic data for **25a** match those reported in the literature.[3]

(Cyclohexylsulfonyl)benzene 26a

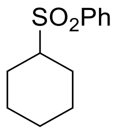

26a

According to **General procedure 4** with cyclohexanol (300 mg, 3.0 mmol, 1.0 equiv.), the reaction mixture was purified by column chromatography on silica gel (hexane/EtOAc = 10/1) to yield the product **26a** as a white solid (376 mg, 1.68 mmol, 56% yield). **^{1}H NMR** (300 MHz, $CDCl_3$): δ = 7.89 – 7.85 (m, 2H), 7.68 – 7.62 (m, 1H), 7.59 – 7.53 (m, 2H), 2.95 – 2.85 (m, 1H), 2.09 – 2.03 (m, 2H), 1.90 – 1.83 (m, 2H), 1.69 – 1.62 (m, 1H), 1.47 – 1.34 (m, 2H), 1.29 – 1.09 (m, 3H). **^{13}C{^{1}H} NMR** (75 MHz, $CDCl_3$): δ = 137.4, 133.7, 129.2, 129.1, 63.6, 25.6, 25.23, 25.19. **HRMS-ASAP** (m/z): Calculated (found) for $C_{12}H_{17}O_2S$ $[M+H]^+$ 225.0944 (225.0938).

The spectroscopic data for **26a** match those reported in the literature.[18]

(Cyclohex-2-en-1-ylsulfonyl)benzene 27a

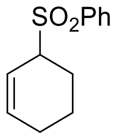

27a

According to **General procedure 4** with cyclohex-2-enol (294 mg, 3.0 mmol, 1.0 equiv.), the reaction mixture was purified by column chromatography on silica gel (hexane/EtOAc = 10/1) to yield the product **27a** as a colorless liquid (280 mg, 1.26 mmol, 42% yield). **^{1}H NMR** (300 MHz, $CDCl_3$): δ = 7.90 – 7.86 (m, 2H), 7.68 – 7.62 (m, 1H), 7.58 – 7.52 (m, 2H), 6.12 – 6.05 (m, 1H), 5.78 (d, J = 10 Hz, 1H), 3.80 – 3.72 (m, 1H), 2.01 – 1.72 (m, 5H), 1.56 – 1.43 (m, 1H). **^{13}C{^{1}H} NMR** (75 MHz, $CDCl_3$): δ = 137.5, 135.5, 133.8, 129.3, 129.1, 118.6, 61.9, 24.5, 22.8, 19.6. **HRMS-ASAP** (m/z): Calculated (found) for $C_{12}H_{15}O_2S$ $[M+H]^+$ 223.0787 (223.0786).

The spectroscopic data for **27a** match those reported in the literature.[18]

***tert*-Butyl 4-(phenylsulfonyl)piperidine-1-carboxylate 28a**

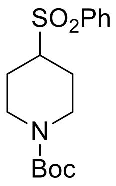

28a

According to **General procedure 4** with N-Boc 4-hydroxupiperidine (604 mg, 3.0 mmol, 1.0 equiv.), the reaction mixture was purified by column chromatography on silica gel (hexane/EtOAc = 10/1) to yield the product **28a** as a white solid (498 mg, 1.53 mmol, 51% yield). ^{1}**H NMR** (300 MHz, $CDCl_3$): δ = 7.89 – 7.85 (m, 2H), 7.71 – 7.65 (m, 1H), 7.61 – 7.55 (m, 2H), 4.22 (d, J = 13.5 Hz, 2H), 3.08 – 2.98 (m, 1H), 2.65 (t, J = 13 Hz, 2H), 1.97 (d, J = 13 Hz, 2H), 1.67 – 1.53 (m, 2H), 1.42 (s, 9H). ^{13}**C{^{1}H} NMR** (75 MHz, $CDCl_3$): δ = 154.5, 136.8, 134.1, 129.4, 129.2, 80.3, 61.9, 42.7, 28.5, 25.2. **HRMS-ASAP** (m/z): Calculated (found) for $C_{16}H_{24}NO_4S$ $[M+H]^+$ 326.1421 (326.1415).

The spectroscopic data for **28a** match those reported in the literature.[19]

<u>((1-Phenylpropan-2-yl)sulfonyl)benzene 29a</u>

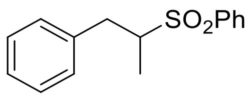

29a

According to **General procedure 4** with 1-phenylpropan-2-ol (408 mg, 3.0 mmol, 1.0 equiv.), the reaction mixture was purified by column chromatography on silica gel (hexane/EtOAc = 10/1) to yield the product **29a** as a white solid (421 mg, 1.62 mmol, 54% yield). ^{1}**H NMR** (400 MHz, $CDCl_3$): δ = 7.95 – 7.93 (m, 2H), 7.71 – 7.66 (m, 1H), 7.62 – 7.58 (m, 2H), 7.30 – 7.20 (m, 3H), 7.11 – 7.09 (m, 2H), 3.43 (dd, J = 3, 13 Hz, 1H), 3.31 – 3.22 (m, 1H), 2.54 (dd, J = 11, 13 Hz, 1H), 1.15 (d, J = 7 Hz, 3H). ^{13}**C{^{1}H} NMR** (100 MHz, $CDCl_3$): δ = 137.1, 137.0, 133.9, 129.3, 129.2, 129.1, 128.8, 127.0, 61.7, 35.4, 12.8. **HRMS-ASAP** (m/z): Calculated (found) for $C_{15}H_{17}O_2S$ $[M+H]^+$ 261.0944 (261.09337).

The spectroscopic data for **29a** match those reported in the literature.[20]

<u>1-Fluoro-4-(2-(phenylsulfonyl)propyl)benzene 30a</u>

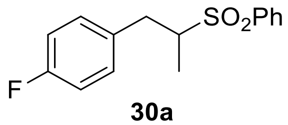

30a

According to **General procedure 4** with 1-(4-fluorophenyl)propan-2-ol (462 mg, 3.0 mmol, 1.0 equiv.), the reaction mixture was purified by column chromatography on silica gel (hexane/EtOAc = 10/1) to yield the product **30a** as a white solid (417 mg, 1.50 mmol, 50% yield). ^{1}**H NMR** (400 MHz, $CDCl_3$): δ = 7.95 – 7.92 (m, 2H), 7.71 – 7.66 (m, 1H), 7.62 – 7.57 (m, 2H), 7.08 – 7.04 (m, 2H), 6.99 – 6.94 (m, 2H),

3.40 (dd, J = 3, 13 Hz, 1H), 3.26 – 3.17 (m, 1H), 2.54 (dd, J = 11, 13 Hz, 1H), 1.14 (d, J = 7 Hz, 3H). **^{13}C{^{1}H} NMR** (100 MHz, $CDCl_3$): δ = 161.9 (d, J = 244 Hz), 137.1, 134.0, 132.7 (d, J = 3 Hz), 130.7 (d, J = 8 Hz), 129.4, 129.1, 115.7 (d, J = 22 Hz), 61.7 (d, J = 2 Hz), 34.6, 12.8. **^{19}F{^{1}H} NMR** (376 MHz, $CDCl_3$): δ = -115.6 (s). **HRMS-ASAP** (m/z): Calculated (found) for $C_{15}H_{16}FO_2S$ $[M+H]^+$ 279.0850 (279.0847).

1-Methoxy-4-(3-(phenylsulfonyl)butyl)benzene 31a

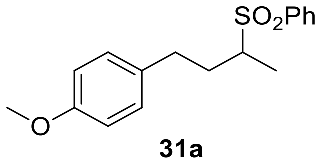

31a

According to **General procedure 4** with 1-methoxy-4-(3-(phenylsulfonyl)butyl)benzene (462 mg, 3.0 mmol, 1.0 equiv.), the reaction mixture was purified by column chromatography on silica gel (hexane/EtOAc = 10/1) to yield the product **31a** as a white solid (557 mg, 1.83 mmol, 61% yield). **^{1}H NMR** (400 MHz, $CDCl_3$): δ = 7.85 – 7.83 (m, 2H), 7.64 (t, J = 7 Hz, 1H), 7.56 – 7.52 (m, 2H), 7.01 (d, J = 8 Hz, 2H), 6.80 (d, J = 8 Hz, 2H), 3.78 (s, 3H), 3.06 – 2.97 (m, 1H), 2.79 – 2.72 (m, 1H), 2.56 – 2.49 (m, 1H), 2.32 – 2.23 (m, 1H), 1.73 – 1.63 (m, 1H), 1.30 (d, J = 7 Hz, 3H). **^{13}C{^{1}H} NMR** (100 MHz, $CDCl_3$): δ = 158.2, 137.3, 133.7, 132.2, 129.3, 129.2, 129.1, 114.1, 59.2, 55.4, 31.6, 31.0, 13.3. **HRMS-ASAP** (m/z): Calculated (found) for $C_{17}H_{21}O_3S$ $[M+H]^+$ 305.1206 (305.1203).

The spectroscopic data for **31a** match those reported in the literature.[21]

((9*Z*,12*Z*)-octadeca-9,12-dien-1-ylsulfonyl)benzene 32a

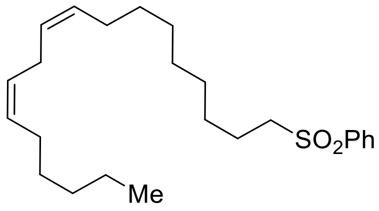

32a

According to **General procedure 3** with linolenyl alcohol (792 mg, 3.0 mmol, 1.0 equiv.), the reaction mixture was purified by column chromatography on silica gel (hexane/EtOAc = 10/1) to yield the product **32a** as a colorless oil (656 mg, 1.68 mmol, 56% yield). **^{1}H NMR** (400 MHz, $CDCl_3$): δ = 7.92 – 7.89 (m, 2H), 7.67 – 7.63 (m, 1H), 7.59 – 7.54 (m, 2H), 5.44 – 5.31 (m, 4H), 3.09 – 3.05 (m, 2H), 2.77 – 2.64 (m, 2H), 2.04 – 1.92 (m, 4H), 1.73 – 1.65 (m, 2H), 1.35 – 1.22 (m, 16H), 0.87 (t, J = 8 Hz, 3H). **^{13}C{^{1}H} NMR** (100 MHz, $CDCl_3$): δ = 139.2, 133.7, 131.2, 131.0, 129.4, 128.8, 128.6, 128.1, 56.4, 35.7, 32.64, 32.60, 31.5, 29.5, 29.3, 29.2, 29.1, 29.0, 28.3, 22.7, 22.6, 14.2. **HRMS-ASAP** (m/z): Calculated

(found) for $C_{24}H_{39}O_2S$ $[M+H]^+$ 391.2665 (391.2657). **Anal.** for $C_{24}H_{38}O_2S$ calcd: C, 73.79; H, 9.81; S, 8.21. found: C, 73.70; H, 9.69; S, 8.40.

The synthesis of substrate 33a

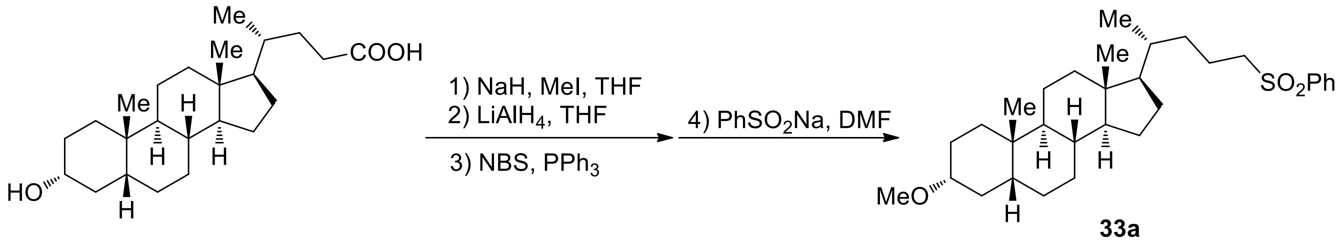

33a

NaH (9 mmol, 3.0 equiv., 60 % dispersion in mineral oil) was added at 0 °C in portions to a solution of hyodeoxycholic acid (3 mmol, 1.0 equiv.) in THF (30 mL, 0.1 M). The reaction mixture was stirred for 3 h at room temperature. Afterwards, MeI (6 mmol, 2 equiv.) was slowly added and the mixture was stirred at 45 ^oC for 24 hours. The reaction mixture was quenched with saturated aqueous NH_4Cl at 0 ^oC. The aqueous layer was extracted three times with EtOAc and the combined organic layers were washed with brine, dried over Na_2SO_4 and concentrated under reduced pressure.

$LiAlH_4$ (9 mmol, 3 equiv.) was added slowly at 0 °C to a solution of the crude product in 20 mL anhydrous THF. The mixture was allowed to warm to room temperature and stirred for another 4 h, then quenched with 1.0 M NaOH. We extracted the mixture three times with CH_2Cl_2 and the combined organic layer was washed with brine, dried over Na_2SO_4 and concentrated under reduced pressure. PPh_3 (1.2 equiv.) and NBS (1.2 equiv.) were added sequentially to a solution of the crude alcohol in 10 mL DCM in an ice bath. After 0.5 h stirring at room temperature, the reaction mixture was concentrated, and the crude material was purified by silica gel to deliver the primary bromide as a white solid.

The primary bromide (1.5 mmol, 1.0 equiv.) and $PhSO_2Na$ (2.25 mmol, 1.5 equiv.) was dissolved in 5 mL DMF. The mixture was stirred for 12 h at 85 °C, then diluted with EtOAc (20 mL) and 3% aqueous $Na_2S_2O_3$ (20 mL). The layers were separated and the aqueous phase was extracted with EtOAc (2 x 30 mL). The organic layer was washed with water and brine, and then dried over anhydrous Na_2SO_4, filtered, and the solvent was removed under vacuum. The crude product was purified by flash column chromatography on silica gel with hexane/EtOAc: 5/1 to afford **33a**.

(3*R*,5*R*,8*R*,9*S*,10*S*,13*R*,14*S*,17*R*)-3-methoxy-10,13-dimethyl-17-((*R*)-5-(phenylsulfonyl)pentan-2-yl)hexadecahydro-1*H*-cyclopenta[*a*]phenanthrene 33a

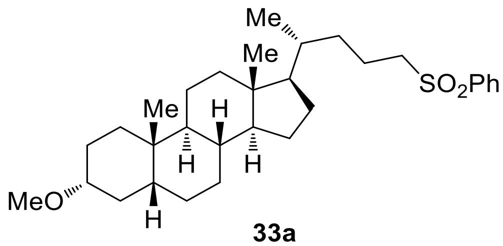

33a

Yield: 631 mg (1.26 mmol, 42%) of a white solid. **^{1}H NMR** (400 MHz, $CDCl_3$): δ = 7.93 – 7.90 (m, 2H), 7.68 – 7.64 (m, 1H), 7.60 – 7.55 (m, 2H), 3.34 (s, 3H), 3.19 – 2.97 (m, 3H), 1.93 – 1.49 (m, 9H), 1.43 – 0.95 (m, 19H), 0.90 (s, 3H), 0.85 (d, J = 6 Hz, 3H), 0.59 (s, 3H). **^{13}C{^{1}H} NMR** (100 MHz, $CDCl_3$): δ = 139.3, 133.8, 129.4, 128.2, 80.5, 56.8, 56.5, 56.0, 55.7, 42.8, 42.1, 40.4, 40.2, 35.9, 35.6, 35.4, 35.0, 34.6, 32.8, 28.4, 27.4, 26.9, 26.5, 24.3, 23.5, 20.9, 19.6, 18.4, 12.1. **HRMS-ASAP** (m/z): Calculated (found) for $C_{31}H_{49}O_3S$ $[M+H]^+$ 501.3397 (501.3389). **Anal.** for $C_{31}H_{48}O_3S$ calcd: C, 74.35; H, 9.66; S, 6.40. found: C, 74.51; H, 9.71; S, 6.28.

(3*S*,8*S*,9*S*,10*R*,13*R*,14*S*,17*R*)-10,13-dimethyl-17-((*R*)-6-methylheptan-2-yl)-3-(phenylsulfonyl)-2,3,4,7,8,9,10,11,12,13,14,15,16,17-tetradecahydro-1*H*-cyclopenta[*a*]phenanthrene 34a

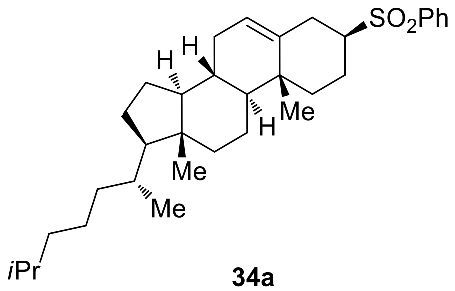

34a

According to **General procedure 4** with cholesterol (1.16 g, 3.0 mmol, 1.0 equiv.), the reaction mixture was purified by column chromatography on silica gel (hexane/EtOAc = 5/1) to yield the product **34a** as a white solid (429 mg, 0.84 mmol, 28% yield). **^{1}H NMR** (400 MHz, $CDCl_3$): δ = 7.73 – 7.71 (m, 2H), 7.54 – 7.52 (m, 3H), 5.41 – 5.25 (m, 1H), 4.25 – 4.17 (m, 1H), 2.50 – 2.18 (m, 2H), 2.06 – 1.69 (m, 6H), 1.60 – 1.23 (m, 10H), 1.21 – 0.91 (m, 10H), 0.99 (s, 3H), 0.90 (d, J = 7 Hz, 3H), 0.86 (dd, J = 2, 7 Hz, 6H), 0.66 (s, 3H). **^{13}C{^{1}H} NMR** (100 MHz, $CDCl_3$): δ = 145.7, 139.6, 132.1, 129.1, 125.2, 123.1, 79.5, 56.8, 56.2, 50.0, 42.4, 40.4, 40.3, 39.8, 39.6, 37.3, 36.6, 36.3, 35.9, 32.0, 31.9, 30.1, 30.0, 28.4, 28.1, 24.4, 23.9, 23.0, 22.7, 21.1, 19.4, 18.8, 12.0. **HRMS-ASAP** (m/z): Calculated (found) for $C_{33}H_{51}O_2S$ $[M+H]^+$ 511.3604 (511.3602). **Anal.** for $C_{33}H_{50}O_2S$ calcd: C, 77.59; H, 9.87; S, 6.28. found: C, 77.50; H, 9.78; S, 6.40.

1,4-Bis(phenylsulfonyl)butane 35a

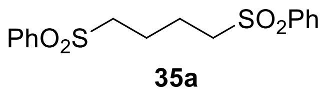

35a

A glass tube was charged with KOH (0.387 g, 6.9 mmol) and thiopheol (0.68 mL, 6.6 mmol) in ethanol (10 mL) and stirred for 1 h. 1,4-Dibromobutane (0.36 mL, 3.0 mmol) in ethanol (5 mL) was slowly added at 0 °C to the solution, and the mixture was stirred at room temperature for 12 h and filtered through a pad of Celite (Ø 3 mm x 8 mm) before the solvent was evaporated. The crude aryl sulfide was dissolved in DCM (12 mL) in an ice-water bath before *m*-CPBA (contains *ca*. 23 wt%, 15.0 mmol, 3.36 g) was added in portions. The mixture was allowed to warm to room temperature. After 12 h, saturated aqueous Na_2CO_3 was added, and the resulting solution was extracted with EtOAc (3 x 30 mL). The combined organic layer was dried over Na_2SO_4 and filtered through a pad of Celite (Ø 3 mm x 8 mm). The reaction mixture was purified by recrystallization (hexane/EtOAc) to yield the product **3a** as a white solid (720 mg, 2.13 mmol, 71% yield).

^{1}H NMR (300 MHz, $CDCl_3$): δ = 7.88 – 7.85 (m, 4H), 7.69 – 7.63 (m, 2H), 7.59 – 7.53 (m, 4H), 3.09 – 3.04 (m, 2H), 1.86 – 1.81 (m, 2H). **^{13}C{^{1}H} NMR** (75 MHz, $CDCl_3$): δ = 138.9, 134.0, 129.5, 128.1, 55.5, 21.7. **HRMS-ASAP** (m/z): Calculated (found) for $C_{16}H_{19}O_4S_2$ $[M+H]^+$ 339.0719 (339.0710).

The spectroscopic data for **35a** match those reported in the literature.[22]

<u>((4-Bromobutyl)sulfonyl)benzene 36a</u>

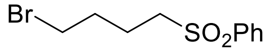

36a

According to **General procedure 1** with 1,4-dibromobutane (410 mg, 3.0 mmol, 1.0 equiv.), $PhSO_2Na$ (492 mg, 3.0 mmol, 1.0 equiv.) and NaI (45 mg, 0.3 mmol, 0.1 equiv.), the reaction mixture was purified by column chromatography on silica gel (hexane/EtOAc = 10/1) to yield the product **36a** as a white solid (565 mg, 2.04 mmol, 68% yield). **^{1}H NMR** (300 MHz, $CDCl_3$): δ = 7.93 – 7.91 (m, 2H), 7.71 – 7.65 (m, 1H), 7.61 – 7.56 (m, 2H), 3.37 (t, J = 6 Hz, 2H), 3.12 (t, J = 8 Hz, 2H), 2.02 – 1.84 (m, 4H). **^{13}C{^{1}H} NMR** (75 MHz, $CDCl_3$): δ = 139.1, 134.0, 129.5, 128.2, 55.4, 32.3, 31.1, 21.7. **HRMS-ASAP** (m/z): Calculated (found) for $C_{10}H_{14}BrO_2S$ $[M+H]^+$ 276.9892 (276.9885).

The spectroscopic data for **36a** match those reported in the literature.[23]

1.4 Details of the Borylation of Alkyl Sulfones

General procedure 5:

In an argon-filled glovebox, the alkyl sulfone (0.5 mmol, 1.0 equiv.), dissolved in DMA (1 mL), was added to a 10 mL thick-walled reaction tube equipped with a magnetic stirring bar. NaO^tBu (144 mg, 1.5 mmol, 3.0 equiv.) and B_2neop_2 (339 mg, 1.5 mmol, 3.0 equiv.) were added. The reaction mixture was stirred at 120 °C for 5 h, then diluted with Et_2O (2 mL) and filtered through a pad of Celite (Ø 3 mm x 8 mm). After careful removal of the solvent *in vacuo*, the product was purified by flash column chromatography (hexane/EtOAc). All alkyl boronate products were unambiguously identified by comparison of HRMS and ^1H, $^{13}C\{^1H\}$, $^{11}B\{^1H\}$ and/or $^{19}F\{^1H\}$ NMR spectra with literature data.

General procedure 6:

In an argon-filled glovebox, alkyl sulfone (0.5 mmol, 1.0 equiv.), dissolved in 1,4-dioxane (2 mL), was added to a 10 mL thick-walled reaction tube equipped with a magnetic stirring bar. KOMe (42 mg, 0.6 mmol, 1.2 equiv.) and B_2neop_2 (135.5 mg, 0.6 mmol, 1.2 equiv.) were added. The reaction mixture was stirred at 110 °C for 2 h, then diluted with Et_2O (2 mL) and filtered through a pad of Celite (Ø 3 mm x 8 mm). The product was purified by flash column chromatography (hexane/EtOAc) after careful removal of the solvent *in vacuo*. All alkyl boronate products were unambiguously identified by comparison of HRMS and ^1H, $^{13}C\{^1H\}$, $^{11}B\{^1H\}$ and/or $^{19}F\{^1H\}$ NMR spectra with literature data.

<u>2-(3-Phenylpropyl)-5,5-dimethyl-1,3,2-dioxaborinane 1b</u>

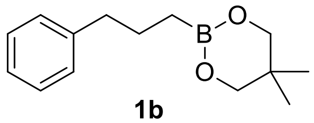

1b

According to **General procedure 5** with **1a-2** (130 mg, 0.5 mmol, 1.0 equiv.), the reaction mixture was purified by column chromatography on silica gel (hexane/EtOAc = 97/3) to yield the product **1b** as a white solid (104 mg, 450 μmol, 90% yield). **^{1}H NMR** (300 MHz, $CDCl_3$): δ = 7.30 – 7.14 (m, 5H), 3.58 (s, 4H), 2.62 (t, J = 8 Hz, 2H), 1.77 – 1.67 (m, 2H), 0.96 (s, 6H), 0.78 (t, J = 8 Hz, 2H). **$^{13}C\{^1H\}$ NMR** (75 MHz, $CDCl_3$): δ = 143.1, 128.7, 128.2, 125.6, 72.1, 38.8, 31.7, 26.3, 22.0. **$^{11}B\{^1H\}$ NMR** (96 MHz, $CDCl_3$): δ = 30.3. **HRMS-ASAP** (m/z): Calculated (found) for $C_{14}H_{22}BO_2$ $[M+H]^+$ 233.1707 (233.1701).

The spectroscopic data for **1b** match those reported in the literature.[25]

2-Benzyl-5,5-dimethyl-1,3,2-dioxaborinane 2b

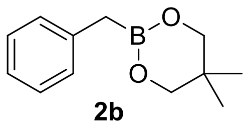

2b

According to **General procedure 6** with **2a** (116 mg, 0.5 mmol, 1.0 equiv.), the reaction mixture was purified by column chromatography on silica gel (hexane/EtOAc = 98/2) to yield the product **2b** as a colorless oil (73 mg, 360 µmol, 72% yield). **^{1}H NMR** (300 MHz, $CDCl_3$): δ = 7.24 – 7.08 (m, 5H), 3.59 (s, 4H), 2.23 (s, 2H), 0.93 (s, 6H). **^{13}C{^{1}H} NMR** (75 MHz, $CDCl_3$): δ = 140.0, 129.0, 128.3, 124.7, 72.4, 31.8, 21.9. **^{11}B{^{1}H} NMR** (96 MHz, $CDCl_3$): δ = 29.3. **HRMS-ASAP** (m/z): Calculated (found) for $C_{12}H_{18}BO_2$ $[M+H]^+$ 205.1394 (205.1387).

The spectroscopic data for **2b** match those reported in the literature.[26]

2-(4-Methylbenzyl)-5,5-dimethyl-1,3,2-dioxaborinane 3b

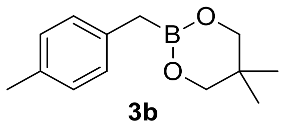

3b

According to **General procedure 6** with **3a** (123 mg, 0.5 mmol, 1.0 equiv.), the reaction mixture was purified by column chromatography on silica gel (hexane/EtOAc = 98/2) to yield the product **3b** as a colorless oil (60 mg, 275 µmol, 55% yield). **^{1}H NMR** (300 MHz, $CDCl_3$): δ = 7.06 (m, 4H), 3.59 (s, 4H), 2.30 (s, 3H), 2.19 (s, 2H), 0.94 (s, 6H). **^{13}C{^{1}H} NMR** (75 MHz, $CDCl_3$): δ = 136.8, 134.0, 129.0, 128.9, 72.4, 31.8, 21.9, 21.1. **^{11}B{^{1}H} NMR** (96 MHz, $CDCl_3$): δ = 29.4. **HRMS-ASAP** (m/z): Calculated (found) for $C_{13}H_{20}BO_2$ $[M+H]^+$ 219.1551 (219.1545).

The spectroscopic data for **3b** match those reported in the literature.[26]

2-(4-Methoxybenzyl)-5,5-dimethyl-1,3,2-dioxaborinane 4b

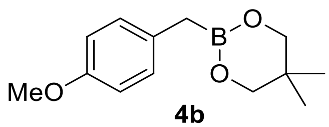

4b

According to **General procedure 6** with **4a** (131 mg, 0.5 mmol, 1.0 equiv.), the reaction mixture was purified by column chromatography on silica gel (hexane/EtOAc = 98/2) to yield the product **4b** as a colorless oil (80 mg, 340 µmol, 68% yield). **^{1}H NMR** (300 MHz, $CDCl_3$): δ = 7.09 (d, *J* = 9 Hz, 2H), 6.80 (d, *J* = 9 Hz, 2H), 3.77 (s, 3H), 3.59 (s, 4H), 2.16 (s, 2H), 0.94 (s, 6H). **^{13}C{^{1}H} NMR** (75 MHz, $CDCl_3$): δ = 157.1, 131.9, 129.8, 113.8, 72.3, 55.3, 31.8, 21.9. **^{11}B{^{1}H} NMR** (96 MHz, $CDCl_3$): δ = 29.4. **HRMS-ASAP** (m/z): Calculated (found) for $C_{13}H_{20}BO_3$ $[M+H]^+$ 235.1500 (235.1492).

The spectroscopic data for **4b** match those reported in the literature.[27]

2-(4-(Trifluoromethoxy)benzyl)-5,5-dimethyl-1,3,2-dioxaborinane 5b

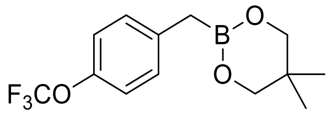

5b

According to **General procedure 6** with **5a** (158 mg, 0.5 mmol, 1.0 equiv.), the reaction mixture was purified by column chromatography on silica gel (hexane/EtOAc = 98/2) to yield the product **5b** as a colorless oil (92 mg, 320 μmol, 64% yield). **^{1}H NMR** (500 MHz, $CDCl_3$): δ = 7.16 (d, J = 9 Hz, 2H), 7.07 (d, J = 9 Hz, 2H), 360 (s, 4H), 2.22 (s, 2H), 0.93 (s, 6H). **^{13}C{^{1}H} NMR** (125 MHz, $CDCl_3$): δ = 146.7 (q, J = 1.25 Hz), 138.9, 130.1, 120.9, 120.7 (q, J = 255 Hz), 72.4, 31.8, 21.9. **^{11}B{^{1}H} NMR** (160 MHz, $CDCl_3$): δ = 29.3. **^{19}F{^{1}H} NMR** (470 MHz, $CDCl_3$): δ = -57.9 (s). **HRMS-ASAP** (m/z): Calculated (found) for $C_{13}H_{17}BF_3O_3$ $[M+H]^+$ 289.1217 (289.1209). **Anal.** for $C_{13}H_{16}BF_3O_3$ calcd: C, 54.20; H, 5.60. found: C, 54.13; H, 5.58.

2-(4-Fluorobenzyl)-5,5-dimethyl-1,3,2-dioxaborinane 6b

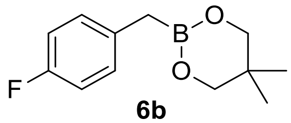

6b

According to **General procedure 6** with **6a** (125 mg, 0.5 mmol, 1.0 equiv.), the reaction mixture was purified by column chromatography on silica gel (hexane/EtOAc = 98/2) to yield the product **6b** as a colorless oil (67 mg, 305 μmol, 61% yield). **^{1}H NMR** (500 MHz, $CDCl_3$): δ = 7.12 – 7.08 (m, 2H), 6.94 – 6.89 (m, 2H), 3.59 (s, 4H), 2.19 (s, 2H), 0.93 (s, 6H). **^{13}C{^{1}H} NMR** (125 MHz, $CDCl_3$): δ = 160.8 (d, J = 240 Hz), 135.5 (d, J = 3.75 Hz), 130.2 (d, J = 8.75 Hz), 115.0 (d, J = 20 Hz), 72.4, 31.8, 21.9. **^{11}B{^{1}H} NMR** (160 MHz, $CDCl_3$): δ = 29.3. **^{19}F{^{1}H} NMR** (470 MHz, $CDCl_3$): δ = -119.8 (s). **HRMS-ASAP** (m/z): Calculated (found) for $C_{12}H_{17}BFO_2$ $[M+H]^+$ 223.1300 (223.1294).

The spectroscopic data for **6b** match those reported in the literature.[26]

2-(4-Chlorobenzyl)-5,5-dimethyl-1,3,2-dioxaborinane 7b

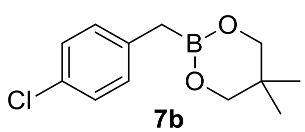

7b

According to **General procedure 6** with **7a** (133 mg, 0.5 mmol, 1.0 equiv.), the reaction mixture was purified by column chromatography on silica gel (hexane/EtOAc = 98/2) to yield the product **7b** as a

colorless oil (67 mg, 280 µmol, 56% yield). **^{1}H NMR** (300 MHz, $CDCl_3$): δ = 7.19 (d, J = 9 Hz, 2H), 7.09 (d, J = 9 Hz, 2H), 3.59 (s, 4H), 2.19 (s, 2H), 0.93 (s, 6H). **^{13}C{^{1}H} NMR** (75 MHz, $CDCl_3$): δ = 138.5, 130.41, 130.35, 128.3, 72.4, 31.8, 21.9. **^{11}B{^{1}H} NMR** (96 MHz, $CDCl_3$): δ = 29.2. **HRMS-ASAP** (m/z): Calculated (found) for $C_{12}H_{17}BClO_2$ $[M+H]^+$ 239.1005 (239.0999).

The spectroscopic data for **7b** match those reported in the literature.[26]

2-(4-Bromobenzyl)-5,5-dimethyl-1,3,2-dioxaborinane 8b

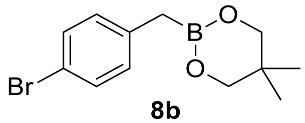

8b

According to **General procedure 6** with **8a** (156 mg, 0.5 mmol, 1.0 equiv.), the reaction mixture was purified by column chromatography on silica gel (hexane/EtOAc = 98/2) to yield the product **8b** as a colorless oil (89 mg, 315 µmol, 63% yield). **^{1}H NMR** (300 MHz, $CDCl_3$): δ = 7.34 (d, J = 9 Hz, 2H), 7.04 (d, J = 9 Hz, 2H), 3.59 (s, 4H), 2.17 (s, 2H), 0.93 (s, 6H). **^{13}C{^{1}H} NMR** (75 MHz, $CDCl_3$): δ = 139.1, 131.3, 130.8, 118.4, 72.4, 31.8, 21.9. **^{11}B{^{1}H} NMR** (96 MHz, $CDCl_3$): δ = 29.1. **HRMS-ASAP** (m/z): Calculated (found) for $C_{12}H_{17}BBrO_2$ $[M+H]^+$ 283.0499 (283.0495).

The spectroscopic data for **8b** match those reported in the literature.[26]

2-Phenethyl-5,5-dimethyl-1,3,2-dioxaborinane 9b

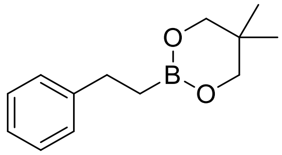

9b

According to **General procedure 5** with **9a** (123 mg, 0.5 mmol, 1.0 equiv.), the reaction mixture was purified by column chromatography on silica gel (hexane/EtOAc = 98/2) to yield the product **9b** as a colorless oil (100 mg, 460 µmol, 92% yield). **^{1}H NMR** (400 MHz, $CDCl_3$): δ = 7.29 – 7.13 (m, 5H), 3.60 (s, 4H), 2.71 (t, J = 8 Hz, 2H), 1.09 (t, J = 8 Hz, 2H), 0.94 (s, 6H). **^{13}C{^{1}H} NMR** (100 MHz, $CDCl_3$): δ = 145.1, 128.3, 128.1, 125.5, 72.1, 31.8, 30.2, 21.9. **^{11}B{^{1}H} NMR** (128 MHz, $CDCl_3$): δ = 29.3. **HRMS-ASAP** (m/z): Calculated (found) for $C_{13}H_{20}BO_2$ $[M+H]^+$ 219.1551 (219.1547).

The spectroscopic data for **9b** match those reported in the literature.[28, 33]

5,5-Dimethyl-2-octyl-1,3,2-dioxaborinane 10b

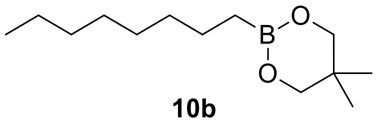

10b

According to **General procedure 5** with **10a** (127 mg, 0.5 mmol, 1.0 equiv.), the reaction mixture was purified by column chromatography on silica gel (hexane/EtOAc = 98/2) to yield the product **10b** as a colorless oil (106 mg, 470 µmol, 94% yield). **^{1}H NMR** (300 MHz, $CDCl_3$): δ = 3.58 (s, 4H), 1.38 – 1.26 (m, 12H), 0.95 (s, 6H), 0.89 – 0.85 (m, 3H), 0.69 (t, J = 7 Hz, 2H). **^{13}C{^{1}H} NMR** (75 MHz, $CDCl_3$): δ = 72.1, 32.7, 32.1, 31.8, 29.6, 29.4, 24.3, 22.8, 22.0, 14.3. **^{11}B{^{1}H} NMR** (96 MHz, $CDCl_3$): δ = 30.3. **HRMS-ASAP** (m/z): Calculated (found) for $C_{13}H_{28}BO_2$ $[M+H]^+$ 227.2177 (227.2169).

The spectroscopic data for **10b** match those reported in the literature.[27]

2-(3-(4-Methoxyphenyl)propyl)-5,5-dimethyl-1,3,2-dioxaborinane 11b

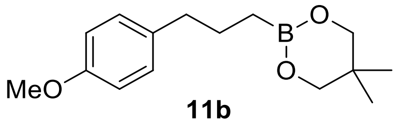

11b

According to **General procedure 5** with **11a** (145 mg, 0.5 mmol, 1.0 equiv.), the reaction mixture was purified by column chromatography on silica gel (hexane/EtOAc = 98/2) to yield the product **11b** as a colorless oil (114 mg, 435 µmol, 87% yield). **^{1}H NMR** (300 MHz, $CDCl_3$): δ = 7.12 – 7.07 (m, 2H), 6.84 – 6.79 (m, 2H), 3.78 (s, 3H), 3.57 (s, 4H), 2.54 (t, J = 8 Hz, 2H), 1.72 – 1.61 (m, 2H), 0.95 (s, 6H), 0.75 (t, J = 8 Hz, 2H). **^{13}C{^{1}H} NMR** (75 MHz, $CDCl_3$): δ = 157.7, 135.3, 129.6, 113.7, 72.1, 55.4, 37.9, 31.7, 26.5, 22.0. **^{11}B{^{1}H} NMR** (96 MHz, $CDCl_3$): δ = 30.2. **HRMS-ASAP** (m/z): Calculated (found) for $C_{15}H_{24}BO_3$ $[M+H]^+$ 263.1813 (263.1808).

The spectroscopic data for **11b** match those reported in the literature.[28]

2-(4-Fluorophenethyl)-5,5-dimethyl-1,3,2-dioxaborinane 12b

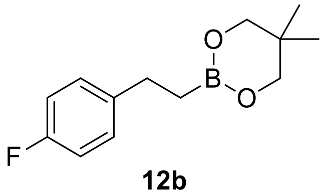

12b

According to **General procedure 5** with **12a** (132 mg, 0.5 mmol, 1.0 equiv.), the reaction mixture was purified by column chromatography on silica gel (hexane/EtOAc = 98/2) to yield the product **12b** as a white solid (100 mg, 425 µmol, 85%). **^{1}H NMR** (400 MHz, $CDCl_3$): δ = 7.18 – 7.13 (m, 2H), 6.96 – 6.90 (m, 2H), 3.59 (s, 4H), 2.67 (t, J = 8 Hz, 2H), 1.05 (t, J = 8 Hz, 2H), 0.92 (s, 6H). **^{13}C{^{1}H} NMR** (100 MHz,

$CDCl_3$): δ = 161.1 (d, J = 240 Hz), 140.6 (d, J = 3 Hz), 129.4 (d, J = 8 Hz), 114.9 (d, J = 21 Hz), 72.1, 31.8, 29.4, 21.9. **^{19}F{^{1}H} NMR** (376 MHz, $CDCl_3$): δ = -118.6 (s). **^{11}B{^{1}H} NMR** (128 MHz, $CDCl_3$): δ = 30.0. **HRMS-ASAP** (m/z): Calculated (found) for $C_{13}H_{19}BFO_2$ $[M+H]^+$ 237.1457 (237.1455).

The spectroscopic data for **12b** match those reported in the literature.[29]

<u>2-(2-(1,3-Dioxan-2-yl)ethyl)-5,5-dimethyl-1,3,2-dioxaborinane 14b</u>

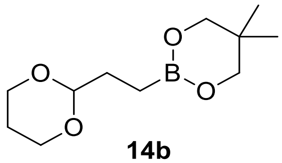

14b

According to **General procedure 5** with **14a** (128 mg, 0.5 mmol, 1.0 equiv.), the reaction mixture was purified by column chromatography on silica gel (hexane/EtOAc = 98/2) to yield the product **14b** as a colorless oil (95 mg, 420 µmol, 84% yield). **^{1}H NMR** (300 MHz, $CDCl_3$): δ = 4.45 (t, J = 5 Hz, 1H), 4.08 (dd, J = 5, 11 Hz, 2H), 3.74 (dt, J = 2, 12 Hz, 2H), 3.57 (s, 4H), 2.15 – 1.99 (m, 1H), 1.70 – 1.64 (m, 2H), 1.34 – 1.27 (m, 1H), 0.94 (s, 6H), 0.75 (t, J = 8 Hz, 2H). **^{13}C{^{1}H} NMR** (75 MHz, $CDCl_3$): δ = 103.7, 72.1, 67.0, 31.8, 29.7, 26.0, 22.0. **^{11}B{^{1}H} NMR** (96 MHz, $CDCl_3$): δ = 30.2. **HRMS-ASAP** (m/z): Calculated (found) for $C_{11}H_{22}BO_4$ $[M+H]^+$ 229.1606 (229.1602).

The spectroscopic data for **14b** match those reported in the literature.[28]

<u>Methyl 4-(5,5-dimethyl-1,3,2-dioxaborinan-2-yl)butanoate 15b</u>

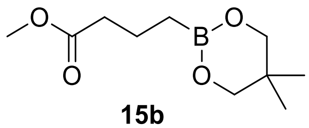

15b

According to **General procedure 5** with **15a** (121 mg, 0.5 mmol, 1.0 equiv.), the reaction mixture was purified by column chromatography on silica gel (hexane/EtOAc = 96/4) to yield the product **15b** as a colorless oil (56 mg, 265 µmol, 53% yield). **^{1}H NMR** (500 MHz, $CDCl_3$): δ = 3.64 (s, 3H), 3.57 (s, 4H), 2.30 (t, J = 8 Hz, 2H), 1.73 – 1.67 (m, 2H), 0.94 (s, 6H), 0.74 (t, J = 8 Hz, 2H). **^{13}C{^{1}H} NMR** (125 MHz, $CDCl_3$): δ = 174.5, 72.1, 51.5, 36.6, 31.8, 22.0, 19.9. **^{11}B{^{1}H} NMR** (160 MHz, $CDCl_3$): δ = 30.1. **HRMS-ASAP** (m/z): Calculated (found) for $C_{10}H_{20}BO_4$ $[M+H]^+$ 215.1449 (215.1441).

The spectroscopic data for **15b** match those reported in the literature.[30]

5,5-Dimethyl-2-(4-methylpent-3-en-1-yl)-1,3,2-dioxaborinane 16b

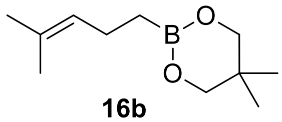

16b

According to **General procedure 5** with **16a** (112 mg, 0.5 mmol, 1.0 equiv.), the reaction mixture was purified by column chromatography on silica gel (hexane/EtOAc = 98/2) to yield the product **16b** as a colorless oil (50 mg, 255 μmol, 51% yield). **^{1}H NMR** (500 MHz, $CDCl_3$): *δ* = 5.15 – 5.11 (m, 1H), 3.59 (s, 4H), 2.07 – 2.02 (m, 2H), 1.66 (s, 3H), 1.60 (s, 3H), 0.95 (s, 6H), 0.75 (t, *J* = 8 Hz, 2H). **^{13}C{^{1}H} NMR** (125 MHz, $CDCl_3$): *δ* = 130.3, 127.4, 72.1, 31.8, 25.9, 22.7, 22.0, 17.7. **^{11}B{^{1}H} NMR** (160 MHz, $CDCl_3$): *δ* = 30.3. **HRMS-ASAP** (m/z): Calculated (found) for $C_{11}H_{22}BO_2$ $[M+H]^+$ 197.1707 (197.1701). **Anal.** for $C_{11}H_{21}BO_2$ calcd: C, 67.37; H, 10.79. found: C, 67.21; H, 10.81.

2-(5-Fluoropentyl)-5,5-dimethyl-1,3,2-dioxaborinane 17b

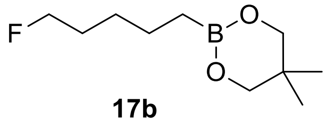

17b

According to **General procedure 5** with **17a** (115 mg, 0.5 mmol, 1.0 equiv.), the reaction mixture was purified by column chromatography on silica gel (hexane/EtOAc = 98/2) to yield the product **17b** as a colorless oil (75 mg, 370 μmol, 74% yield). **^{1}H NMR** (400 MHz, $CDCl_3$): *δ* = 4.48 (t, *J* = 6 Hz, 1H), 4.37 (t, *J* = 6 Hz, 1H), 3.58 (s, 4H), 1.75 – 1.62 (m, 2H), 1.40 – 1.38 (m, 4H), 0.95 (s, 6H), 0.72 (t, *J* = 7 Hz, 2H). **^{13}C{^{1}H} NMR** (100 MHz, $CDCl_3$): *δ* = 84.4 (d, *J* = 163 Hz), 72.1, 31.8, 30.4 (d, *J* = 19 Hz), 28.0 (d, *J* = 6 Hz), 23.9, 22.0. **^{19}F{^{1}H} NMR** (376 MHz, $CDCl_3$): *δ* = -217.8 (s). **^{11}B{^{1}H} NMR** (128 MHz, $CDCl_3$): *δ* = 30.7. **HRMS-ASAP** (m/z): Calculated (found) for $C_{10}H_{21}BFO_2$ $[M+H]^+$ 203.1613 (203.1610). **Anal.** for $C_{10}H_{21}BFO_2$ calcd: C, 59.44; H, 9.98. found: C, 59.53; H, 10.03.

2-(6-Chlorohexyl)-5,5-dimethyl-1,3,2-dioxaborinane 18b

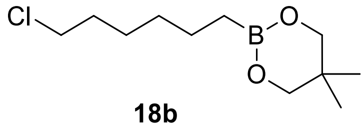

18b

According to **General procedure 5** with **18a** (130 mg, 0.5 mmol, 1.0 equiv.), the reaction mixture was purified by column chromatography on silica gel (hexane/EtOAc = 98/2) to yield the product **18b** as a colorless oil (79 mg, 340 μmol, 68% yield). **^{1}H NMR** (300 MHz, $CDCl_3$): *δ* = 3.59 (s, 4H), 3.52 (t, *J* = 7 Hz, 2H), 1.81 – 1.71 (m, 2H), 1.44 – 1.24 (m, 6H), 0.95 (s, 6H), 0.71 (t, *J* = 7 Hz, 2H). **^{13}C{^{1}H} NMR** (75 MHz, $CDCl_3$): *δ* = 72.1, 45.4, 32.7, 31.82, 31.76, 26.9, 24.1, 22.0. **^{11}B{^{1}H} NMR** (96 MHz, $CDCl_3$): *δ*

= 30.4. **HRMS-ASAP** (m/z): Calculated (found) for $C_{11}H_{23}BClO_2$ $[M+H]^+$ 233.1474 (233.1477). **Anal.** for $C_{11}H_{22}BClO_2$ calcd: C, 56.81; H, 9.54. found: C, 56.88; H, 9.51.

6-(5,5-Dimethyl-1,3,2-dioxaborinan-2-yl)hexanenitrile 19b

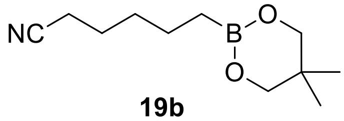

19b

According to **General procedure 5** with **19a** (119 mg, 0.5 mmol, 1.0 equiv.), the reaction mixture was purified by column chromatography on silica gel (hexane/EtOAc = 95/5) to yield the product **19b** as a colorless oil (79 mg, 380 µmol, 76% yield). **^{1}H NMR** (500 MHz, $CDCl_3$): δ = 3.58 (s, 4H), 2.32 (t, J = 8 Hz, 2H), 1.68 – 1.62 (m, 2H), 1.47 – 1.36 (m, 4H), 0.95 (s, 6H), 0.72 (t, J = 8 Hz, 2H). **^{13}C{^{1}H} NMR** (125 MHz, $CDCl_3$): δ = 120.1, 72.1, 31.8, 31.4, 25.4, 23.3, 22.0, 17.2. **^{11}B{^{1}H} NMR** (160 MHz, $CDCl_3$): δ = 30.3. **HRMS-ASAP** (m/z): Calculated (found) for $C_{11}H_{21}BNO_2$ $[M+H]^+$ 210.1660 (210.1658).

The spectroscopic data for **19b** match those reported in the literature.[30]

9-(4-(5,5-Dimethyl-1,3,2-dioxaborinan-2-yl)butyl)-9*H*-carbazole 20b

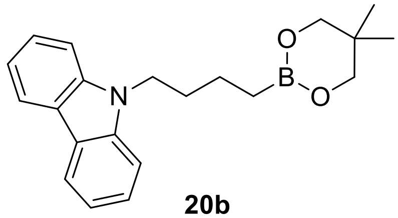

20b

According to **General procedure 5** with **20a** (182 mg, 0.5 mmol, 1.0 equiv.), the reaction mixture was purified by column chromatography on silica gel (hexane/EtOAc = 95/5) to yield the product **20b** as a colorless oil (115 mg, 345 µmol, 69% yield). **^{1}H NMR** (500 MHz, $CDCl_3$): δ = 8.12 – 8.10 (m, 2H), 7.48 – 7.42 (m, 4H), 7.24 – 7.21 (m, 2H), 4.30 (t, J = 7 Hz, 2H), 3.57 (s, 4H), 1.92 – 1.85 (m, 2H), 1.56 – 1.49 (m, 2H), 0.94 (s, 6H), 0.82 – 0.79 (m, 2H). **^{13}C{^{1}H} NMR** (125 MHz, $CDCl_3$): δ = 140.6, 125.6, 122.9, 120.4, 118.7, 108.9, 72.1, 43.1, 31.7, 31.6, 22.0, 21.9. **^{11}B{^{1}H} NMR** (160 MHz, $CDCl_3$): δ = 30.3. **HRMS-ASAP** (m/z): Calculated (found) for $C_{21}H_{27}BNO_2$ $[M+H]^+$ 336.2129 (336.2119).

The spectroscopic data for **20b** match those reported in the literature.[25]

1-(4-(5,5-Dimethyl-1,3,2-dioxaborinan-2-yl)butyl)-1*H*-indole 21b

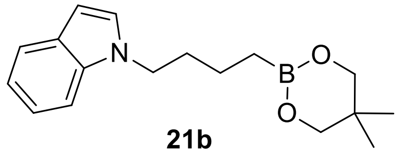

21b

According to **General procedure 5** with **21a** (157 mg, 0.5 mmol, 1.0 equiv.), the reaction mixture was purified by column chromatography on silica gel (hexane/EtOAc = 95/5) to yield the product **21b** as a colorless oil (115 mg, 405 µmol, 81% yield). **^{1}H NMR** (300 MHz, $CDCl_3$): δ = 7.63 (d, J = 7 Hz, 1H), 7.36 (d, J = 7 Hz, 1H), 7.23 – 7.17 (m, 1H), 7.12 – 7.06 (m, 2H), 6.48 (d, J = 3 Hz, 1H), 4.11 (t, J = 7 Hz, 2H), 3.58 (s, 4H), 1.90 – 1.80 (m, 2H), 1.49 – 1.39 (m, 2H), 0.95 (s, 6H), 0.77 (t, J = 8 Hz, 2H). **^{13}C{^{1}H} NMR** (75 MHz, $CDCl_3$): δ = 136.1, 128.7, 127.9, 121.3, 121.0, 119.2, 109.6, 100.8, 72.1, 46.5, 32.9, 31.7, 22.0, 21.7. **^{11}B{^{1}H} NMR** (96 MHz, $CDCl_3$): δ = 30.1. **HRMS-ASAP** (m/z): Calculated (found) for $C_{17}H_{25}BNO_2$ $[M+H]^+$ 286.1973 (286.1969).

The spectroscopic data for **21b** match those reported in the literature.[25]

2-(Pentan-3-yl)-5,5-dimethyl-1,3,2-dioxaborinane 22b

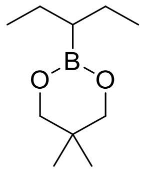

22b

According to **General procedure 5** with **22a** (106 mg, 0.5 mmol, 1.0 equiv.), the reaction mixture was purified by column chromatography on silica gel (hexane/EtOAc = 99/1) to yield the product **22b** as a colorless oil (68 mg, 370 µmol, 74% yield). **^{1}H NMR** (400 MHz, $CDCl_3$): δ = 3.59 (s, 4H), 1.44 – 1.31 (m, 4H), 0.96 (s, 6H), 0.88 (t, J = 7 Hz, 6H), 0.73 – 0.65 (m, 1H). **^{13}C{^{1}H} NMR** (100 MHz, $CDCl_3$): δ = 72.0, 31.7, 24.1, 22.1, 14.0. **^{11}B{^{1}H} NMR** (128 MHz, $CDCl_3$): δ = 29.9. **HRMS-ASAP** (m/z): Calculated (found) for $C_{10}H_{22}BO_2$ $[M+H]^+$ 185.1707 (185.1702).

The spectroscopic data for **22b** match those reported in the literature.[31]

2-Cyclopropyl-5,5-dimethyl-1,3,2-dioxaborinane 23b

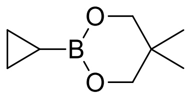

23b

According to **General procedure 5** with **23a** (91 mg, 0.5 mmol, 1.0 equiv.), the reaction mixture was purified by column chromatography on silica gel (hexane/EtOAc = 99/1) to yield the product **23b** as a

colorless oil (39 mg, 255 μmol, 51% yield). **^{1}H NMR** (400 MHz, $CDCl_3$): δ = 3.55 (s, 4H), 0.94 (s, 6H), 0.54 – 0.50 (m, 2H), 0.44 – 0.40 (m, 2H), -0.27 – -0.35 (m, 1H). **^{13}C{^{1}H} NMR** (100 MHz, $CDCl_3$): δ = 72.1, 31.9, 21.9, 3.54. **^{11}B{^{1}H} NMR** (128 MHz, $CDCl_3$): δ = 28.8. **HRMS-ASAP** (m/z): Calculated (found) for $C_8H_{16}BO_2$ $[M+H]^+$ 155.1238 (155.1232).

2-Cyclobutyl-5,5-dimethyl-1,3,2-dioxaborinane 24b

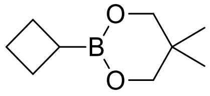

24b

According to **General procedure 5** with **24a** (98 mg, 0.5 mmol, 1.0 equiv.), the reaction mixture was purified by column chromatography on silica gel (hexane/EtOAc = 99/1) to yield the product **24b** as a colorless oil (51 mg, 305 μmol, 61% yield). **^{1}H NMR** (400 MHz, $CDCl_3$): δ = 3.60 (s, 4H), 2.09 – 1.98 (m, 5H), 1.96 – 1.81 (m, 2H), 0.96 (s, 6H). **^{13}C{^{1}H} NMR** (100 MHz, $CDCl_3$): δ = 72.2, 31.8, 24.1, 22.6, 21.9. **^{11}B{^{1}H} NMR** (128 MHz, $CDCl_3$): δ = 29.1. **HRMS-ASAP** (m/z): Calculated (found) for $C_9H_{18}BO_2$ $[M+H]^+$ 169.1394 (169.1387).

2-Cyclopentyl-5,5-dimethyl-1,3,2-dioxaborinane 25b

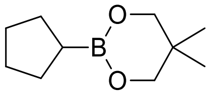

25b

According to **General procedure 5** with **25a** (105 mg, 0.5 mmol, 1.0 equiv.), the reaction mixture was purified by column chromatography on silica gel (hexane/EtOAc = 99/1) to yield the product **25b** as a colorless oil (58 mg, 320 μmol, 64% yield). **^{1}H NMR** (400 MHz, $CDCl_3$): δ = 3.59 (s, 4H), 1.76 – 1.68 (m, 2H), 1.62 – 1.40 (m, 6H), 1.12 – 1.05 (m, 1H), 0.95 (s, 6H). **^{13}C{^{1}H} NMR** (100 MHz, $CDCl_3$): δ = 72.2, 31.6, 28.8, 27.0, 21.9. **^{11}B{^{1}H} NMR** (128 MHz, $CDCl_3$): δ = 30.9. **HRMS-ASAP** (m/z): Calculated (found) for $C_{10}H_{20}BO_2$ $[M+H]^+$ 183.1551 (183.1545).

The spectroscopic data for **25b** match those reported in the literature.[28,31]

2-Cyclohexyl-5,5-dimethyl-1,3,2-dioxaborinane 26b

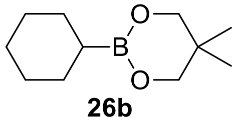

26b

According to **General procedure 5** with **26a** (112 mg, 0.5 mmol, 1.0 equiv.), the reaction mixture was purified by column chromatography on silica gel (hexane/EtOAc = 99/1) to yield the product **26b** as a

colorless oil (52 mg, 265 µmol, 53% yield). **^{1}H NMR** (300 MHz, $CDCl_3$): δ = 3.58 (s, 4H), 1.68 – 1.59 (m, 5H), 1.28 – 1.23 (m, 5H), 0.94 (s, 6H), 0.89 – 0.80 (m, 1H). **^{13}C{^{1}H} NMR** (75 MHz, $CDCl_3$): δ = 72.1, 31.8, 28.3, 27.6, 27.1, 21.9. **^{11}B{^{1}H} NMR** (96 MHz, $CDCl_3$): δ = 30.1. **HRMS-ASAP** (m/z): Calculated (found) for $C_{11}H_{22}BO_2$ $[M+H]^+$ 197.1707 (197.1701).

The spectroscopic data for **26b** match those reported in the literature.[25,28]

2-(Cyclohex-2-en-1-yl)-5,5-dimethyl-1,3,2-dioxaborinane 27b

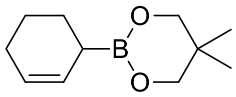

27b

According to **General procedure 6** with **27a** (111 mg, 0.5 mmol, 1.0 equiv.), the reaction mixture was purified by column chromatography on silica gel (hexane/EtOAc = 99/1) to yield the product **27b** as a colorless oil (65 mg, 335 µmol, 67% yield). **^{1}H NMR** (300 MHz, $CDCl_3$): δ = 5.77 – 5.72 (m, 1H), 5.68 – 5.62 (m, 1H), 3.60 (s, 4H), 2.01 – 1.96 (m, 2H), 1.73 – 1.62 (m, 4H), 0.95 (s, 6H), 0.89 – 0.81 (m, 1H). **^{13}C{^{1}H} NMR** (75 MHz, $CDCl_3$): δ = 128.9, 125.6, 72.3, 31.8, 25.3, 24.4, 22.8, 21.9. **^{11}B{^{1}H} NMR** (96 MHz, $CDCl_3$): δ = 29.5. **HRMS-ASAP** (m/z): Calculated (found) for $C_{11}H_{20}BO_2$ $[M+H]^+$ 195.1551 (195.1550).

The spectroscopic data for **27b** match those reported in the literature.[31]

***tert*-Butyl 4-(5,5-dimethyl-1,3,2-dioxaborinan-2-yl)piperidine-1-carboxylate 28b**

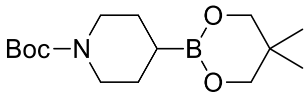

28b

According to **General procedure 5** with **28a** (163 mg, 0.5 mmol, 1.0 equiv.), the reaction mixture was purified by column chromatography on silica gel (hexane/EtOAc = 95/5) to yield the product **28b** as a colorless oil (105 mg, 355 µmol, 71% yield). **^{1}H NMR** (300 MHz, $CDCl_3$): δ = 3.86 (dt, J = 4, 13 Hz, 2H), 3.57 (s, 4H), 2.85 – 2.76 (m, 2H), 1.65 – 1.57 (m, 2H), 1.44 (s, 9H), 1.42 – 1.33 (m, 2H), 1.01 – 0.96 (m, 1H), 0.93 (s, 6H). **^{13}C{^{1}H} NMR** (75 MHz, $CDCl_3$): δ = 155.1, 79.1, 72.1, 45.2, 31.8, 28.6, 27.3, 21.9. **^{11}B{^{1}H} NMR** (96 MHz, $CDCl_3$): δ = 30.0. **HRMS-ASAP** (m/z): Calculated (found) for $C_{15}H_{29}BNO_4$ $[M+H]^+$ 298.2184 (298.2179).

The spectroscopic data for **28b** match those reported in the literature.[32]

2-(1-Phenylpropan-2-yl)-5,5-dimethyl-1,3,2-dioxaborinane 29b

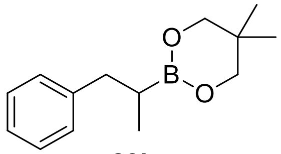

29b

According to **General procedure 5** with **29a** (130 mg, 0.5 mmol, 1.0 equiv.), the reaction mixture was purified by column chromatography on silica gel (hexane/EtOAc = 95/5) to yield the product **29b** as a colorless oil (75 mg, 325 µmol, 65% yield). **^{1}H NMR** (400 MHz, $CDCl_3$): δ = 7.24 (d, J = 7 Hz, 2H), 7.18 (d, J = 7 Hz, 2H), 7.15 (t, J = 7 Hz, 1H), 3.57 (s, 4H), 2.82 (dd, J = 7, 14 Hz, 1H), 2.46 (dd, J = 9, 14 Hz, 1H), 1.27 – 1.22 (m, 1H), 0.92 (d, J = 7 Hz, 3H), 0.90 (s, 6H). **^{13}C{^{1}H} NMR** (100 MHz, $CDCl_3$): δ = 143.1, 129.0, 128.1, 125.5, 72.1, 39,2, 31.7, 21.9, 15.5. **^{11}B{^{1}H} NMR** (128 MHz, $CDCl_3$): δ = 29.9. **HRMS-ASAP** (m/z): Calculated (found) for $C_{14}H_{22}BO_2$ $[M+H]^+$ 233.1707 (233.1704).

The spectroscopic data for **29b** match those reported in the literature.[33]

2-(1-(4-Fluorophenyl)propan-2-yl)-5,5-dimethyl-1,3,2-dioxaborinane 30b

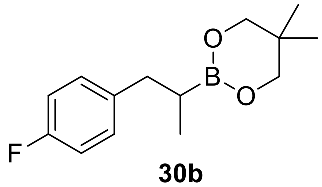

30b

According to **General procedure 5** with **30a** (139 mg, 0.5 mmol, 1.0 equiv.), the reaction mixture was purified by column chromatography on silica gel (hexane/EtOAc = 95/5) to yield the product **30b** as a colorless oil (86 mg, 345 µmol, 69% yield). **^{1}H NMR** (400 MHz, $CDCl_3$): δ = 7.15 – 7.10 (m, 2H), 6.96 – 6.90 (m, 2H), 3.56 (s, 4H), 2.79 – 2.73 (m, 1H), 2.46 – 2.41 (m, 1H), 1.23 – 1.67 (m, 1H), 0.91 (d, J = 7 Hz, 3H), 0.89 (s, 6H). **^{13}C{^{1}H} NMR** (100 MHz, $CDCl_3$): δ = 161.2 (d, J = 241 Hz), 138.6 (d, J = 3 Hz), 130.2 (d, J = 8 Hz), 114.8 (d, J = 21 Hz), 72.1, 38.4, 31.7, 21.9, 15.5. **^{19}F{^{1}H} NMR** (376 MHz, $CDCl_3$): δ = -118.6 (s). **^{11}B{^{1}H} NMR** (128 MHz, $CDCl_3$): δ = 30.1. **HRMS-ASAP** (m/z): Calculated (found) for $C_{14}H_{21}BFO_2$ $[M+H]^+$ 251.1613 (251.1610). **Anal.** for $C_{14}H_{20}BFO_2$ calcd: C, 67.23; H, 8.06. found: C, 67.17; H, 8.20.

2-(4-(4-Methoxyphenyl)butan-2-yl)-5,5-dimethyl-1,3,2-dioxaborinane 31b

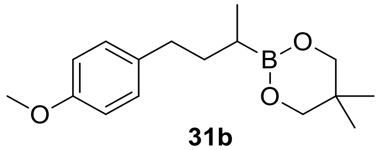

31b

According to **General procedure 5** with **31a** (152 mg, 0.5 mmol, 1.0 equiv.), the reaction mixture was purified by column chromatography on silica gel (hexane/EtOAc = 95/5) to yield the product **31b** as a colorless oil (98 mg, 355 μmol, 71% yield). **^{1}H NMR** (400 MHz, $CDCl_3$): δ = 7.12 (d, J = 8 Hz, 2H), 6.82 (d, J = 8 Hz, 2H), 3.78 (s, 3H), 3.57 (s, 4H), 2.63 – 2.49 (m, 2H), 1.79 – 1.70 (m, 1H), 1.55 – 1.47 (m, 1H), 0.99 – 0.96 (m, 1H), 0.98 (s, 3H), 0.95 (s, 6H). **^{13}C{^{1}H} NMR** (100 MHz, $CDCl_3$): δ = 157.6, 135.5, 129.5, 113.7, 72.0, 55.4, 35.7, 34.6, 31.7, 21.9, 15.8. **^{11}B{^{1}H} NMR** (128 MHz, $CDCl_3$): δ = 29.7. **HRMS-ASAP** (m/z): Calculated (found) for $C_{16}H_{26}BO_3$ $[M+H]^+$ 277.1970 (277.1966).

The spectroscopic data for **31b** match those reported in the literature.[25]

2-((9*Z*,12*Z*)-Octadeca-9,12-dien-1-yl)- 5,5-dimethyl-1,3,2-dioxaborinane 32b

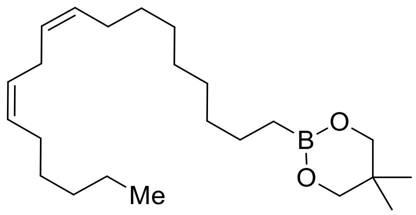

32b

According to **General procedure 5** with **32a** (78 mg, 0.2 mmol, 1.0 equiv.), the reaction mixture was purified by column chromatography on silica gel (hexane/EtOAc = 95/5) to yield the product **32b** as a colorless oil (48.5 mg, 134 μmol, 67% yield). **^{1}H NMR** (400 MHz, $CDCl_3$): δ = 5.42 – 5.29 (m, 4H), 3.59 (s, 4H), 2.77 (t, J = 6 Hz, 2H), 2.07 – 2.01 (m, 4H), 1.39 – 1.26 (m, 18H), 0.95 (s, 6H), 0.89 (t, J = 7 Hz, 3H), 0.70 (t, J = 7 Hz, 2H). **^{13}C{^{1}H} NMR** (100 MHz, $CDCl_3$): δ = 130.4, 130.3, 128.1, 128.0, 72.1, 32.8, 32.7, 31.8, 31.7, 29.9, 29.7, 29.51, 29.49, 27.4, 27.3, 25.8, 24.3, 22.7, 22.0, 14.2. **^{11}B{^{1}H} NMR** (128 MHz, $CDCl_3$): δ = 29.9. **HRMS-ASAP** (m/z): Calculated (found) for $C_{23}H_{44}BO_2$ $[M+H]^+$ 363.3429 (363.3424). **Anal.** for $C_{23}H_{43}BO_2$ calcd: C, 76.23; H, 11.96. found: C, 76.44; H, 12.18.

2-((*R*)-4-((3*R*,5*R*,8*R*,9*S*,10*S*,13*R*,14*S*,17*R*)-3-methoxy-10,13-dimethylhexadecahydro-1*H*-cyclopenta[*a*]phenanthren-17-yl)pentyl)-5,5-dimethyl-1,3,2-dioxaborinane 33b

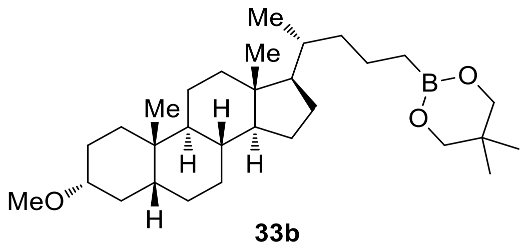

33b

According to **General procedure 5** with **33a** (100 mg, 0.2 mmol, 1.0 equiv.), the reaction mixture was purified by column chromatography on silica gel (hexane/EtOAc = 93/7) to yield the product **33b** as a colorless oil (67 mg, 142 μmol, 71% yield). **^{1}H NMR** (400 MHz, $CDCl_3$): δ = 3.58 (s, 4H), 3.34 (s, 3H),

3.19 – 3.11 (m, 1H), 1.96 – 1.92 (m, 1H), 1.88 – 1.73 (m, 4H), 1.70 – 1.47 (m, 4H), 1.42 – 0.99 (m, 19H), 0.95 (s, 6H), 0.90 (s, 3H), 0.88 (d, *J* = 6 Hz, 3H), 0.75 – 0.63 (m, 2H), 0.61 (s, 3H). **^{13}C{^{1}H} NMR** (100 MHz, $CDCl_3$): δ = 80.6, 72.1, 56.6, 56.2, 55.7, 42.8, 42.2, 40.4, 40.3, 39.1, 36.0, 35.9, 35.4, 35.0, 32.9, 31.7, 28.4, 27.5, 26.9, 26.5, 24.4, 23.6, 22.0, 20.9, 20.7, 18.3, 12.1. **^{11}B{^{1}H} NMR** (128 MHz, $CDCl_3$): δ = 30.3. **HRMS-ASAP** (m/z): Calculated (found) for $C_{30}H_{54}BO_3$ $[M+H]^+$ 473.4161 (473.4155). **Anal.** for $C_{30}H_{53}BO_3$ calcd: C, 76.25; H, 11.30. found: C, 76.08; H, 11.47.

2-((3*S*,8*S*,9*S*,10*R*,13*R*,14*S*,17*R*)-10,13-dimethyl-17-((*R*)-6-methylheptan-2-yl)-2,3,4,7,8,9,10,11,12,13,14,15,16,17-tetradecahydro-1*H*-cyclopenta[*a*]phenanthren-3-yl)-5,5-dimethyl-1,3,2-dioxaborinane 34b

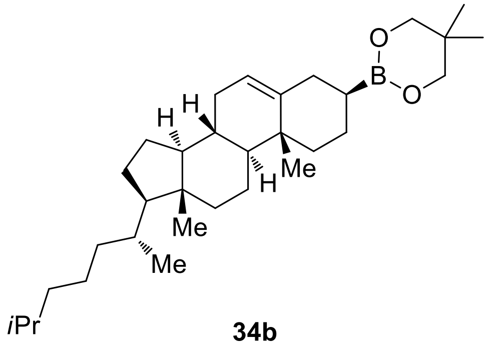

34b

According to **General procedure 5** with **34a** (102 mg, 0.2 mmol, 1.0 equiv.), the reaction mixture was purified by column chromatography on silica gel (hexane/EtOAc = 93/7) to yield the product **34b** as a colorless oil (60 mg, 124 μmol, 62% yield). **^{1}H NMR** (400 MHz, $CDCl_3$): δ = 5.26 – 5.25 (m, 1H), 3.58 (s, 4H), 2.21 – 2.13 (m, 1H), 2.01 – 1.77 (m, 6H), 1.56 – 0.99 (m, 22H), 0.98 (s, 3H), 0.94 (s, 6H), 0.90 (d, *J* = 6 Hz, 3H), 0.86 (dd, *J* = 2, 7 Hz, 6H), 0.66 (s, 3H). **^{13}C{^{1}H} NMR** (100 MHz, $CDCl_3$): δ = 144.5, 118.4, 72.1, 57.0, 56.3, 53.6, 50.7, 42.4, 41.2, 40.0, 39.7, 37.5, 36.3, 35.9, 34.3, 32.1, 32.0, 31.8, 28.4, 28.2, 24.42, 24.37, 24.0, 23.0, 22.7, 21.9, 20.9, 19.7, 18.9, 12.0. **^{11}B{^{1}H} NMR** (128 MHz, $CDCl_3$): δ = 30.7. **HRMS-ASAP** (m/z): Calculated (found) for $C_{32}H_{56}O_2S$ $[M+H]^+$ 483.4368 (483.4366). **Anal.** for $C_{32}H_{55}BO_2$ calcd: C, 79.64; H, 11.49. found: C, 79.81; H, 11.56.

1.5 Unsuccessful Tertiary Sulfone Substrates

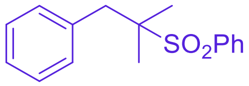

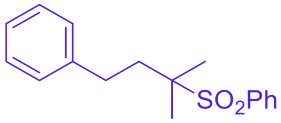

2 Gram-scale Reaction

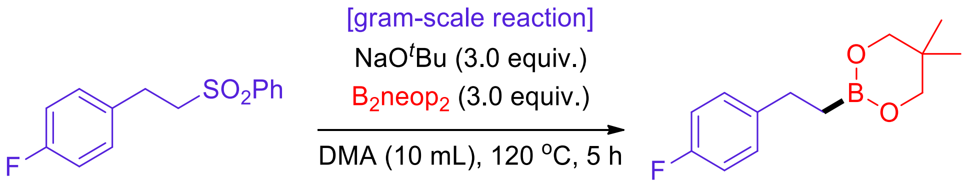

12a, 5 mmol
1.32 g

12b, 72%

In an argon-filled glovebox, the alkyl sulfone **12a** (1.32 g, 5 mmol, 1.0 equiv.), dissolved in DMA (10 mL), was added to a 20 mL Schlenk tube equipped with a magnetic stirring bar. NaO*t*Bu (1.44 mg, 15 mmol, 3.0 equiv.) and B_2neop$_2$ (3.39 g, 15 mmol, 3.0 equiv.) were added. The reaction mixture was stirred at 120 °C for 5 h, then diluted with Et_2O (20 mL) and filtered through a pad of Celite (Ø 3 mm x 8 mm). After careful removal of the solvent *in vacuo*, the reaction mixture was purified by column chromatography on silica gel (hexane/EtOAc (98/2)) to yield the product **12b** as a white solid (0.85 g, 3.6 mmol, 72%).

3 Selective Borylation

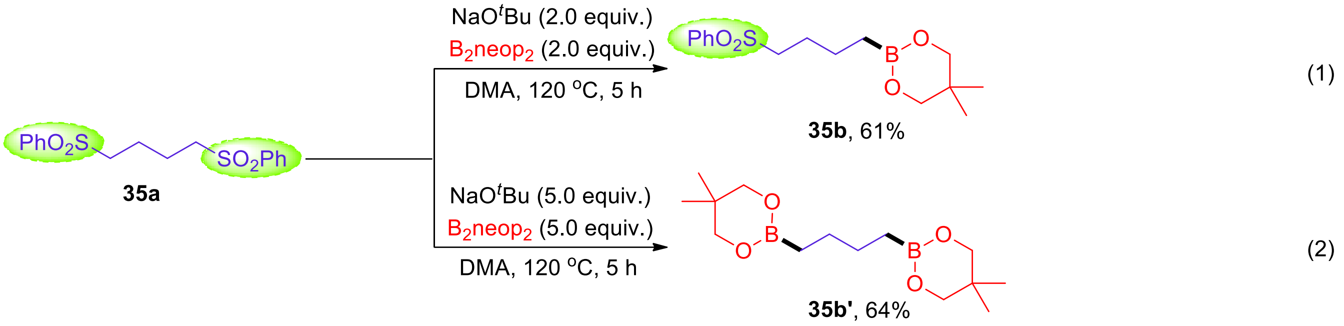

In an argon-filled glovebox, the sulfone **35a** (169 mg, 0.5 mmol, 1.0 equiv.), dissolved in DMA (1 mL), was added to a 10 mL thick-walled reaction tube equipped with a magnetic stirring bar. NaO^{t}Bu (96 mg, 1.0 mmol, 2.0 equiv.) and B_2neop_2 (226 mg, 1.0 mmol, 2.0 equiv.) were added. The reaction mixture was stirred at 120 °C for 5 h, then diluted with Et_2O (2 mL) and filtered through a pad of Celite (Ø 3 mm x 8 mm). After careful removal of the solvent *in vacuo*, the reaction mixture was purified by column chromatography on silica gel (hexane/EtOAc (10/1)) to yield the product **35b** as a white solid (94.6 mg, 305 µmol, 61% yield).

5,5-Dimethyl-2-(4-(phenylsulfonyl)butyl)-1,3,2-dioxaborinane 35b

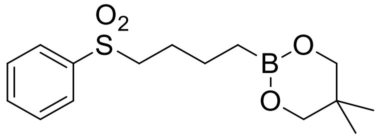

35b

^{1}H NMR (500 MHz, $CDCl_3$): δ = 7.90 – 7.88 (m, 2H), 7.65 – 7.62 (m, 1H), 7.57 – 7.53 (m, 2H), 3.53 (s, 4H), 3.09 – 3.05 (m, 2H), 1.70 – 1.64 (m, 2H), 1.44 – 1.38 (m, 2H), 0.90 (s, 6H), 0.66 (t, J = 8 Hz, 2H). **^{13}C{^{1}H} NMR** (125 MHz, $CDCl_3$): δ = 139.3, 133.6, 129.3, 128.2, 72.1, 56.4, 31.7, 25.2, 23.0, 21.9. **^{11}B{^{1}H} NMR** (160 MHz, $CDCl_3$): δ = 30.1. **HRMS-ASAP** (m/z): Calculated (found) for $C_{15}H_{24}BO_4S$ $[M+H]^+$ 311.1483 (311.1476). **Anal.** for $C_{15}H_{23}BO_4S$ calcd: C, 58.08; H, 7.47; S, 10.34. found: C, 57.82; H, 7.34; S, 10.41.

In an argon-filled glovebox, the sulfone **35a** (169 mg, 0.5 mmol, 1.0 equiv.), dissolved in DMA (1 mL), was added to a 10 mL thick-walled reaction tube equipped with a magnetic stirring bar. NaO^{t}Bu (240 mg, 2.5 mmol, 5.0 equiv.) and B_2neop_2 (565 mg, 2.5 mmol, 5.0 equiv.) were added. The reaction mixture was stirred at 120 °C for 5 h, then diluted with Et_2O (2 mL) and filtered through a pad of Celite (Ø 3 mm x 8 mm). After careful removal of the solvent *in vacuo*, the reaction mixture was purified by column chromatography on silica gel (hexane/EtOAc (97/3)) to yield the product **35b’** as a white solid (90.2 mg, 320 µmol, 64% yield).

1,4-Bis(5,5-dimethyl-1,3,2-dioxaborinan-2-yl)butane 35b'

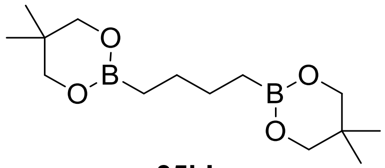

35b'

^{1}H NMR (500 MHz, $CDCl_3$): δ = 3.57 (s, 8H), 1.36 – 1.33 (m, 4H), 0.94 (s, 12H), 0.71 – 0.68 (m, 4H). **^{13}C{^{1}H} NMR** (125 MHz, $CDCl_3$): δ = 72.1, 31.7, 27.3, 22.0. **^{11}B{^{1}H} NMR** (160 MHz, $CDCl_3$): δ = 30.5. **HRMS-ASAP** (m/z): Calculated (found) for $C_{14}H_{29}B_2O_4$ $[M+H]^+$ 283.2246 (283.2240). **Anal.** for $C_{14}H_{28}B_2O_4$ calcd: C, 59.63; H, 10.01. found: C, 59.71; H, 9.88.

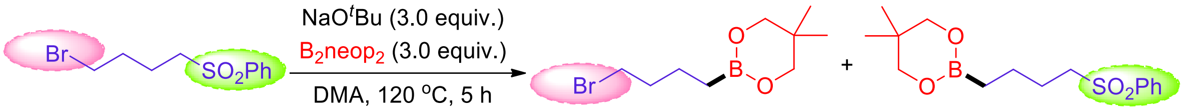

(3)

In an argon-filled glovebox, the sulfone **36a** (138 mg, 0.5 mmol, 1.0 equiv.), dissolved in DMA (1 mL), was added to a 10 mL thick-walled reaction tube equipped with a magnetic stirring bar. NaO^{t}Bu (144 mg, 1.5 mmol, 3.0 equiv.) and B_2neop_2 (339 mg, 1.5 mmol, 3.0 equiv.) were added. The reaction mixture was stirred at 120 °C for 5 h, then diluted with Et_2O (2 mL) and filtered through a pad of Celite (Ø 3 mm x 8 mm). After careful removal of the solvent *in vacuo*, the reaction mixture was purified by column chromatography on silica gel (hexane/EtOAc) to yield the product **36b** as colorless oil (86 mg, 345 µmol, 69% yield) and **35b** as a white solid (12 mg, 40 µmol, 8% yield).

2-(4-Bromobutyl)-5,5-dimethyl-1,3,2-dioxaborinane 36b

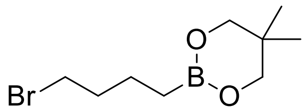

36b

^{1}H NMR (300 MHz, $CDCl_3$): δ = 3.58 (s, 4H), 3.40 (t, J = 7 Hz, 2H), 1.90 – 1.81 (m, 2H), 1.55 – 1.45 (m, 2H), 0.95 (s, 6H), 0.72 (t, J = 8 Hz, 2H). **^{13}C{^{1}H} NMR** (75 MHz, $CDCl_3$): δ = 72.1, 35.6, 34.1, 31.8, 23.0, 22.0. **^{11}B{^{1}H} NMR** (96 MHz, $CDCl_3$): δ = 30.1. **HRMS-ASAP** (m/z): Calculated (found) for $C_9H_{19}BBrO_2$ $[M+H]^+$ 249.0656 (249.0648).

4 Preliminary Mechanistic Investigations

4.1 Radical Trap Experiments

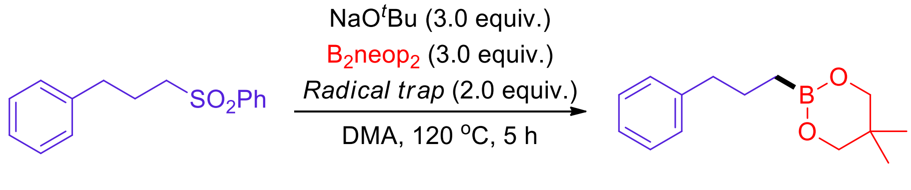

1a-2 **1b**

Radical trap	**1b**
TEMPO	trace
9,10-dihydroanthracene	trace
BHT	trace

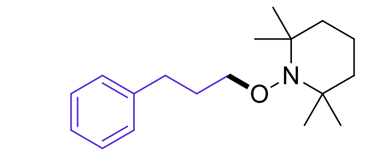

1c, Detected by GC-MS with TEMPO

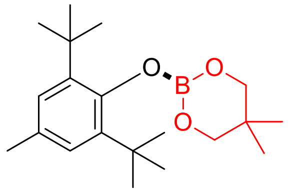

1d, Detected by GC-MS with BHT

In an argon-filled glovebox, ((3-phenylpropyl)sulfonyl)benzene **1a-2** (130 mg, 0.5 mmol, 1.0 equiv.) in DMA (1 mL) was added to a 10 mL thick-walled reaction tube equipped with a magnetic stirring bar. NaO^{t}Bu (144 mg, 1.5 mmol, 3.0 equiv.), B_2neop_2 (339 mg, 1.5 mmol, 3.0 equiv.) and radical trap [TEMPO (117 mg, 0.75 mmol, 1.5 equiv.), 9,10-dihydroanthracene (135 mg, 0.75 mmol, 1.5 equiv.) or BHT (165.3 mg, 0.75 mmol, 1.5 equiv.)] were added. The reaction mixture was stirred at 120 °C for 5 h, then diluted with Et_2O (2 mL) and filtered through a pad of Celite (Ø 3 mm x 8 mm). The solvent was evaporated under reduced pressure, dodecane was added as an internal standard and the crude reaction mixture was analyzed by GC-MS.

1c was detected by GC-MS when TEMPO was added, GC-MS: m/z 260 (M^+-CH_3)[34]. **HRMS-ASAP** (m/z): Calculated (found) for $C_{18}H_{30}NO$ $[M+H]^+$ 276.2322 (276.2316).

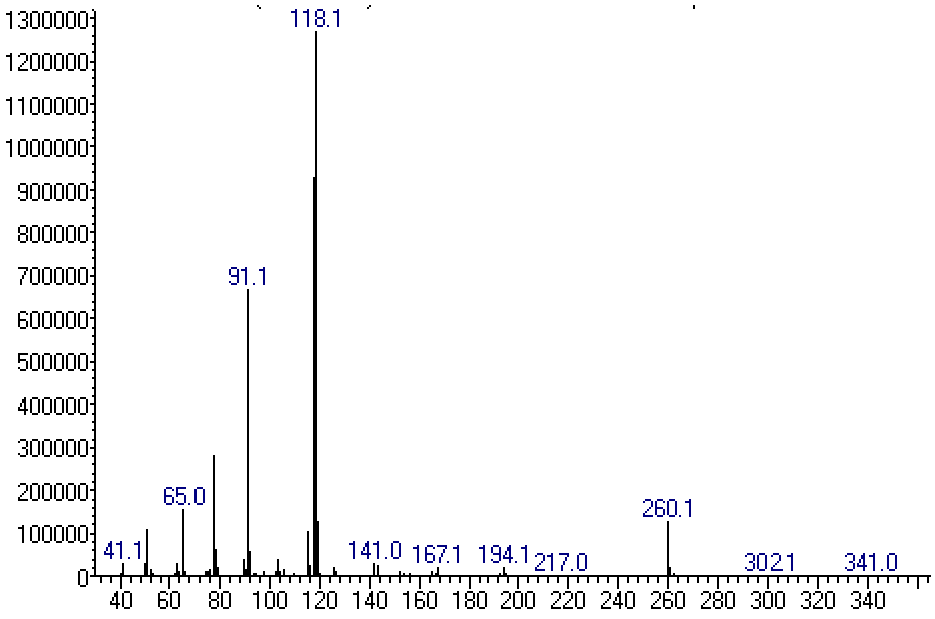

1d was detected by GC-MS when BHT was added, GC-MS: m/z 332 (M^+). **HRMS-ASAP** (m/z): Calculated (found) for $C_{20}H_{33}BO_3$ $[M]^+$ 332.2517 (332.2508).

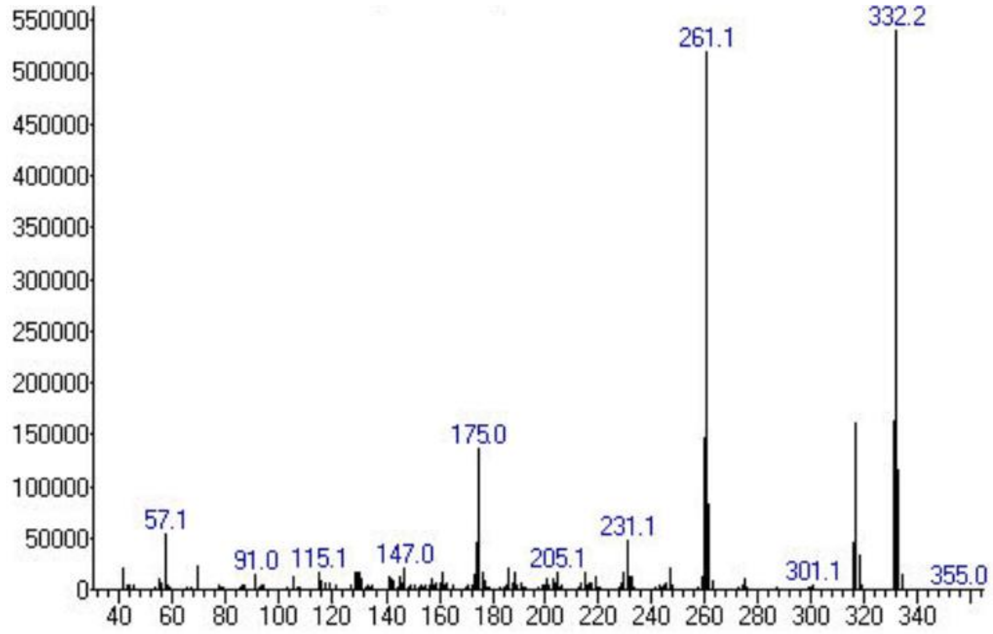

4.2 Radical Clock Experiment

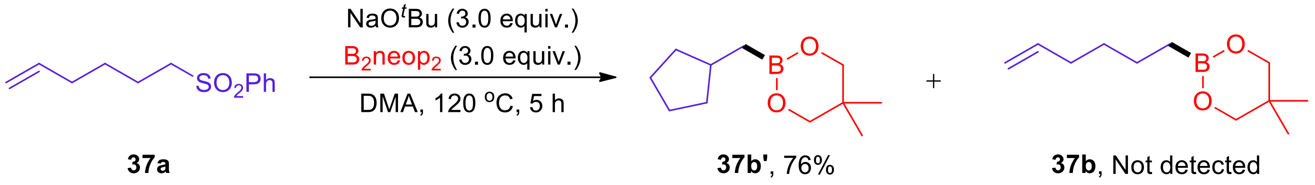

In an argon-filled glovebox, (hex-5-en-1-ylsulfonyl)benzene **37a** (112 mg, 0.5 mmol, 1.0 equiv.) in DMA (1 mL) was added to a 10 mL thick-walled reaction tube equipped with a magnetic stirring bar. NaO^{t}Bu (144 mg, 1.5 mmol, 3.0 equiv.) and B_2neop_2 (339 mg, 1.5 mmol, 3.0 equiv.) were added. The reaction mixture was stirred at 120 °C for 5 h, then diluted with Et_2O (2 mL) and filtered through a pad of Celite (Ø 3 mm x 8 mm). The cyclized product was isolated exclusively in 76% yield following flash column chromatography (hexane/EtOAc (98/2)) after careful removal of the solvent *in vacuo*.

(Hex-5-en-1-ylsulfonyl)benzene 37a

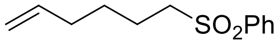

37a

According to **General procedure 1** with hex-5-en-1-ol (300 mg, 3.0 mmol, 1.0 equiv.), the reaction mixture was purified by column chromatography on silica gel (hexane/EtOAc = 10/1) to yield the product **37a** as a colorless oil (505 mg, 2.25 mmol, 75% yield). **^{1}H NMR** (300 MHz, $CDCl_3$): δ = 7.93 – 7.89 (m, 2H), 7.69 – 7.63 (m, 1H), 7.60 – 7.54 (m, 2H), 5.78 – 5.64 (m, 1H), 5.00 – 4.91 (m, 2H), 3.11 – 3.06 (m, 2H), 2.06 – 1.99 (m, 2H), 1.78 – 1.68 (m, 2H), 1.51 – 1.41 (m, 2H). **^{13}C{^{1}H} NMR** (75 MHz, $CDCl_3$): δ = 139.3, 137.6, 133.8, 129.4, 128.2, 115.5, 56.3, 33.2, 27.6, 22.2. **HRMS-ASAP** (m/z): Calculated (found) for $C_{12}H_{17}O_2S$ $[M+H]^+$ 225.0944 (225.0938).

The spectroscopic data for **37a** match those reported in the literature.[35]

2-(Cyclopentylmethyl)-5,5-dimethyl-1,3,2-dioxaborinane 37b'

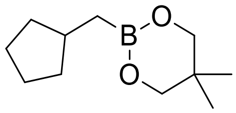

37b'

Yield: 74 mg (380 µmol, 76%) of a colorless oil. **^{1}H NMR** (300 MHz, $CDCl_3$): δ = 3.59 (s, 4H), 1.97 – 1.87 (m, 1H), 1.82 – 1.72 (m, 2H), 1.63 – 1.45 (m, 4H), 1.11 – 1.00 (m, 2H), 0.95 (s, 6H), 0.77 (d, J = 7 Hz, 2H). **^{13}C{^{1}H} NMR** (75 MHz, $CDCl_3$): δ = 72.1, 36.4, 35.3, 31.8, 25.3, 22.0. **^{11}B{^{1}H} NMR** (96 MHz, $CDCl_3$): δ = 30.2. **HRMS-ASAP** (m/z): Calculated (found) for $C_{11}H_{22}BO_2$ $[M+H]^+$ 197.1707 (197.1706).

The spectroscopic data for **37b'** match those reported in the literature.[26]

4.3 EPR Spectroscopic Study

X-band EPR measurements (9.38 GHz) were carried out at room temperature using a Bruker ELEXSYS E580 CW EPR spectrometer. CW EPR spectra were measured using 2 mW microwave power and 0.5 G field modulation at 100 kHz, with a conversion time of 20 ms. The spectral simulations were performed using MATLAB R2021a and the EasySpin 6.0.0 toolbox.[36]

Sample (a)

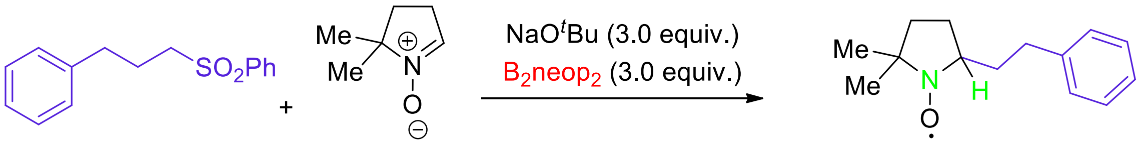

1a-2 **DMPO** **1a-2-DMPO**

Sample (b)

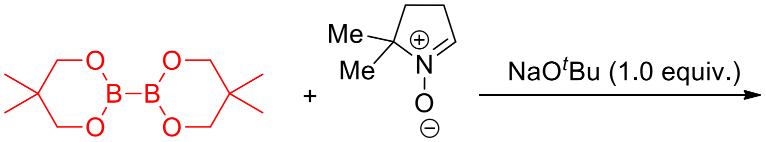

DMPO

Sample (c)

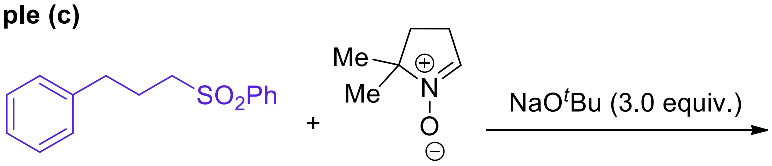

1a-2 **DMPO**

Sample (d)

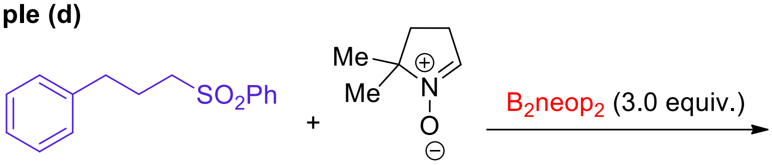

1a-2 **DMPO**

In an argon-filled glovebox, each of the samples was prepared in a 10 mL thick-walled reaction tube equipped with a magnetic stirring bar and stirred for 10 min at 120 °C. The reaction mixtures were transferred into Young's tap tubes. In sample (a), **1a-2** (13 mg, 0.05 mmol, 1.0 equiv.), B_2neop$_2$ (33.9 mg, 0.15 mmol, 3.0 equiv.), NaO^{t}Bu (14.4 mg, 0.15 mmol, 3.0 equiv.), DMPO (11.3 mg, 0.10 mmol, 2.0 equiv.) and dry 1,4-dioxane (0.5 mL) were used. In sample (b), B_2neop$_2$ (33.9 mg, 0.15 mmol, 1.0 equiv.), NaO^{t}Bu (14.4 mg, 0.15 mmol, 1.0 equiv.), DMPO (17.0 mg, 0.15 mmol, 1.0 equiv.) and dry 1,4-dioxane (0.5 mL) were used. In sample (c), **1a-2** (13 mg, 0.05 mmol, 1.0 equiv.), NaO^{t}Bu (14.4 mg, 0.15 mmol, 3.0 equiv.), DMPO (11.3 mg, 0.10 mmol, 2.0 equiv.) and dry 1,4-dioxane (0.5 mL) were used. In sample (d), **1a-2** (13 mg, 0.05 mmol, 1.0 equiv.), B_2neop$_2$ (33.9 mg, 0.15 mmol, 3.0 equiv.), DMPO (11.3 mg, 0.10 mmol, 2.0 equiv.) and dry 1,4-dioxane (0.5 mL) were used.

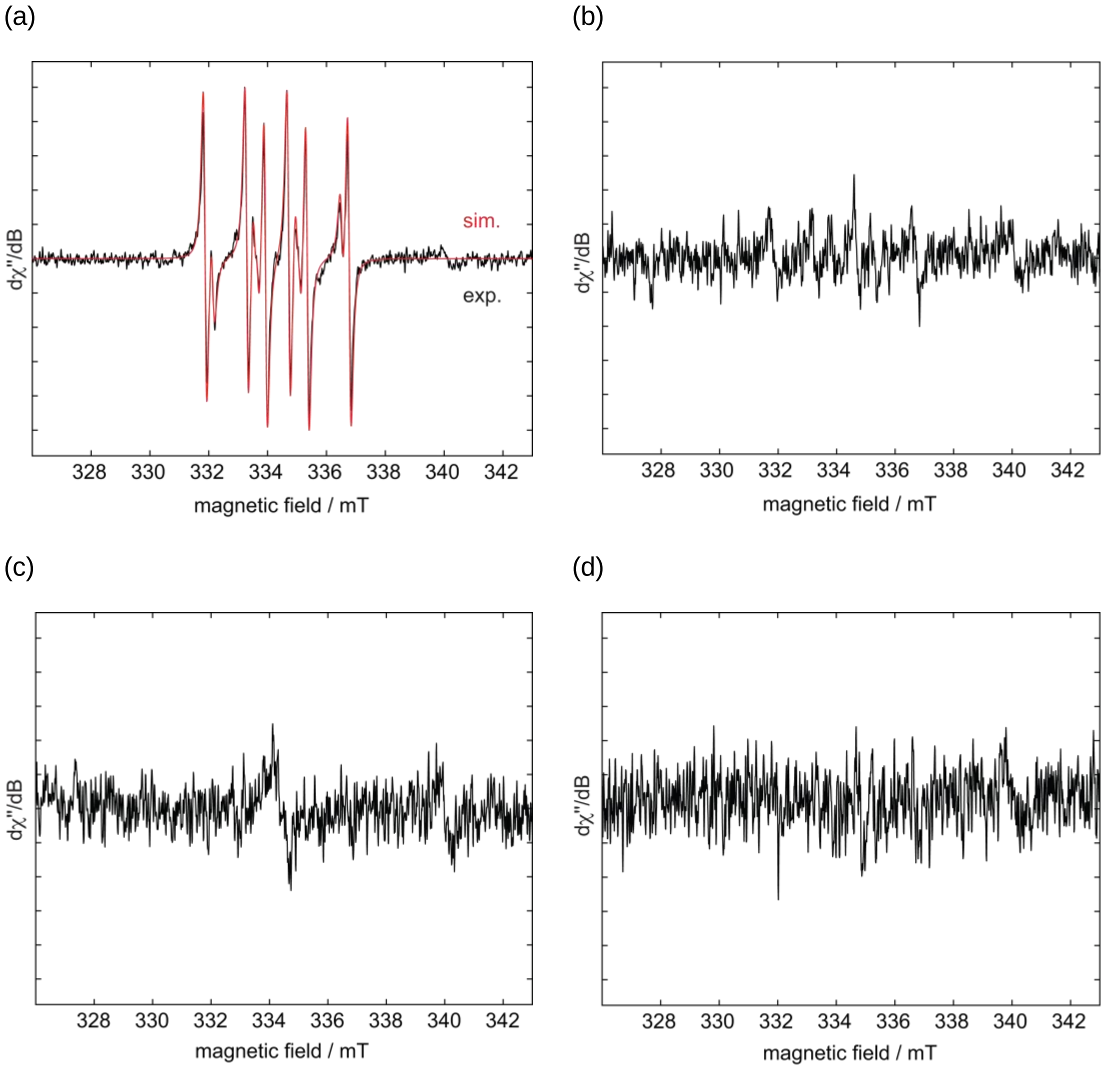

Figure S1. (a) Experimental (black) and simulated (red) continuous-wave (CW) X-band EPR spectra of the DMPO spin trapping experiment in 1,4-dioxane at room temperature: **1a-2** + B_2neop$_2$ + NaO^{t}Bu. Best-fit simulation parameters: g_{iso} = 2.0053, $a(^{14}N)$ = 40 MHz (14.3 G) and $a(^{1}H)$ = 58 MHz (20.6 G, major species); g_{iso} = 2.0053, $a(^{14}N)$ = 40 MHz (14.3 G) and $a(^{1}H)$ = 43 MHz (15.4 G, minor species). **(b)** CW X-band EPR spectra of the DMPO spin trapping experiment in 1,4-dioxane at room temperature: B_2neop$_2$ + NaO^{t}Bu. **(c)** CW X-band EPR spectra of the DMPO spin trapping experiment in 1,4-dioxane at room temperature: **1a-2** + NaO^{t}Bu. **(d)** CW X-band EPR spectra of the DMPO spin trapping experiment in 1,4-dioxane at room temperature: **1a-2** + B_2neop$_2$.

5 Single-Crystal X-ray Diffraction Analysis

A crystal suitable for single-crystal X-ray diffraction was selected, coated in perfluoropolyether oil, and mounted on a microloop. Diffraction data of **34b** was collected on a RIGAKU OXFORD DIFFRACTION XTALAB Synergy diffractometer with a semiconductor HPA-detector (HyPix-6000) and multi-layer mirror monochromated Cu-K_α radiation. The crystal was cooled at 100 K using an Oxford Cryostream 800 low-temperature device. The images were processed and corrected for Lorentz-polarization effects and absorption as implemented in the CrysAlis^{Pro} software. The structure was solved using the intrinsic phasing method (SHELXT)[37] and Fourier expansion technique. All non-hydrogen atoms were refined in anisotropic approximation, with hydrogen atoms 'riding' on idealised positions by full-matrix least squares against F^2 of all data, using SHELXL software[38] and the SHELXLE graphical user interface[39]. The crystal structure was solved as a superstructure of a smaller, commensurately modulated monoclinic structure with lattice parameters a = 12.7032 Å, b = 9.1345 Å, c = 12.9947 Å, and β = 103.01°, with a modulation (q) vector of [0.25, 0, 0.5] and is obtained from the smaller cell by applying the transformation matrix [2 0 1, 0 -1 0, 0 0 -2]. The smaller unit cell is obtained from the large one by applying the transformation matrix [0.5 0 0.25, 0 -1 0, 0 0 -0.5]. Diamond[40] software was used for graphical representation. Crystal data and experimental details are listed in Table S3; full structural information has been deposited with Cambridge Crystallographic Data Centre. CCDC-2079501. These data can be obtained free of charge from The Cambridge Crystallographic Data Centre *via* www.ccdc.cam.ac.uk/data_request/cif.

Table S3: Single-crystal X-ray diffraction data and refinement details of **34b**.

Data	**34b**
CCDC number	2079501
Empirical formula	$C_{32}H_{55}BO_2$
Formula weight / g·mol^{-1}	482.57
T / K	100(2)
Radiation, λ / Å	Cu-K$_\alpha$ 1.54184
Crystal size / mm^3	0.03×0.07×0.17
Crystal color, habit	Colourless needle
μ / mm^{-1}	0.485
Crystal system	Monoclinic
Space group	$P2_1$
a / Å	25.8010(4)
b / Å	9.13450(10)
c / Å	25.9893(4)
α / °	90
β / °	106.377(2)
γ / °	90
Volume / Å^3	5876.62(15)
Z	8
ρ_{calc} / g·cm^{-3}	1.091
F(000)	2144
θ range / °	2.131 – 68.249
Reflections collected	37525
Unique reflections	18290
Parameters / restraints	1324 / 76
GooF on F^2	1.026
R_1 [I>2σ (I)]	0.0482
wR^2 (all data)	0.1307
Max. / min. residual electron density / e·Å^{-3}	0.356 / –0.183

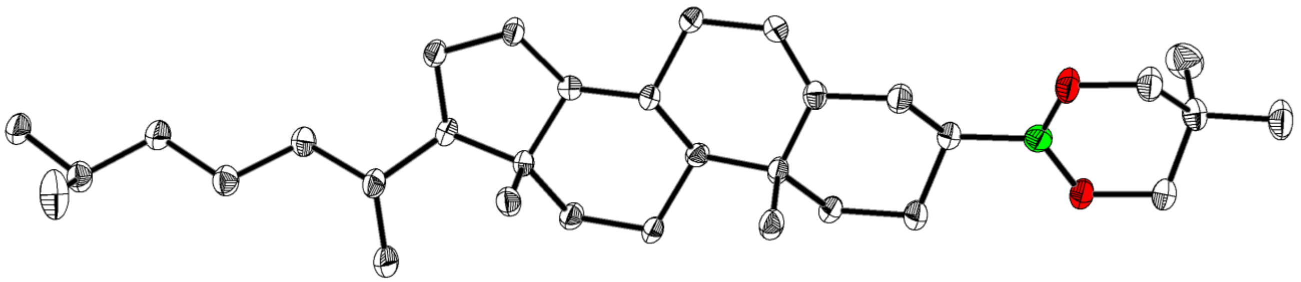

Figure S2. The solid-state molecular structure of **34b** was determined by single-crystal X-ray diffraction at 100 K. All ellipsoids are drawn at the 50% probability level, and H atoms are omitted for clarity. One of four symmetry-independent molecules is shown here. Atom colours: carbon (white), boron (green), oxygen (red).

6 References

[1] A. Fürstner, S. Flügge, O. Larionov, Y. Takahashi, T. Kubota, J. Kobayashi, *Chem. Eur. J.* **2009**, *15*, 4011-4029.

[2] T. Murakami, K. Furusawa, *Synthesis* **2002**, *4*, 479-482.

[3] D.-K. Kim, H.-S. Um, H. Park, S. Kim, J. Choi, C. Lee, *Chem. Sci.* **2020**, *11*, 13071-13078.

[4] X.-J. Dai, H. Wang, C.-J. Li, *Angew. Chem. Int. Ed.* **2017**, *56*, 6302-6306; *Angew. Chem.* **2017**, *129*, 6399-6403.

[5] J. Chen, J.-H. Lin, J.-C. Xiao, *Chem. Commun.* **2018**, *54*, 7034-7037.

[6] M. Nambo, C. M. Crudden, *Angew. Chem. Int. Ed.* **2014**, *53*, 742-746.

[7] D. Zheng, R. Mao, Z. Li, J. Wu, *Org. Chem. Front.* **2016**, *3*, 359-363.

[8] Y. Zheng, Y. You, Q. Shen, J. Zhang, L. Liu, X.-H. Duan, *Org. Chem. Front.* **2020**, *7*, 2069-2074.

[9] P. K. Shyam, H.-Y. Jang, *J. Org. Chem.* **2017***, 82,* 1761-1767.

[10] G. C. Tsui, M. Lautens, *Angew. Chem. Int. Ed.* **2010**, *49*, 8938-8941.

[11] D. R. St. Laurent, L. A. Paquette, *J. Org. Chem.* **1986***, 51,* 3861-3864.

[12] R. C. F. Jones, S. C. Hirst, *Tetrahedron Lett.* **1989**, *30*, 5361-5364.

[13] V. López-Carrillo, A. M. Echavarren, *J. Am. Chem. Soc.* **2010**, *132*, 9292-9294.

[14] D. Li, T.-K. Ma, R. J. Scott, J. D. Wilden, *Chem. Sci.* **2020**, *11*, 5333-5338.

[15] L. Řehová, I. Císařová, U. Jahn, *Eur. J. Org. Chem.* **2014**, 2014, 1461-1476.

[16] J. K. Crandall, C. Pradat, *J. Org. Chem.* **1985***, 50,* 1327-1329.

[17] M. Ociepa, A. J. Wierzba, J. Turkowska, D. Gryko, *J. Am. Chem. Soc.* **2020**, *142*, 5355-5361.

[18] C.-R. Liu, M.-B. Li, D.-J. Cheng, C.-F. Yang, S.-K. Tian, *Org. Lett.* **2009**, *11*, 2543-2545.

[19] S. R. Fletcher, F. Burkamp, P. Blurton, S. K. F. Cheng, R. Clarkson, D. O'Connor, D. Spinks, M. Tudge, M. B. van Niel, S. Patel, K. Chapman, R. Marwood, S. Shepheard, G. Bentley, G. P. Cook, L. J. Bristow, J. L. Castro, P. H. Hutson, A. M. MacLeod, *J. Med. Chem.* **2002**, *45*, 492-503.

[20] R. Scholz, G. Hellmann, S. Rohs, G. Raabe, J. Runsink, D. Özdemir, O. Luche, T. Heß, A. W. Giesen, J. Atodiresei, H. J. Lindner, H.-J. Gais, *Eur. J. Org. Chem.* **2010**, *2010*, 4559-4587.

[21] S. E. Denmark, A. J. Cresswell, *J. Org. Chem.* **2013***, 78,* 12593-12628.

[22] C.-C. Wang, P.-S. Lin, C.-H. Cheng, *Tetrahedron Lett.* **2004**, *45*, 6203-6206.

[23] J. J. Newton, B. J. Jelier, M. Meanwell, R. E. Martin, R. Britton, C. M. Friesen, *Org. Lett.* **2020**, *22*, 1785-1790.

[24] P. Franzmann, S. B. Beil, D. Schollmeyer, S. R. Waldvogel, *Chem. Eur. J.* **2019**, *25*, 1936-1940.

[25] P. K. Verma, K. S. Prasad, D. Varghese, K. Geetharani, *Org. Lett.* **2020**, *22*, 1431-1436.

[26] Z.-F. Jiao, Y.-M. Tian, X.-N. Guo, U. Radius, H. Braunschweig, T. B. Marder, X.-Y. Guo, *J. Catal.* **2021**, *395*, 258-265.

[27] M. Tobisu, H. Kinuta, Y. Kita, E. Rémond, N. Chatani, *J. Am. Chem. Soc.* **2012**, *134*, 115-118.

[28] S. Siddiqui, R. Bhawar, K. Geetharani, *J. Org. Chem.* **2021**, *86*, 1948-1954.

[29] S. Hong, M. Liu, W. Zhang, Q. Zeng, W. Deng, *Tetrahedron Lett.* **2015**, *56*, 2297-2302.

[30] A. Joshi-Pangu, X. Ma, M. Diane, S. Iqbal, R. J. Kribs, R. Huang, C.-Y. Wang, M. R. Biscoe, *J. Org. Chem.* **2012**, *77*, 6629-6633.

[31] X. Lou, Z.-Q. Zhang, J.-H. Liu, X.-Y. Lu, *Chem. Lett.* **2016**, *45*, 200-202.

[32] V. Ganesh, M. Odachowski, V. K. Aggarwal, *Angew. Chem. Int. Ed.* **2017**, *56*, 9752-9756.

[33] X.-F. Zhou, Y.-D. Wu, J.-J. Dai, Y.-J. Li, Y. Huang, H.-J. Xu, *RSC Adv.* **2015**, *5*, 46672-46676.

[34] S. K. Bose, S. Brand, H. O. Omoregie, M. Haehnel, J. Maier, G. Bringmann, T. B. Marder, *ACS Catal.* **2016**, *6*, 8332.

[35] H. Yang, R. G. Carter, *Org. Lett.* **2010**, *12*, 3108-3111.

[36] S. Stoll, A. Schweiger, *J. Magn. Reson.* **2006**, *178*, 42-55.

[37] G. M. Sheldrick, *Acta Crystallogr.* **2015**, *A71*, 3-8.

[38] G. M. Sheldrick, *Acta Crystallogr.* **2015**, *C71*, 3-8.

[39] C. B. Hübschle, G. M. Sheldrick, B. Dittrich, *J. Appl. Crystallogr.* **2011**, *44*, 1281-1284

[40] K. Brandenburg, Diamond (version 4.4.0), Crystal and Molecular Structure Visualization, Crystal Impact H. Putz & K. Brandenburg GbR, Bonn (Germany), **2017**.

7 NMR Spectra

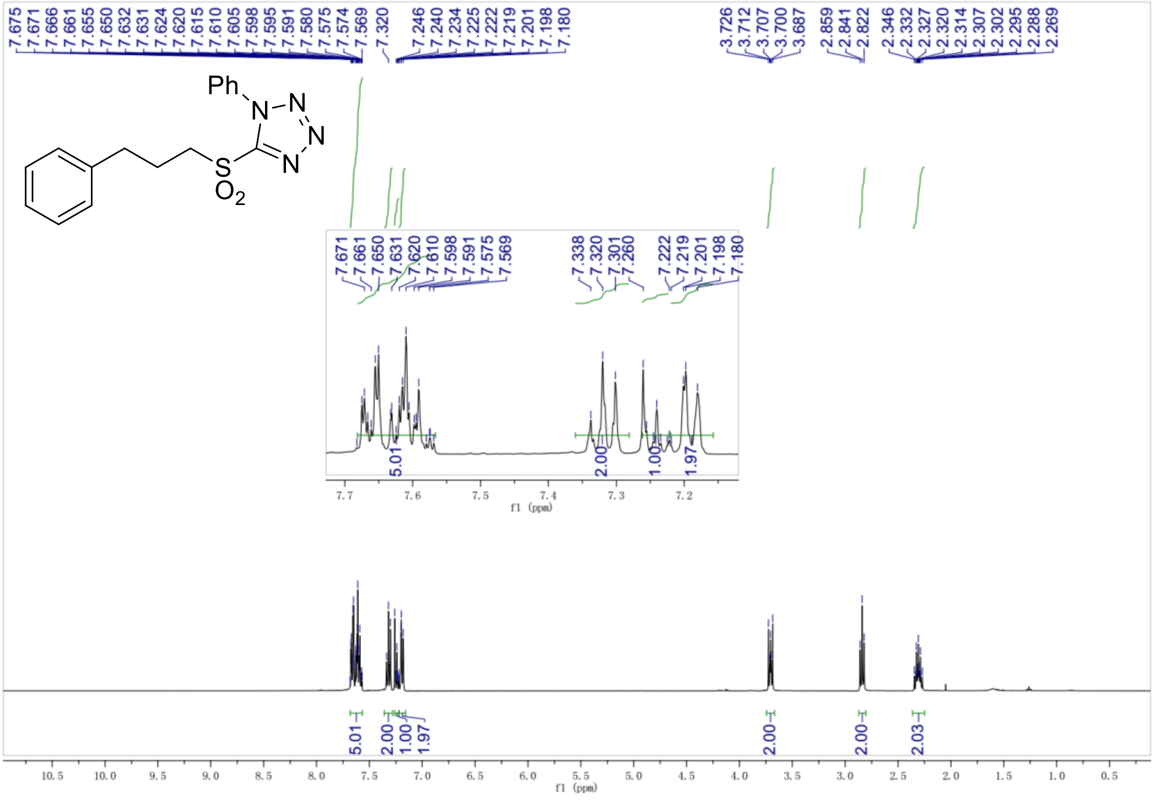

^{1}H NMR spectrum of compound **1a-1** in $CDCl_3$ (400 MHz).

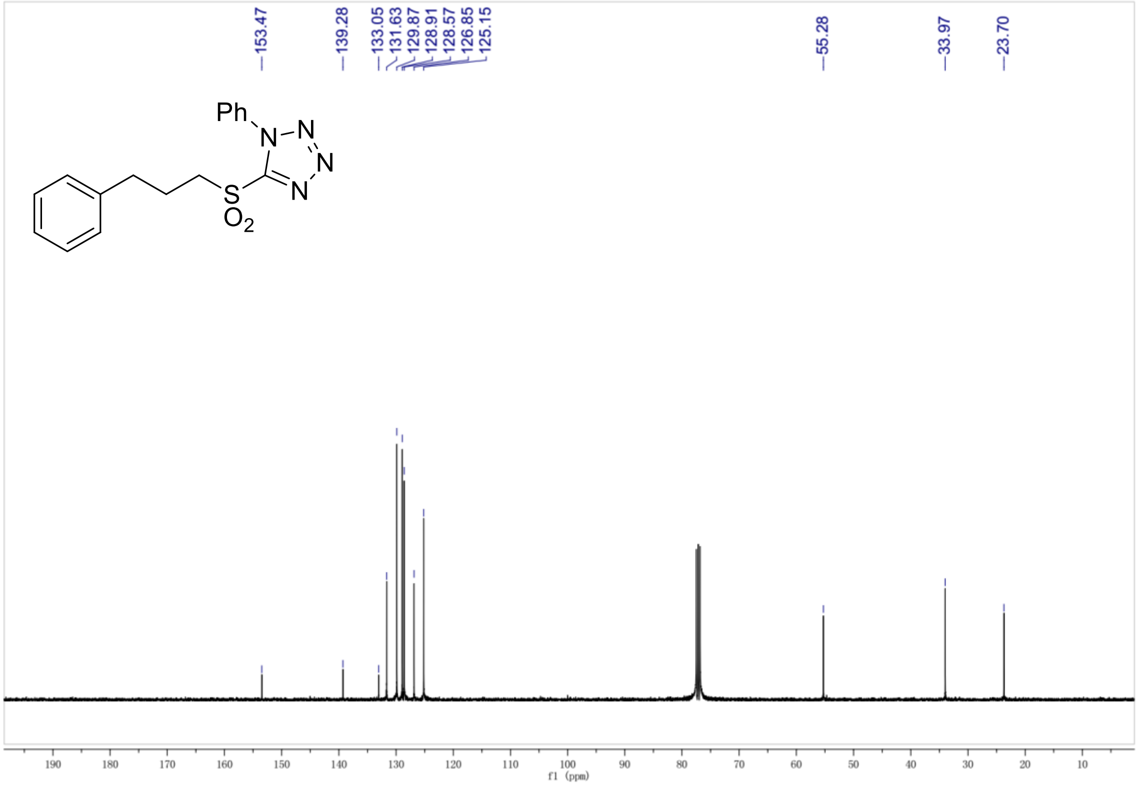

^{13}C{^{1}H} NMR spectrum of compound **1a-1** in $CDCl_3$ (100 MHz).

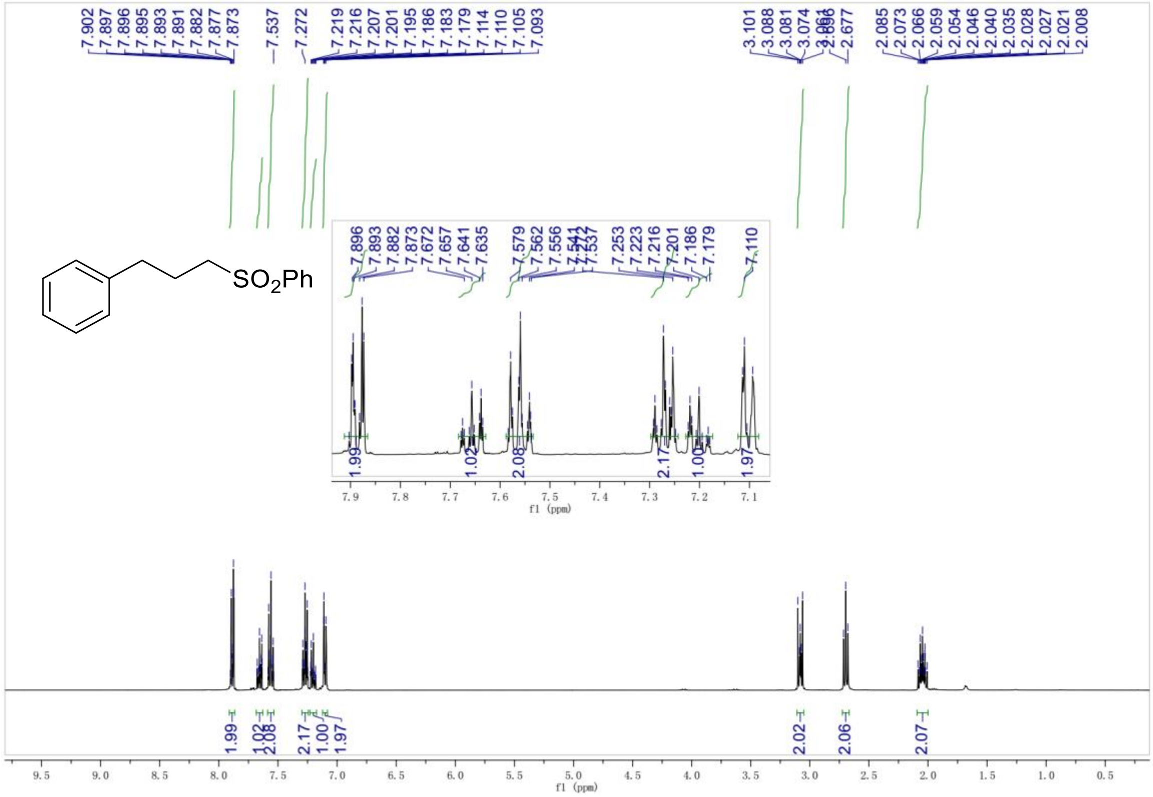

^{1}H NMR spectrum of compound **1a-2** in $CDCl_3$ (400 MHz).

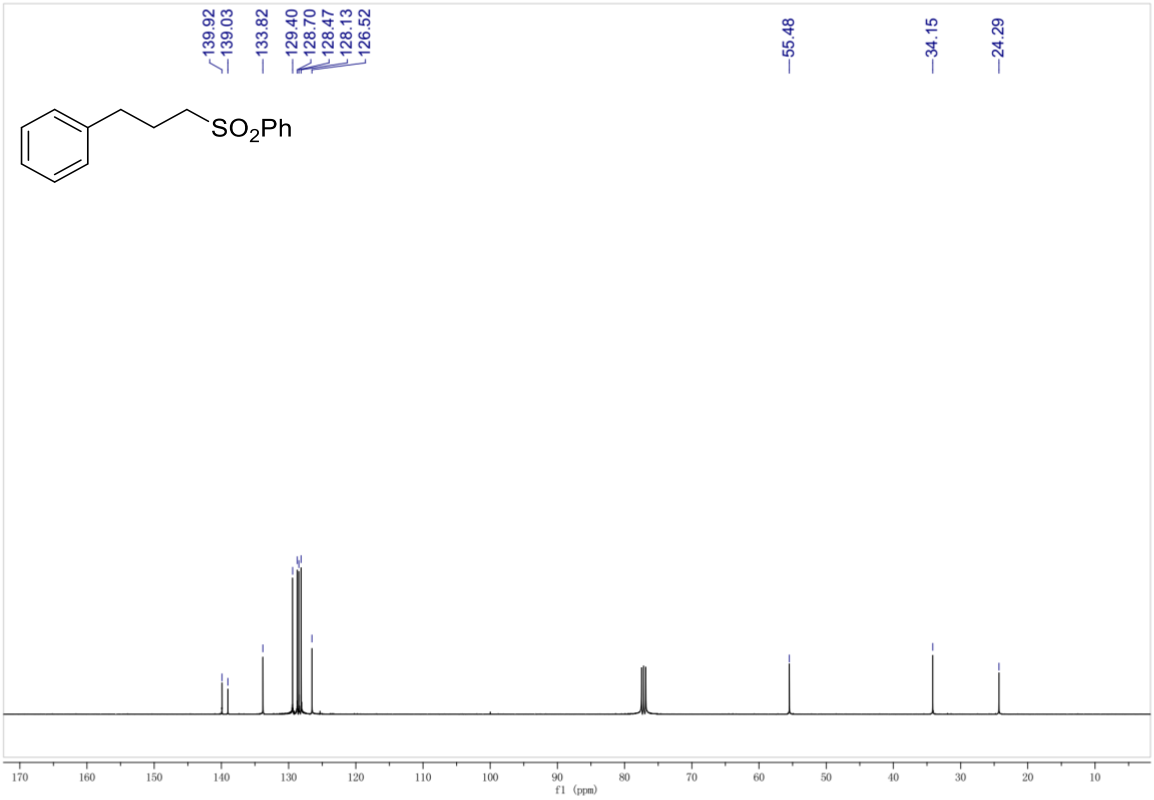

^{13}C{^{1}H} NMR spectrum of compound **1a-2** in $CDCl_3$ (100 MHz).

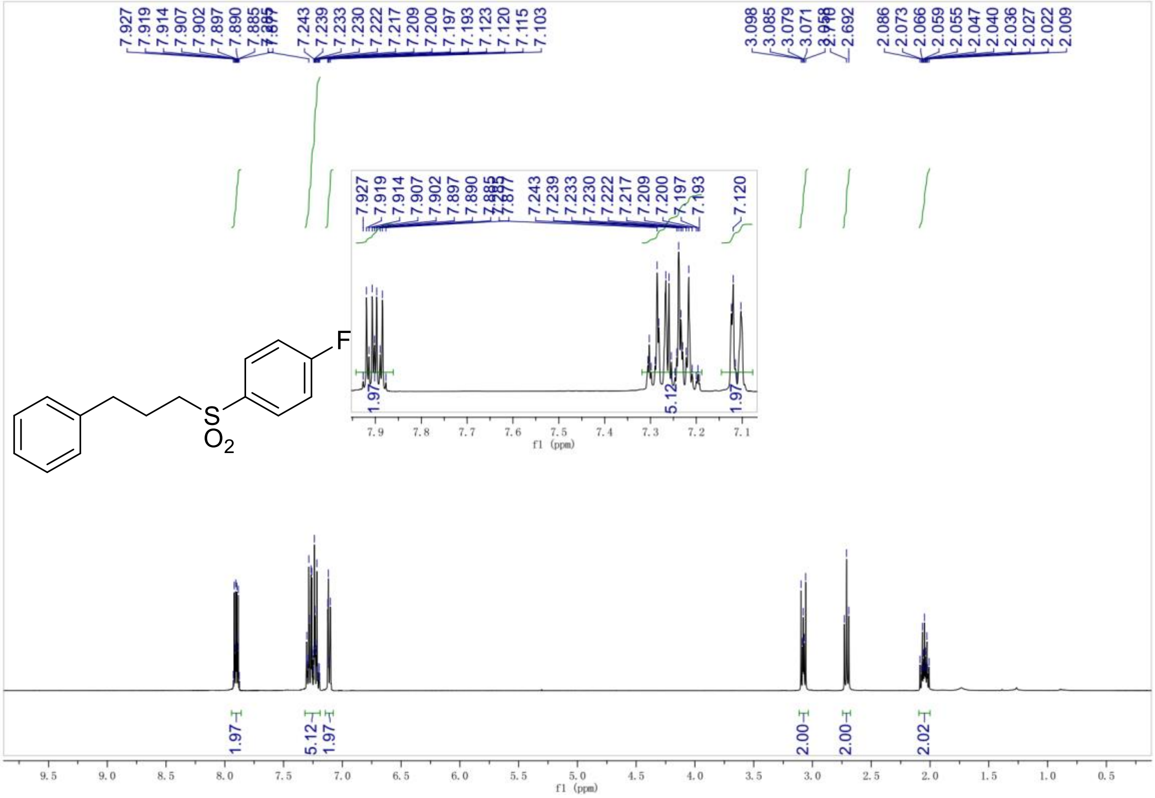

^{1}H NMR spectrum of compound **1a-3** in $CDCl_3$ (400 MHz).

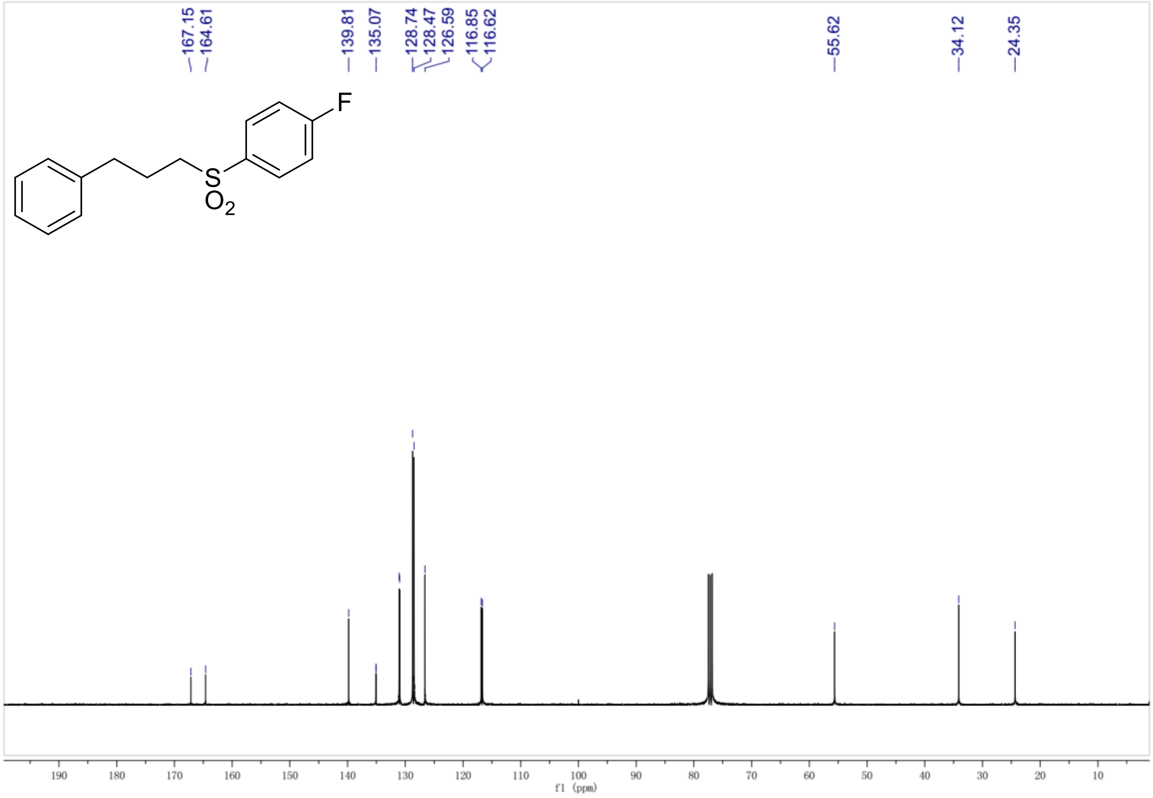

^{13}C{^{1}H} NMR spectrum of compound **1a-3** in $CDCl_3$ (100 MHz).

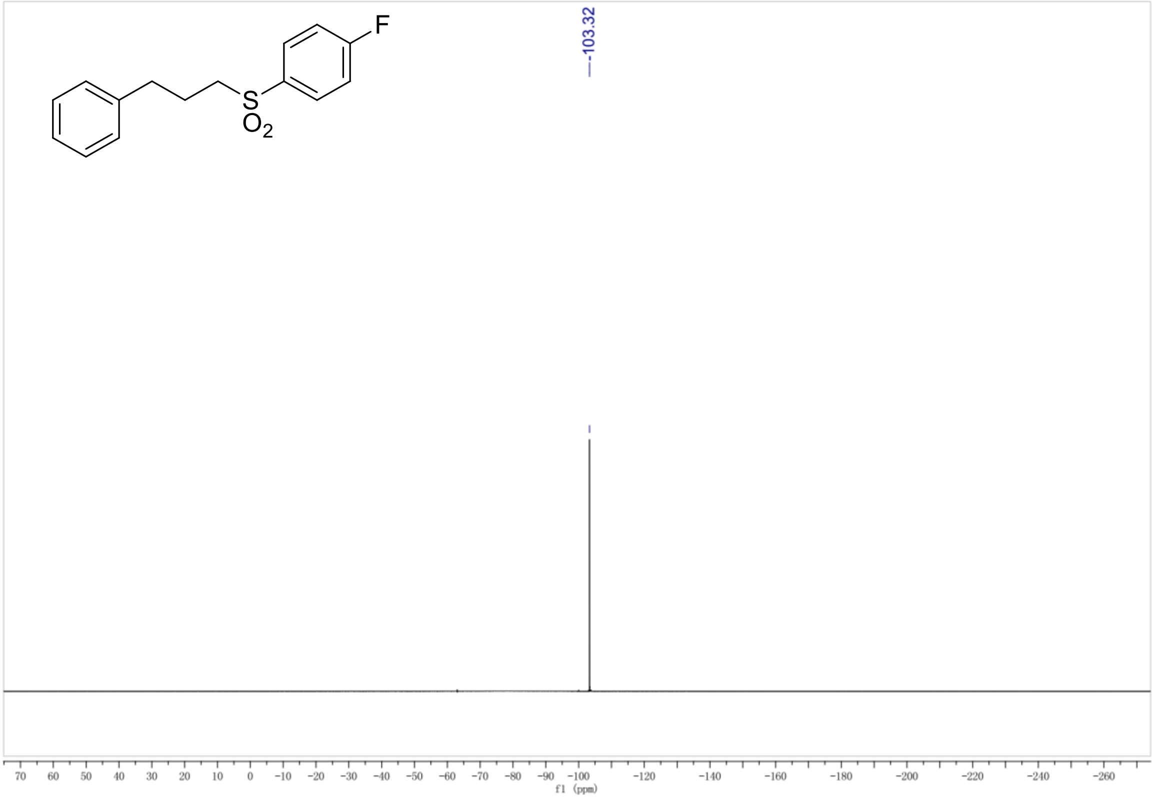

$^{19}F\{^{1}H\}$ NMR spectrum of compound **1a-3** in $CDCl_3$ (376 MHz).

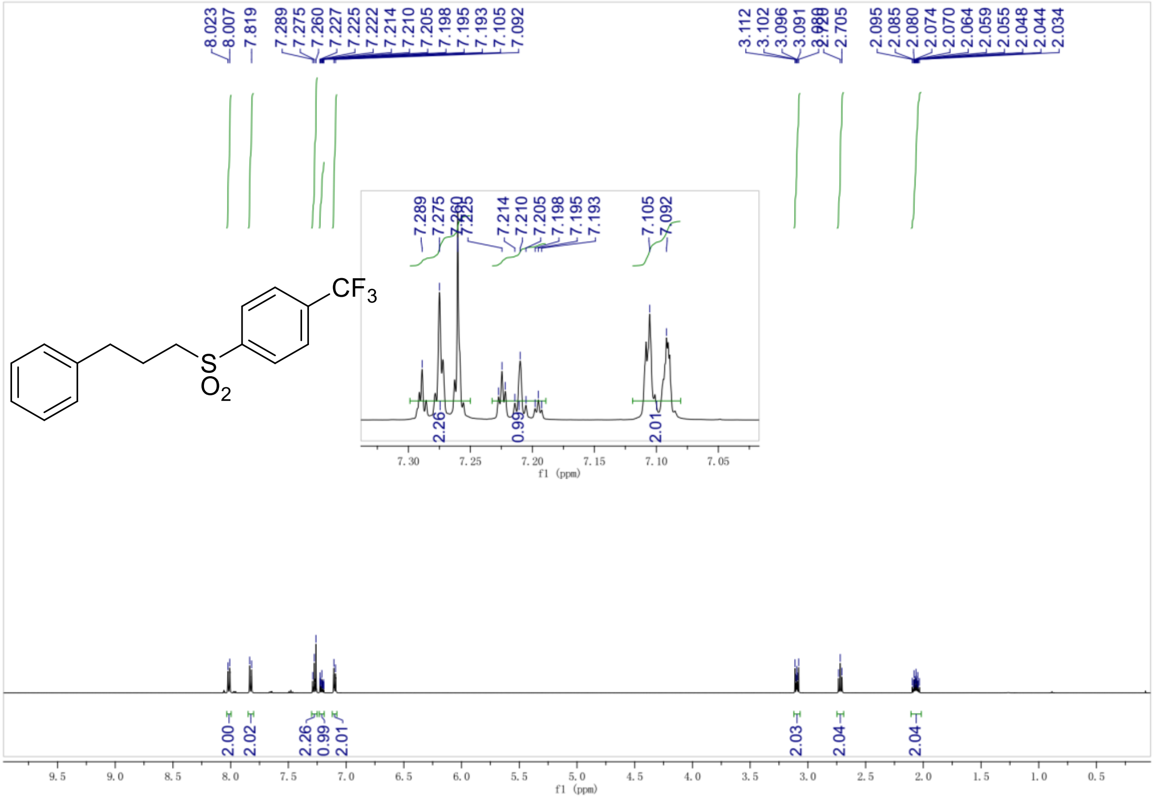

^{1}H NMR spectrum of compound **1a-4** in $CDCl_3$ (500 MHz).

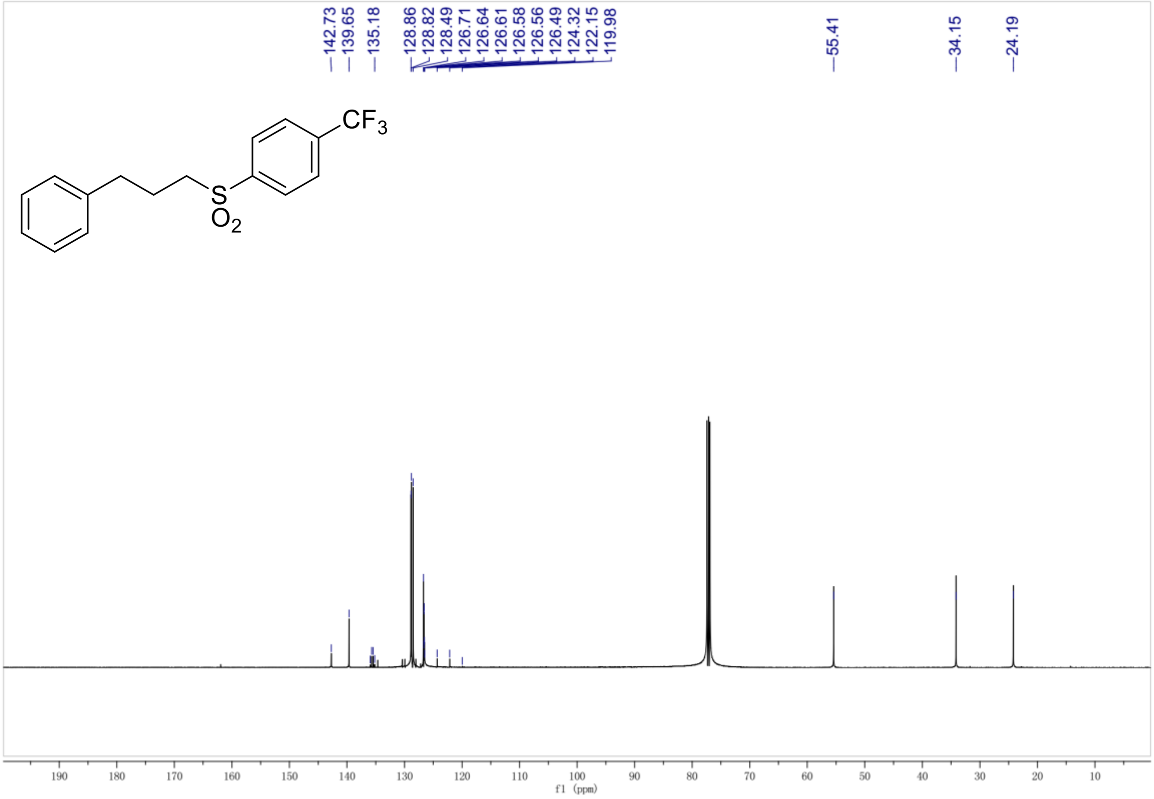

$^{13}C\{^{1}H\}$ NMR spectrum of compound **1a-4** in $CDCl_3$ (125 MHz).

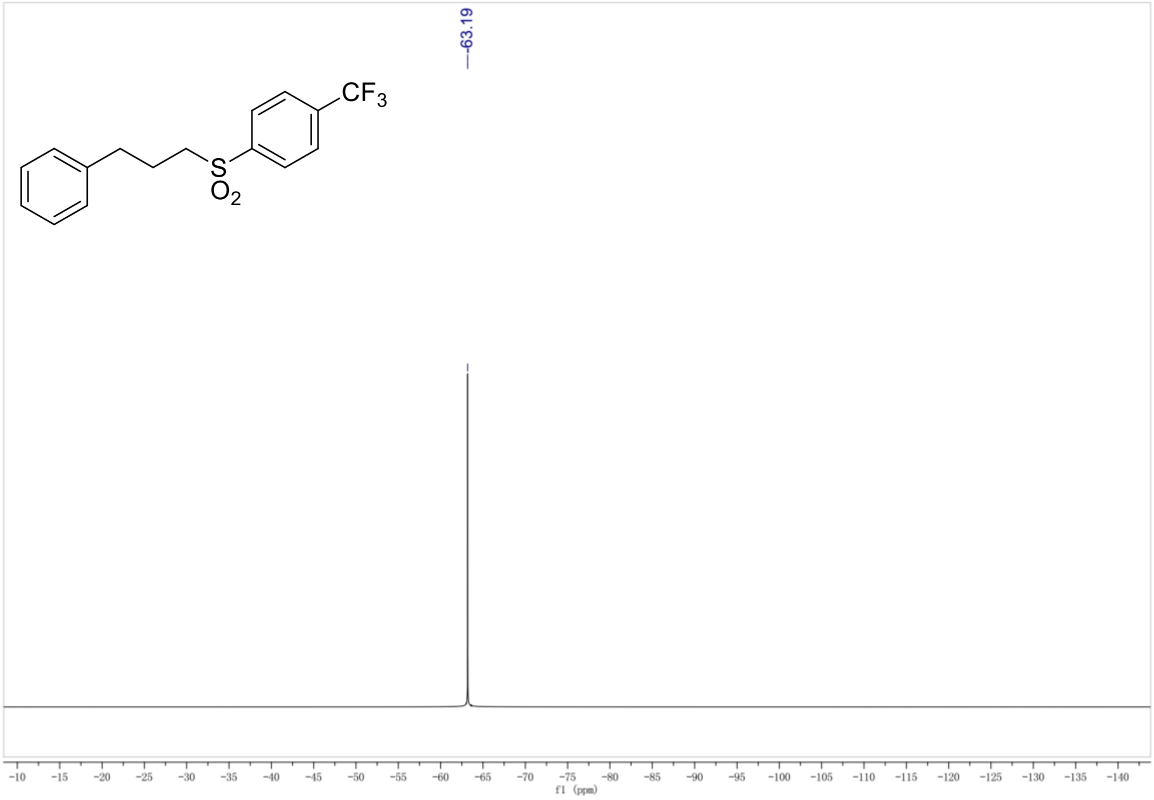

$^{19}F\{^{1}H\}$ NMR spectrum of compound **1a-4** in $CDCl_3$ (470 MHz).

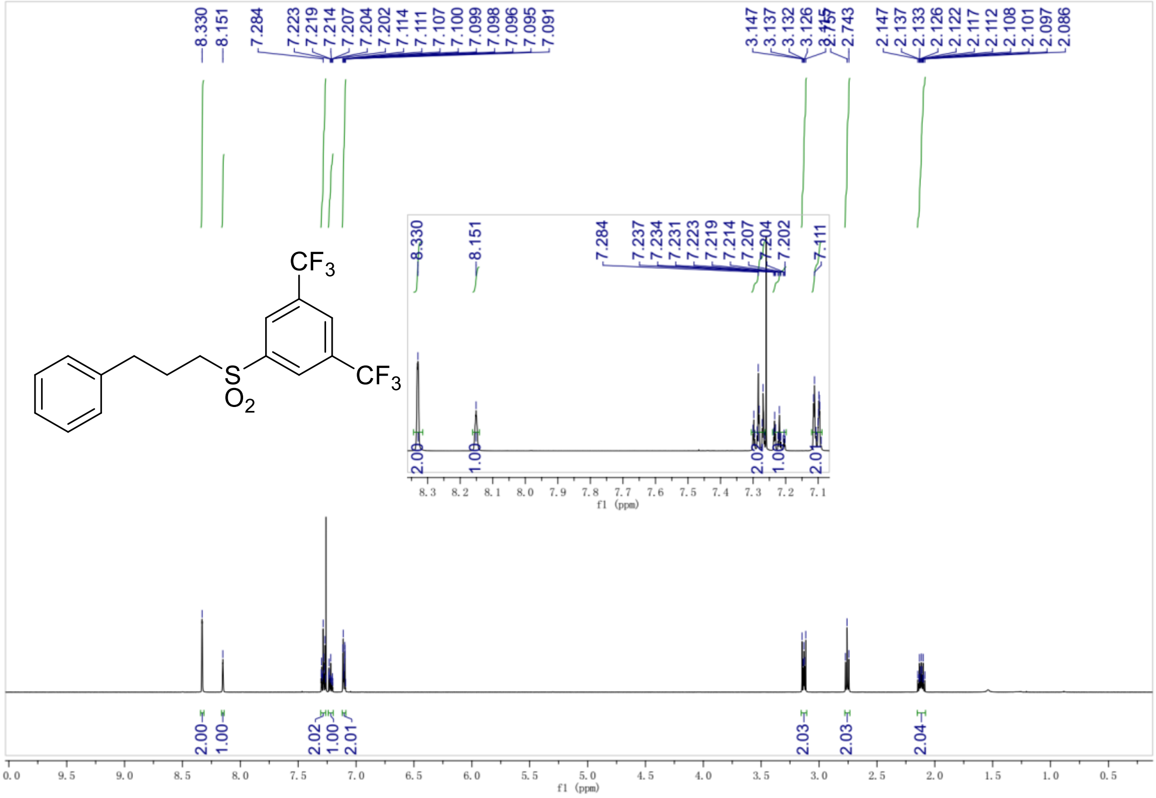

^{1}H NMR spectrum of compound **1a-5** in $CDCl_3$ (500 MHz).

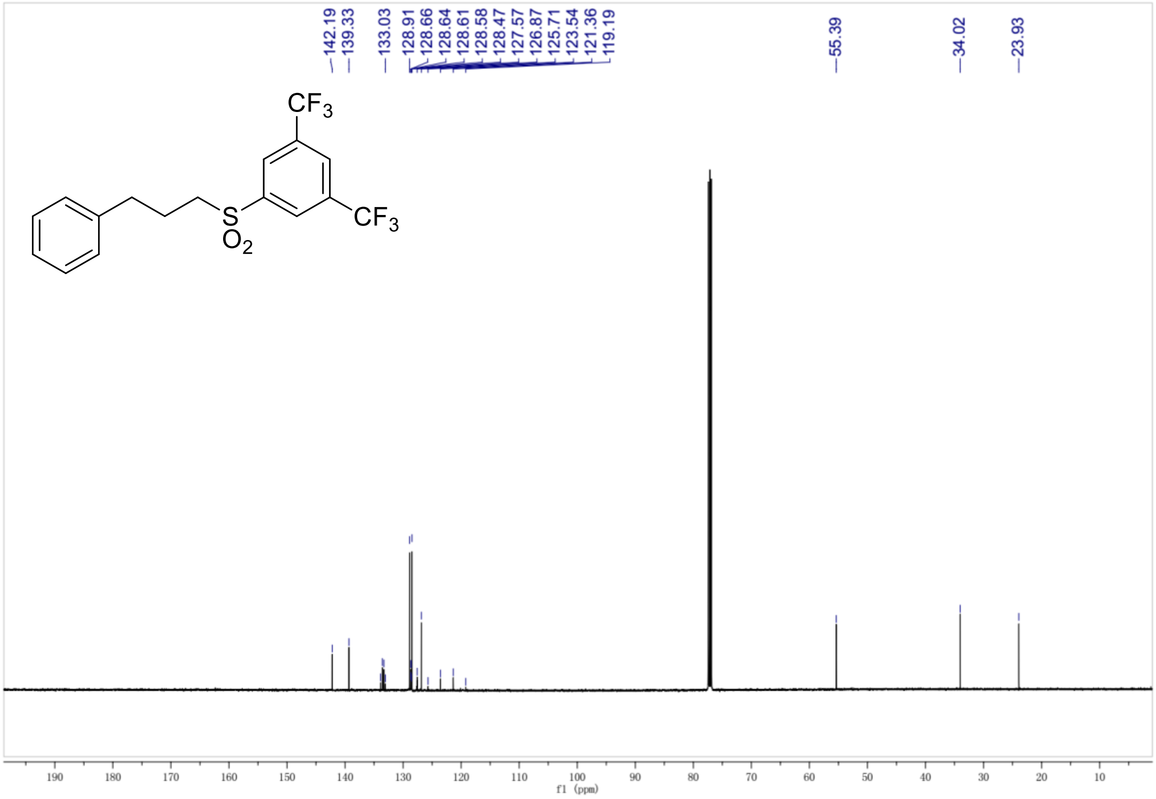

$^{13}C\{^{1}H\}$ NMR spectrum of compound **1a-5** in $CDCl_3$ (125 MHz).

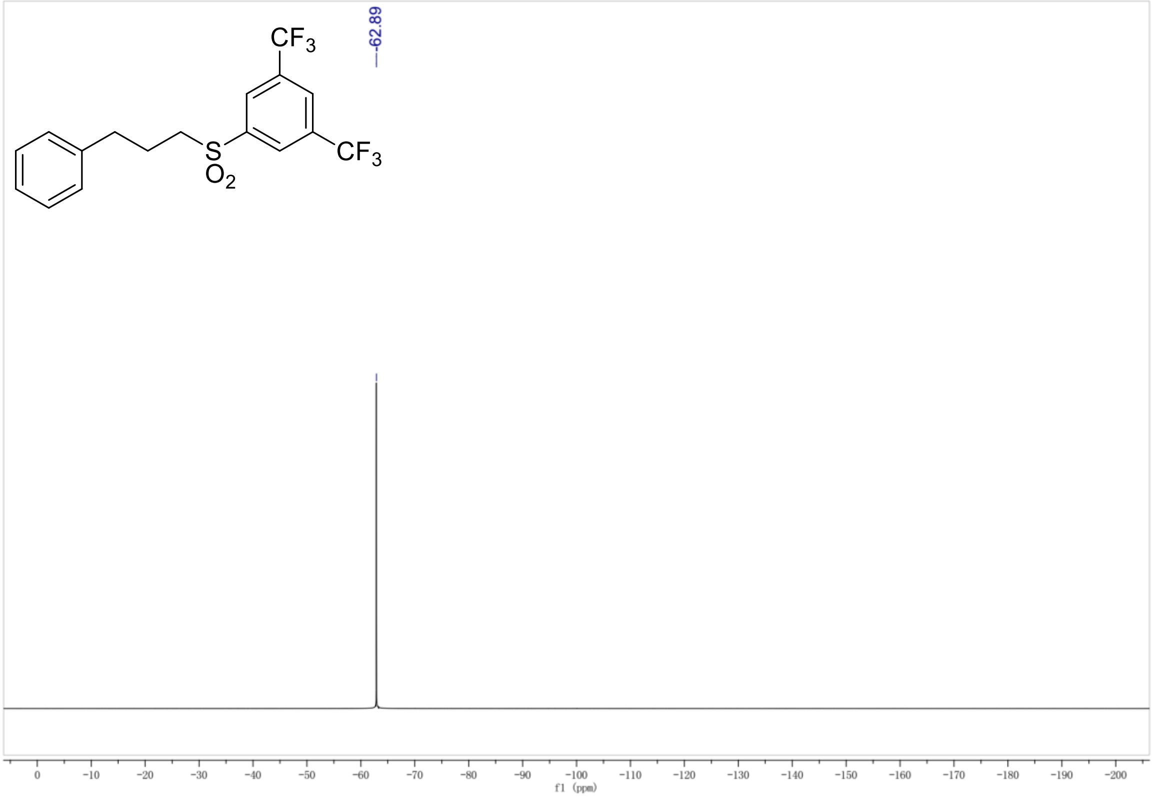

^{19}F{^{1}H} NMR spectrum of compound **1a-5** in $CDCl_3$ (470 MHz).

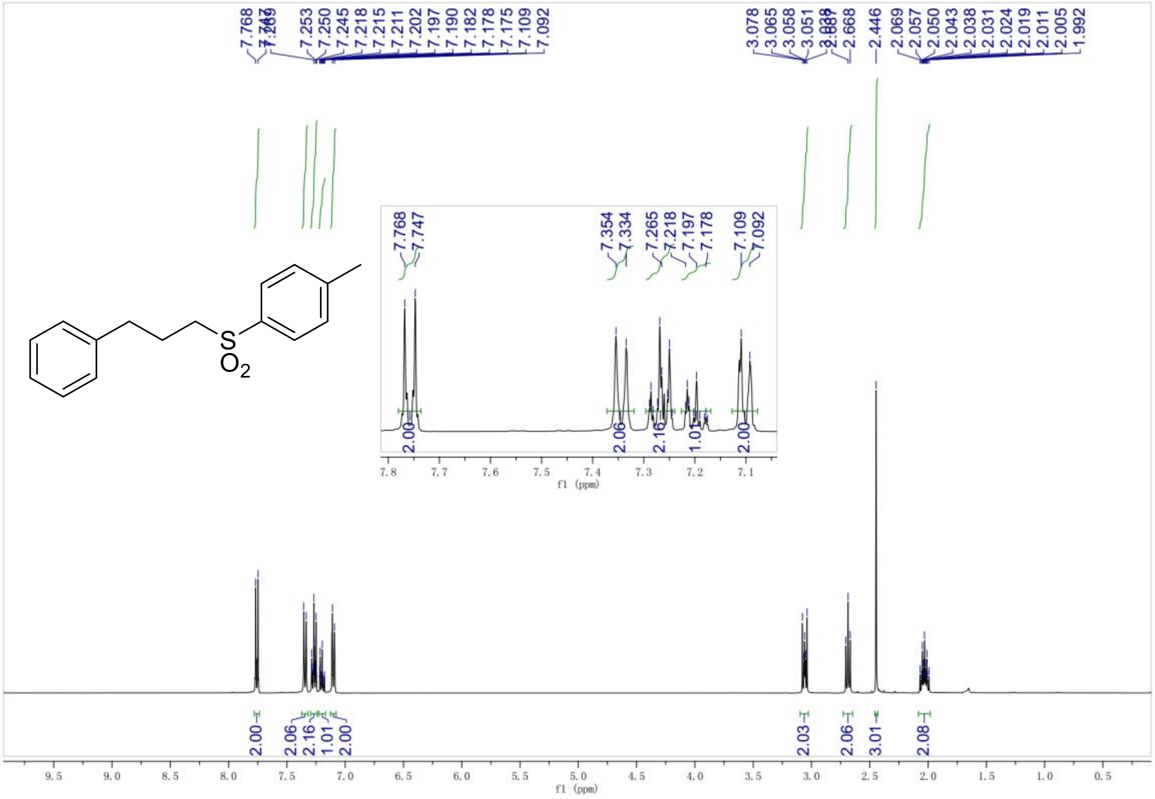

^{1}H NMR spectrum of compound **1a-6** in $CDCl_3$ (400 MHz).

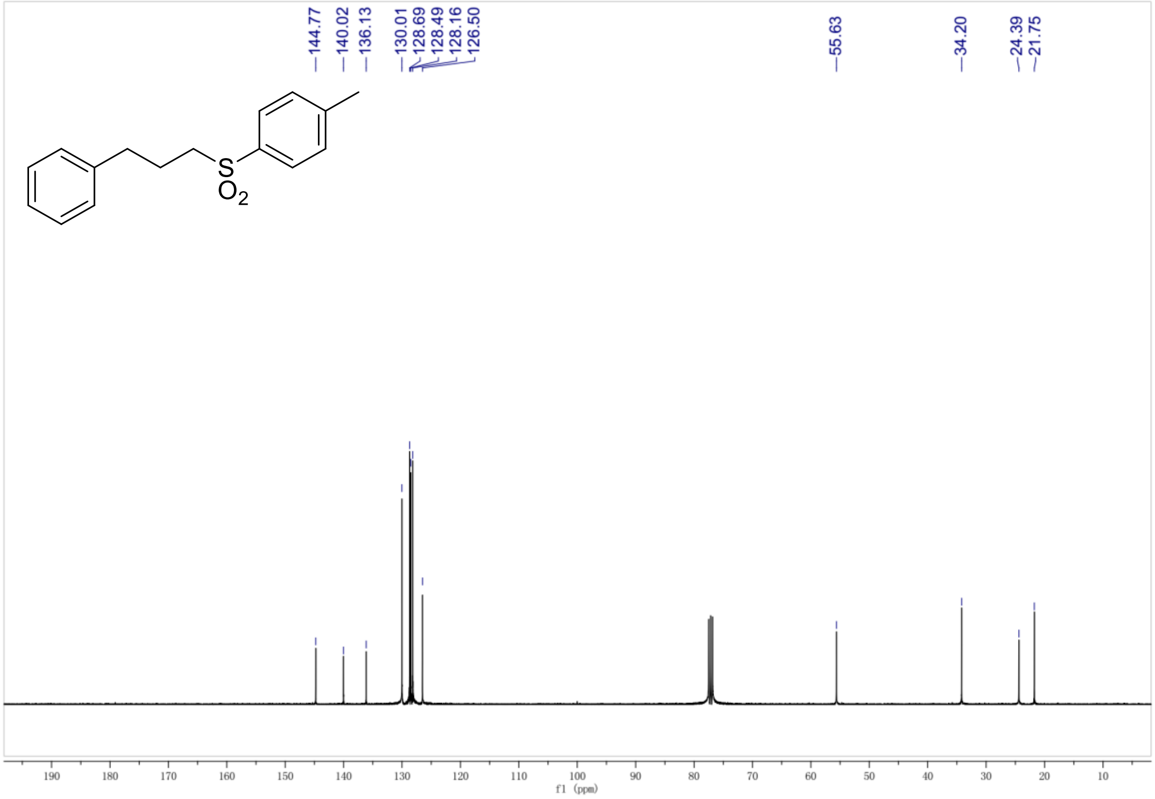

$^{13}C\{^1H\}$ NMR spectrum of compound **1a-6** in $CDCl_3$ (100 MHz).

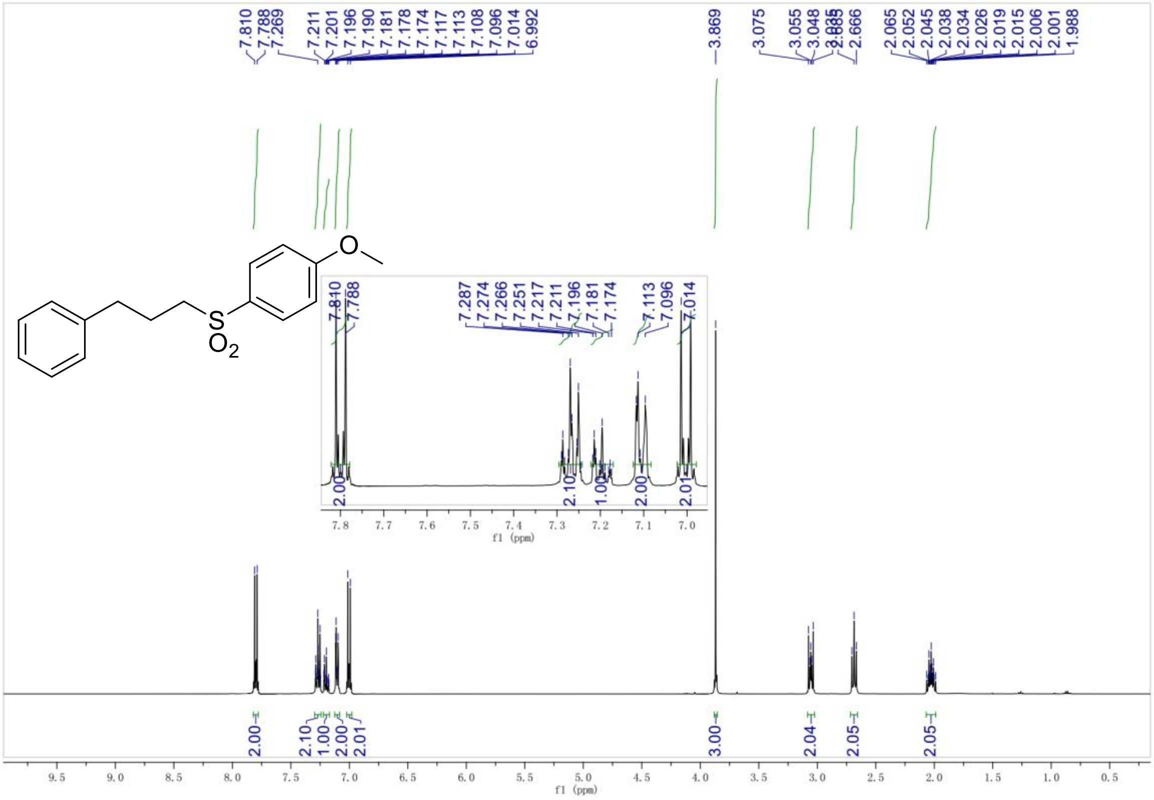

^1H NMR spectrum of compound **1a-7** in $CDCl_3$ (400 MHz).

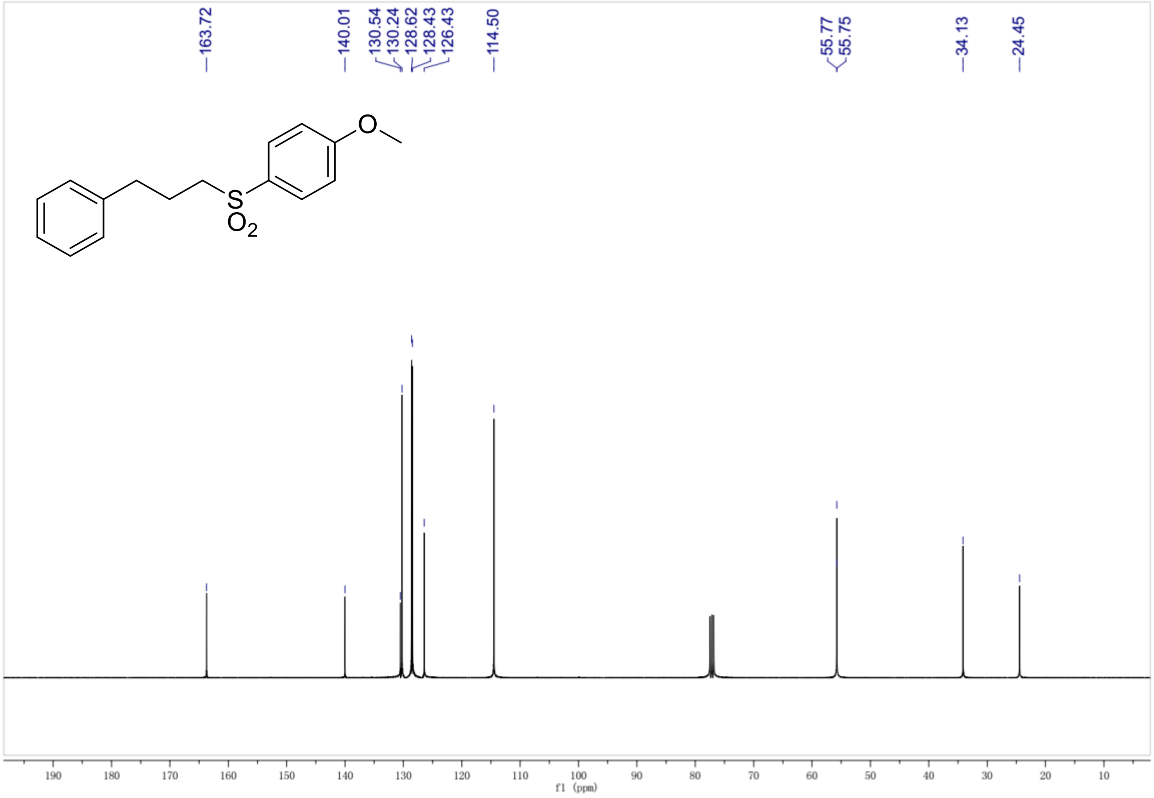

$^{13}C\{^{1}H\}$ NMR spectrum of compound **1a-7** in $CDCl_3$ (100 MHz).

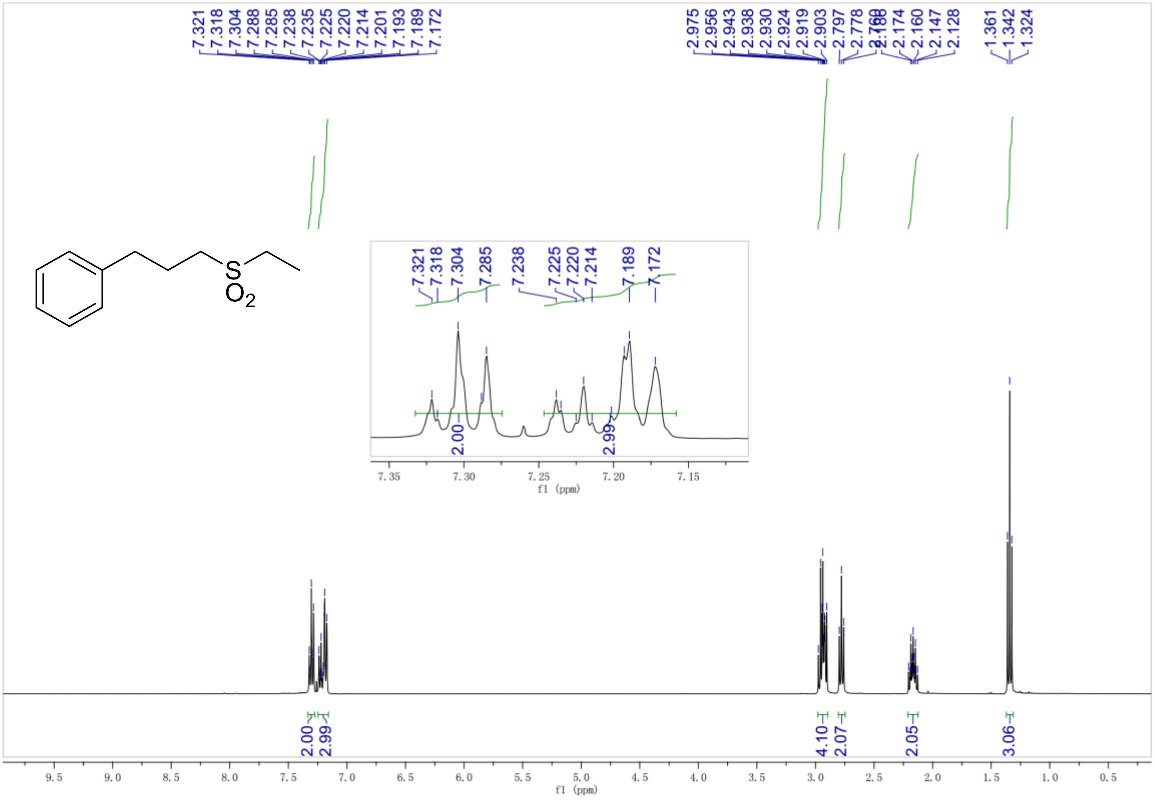

^{1}H NMR spectrum of compound **1a-8** in $CDCl_3$ (400 MHz).

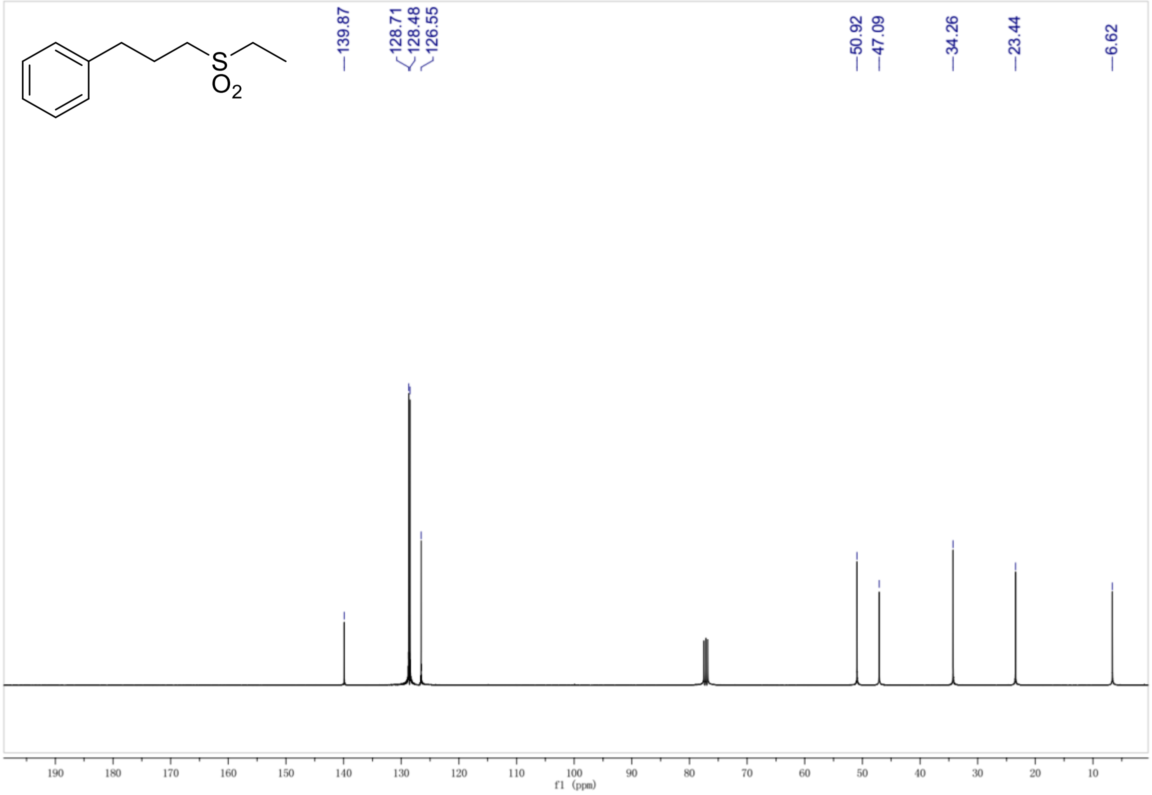

$^{13}C\{^{1}H\}$ NMR spectrum of compound **1a-8** in $CDCl_3$ (100 MHz).

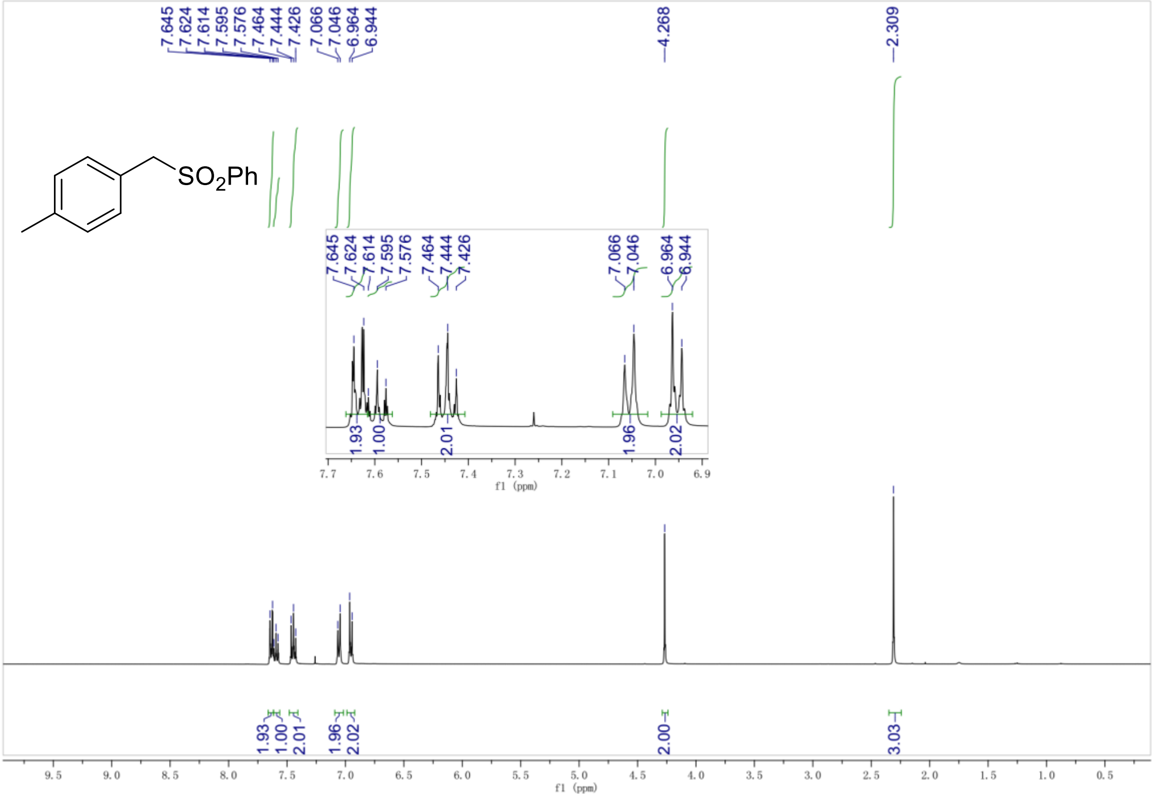

^{1}H NMR spectrum of compound **3a** in $CDCl_3$ (400 MHz).

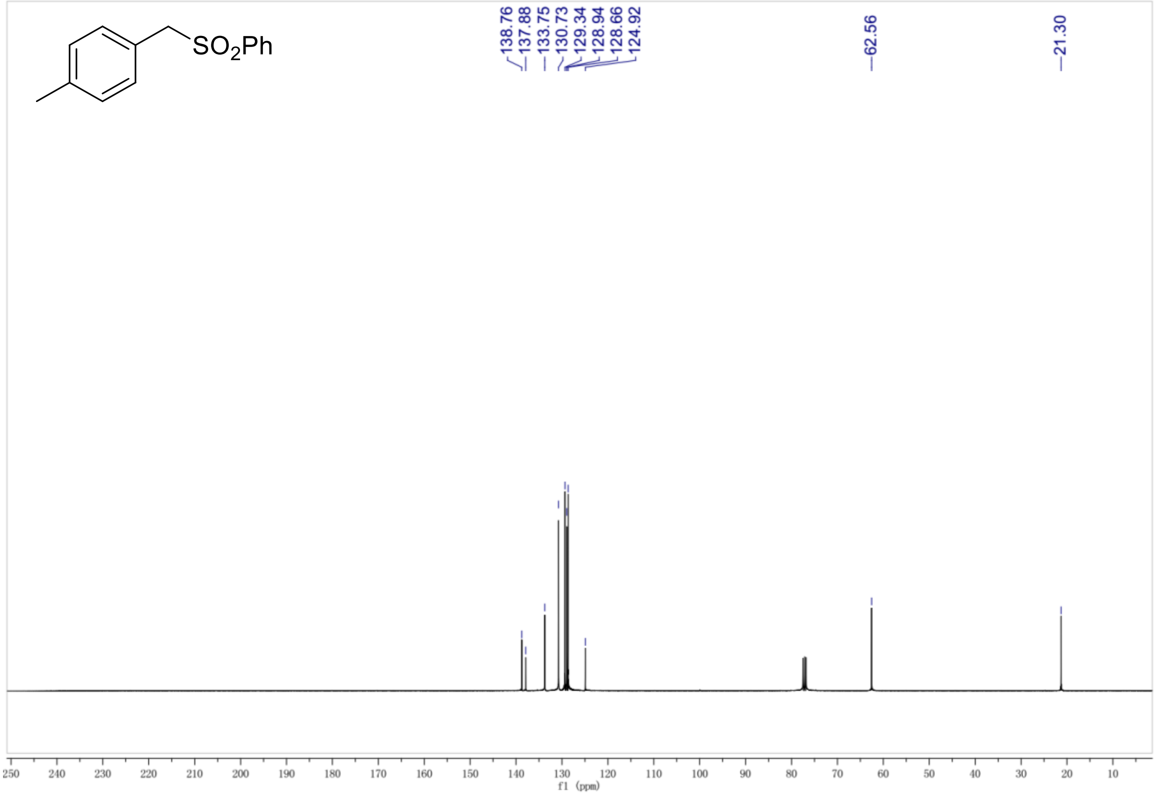

$^{13}C\{^{1}H\}$ NMR spectrum of compound **3a** in $CDCl_3$ (100 MHz).

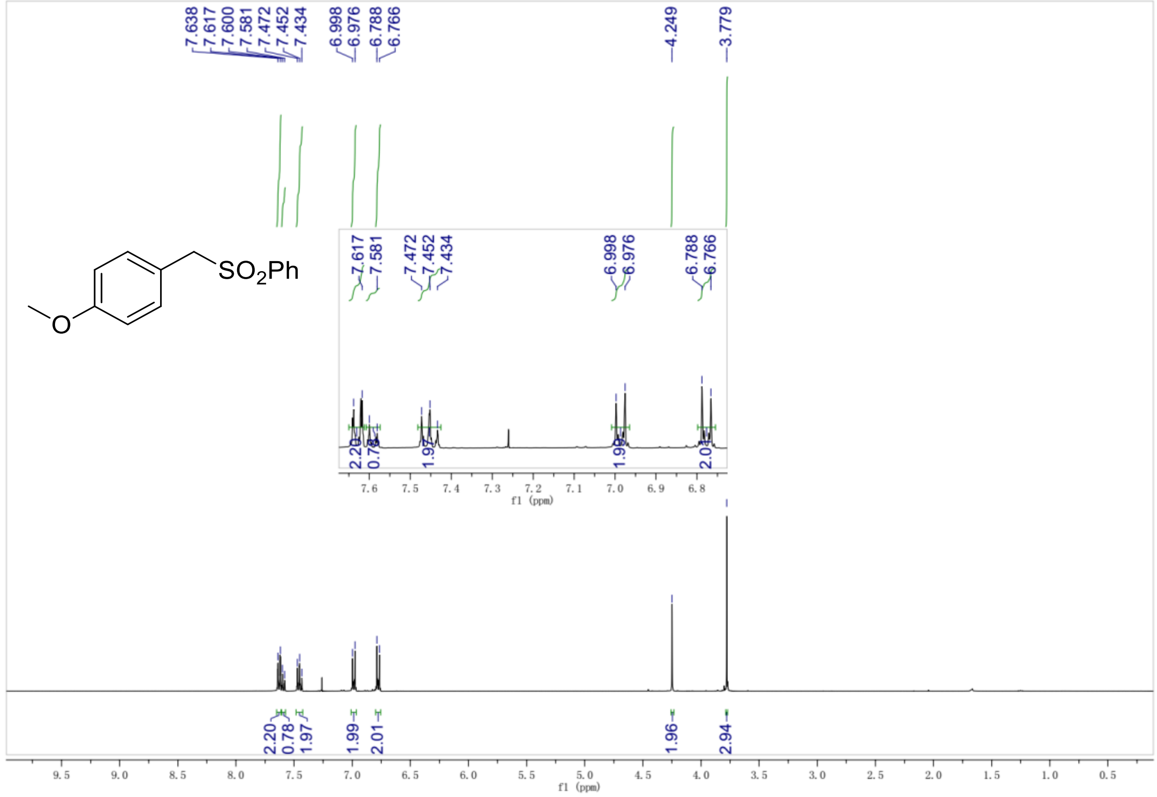

^{1}H NMR spectrum of compound **4a** in $CDCl_3$ (400 MHz).

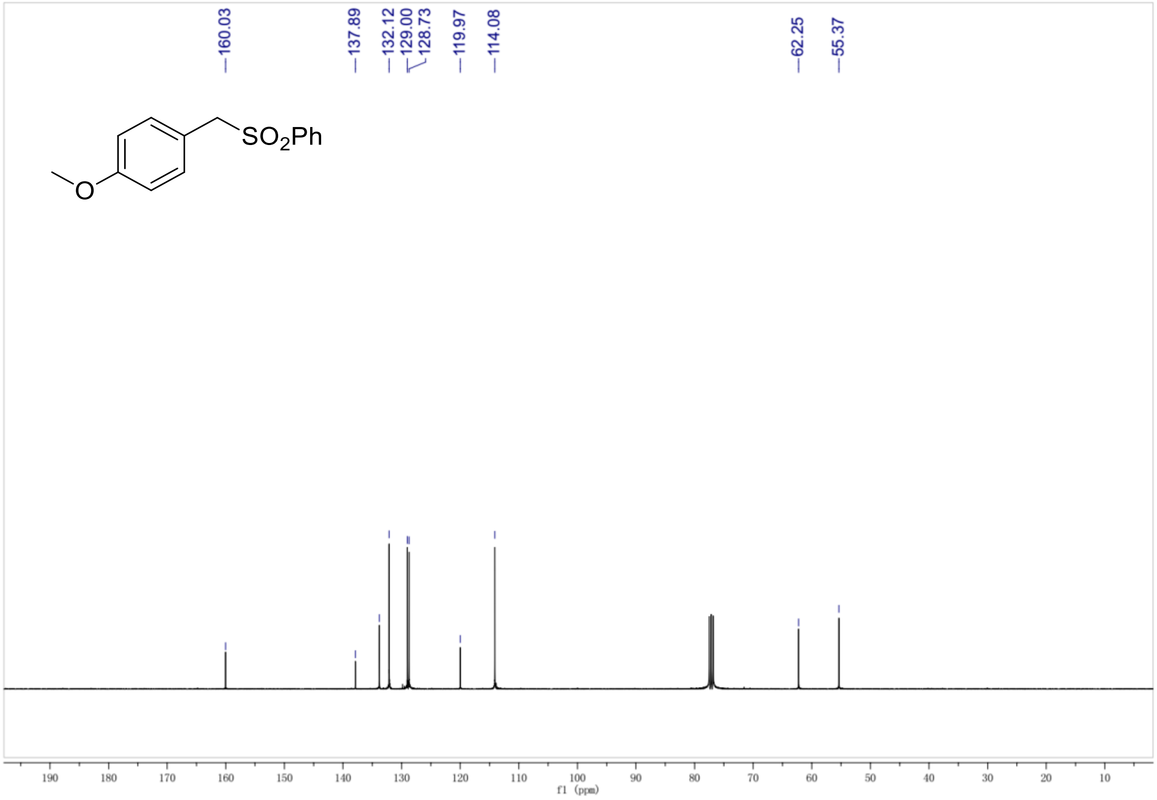

$^{13}C\{^{1}H\}$ NMR spectrum of compound **4a** in $CDCl_3$ (100 MHz).

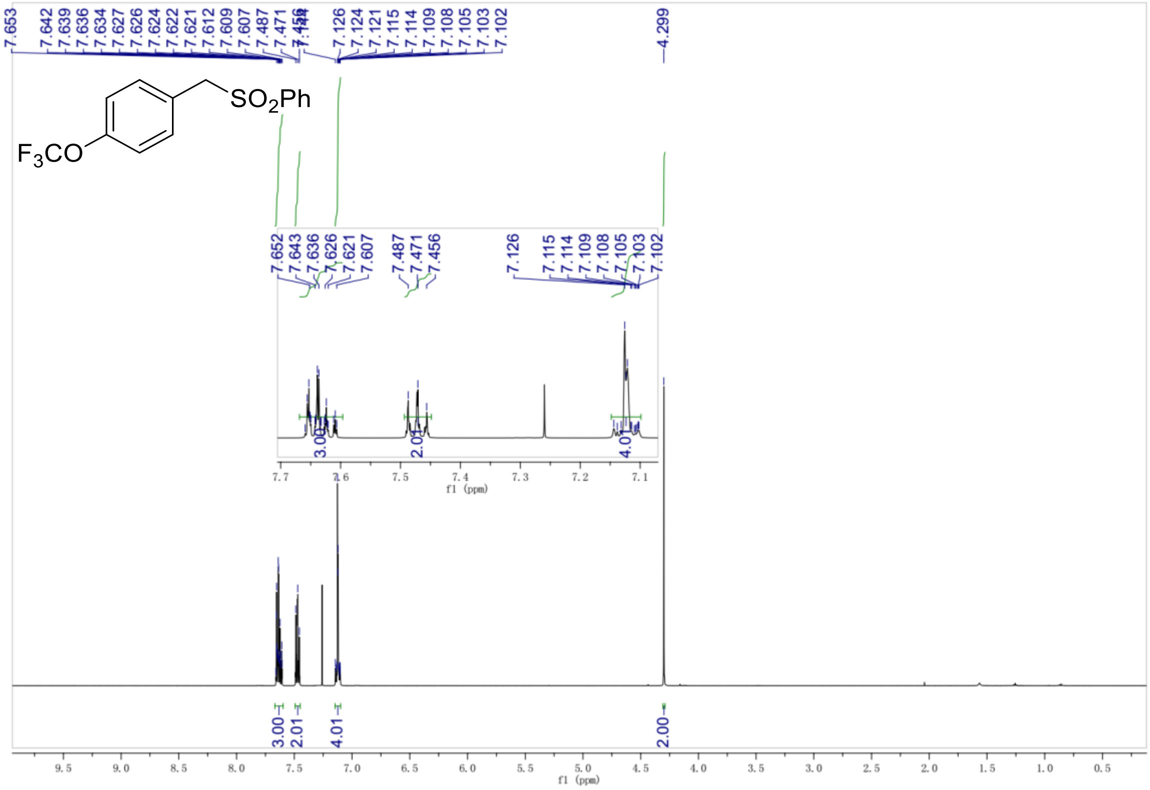

^{1}H NMR spectrum of compound **5a** in $CDCl_3$ (400 MHz).

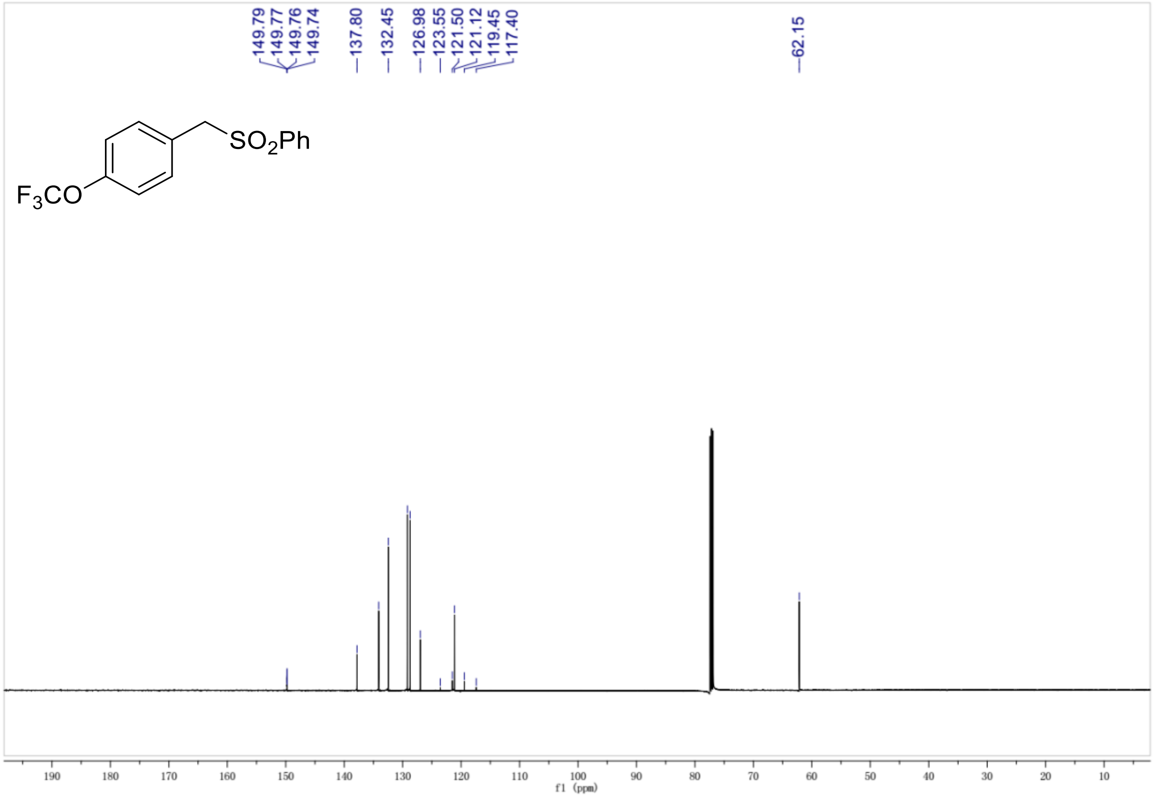

$^{13}C\{^1H\}$ NMR spectrum of compound **5a** in $CDCl_3$ (100 MHz).

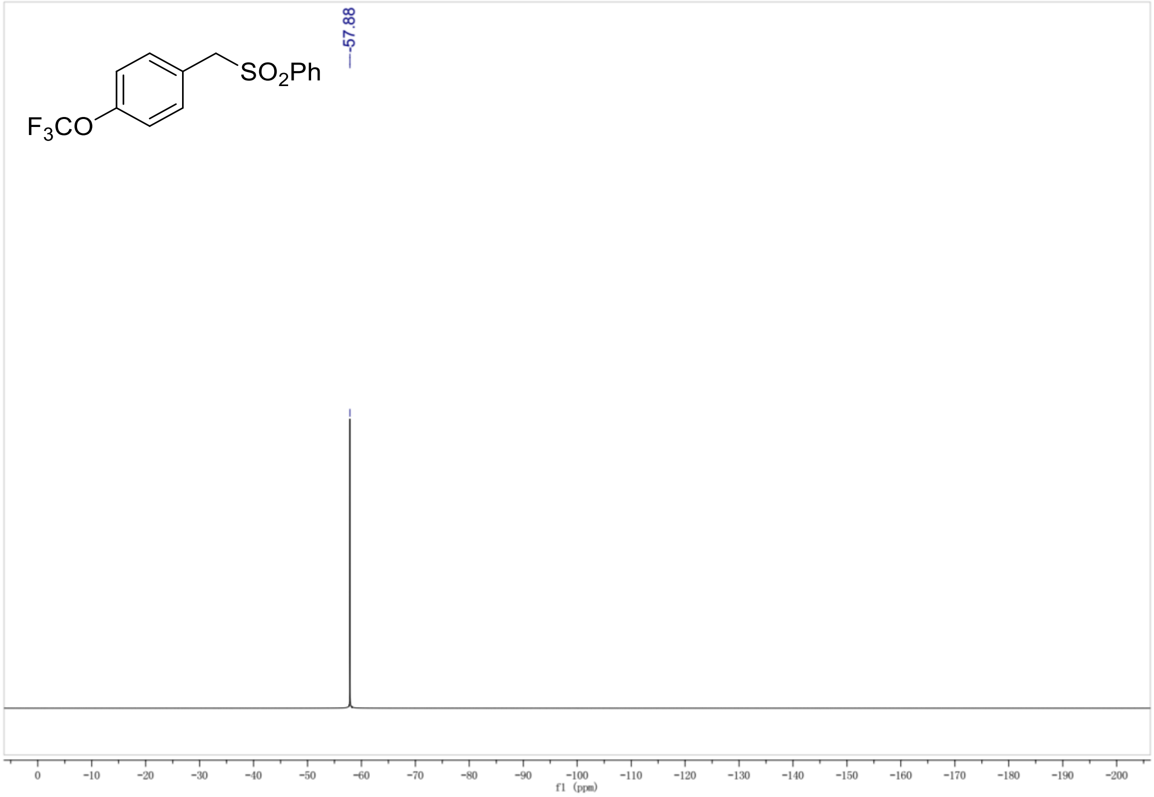

$^{19}F\{^1H\}$ NMR spectrum of compound **5a** in $CDCl_3$ (470 MHz).

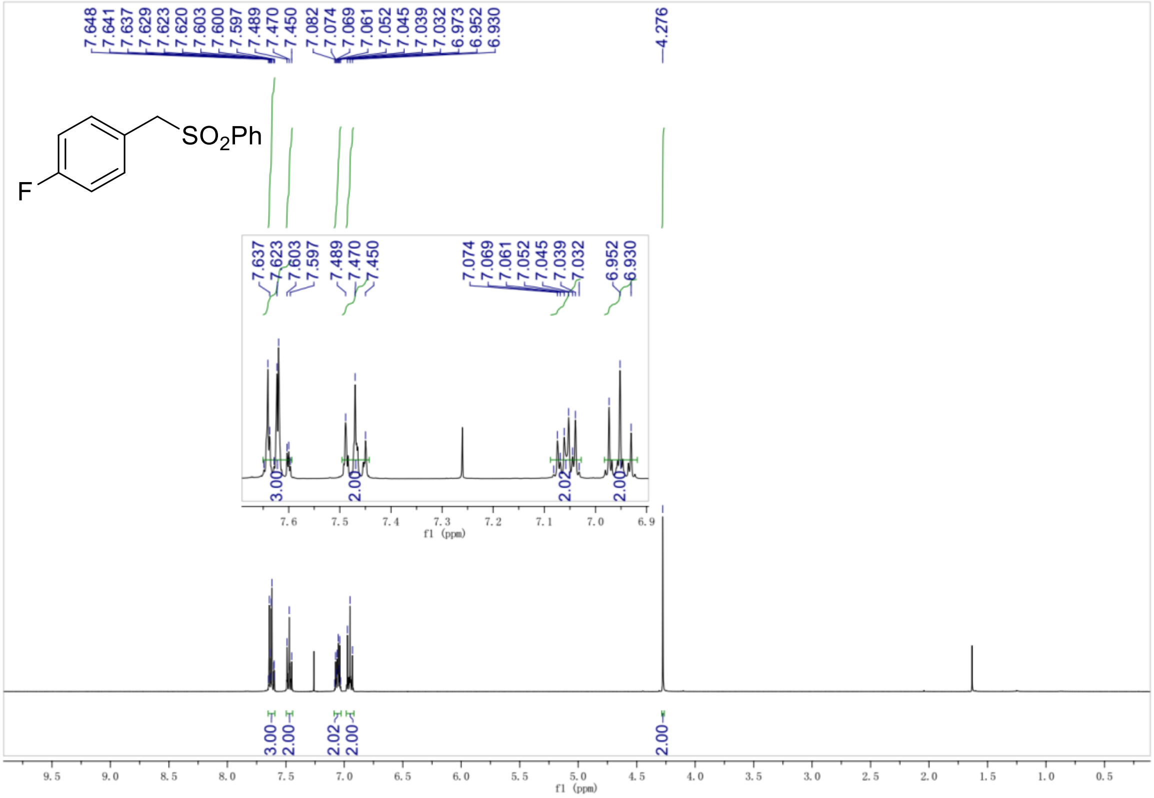

^{1}H NMR spectrum of compound **6a** in $CDCl_3$ (400 MHz).

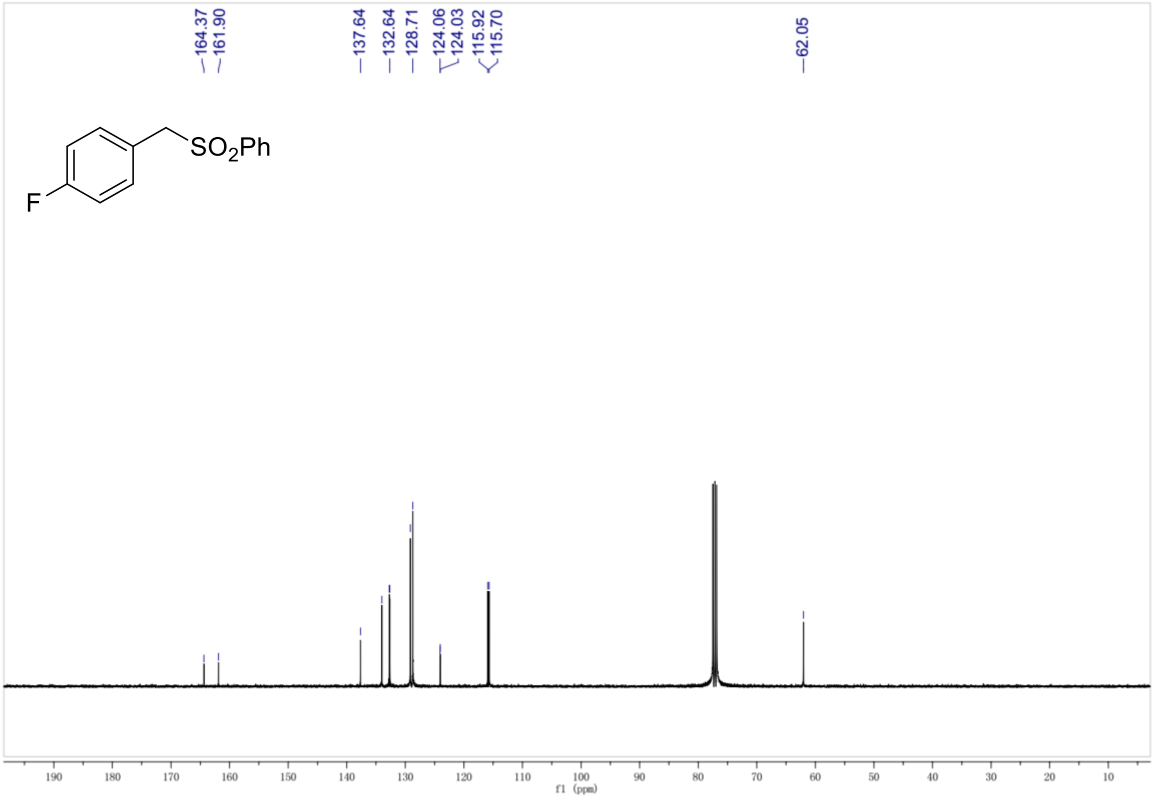

^{13}C{^{1}H} NMR spectrum of compound **6a** in $CDCl_3$ (100 MHz).

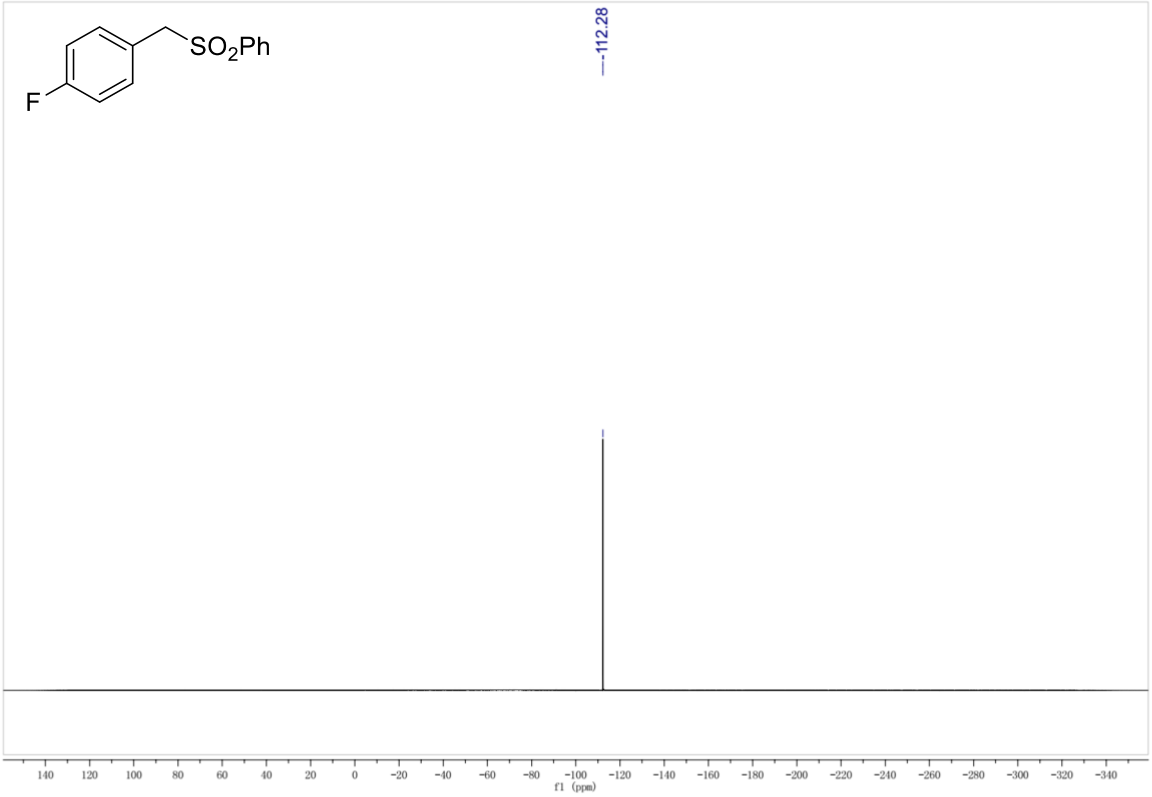

$^{19}F\{^1H\}$ NMR spectrum of compound **6a** in $CDCl_3$ (376 MHz).

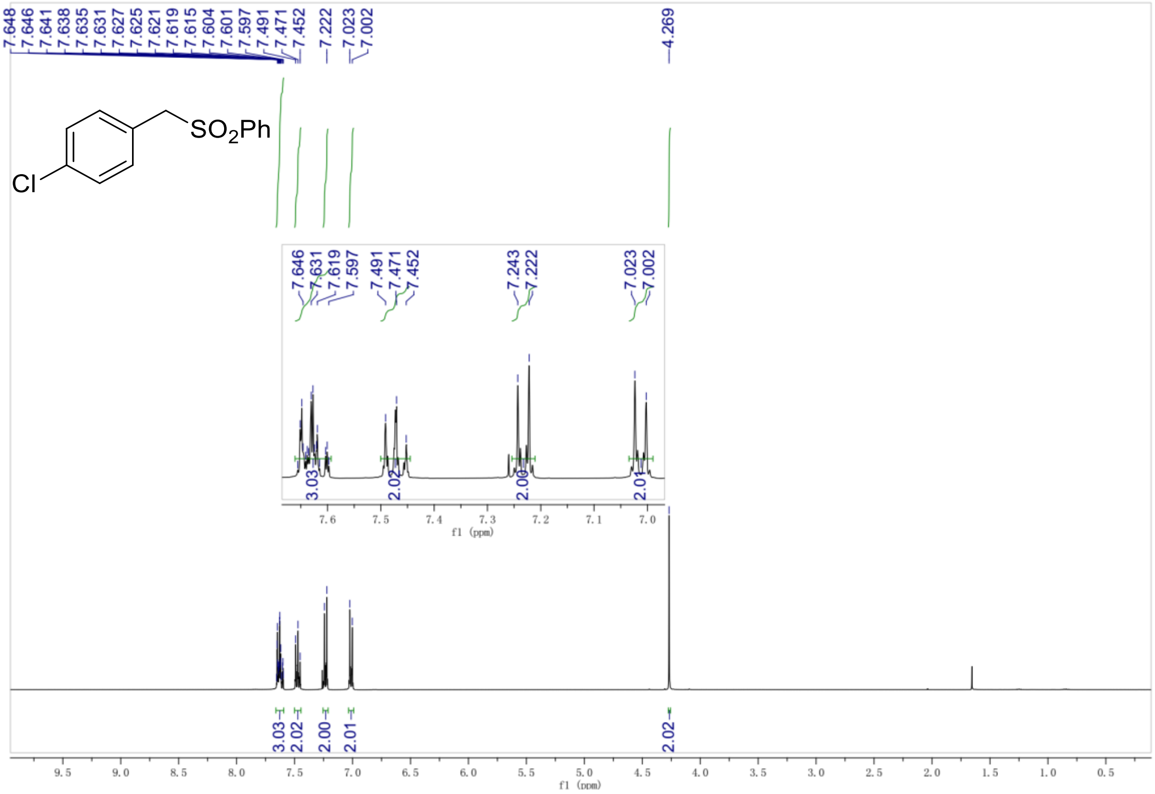

^1H NMR spectrum of compound **7a** in $CDCl_3$ (400 MHz).

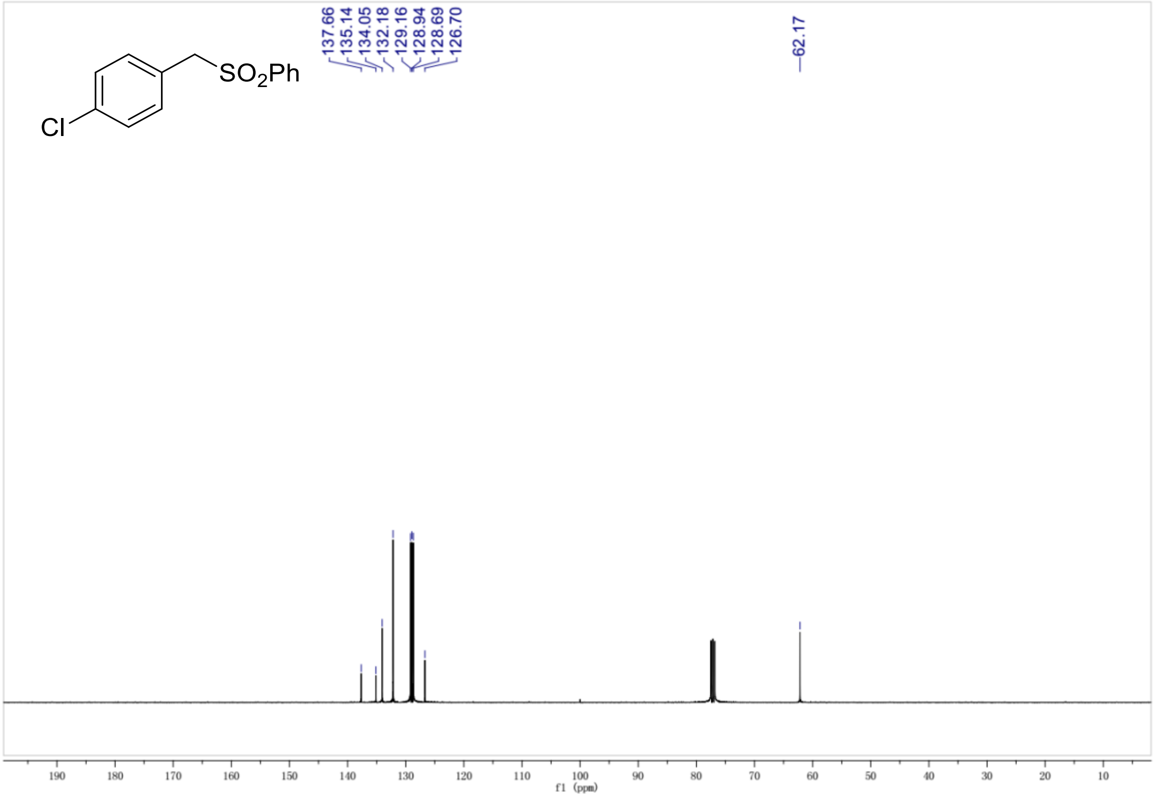

$^{13}C\{^1H\}$ NMR spectrum of compound **7a** in $CDCl_3$ (100 MHz).

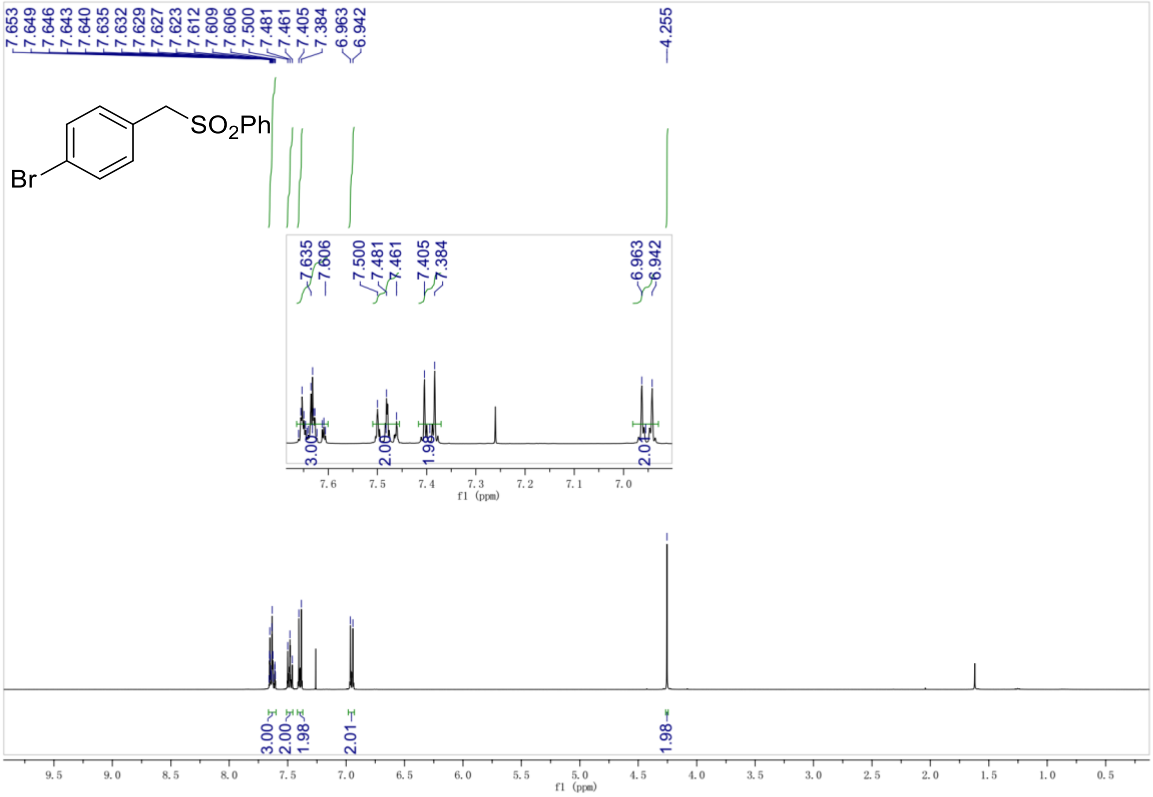

^1H NMR spectrum of compound **8a** in $CDCl_3$ (400 MHz).

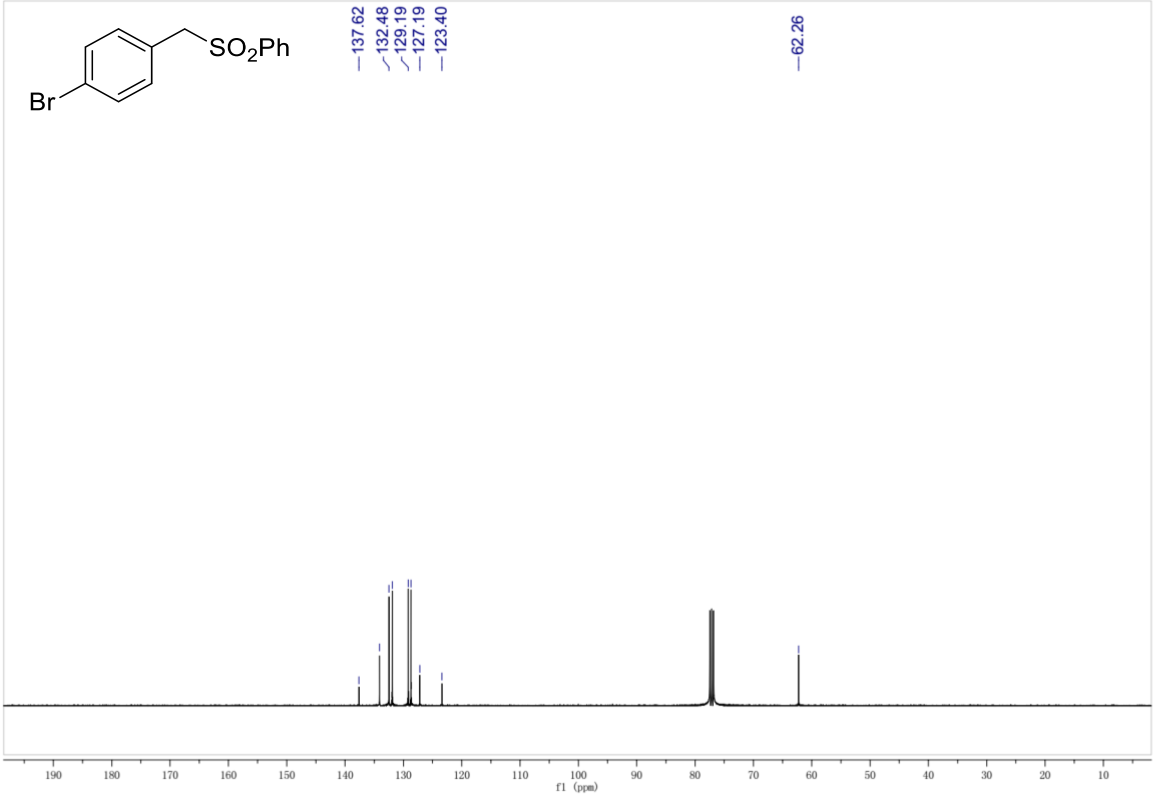

$^{13}C\{^{1}H\}$ NMR spectrum of compound **8a** in $CDCl_3$ (100 MHz).

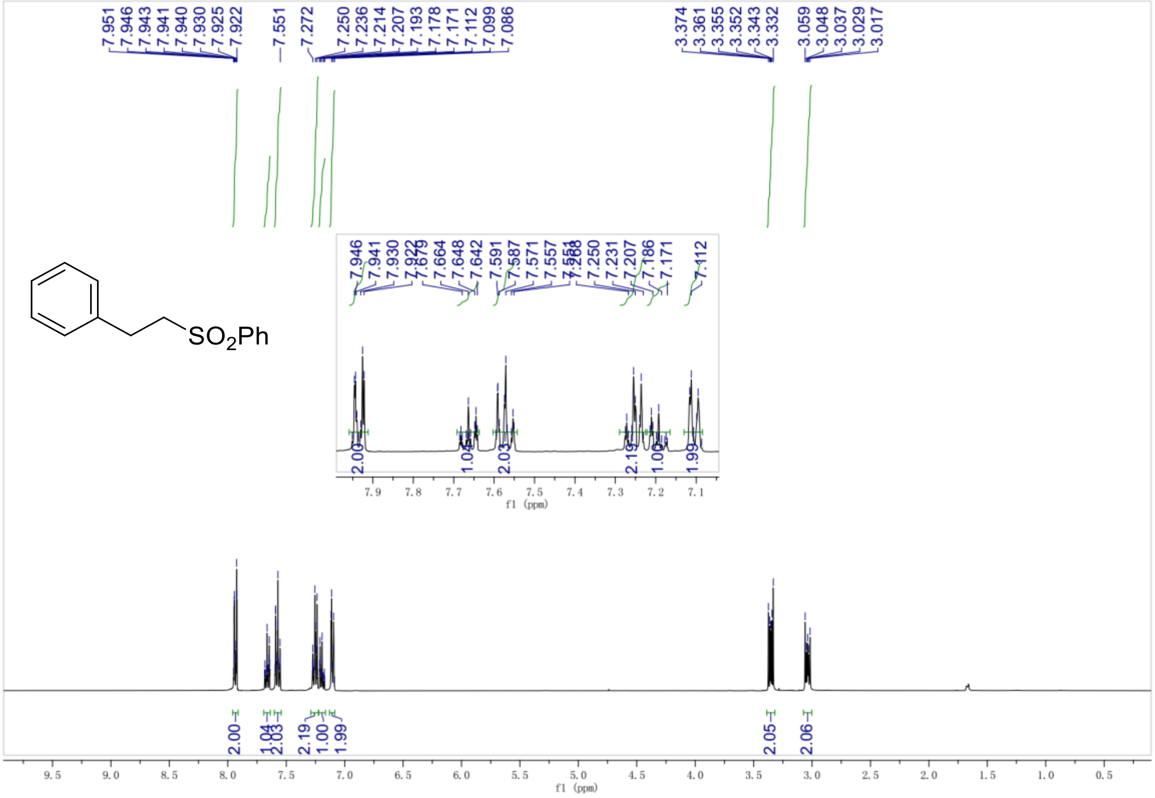

^{1}H NMR spectrum of compound **9a** in $CDCl_3$ (400 MHz).

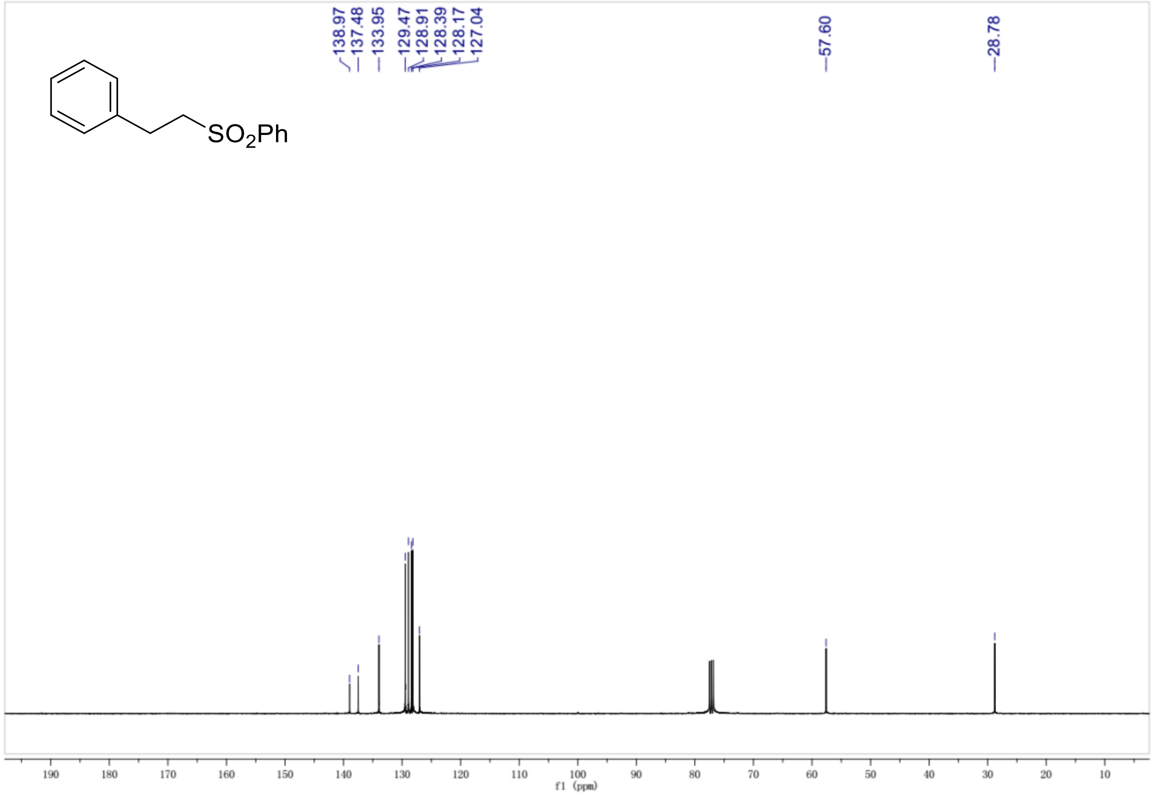

$^{13}C\{^{1}H\}$ NMR spectrum of compound **9a** in $CDCl_3$ (100 MHz).

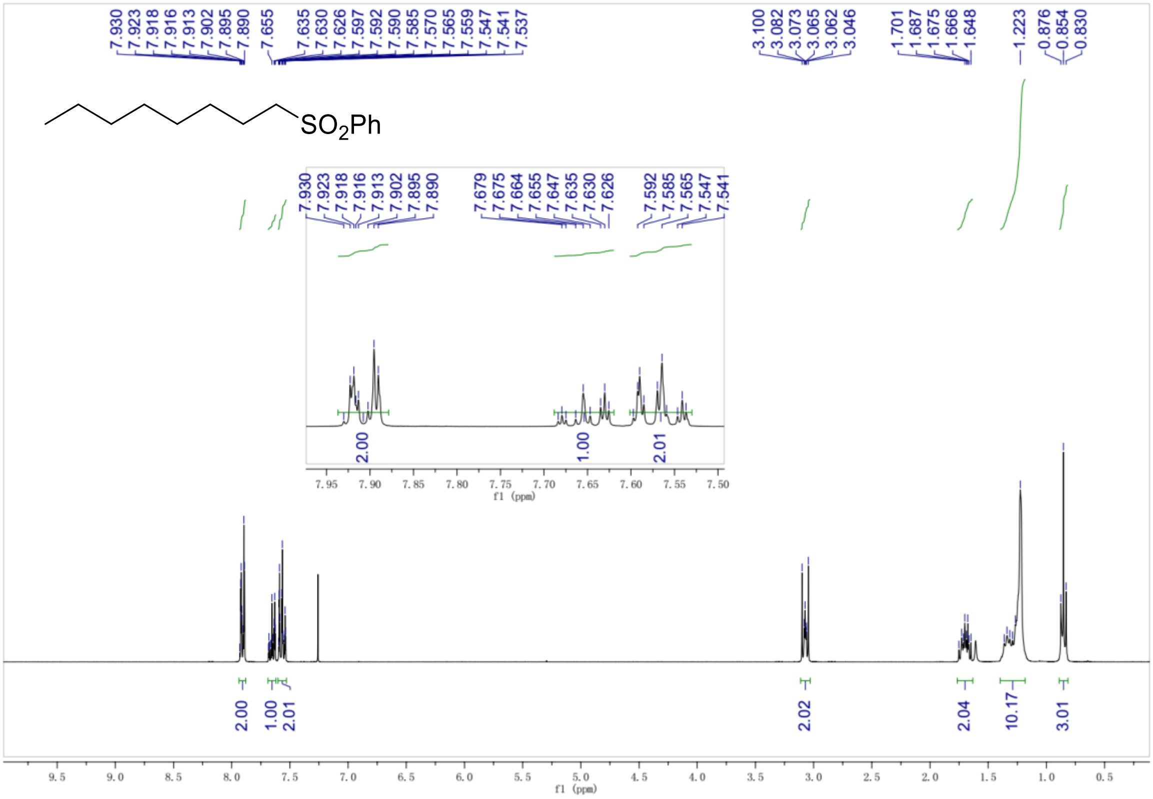

^{1}H NMR spectrum of compound **10a** in $CDCl_3$ (300 MHz).

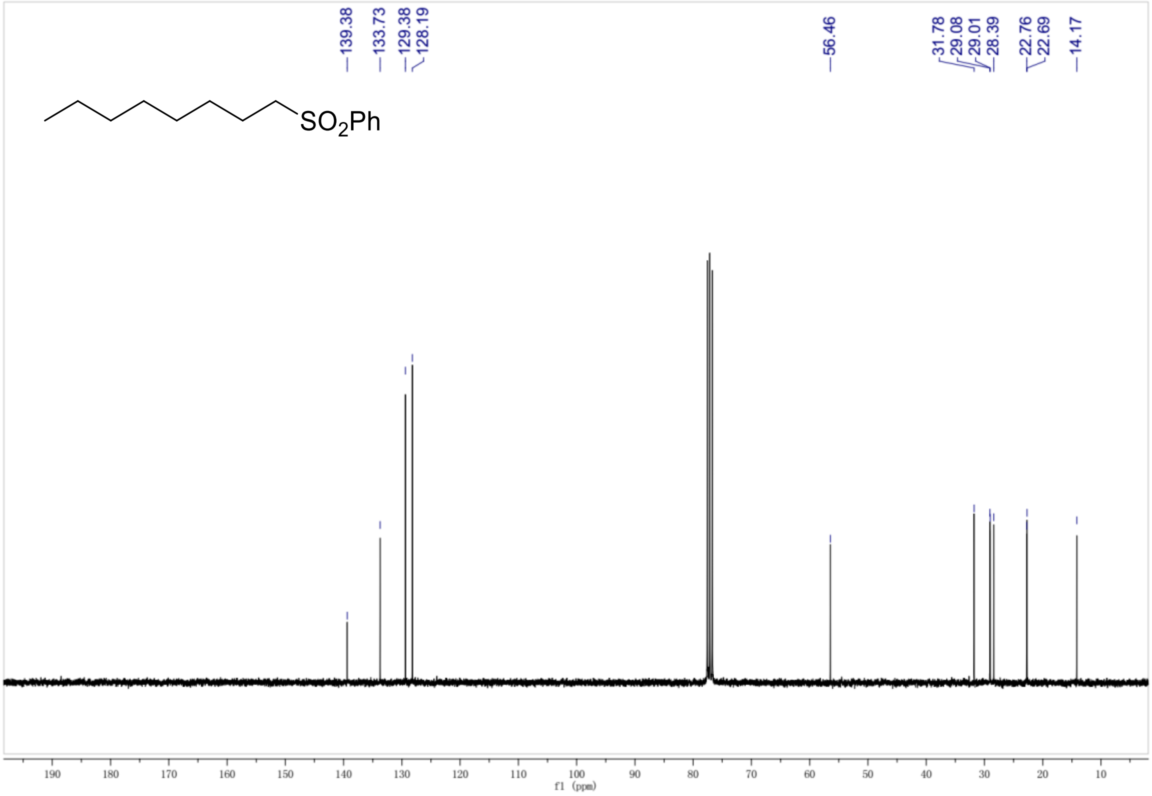

$^{13}C\{^{1}H\}$ NMR spectrum of compound **10a** in $CDCl_3$ (75 MHz).

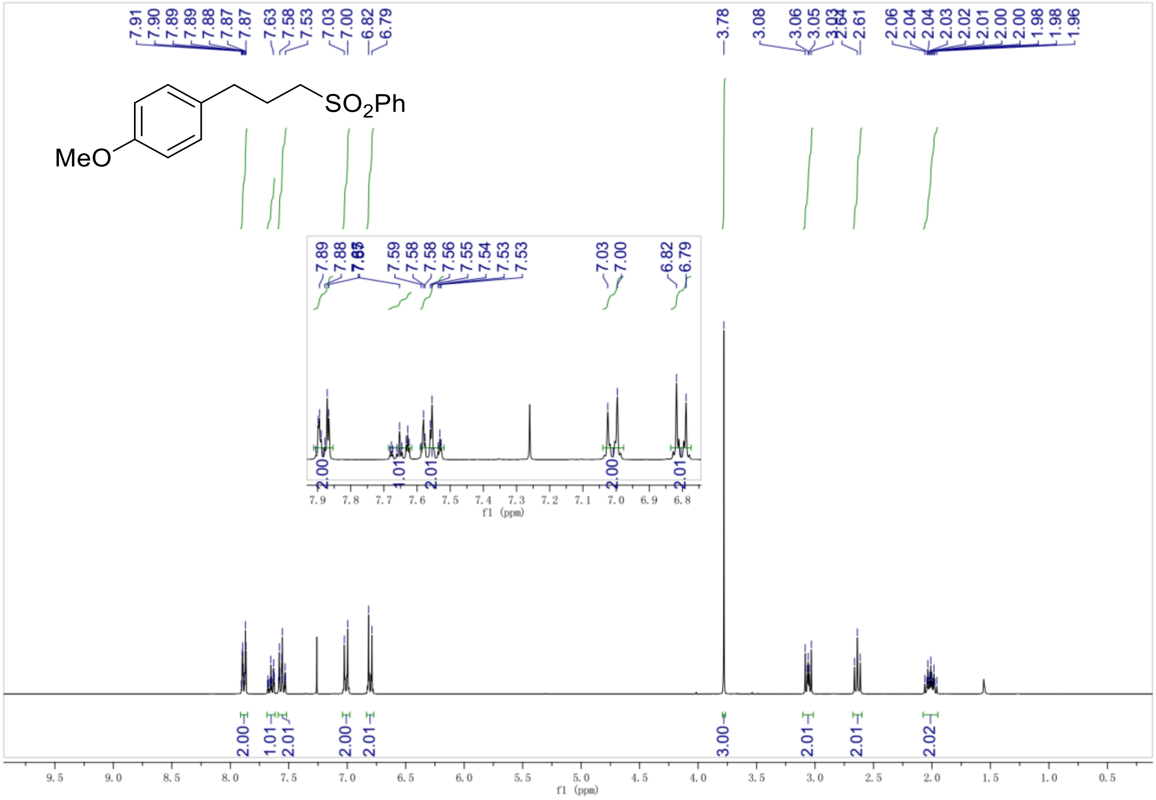

^{1}H NMR spectrum of compound **11a** in $CDCl_3$ (300 MHz).

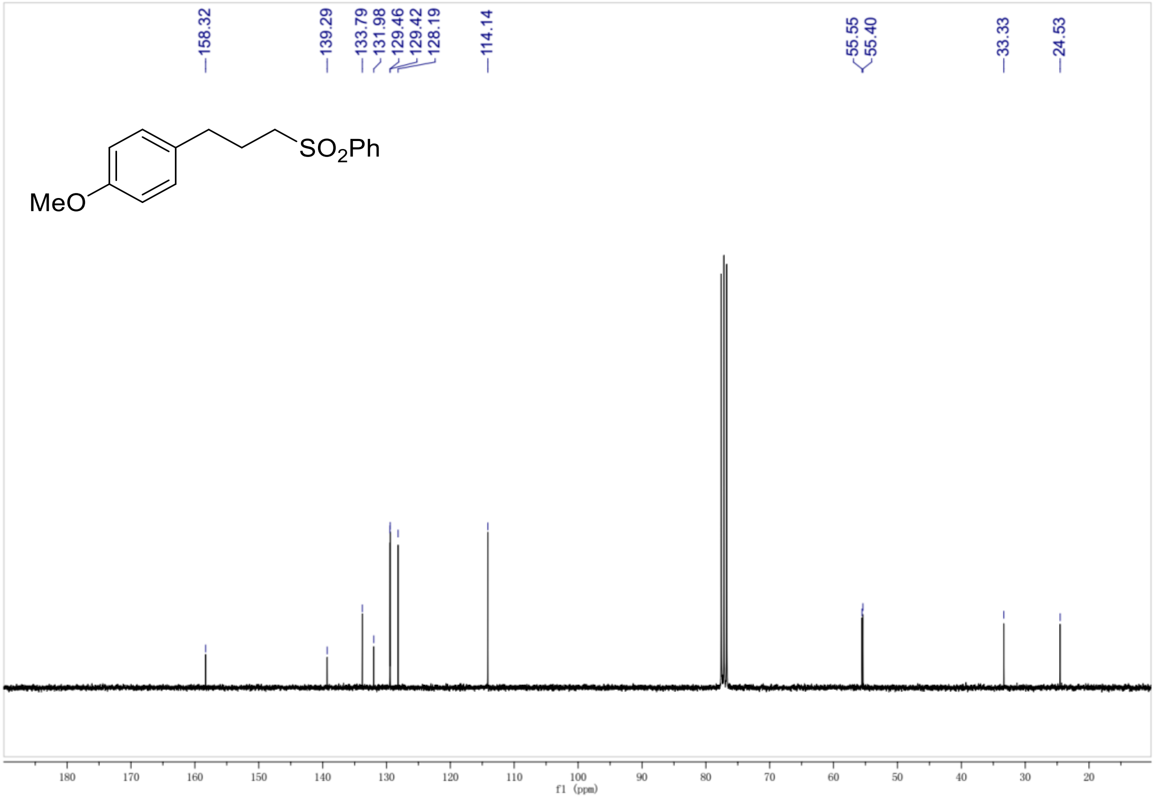

$^{13}C\{^{1}H\}$ NMR spectrum of compound **11a** in $CDCl_3$ (75 MHz).

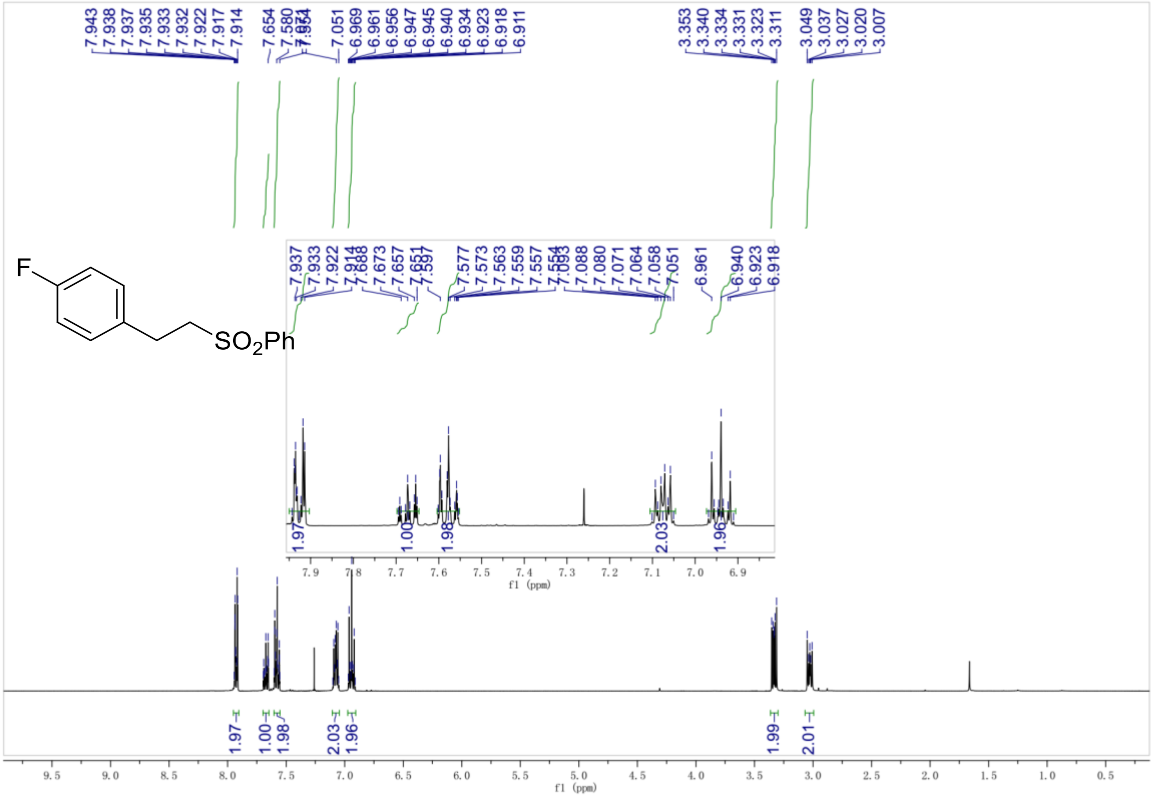

^{1}H NMR spectrum of compound **12a** in $CDCl_3$ (400 MHz).

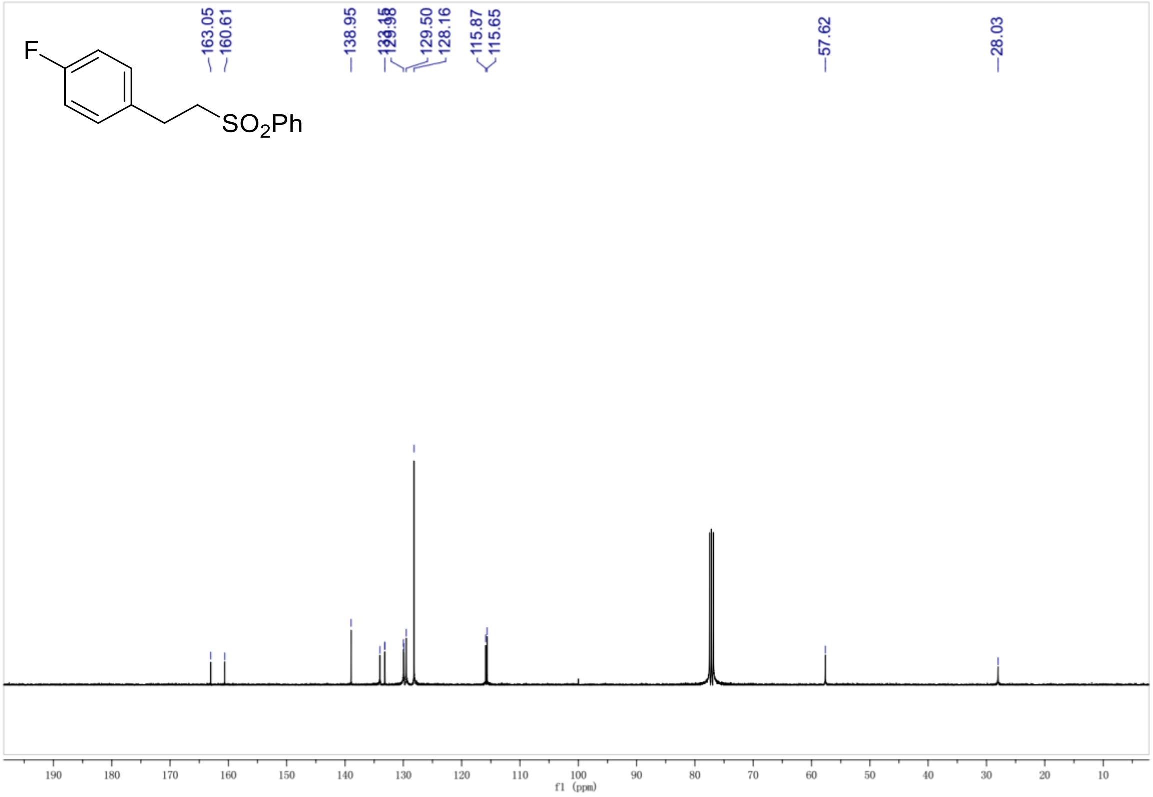

$^{13}C\{^1H\}$ NMR spectrum of compound **12a** in $CDCl_3$ (100 MHz).

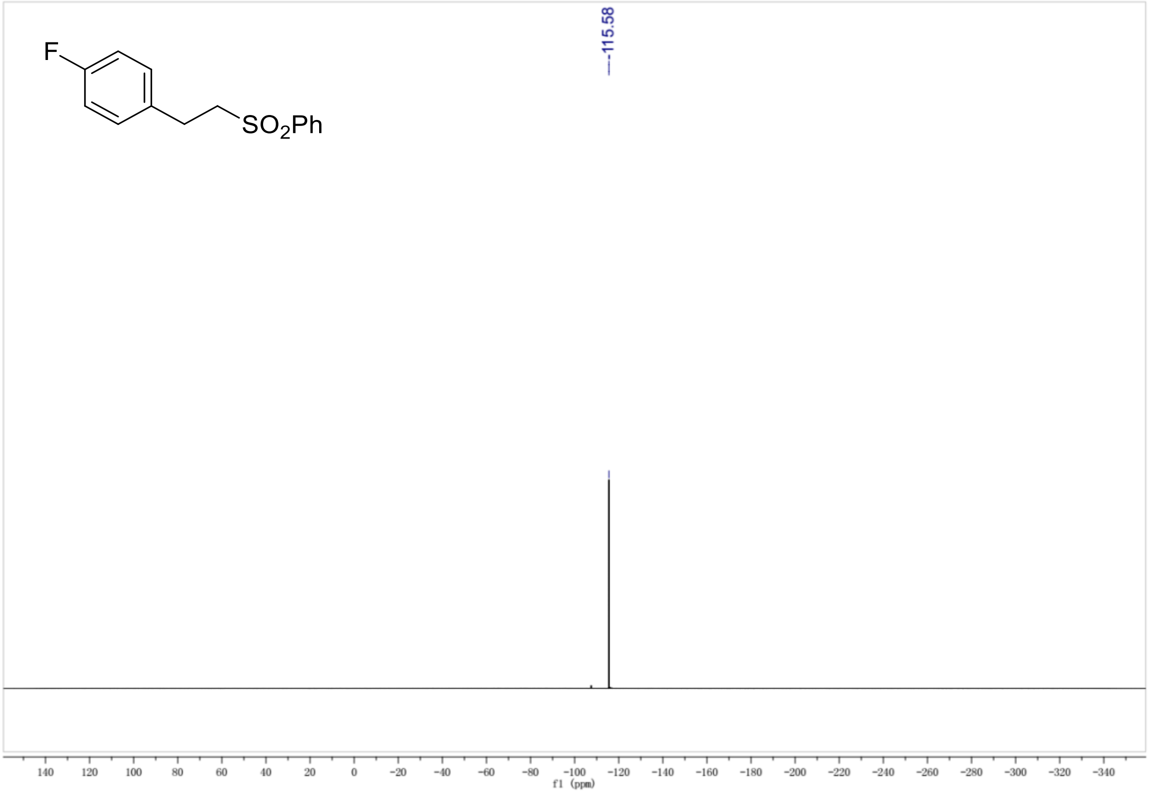

$^{19}F\{^1H\}$ NMR spectrum of compound **12a** in $CDCl_3$ (376 MHz).

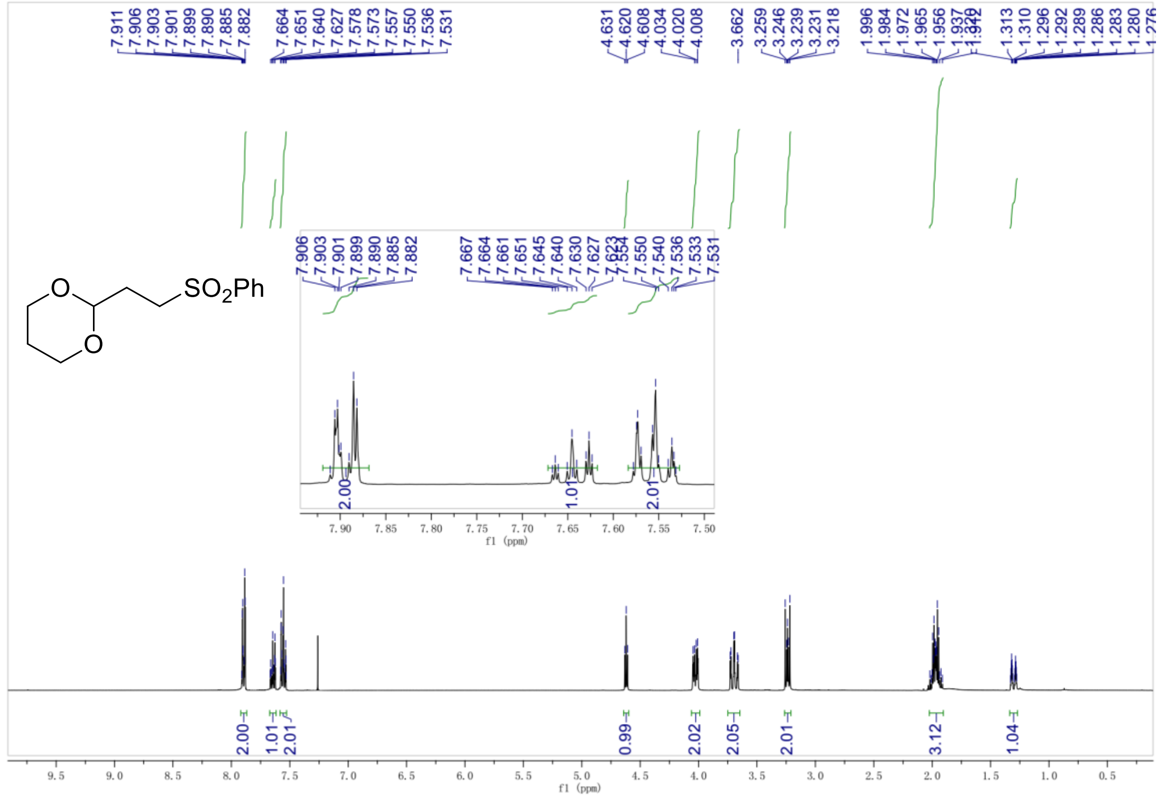

^{1}H NMR spectrum of compound **14a** in $CDCl_3$ (400 MHz).

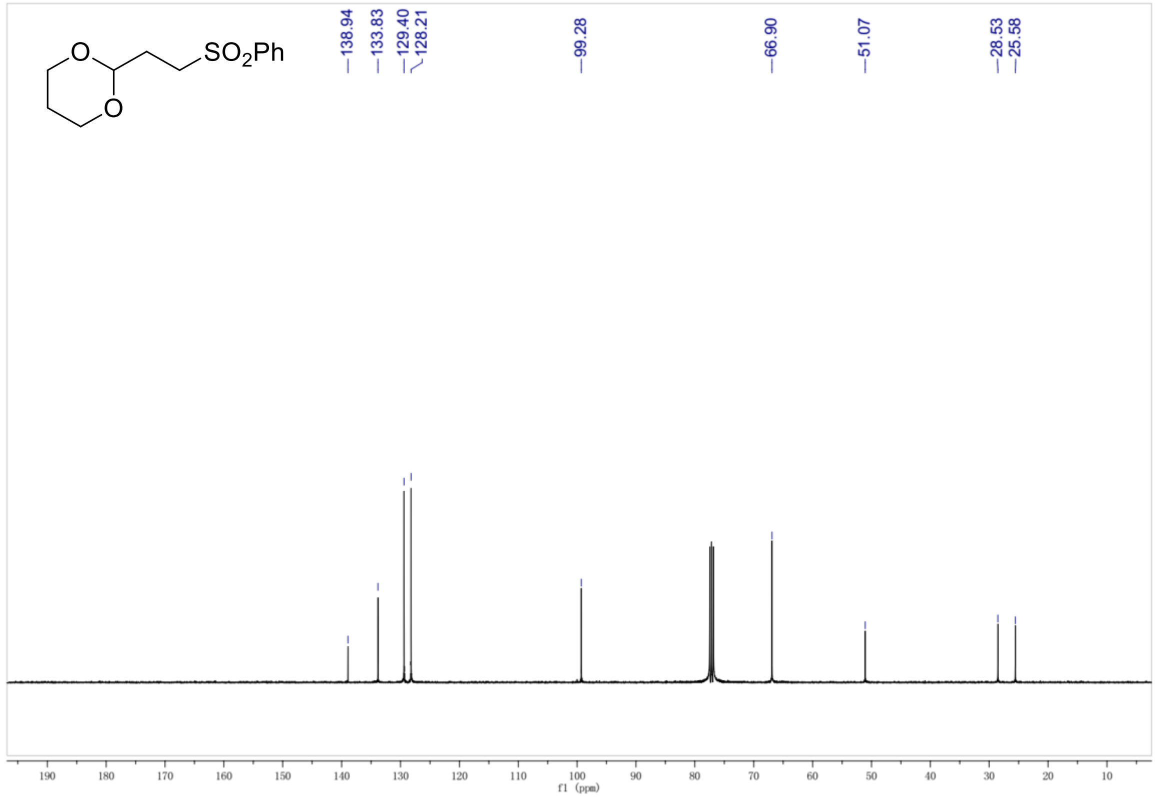

$^{13}C\{^{1}H\}$ NMR spectrum of compound **14a** in $CDCl_3$ (100 MHz).

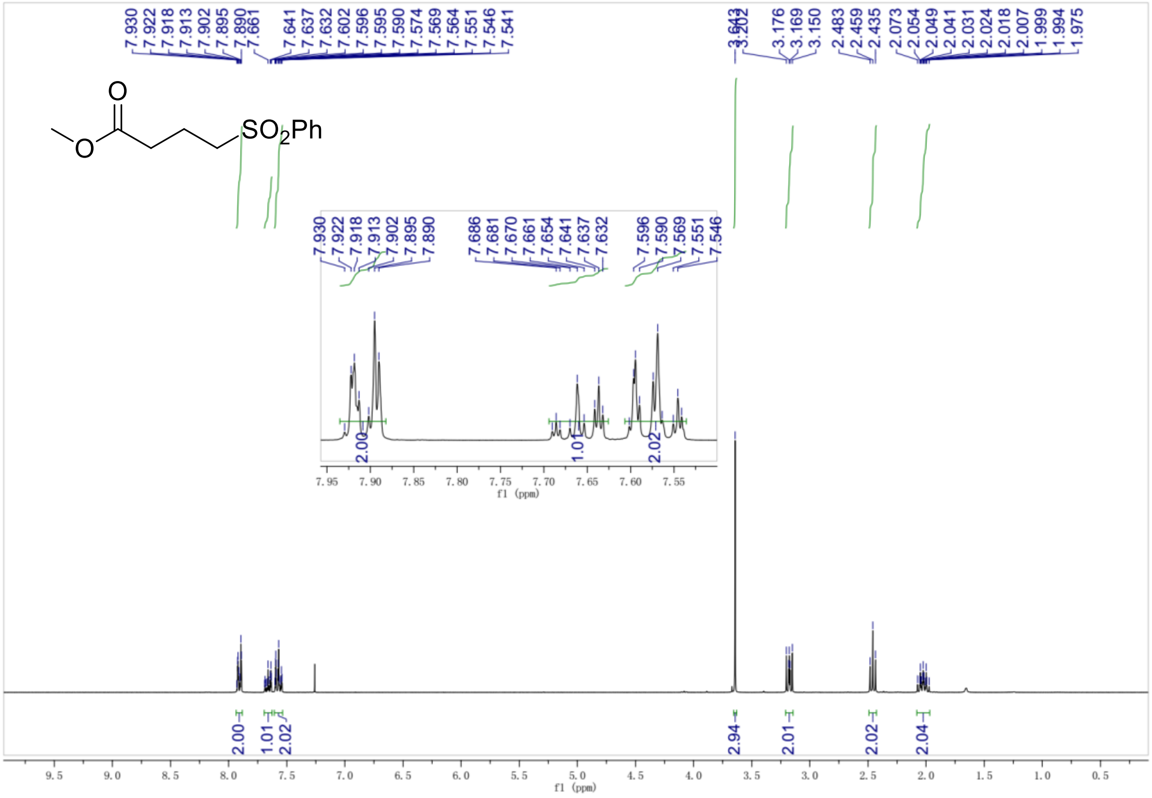

^{1}H NMR spectrum of compound **15a** in $CDCl_3$ (300 MHz).

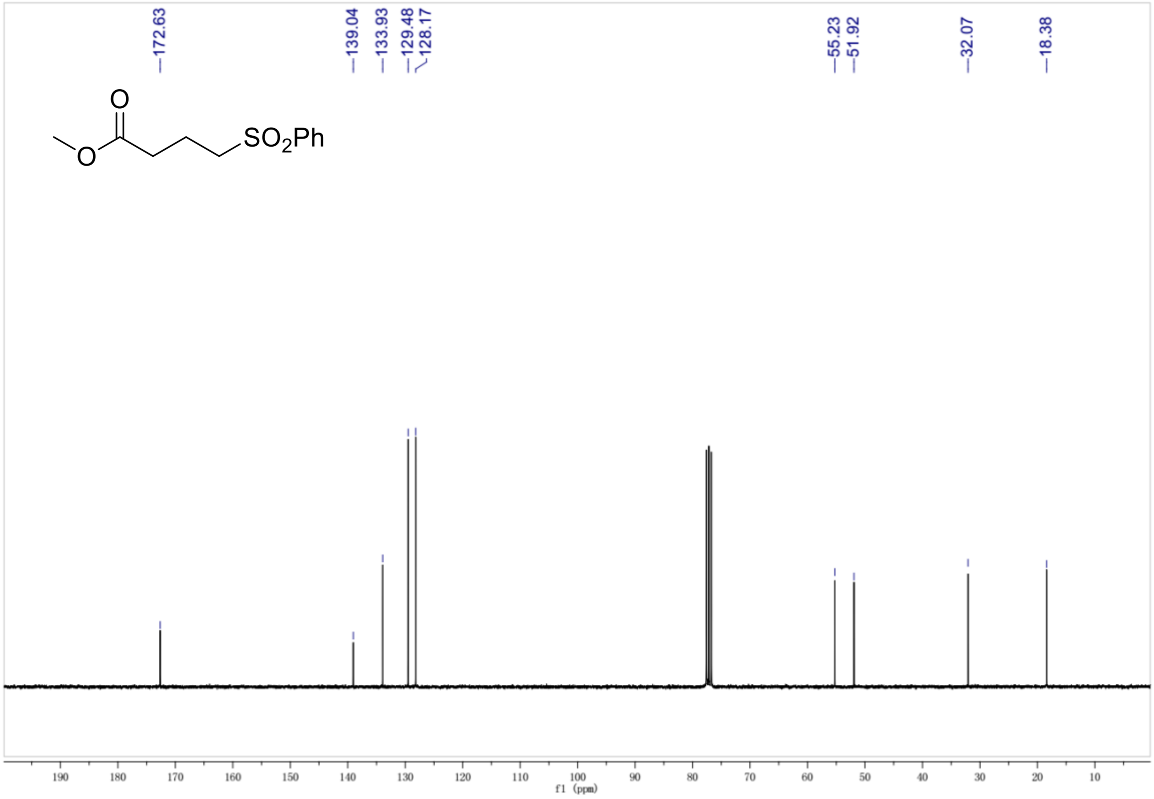

$^{13}C\{^{1}H\}$ NMR spectrum of compound **15a** in $CDCl_3$ (75 MHz).

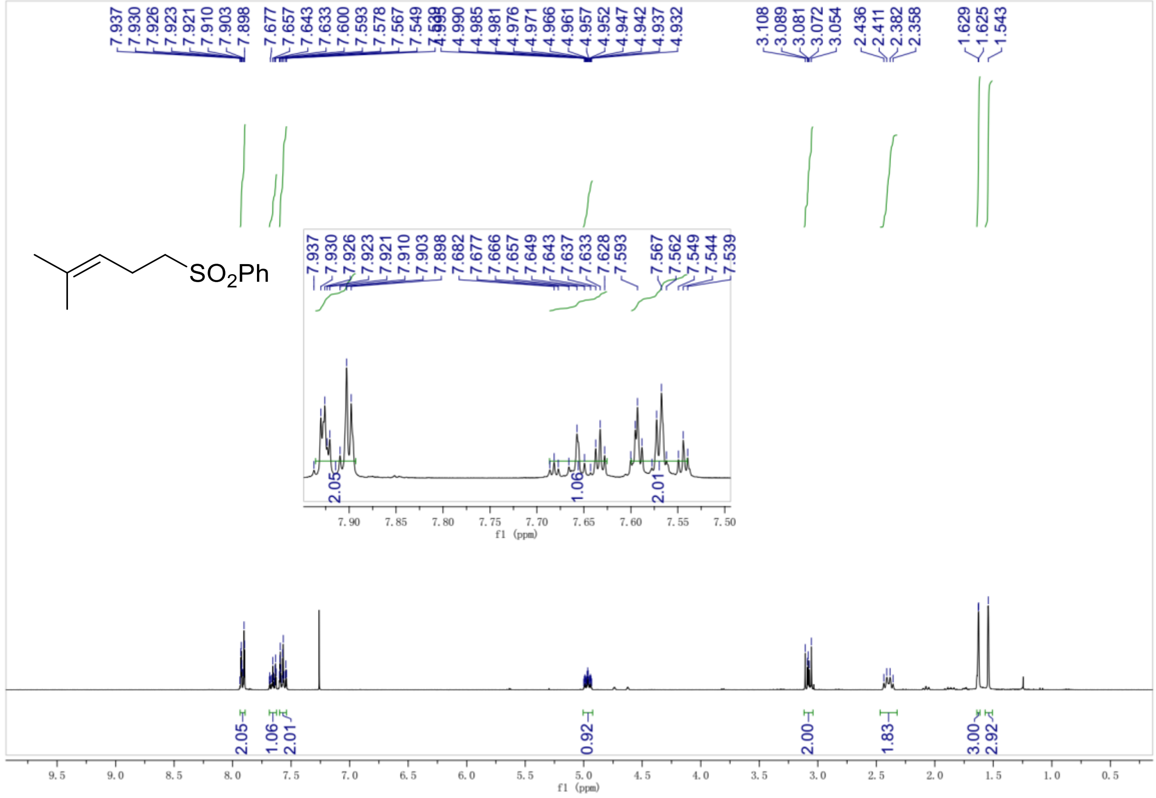

^{1}H NMR spectrum of compound **16a** in $CDCl_3$ (300 MHz).

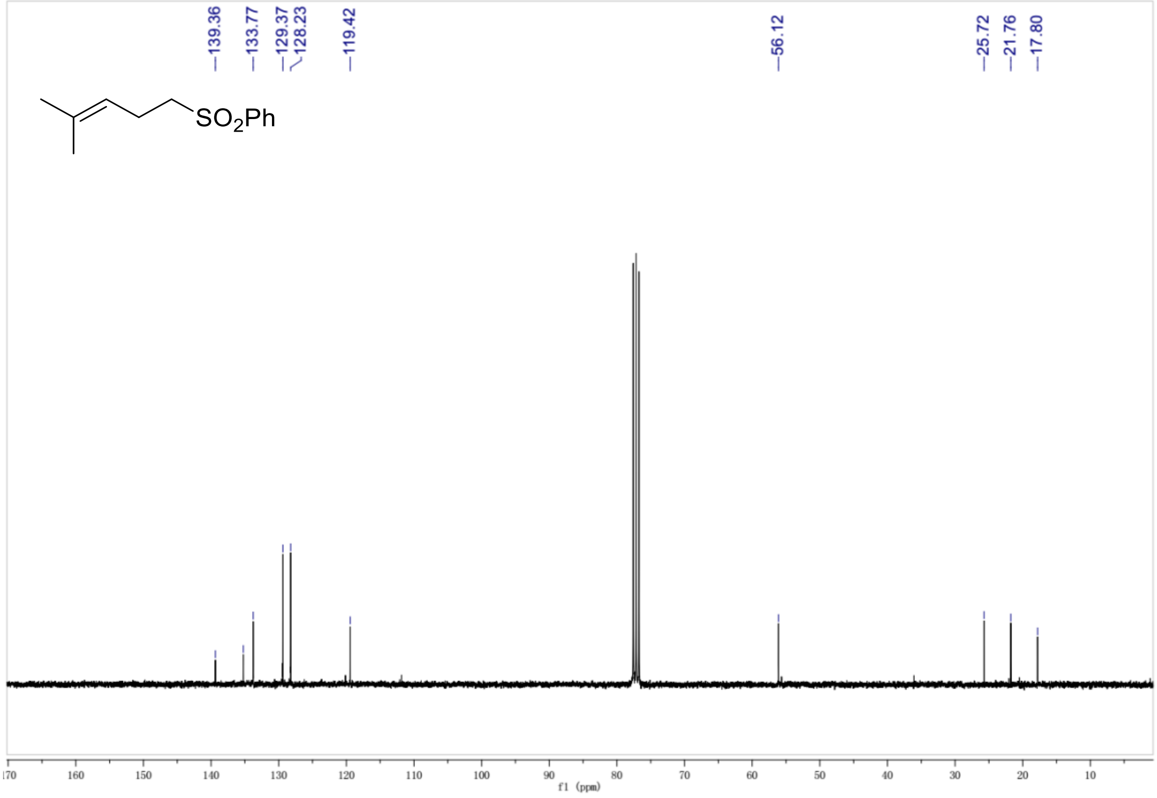

$^{13}C\{^{1}H\}$ NMR spectrum of compound **16a** in $CDCl_3$ (75 MHz).

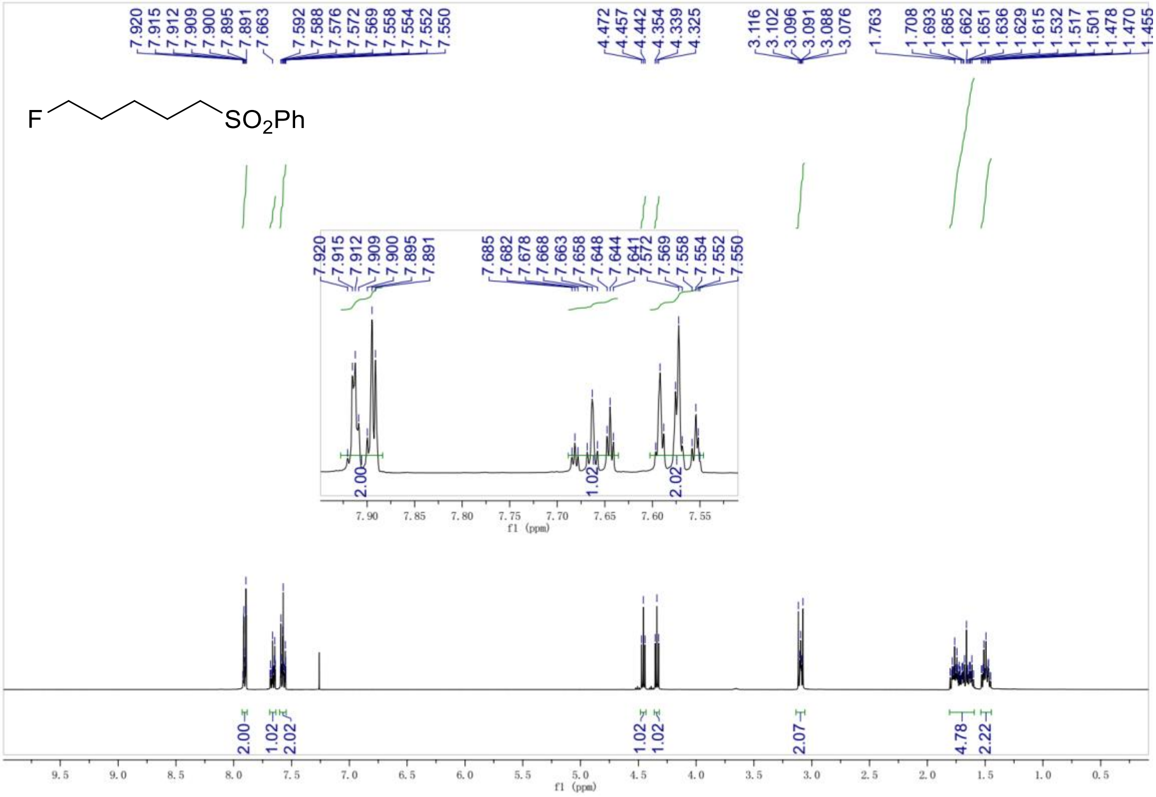

^{1}H NMR spectrum of compound **17a** in $CDCl_3$ (400 MHz).

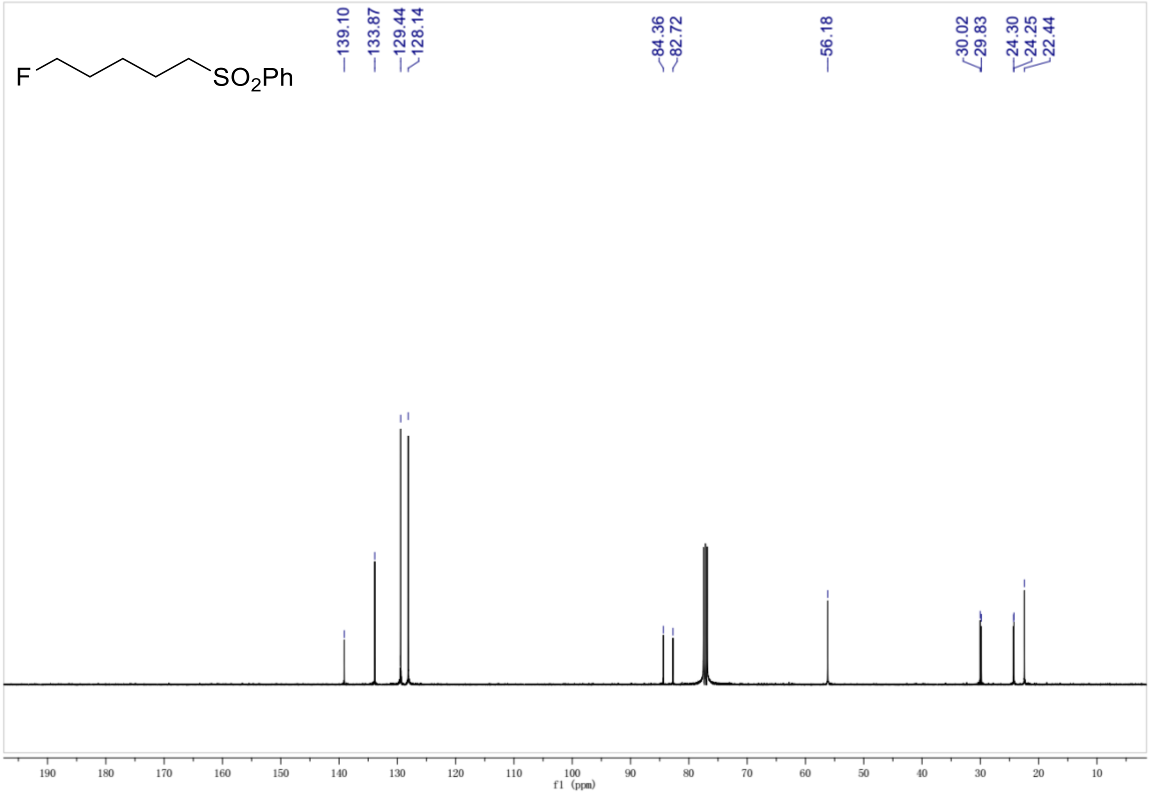

^{13}C{^{1}H} NMR spectrum of compound **17a** in $CDCl_3$ (100 MHz).

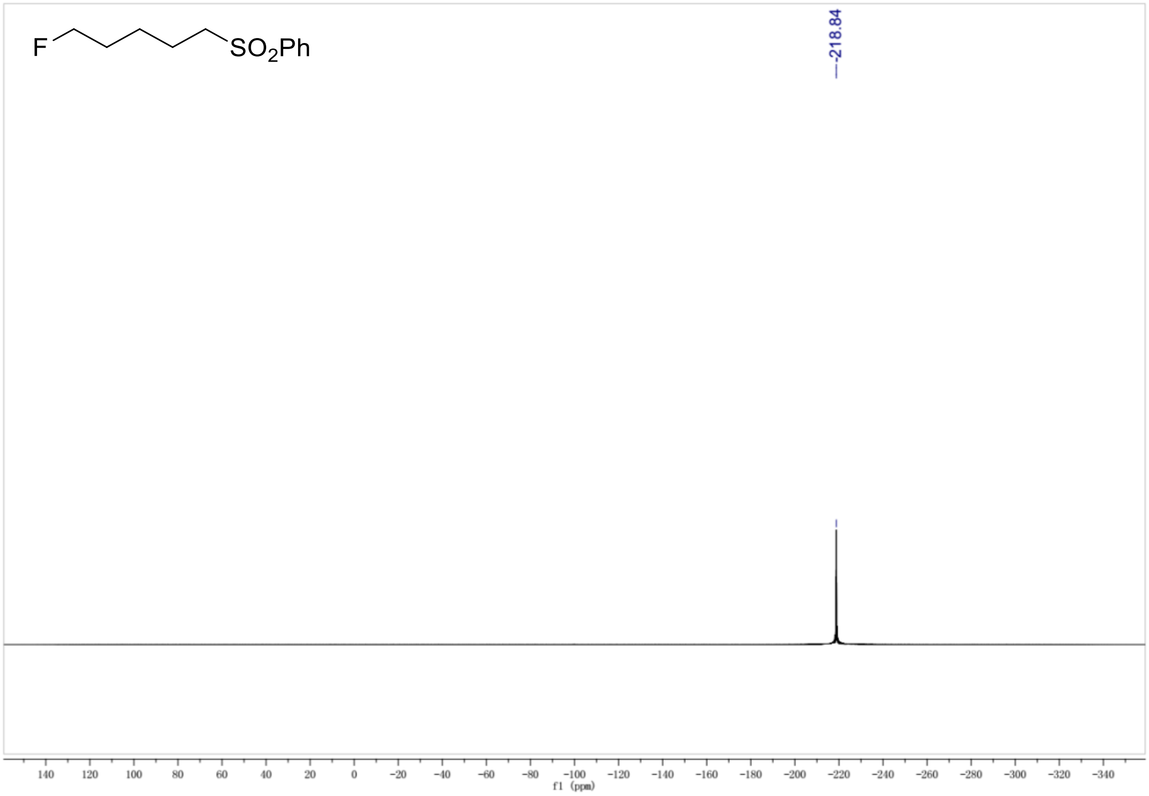

$^{19}F\{^1H\}$ NMR spectrum of compound **17a** in $CDCl_3$ (376 MHz).

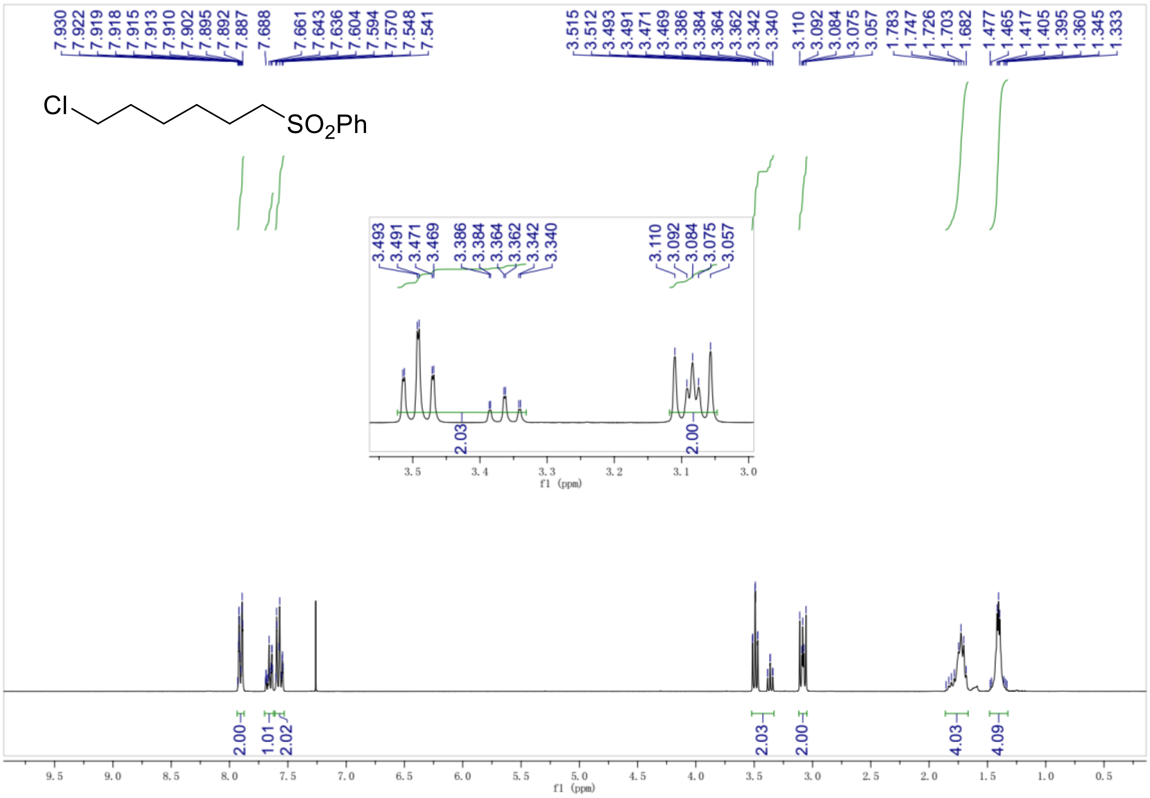

^1H NMR spectrum of compound **18a** in $CDCl_3$ (300 MHz).

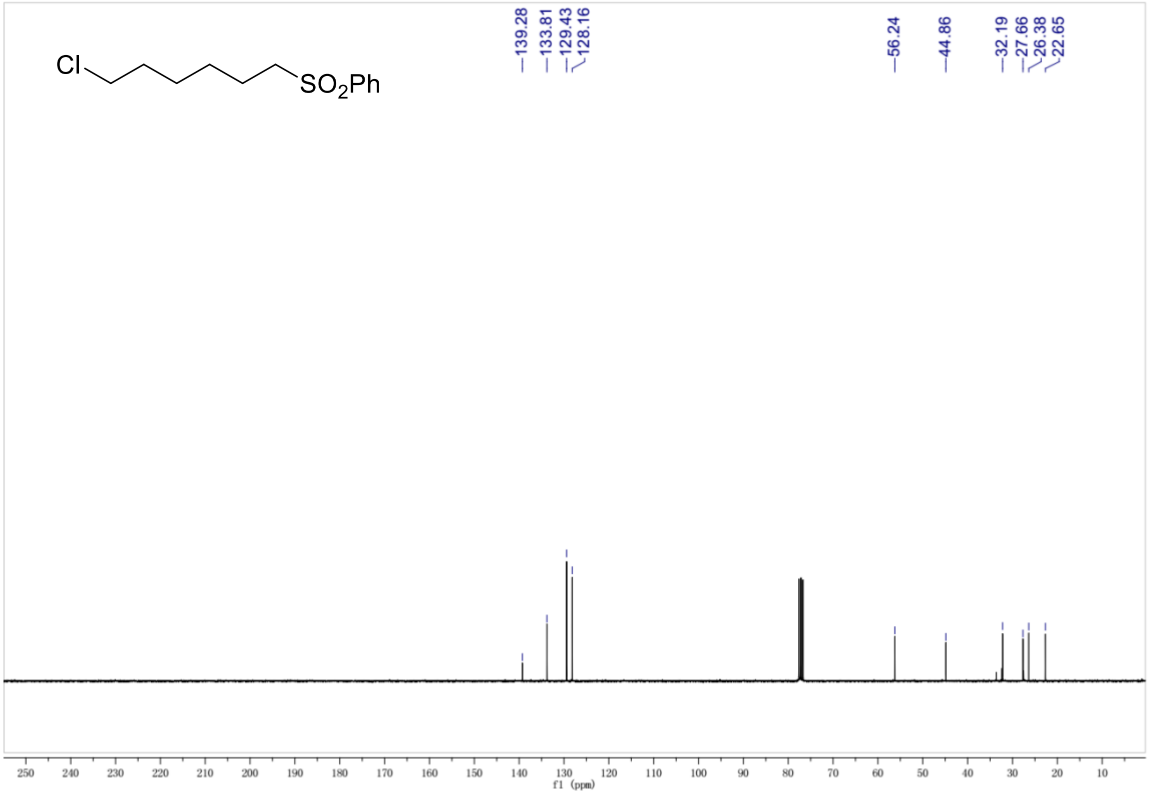

$^{13}C\{^{1}H\}$ NMR spectrum of compound **18a** in $CDCl_3$ (75 MHz).

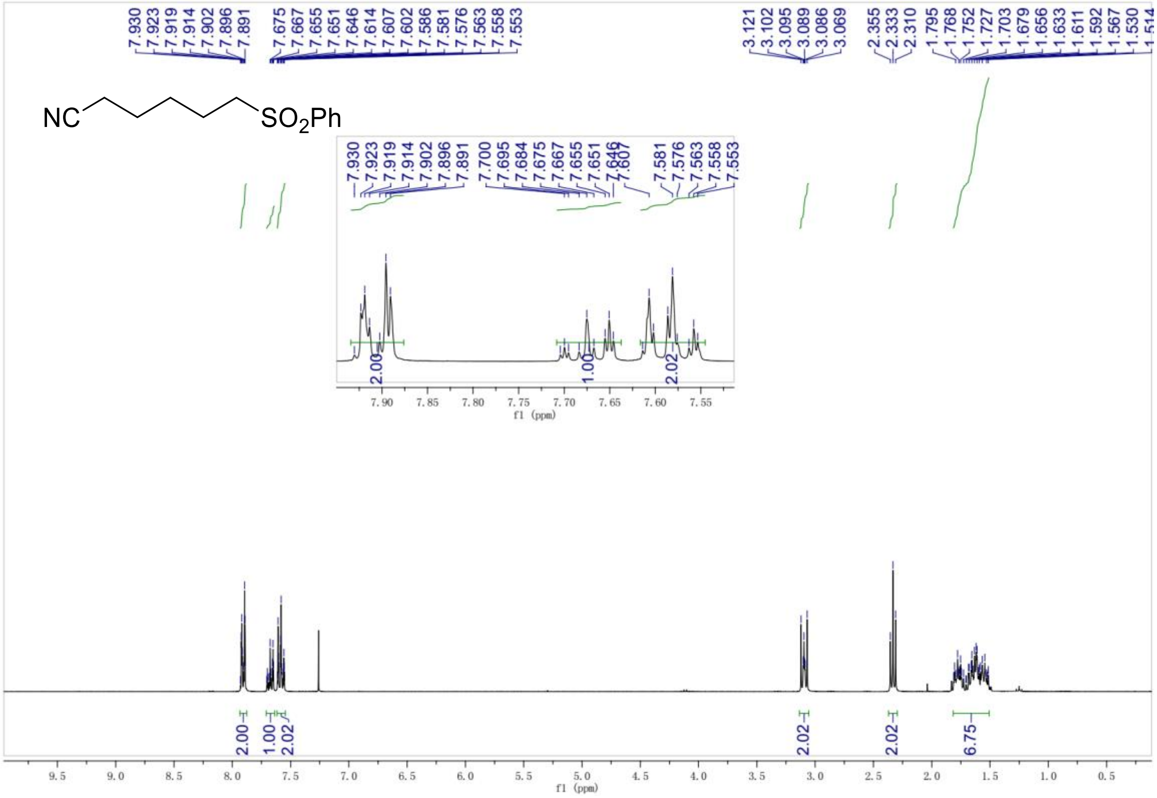

^{1}H NMR spectrum of compound **19a** in $CDCl_3$ (300 MHz).

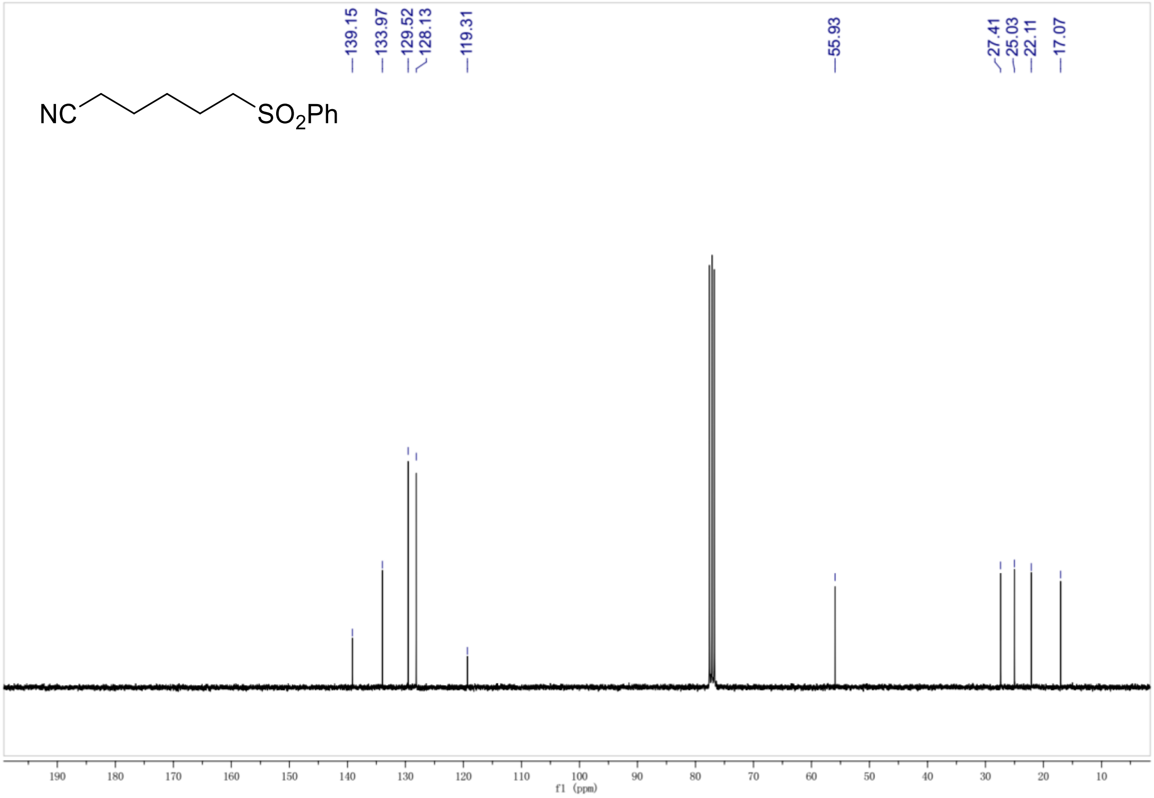

$^{13}C\{^{1}H\}$ NMR spectrum of compound **19a** in $CDCl_3$ (75 MHz).

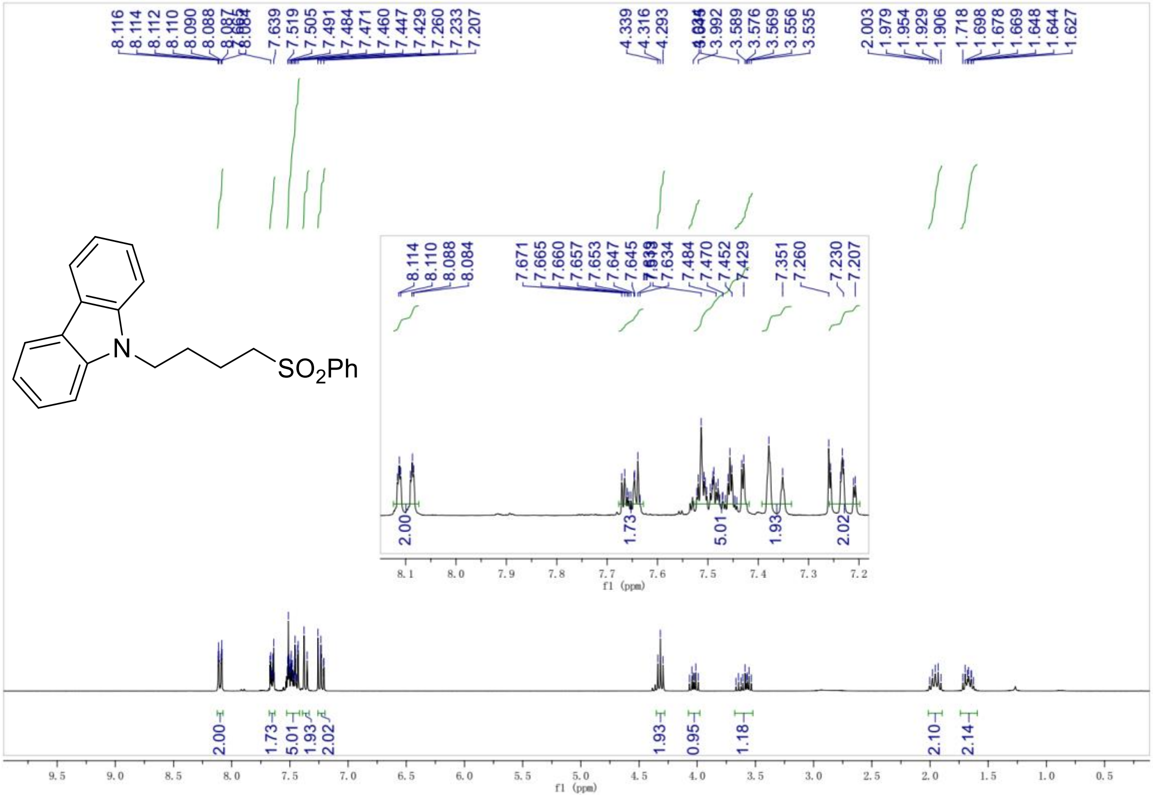

^{1}H NMR spectrum of compound **20a** in $CDCl_3$ (300 MHz).

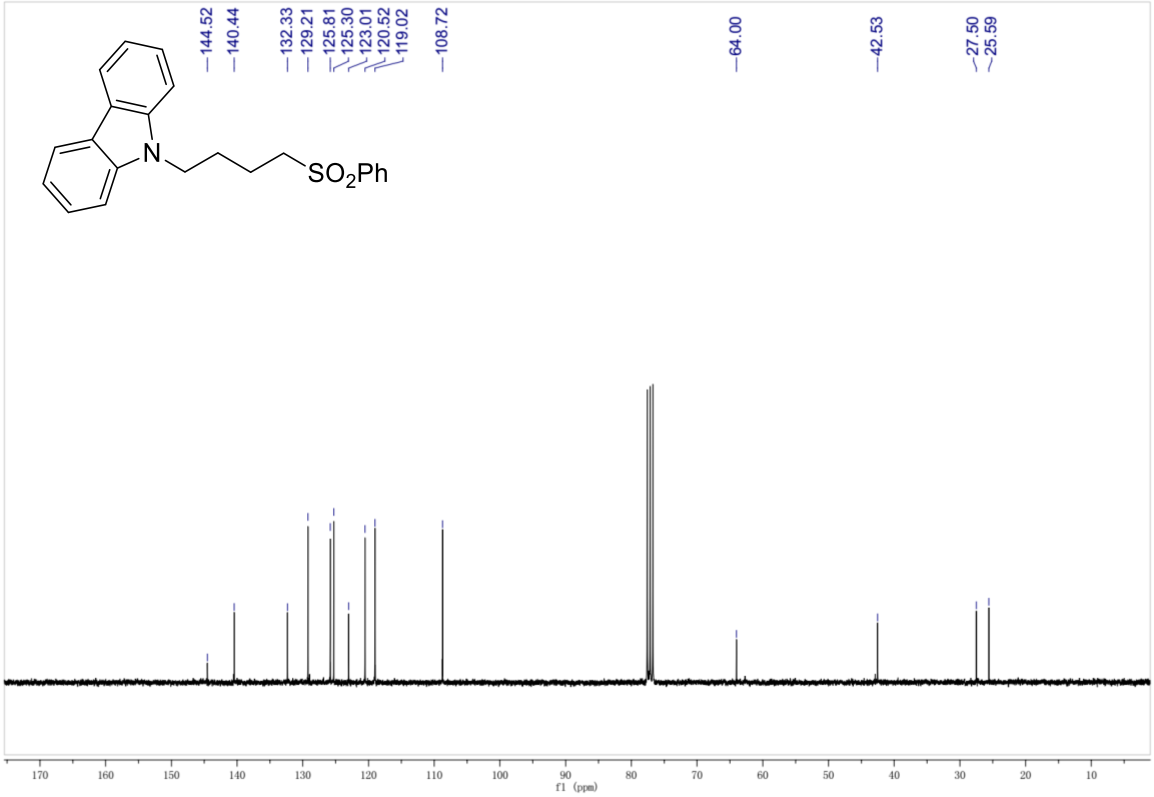

$^{13}C\{^{1}H\}$ NMR spectrum of compound **20a** in $CDCl_3$ (75 MHz).

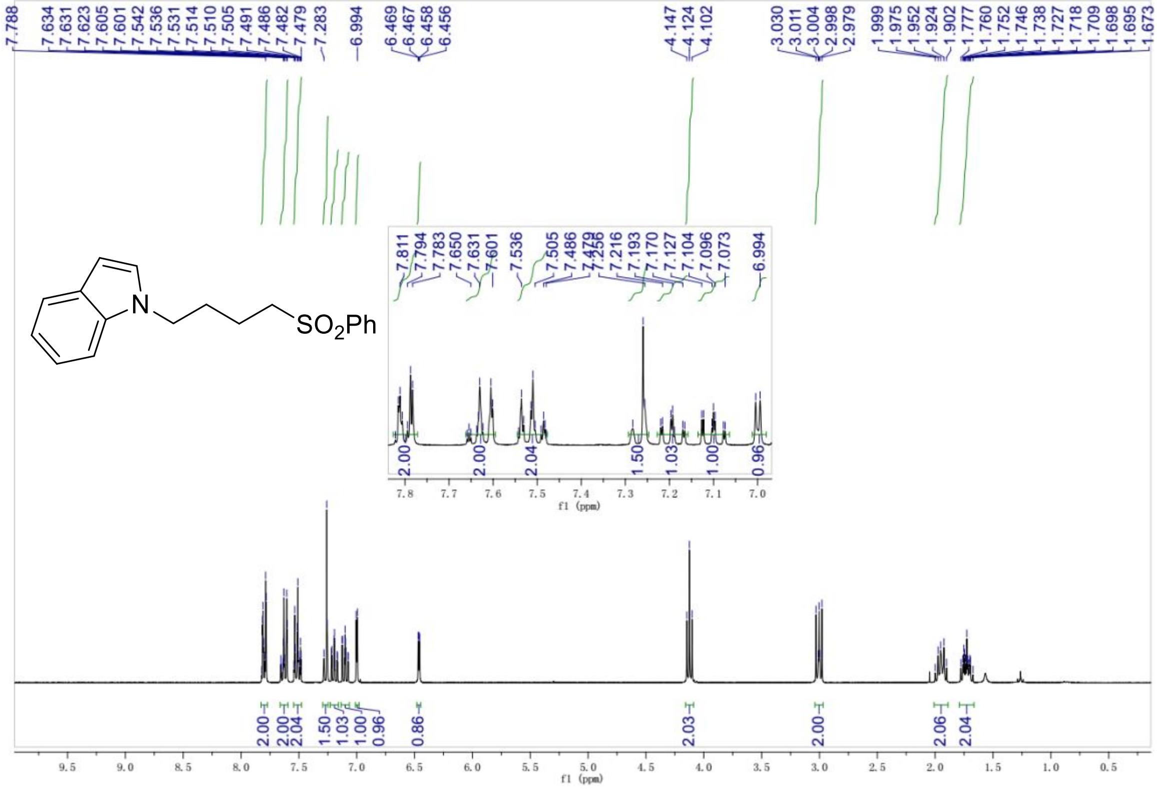

^{1}H NMR spectrum of compound **21a** in $CDCl_3$ (300 MHz).

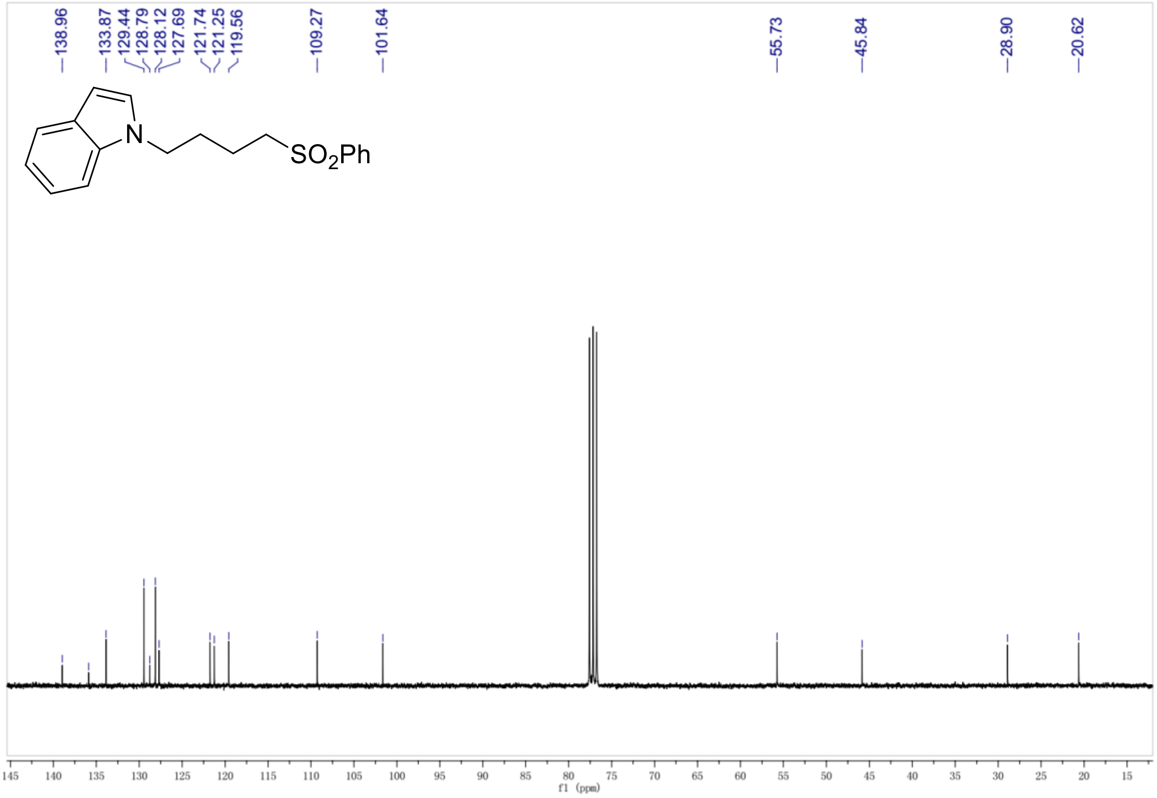

$^{13}C\{^{1}H\}$ NMR spectrum of compound **21a** in $CDCl_3$ (75 MHz).

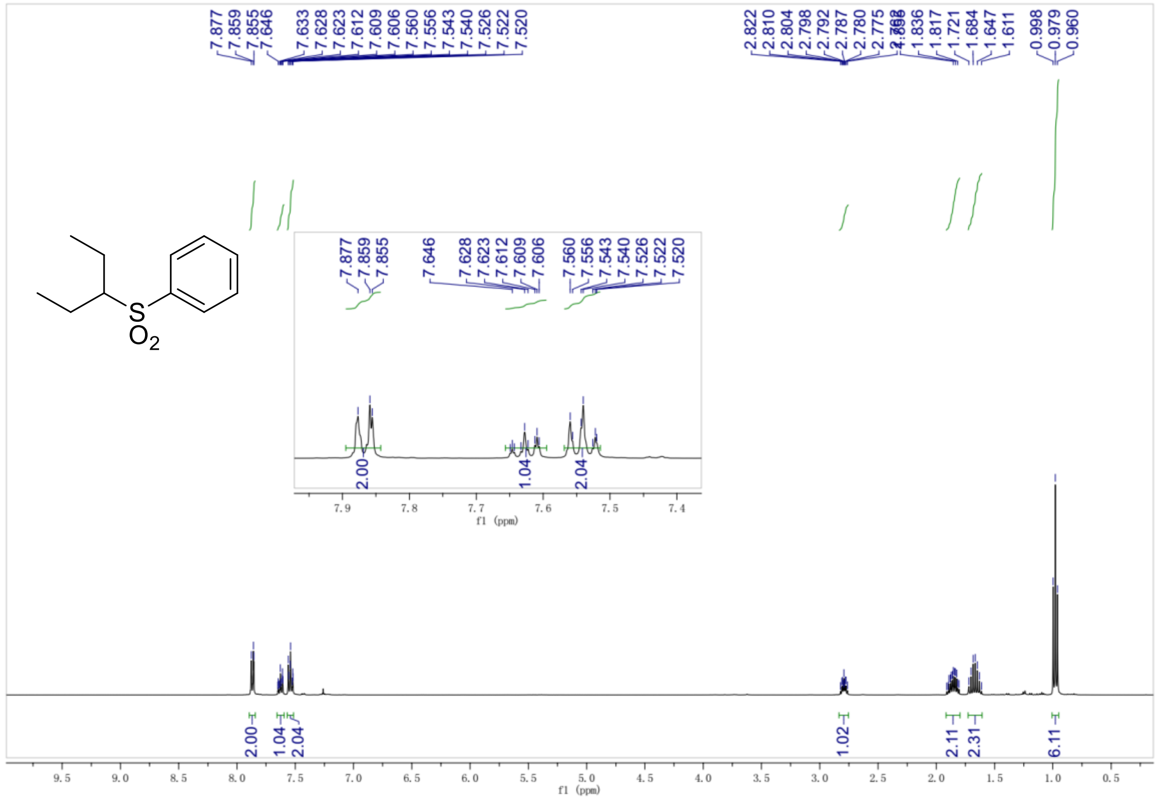

^{1}H NMR spectrum of compound **22a** in $CDCl_3$ (400 MHz).

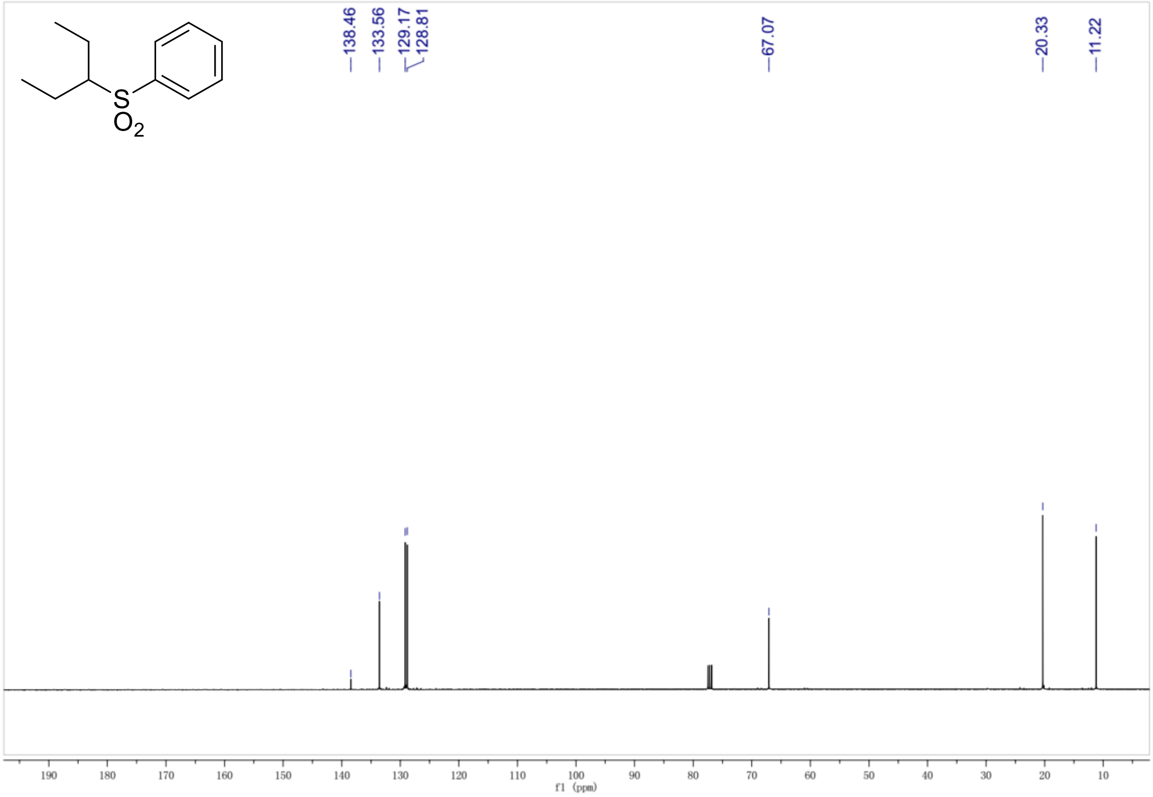

$^{13}C\{^{1}H\}$ NMR spectrum of compound **22a** in $CDCl_3$ (100 MHz).

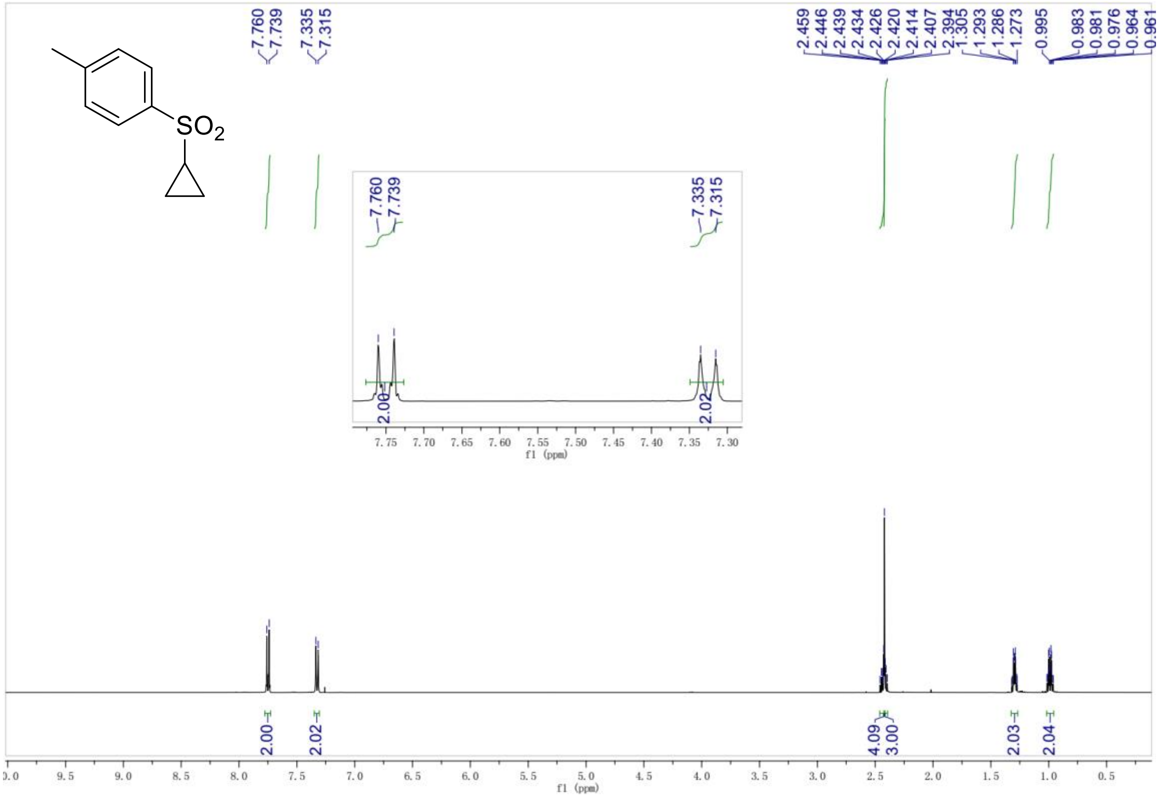

^{1}H NMR spectrum of compound **23a** in $CDCl_3$ (400 MHz).

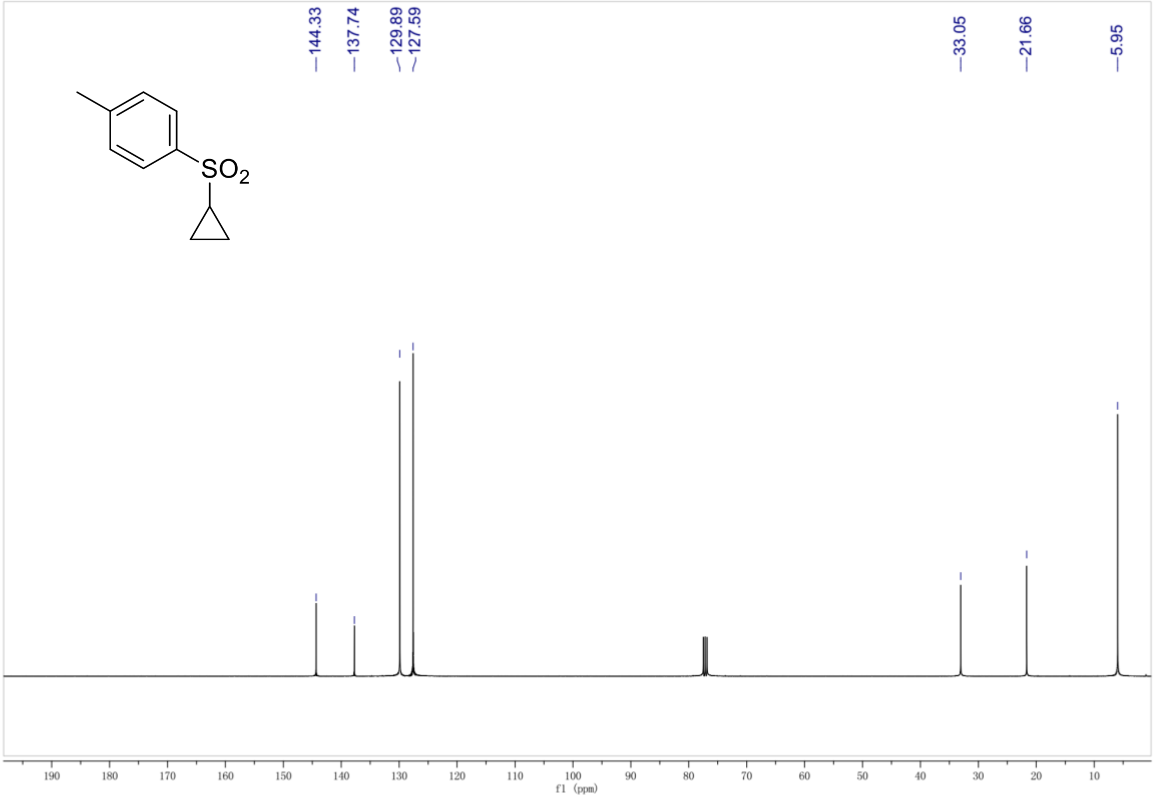

$^{13}C\{^{1}H\}$ NMR spectrum of compound **23a** in $CDCl_3$ (100 MHz).

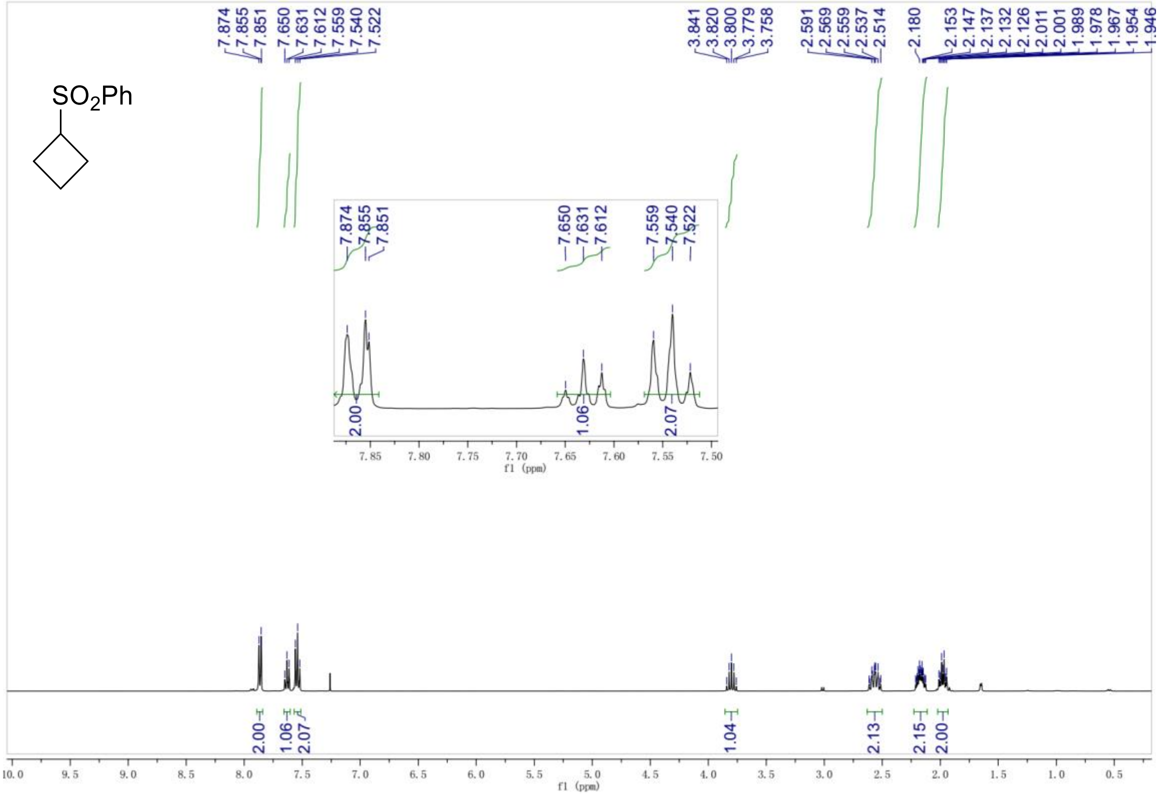

^{1}H NMR spectrum of compound **24a** in $CDCl_3$ (400 MHz).

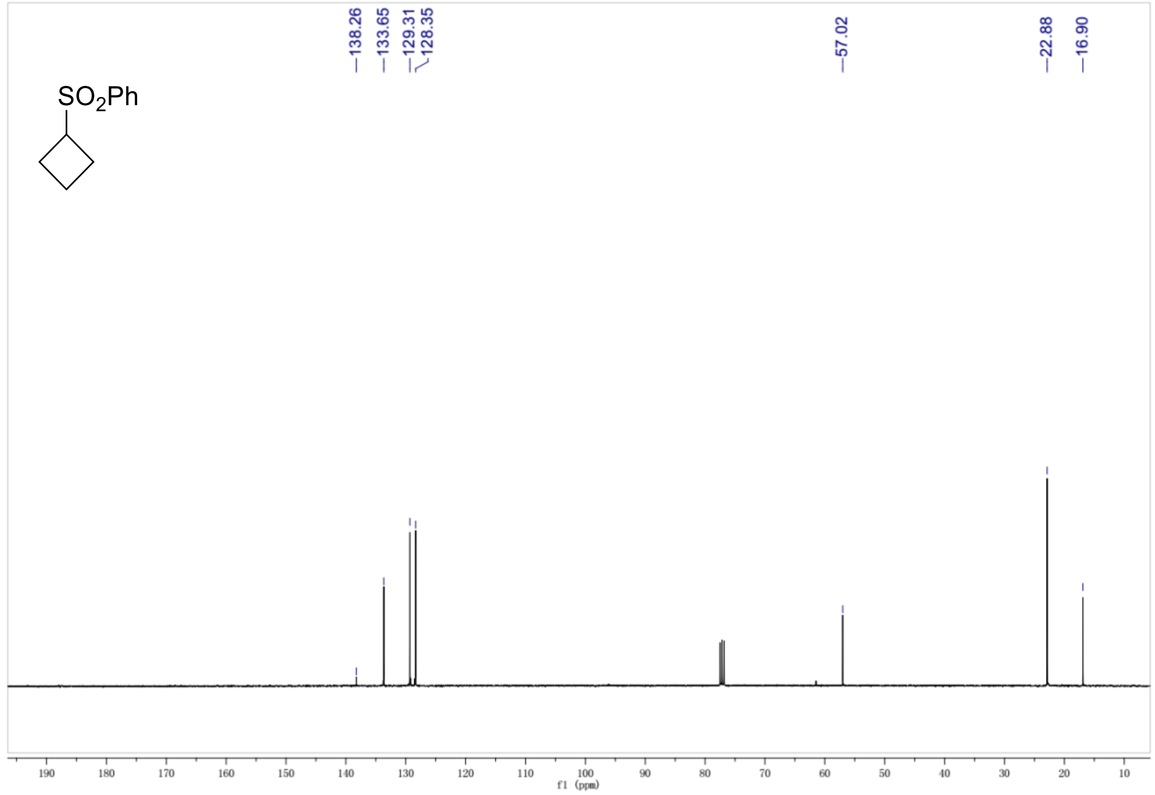

$^{13}C\{^1H\}$ NMR spectrum of compound **24a** in $CDCl_3$ (100 MHz).

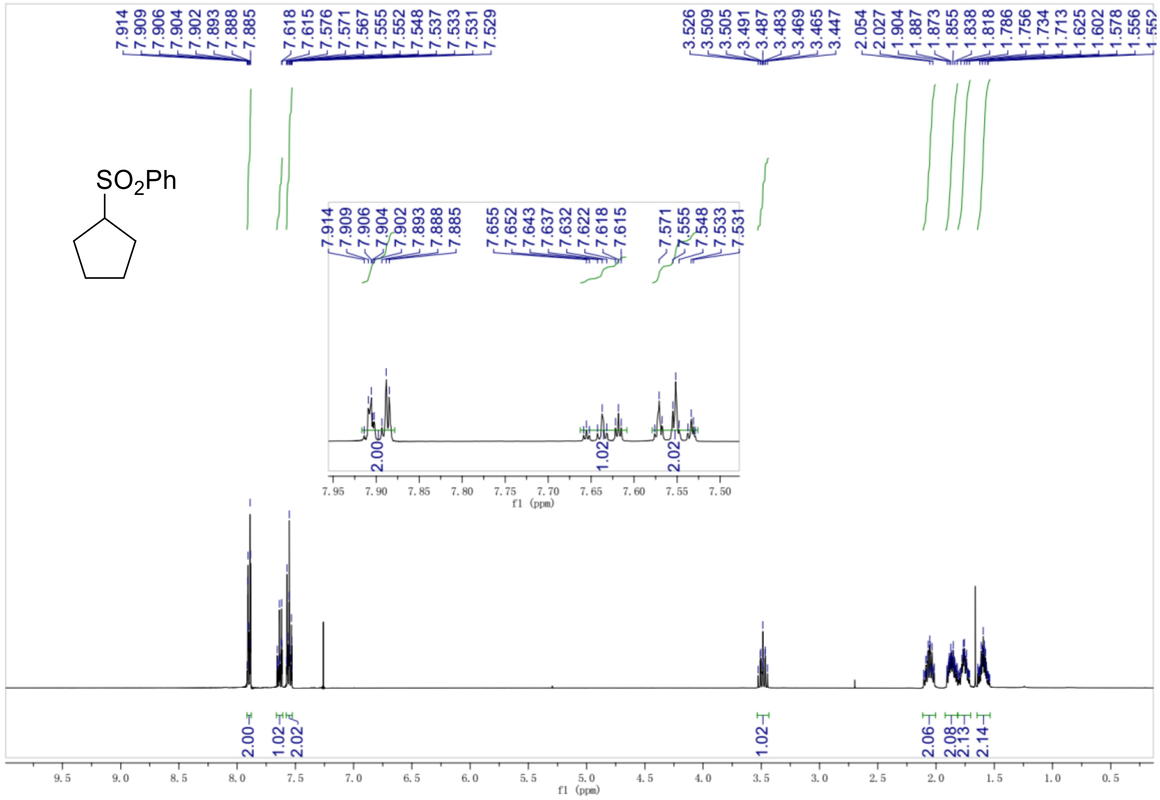

^1H NMR spectrum of compound **25a** in $CDCl_3$ (400 MHz).

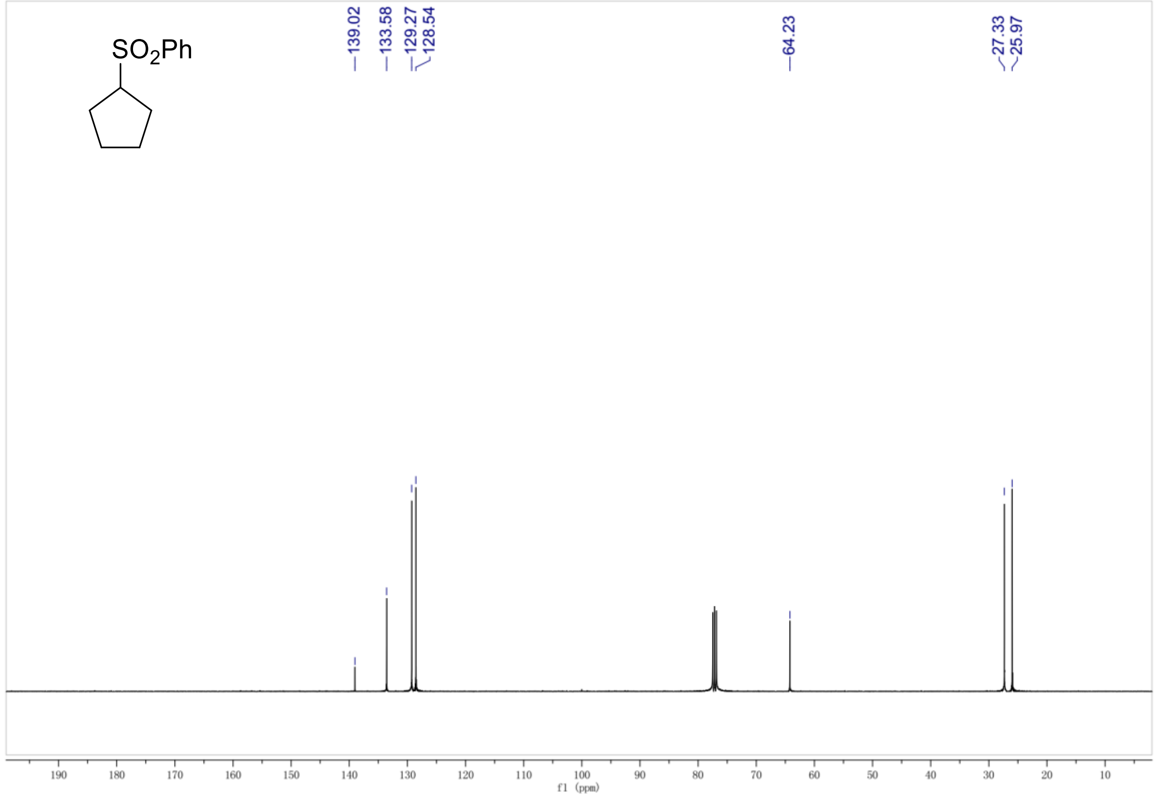

$^{13}C\{^{1}H\}$ NMR spectrum of compound **25a** in $CDCl_3$ (100 MHz).

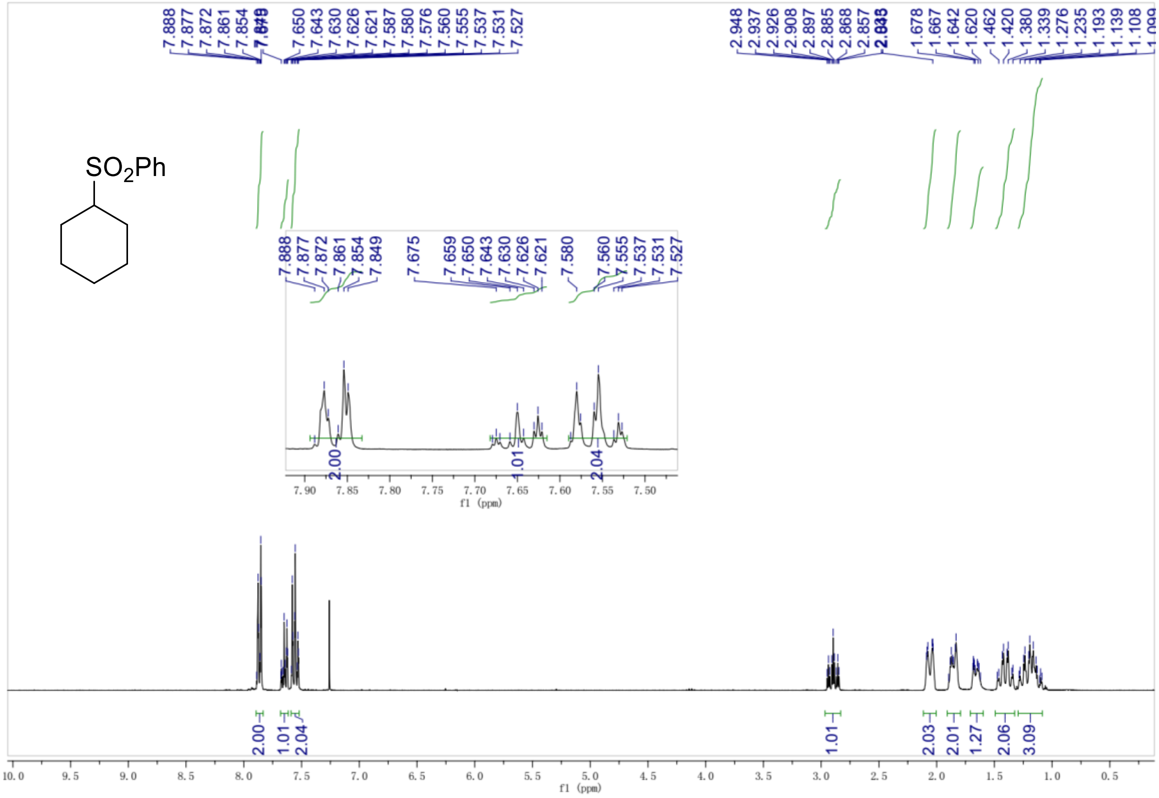

^{1}H NMR spectrum of compound **26a** in $CDCl_3$ (300 MHz).

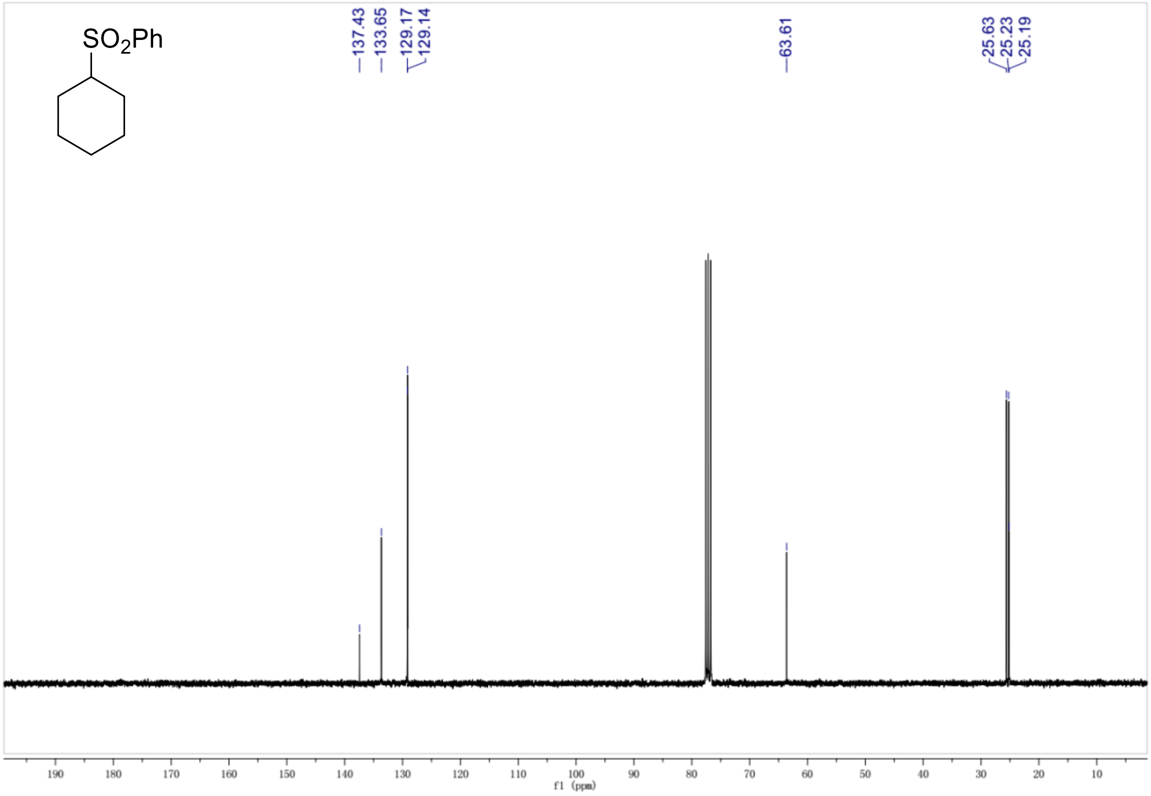

$^{13}C\{^{1}H\}$ NMR spectrum of compound **26a** in $CDCl_3$ (75 MHz).

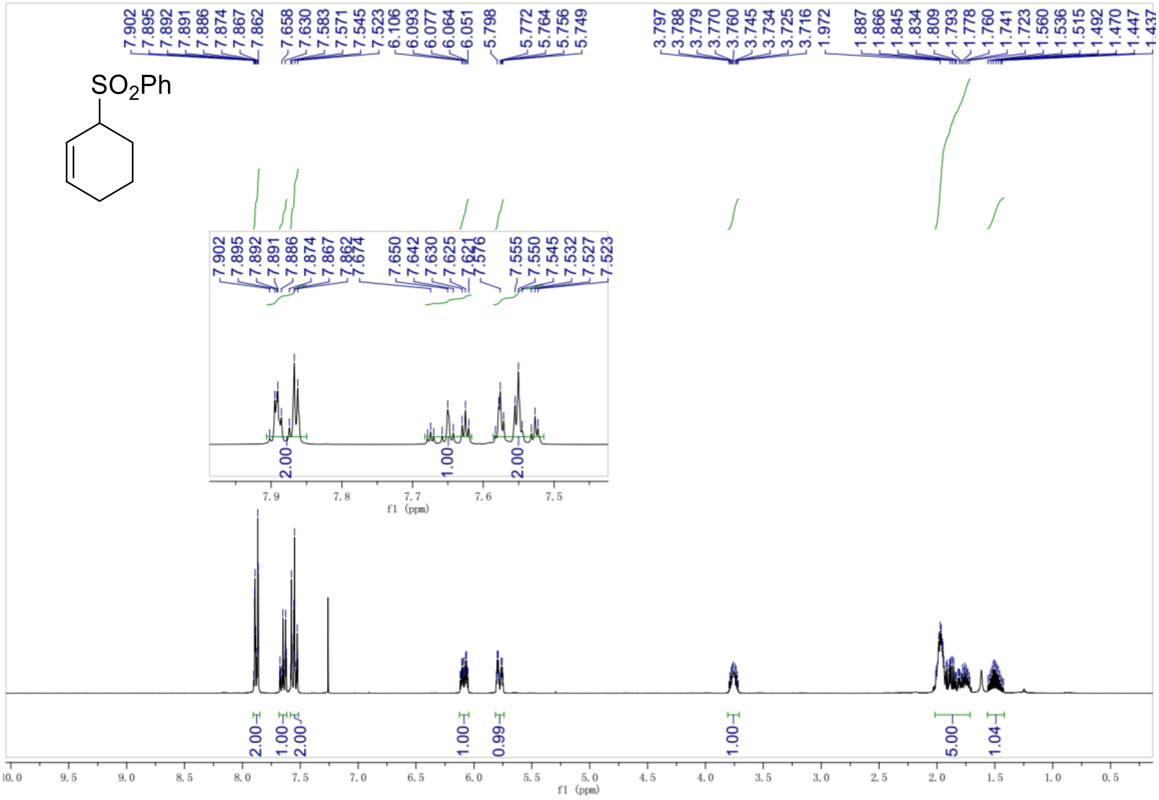

^{1}H NMR spectrum of compound **27a** in $CDCl_3$ (300 MHz).

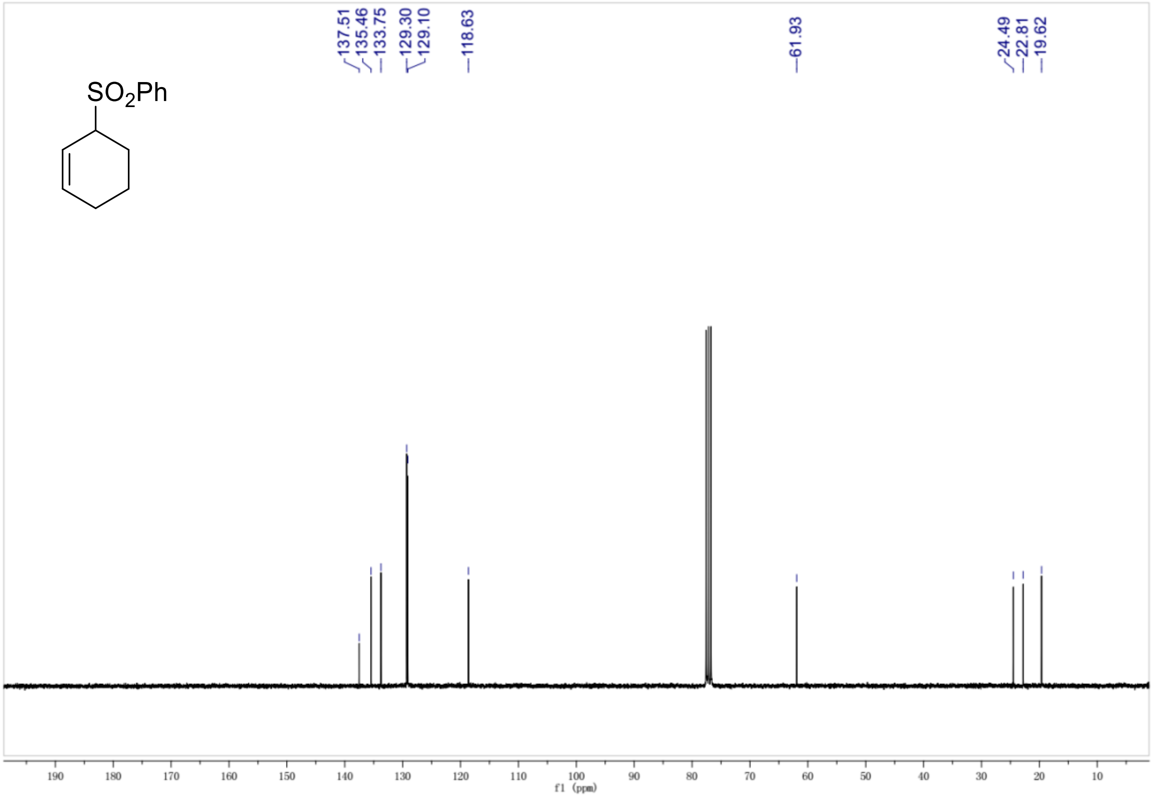

$^{13}C\{^{1}H\}$ NMR spectrum of compound **27a** in $CDCl_3$ (75 MHz).

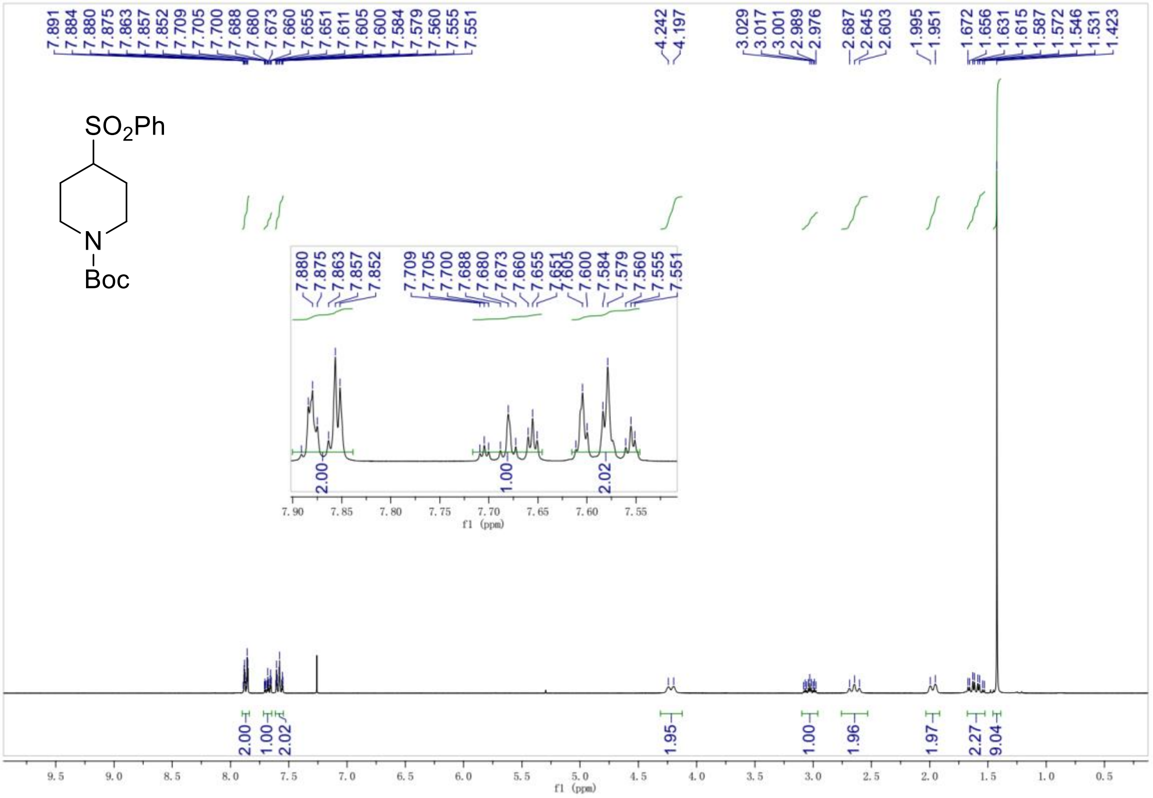

^{1}H NMR spectrum of compound **28a** in $CDCl_3$ (300 MHz).

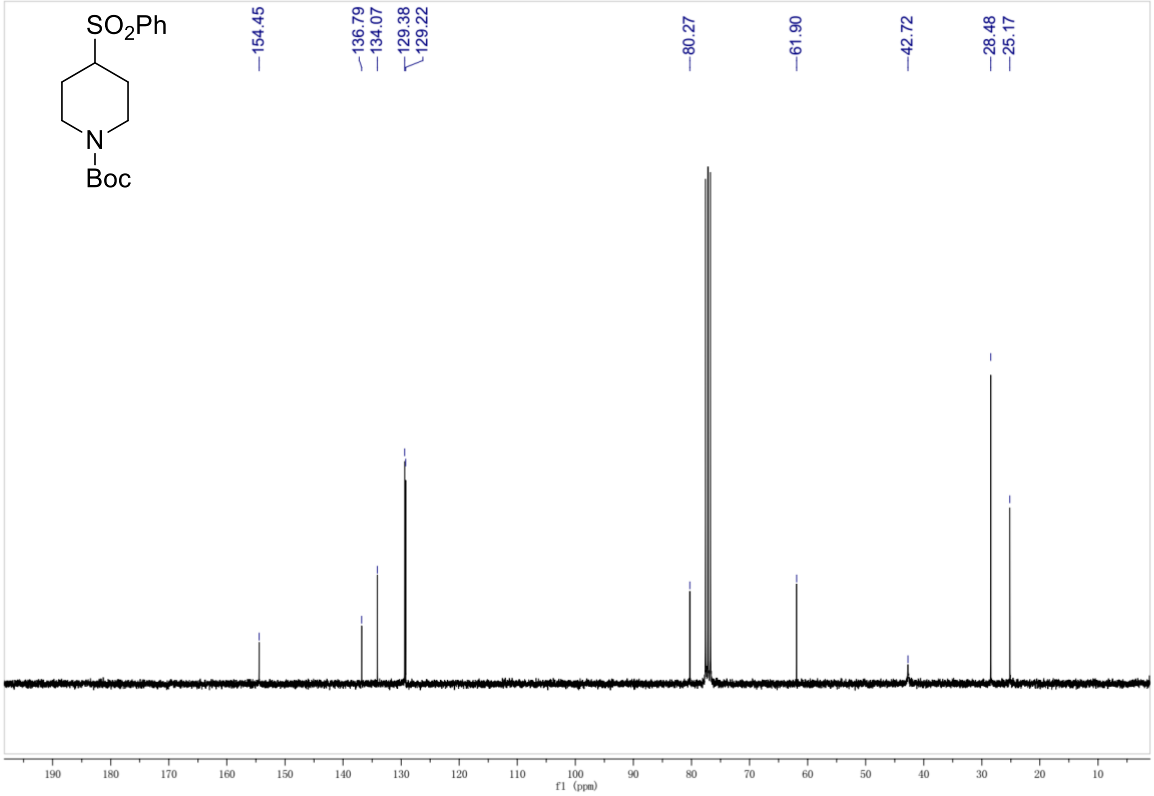

$^{13}C\{^1H\}$ NMR spectrum of compound **28a** in $CDCl_3$ (75 MHz).

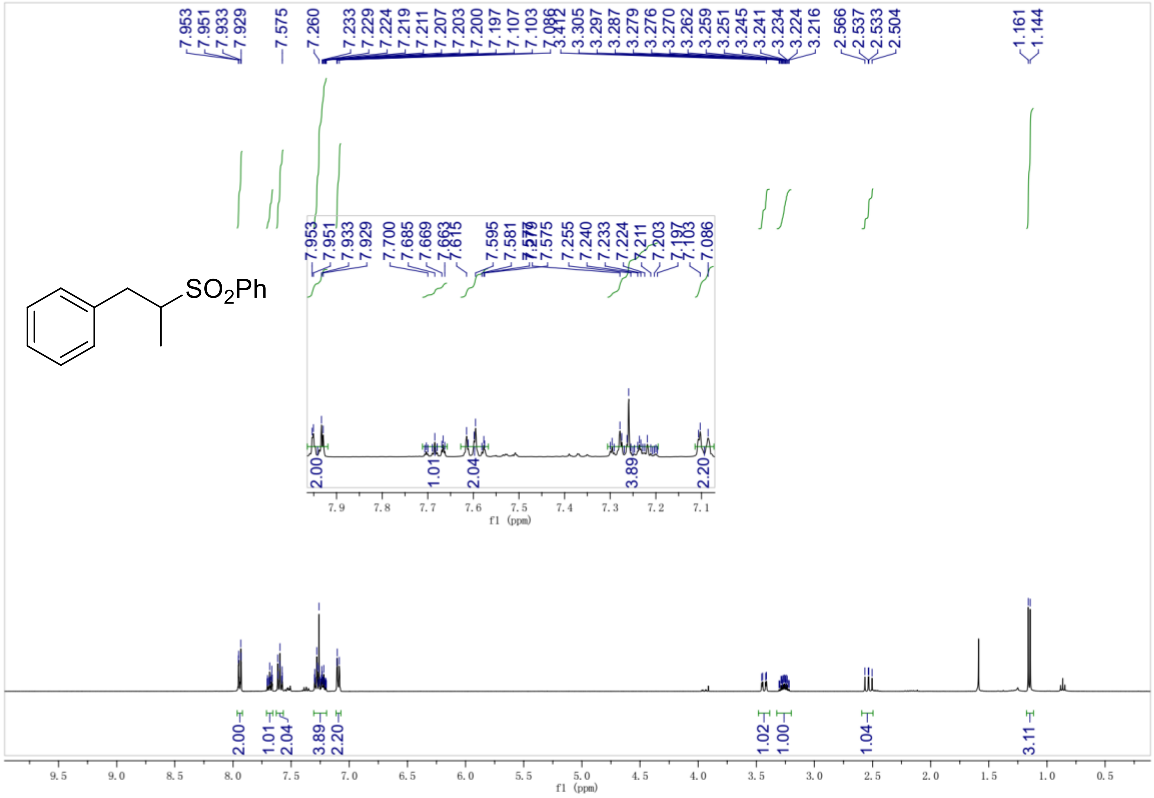

^1H NMR spectrum of compound **29a** in $CDCl_3$ (400 MHz).

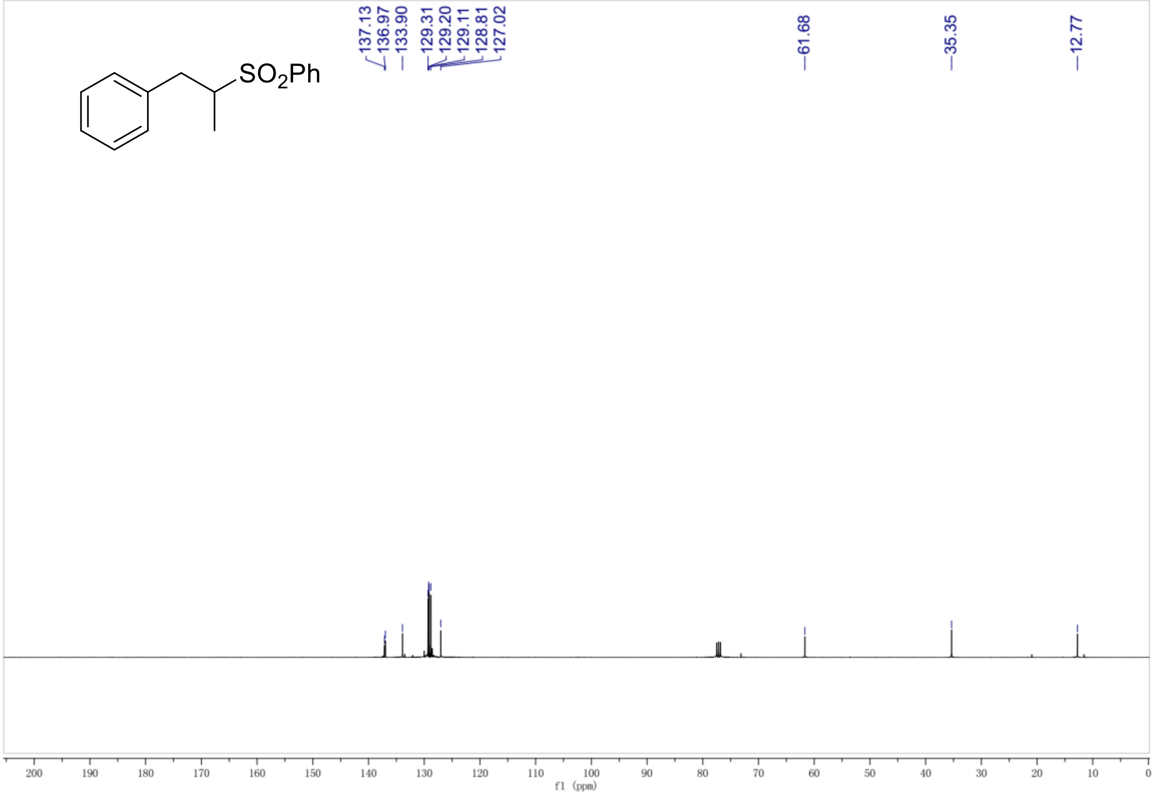

$^{13}C\{^{1}H\}$ NMR spectrum of compound **29a** in $CDCl_3$ (100 MHz).

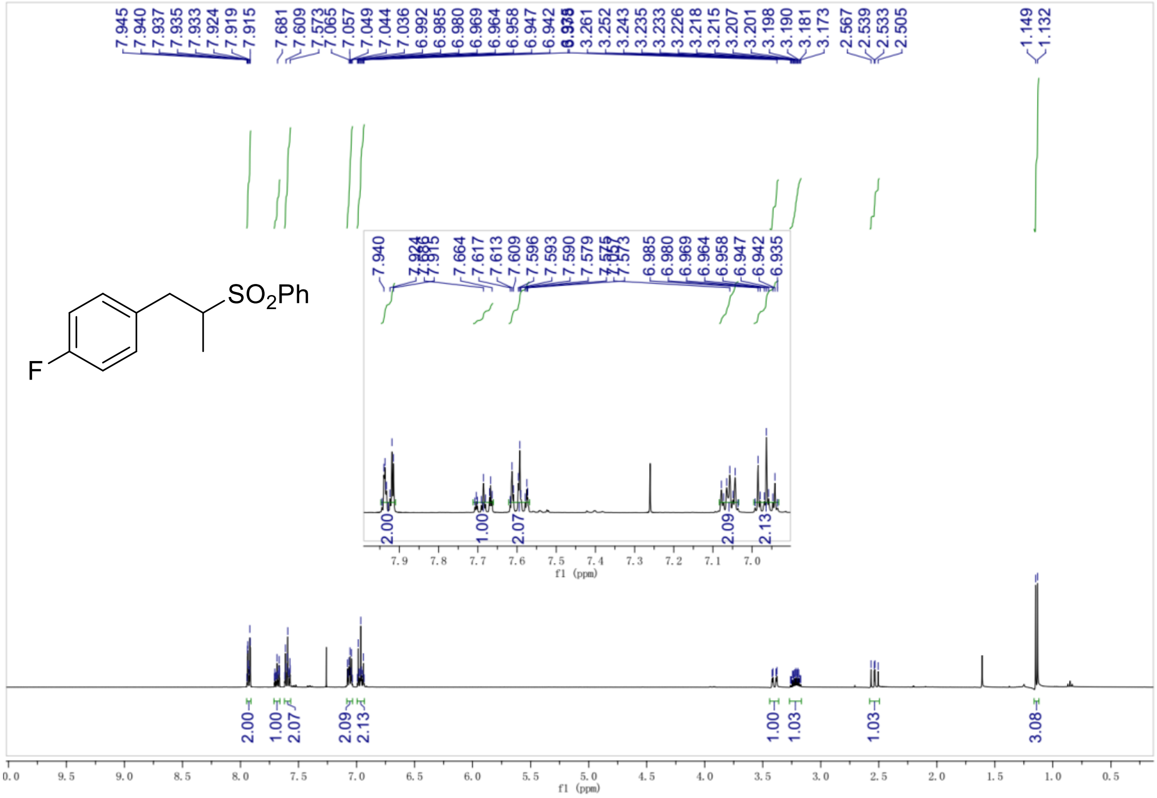

^{1}H NMR spectrum of compound **30a** in $CDCl_3$ (400 MHz).

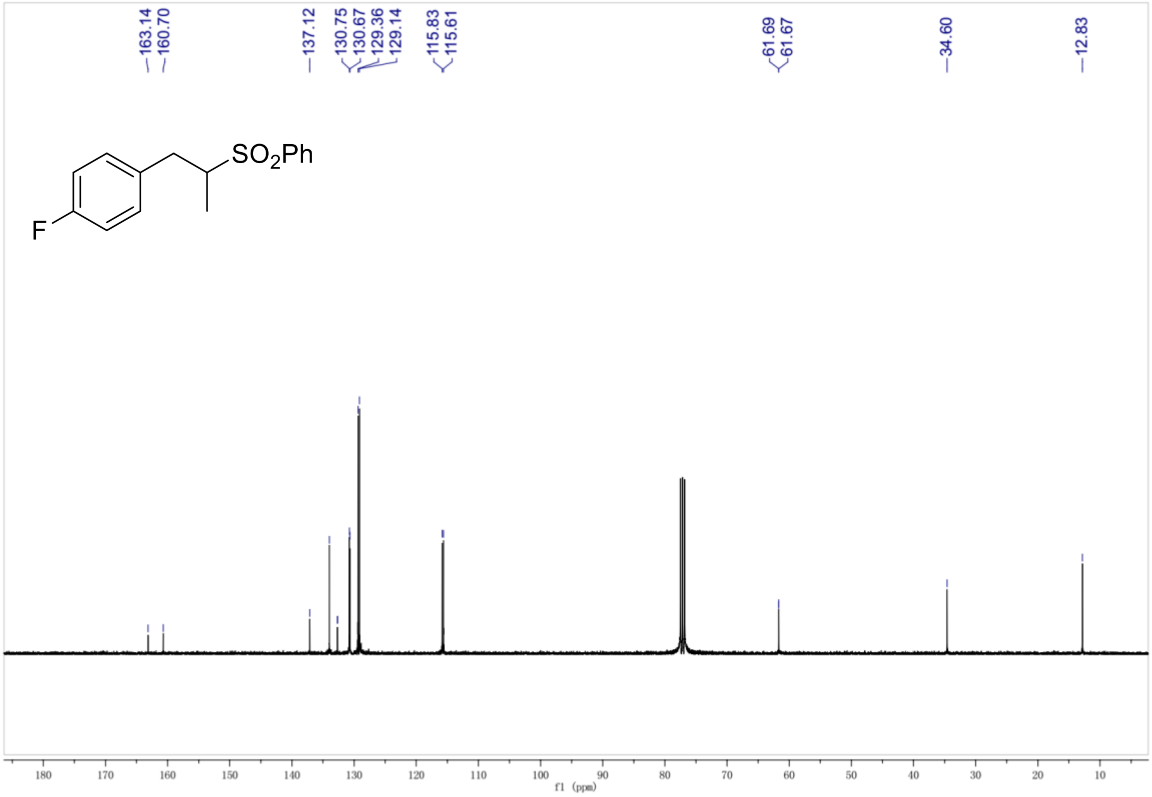

$^{13}C\{^{1}H\}$ NMR spectrum of compound **30a** in $CDCl_3$ (100 MHz).

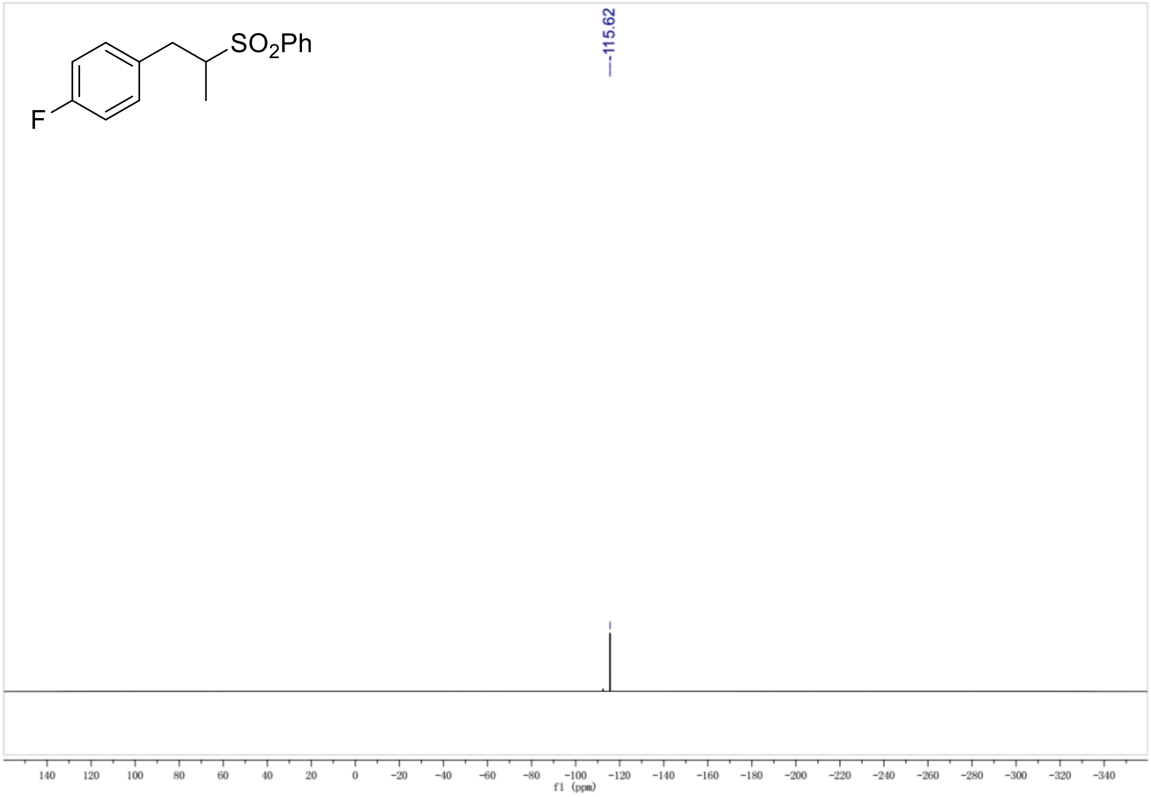

$^{19}F\{^{1}H\}$ NMR spectrum of compound **30a** in $CDCl_3$ (376 MHz).

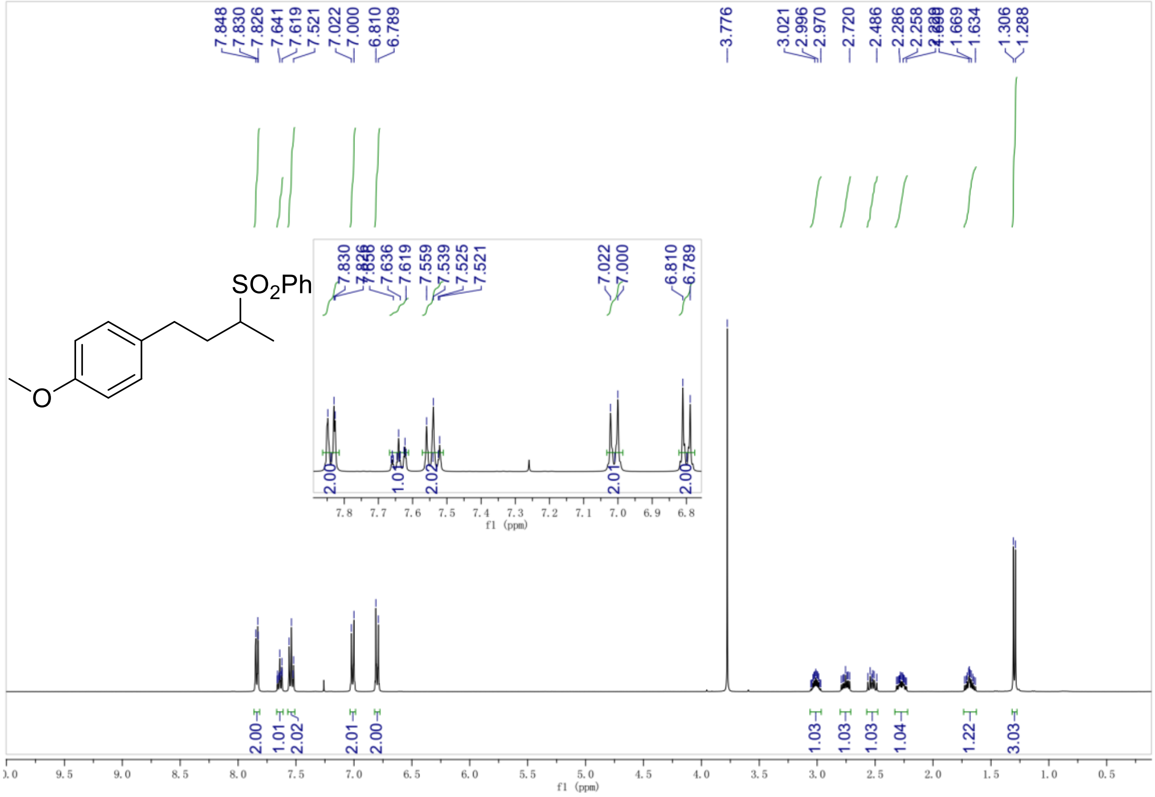

^{1}H NMR spectrum of compound **31a** in $CDCl_3$ (400 MHz).

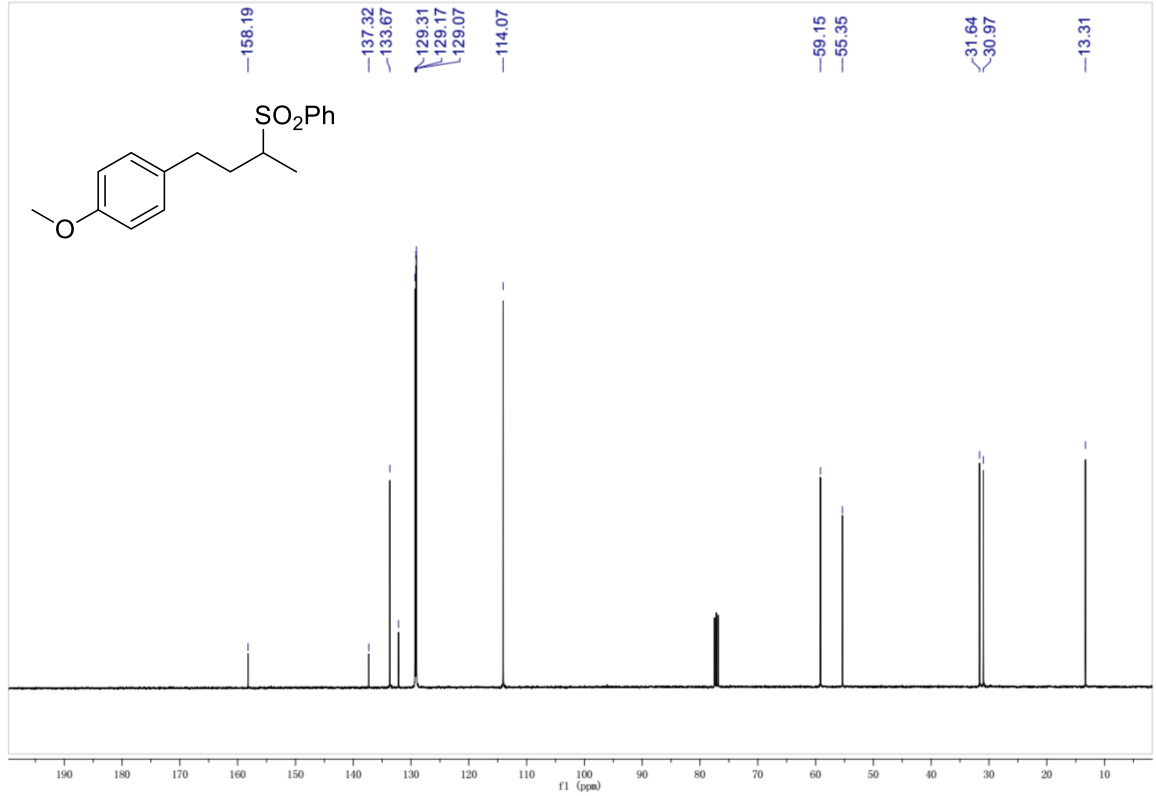

$^{13}C\{^{1}H\}$ NMR spectrum of compound **31a** in $CDCl_3$ (100 MHz).

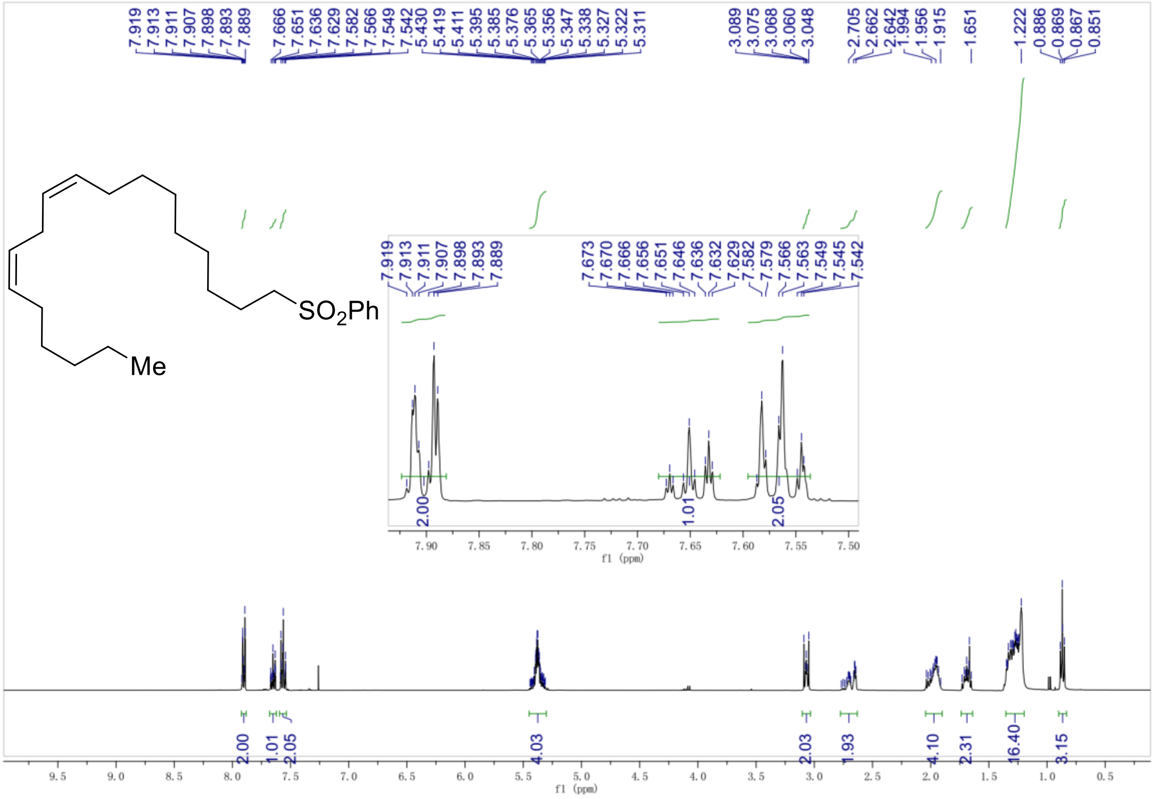

^{1}H NMR spectrum of compound **32a** in $CDCl_3$ (400 MHz).

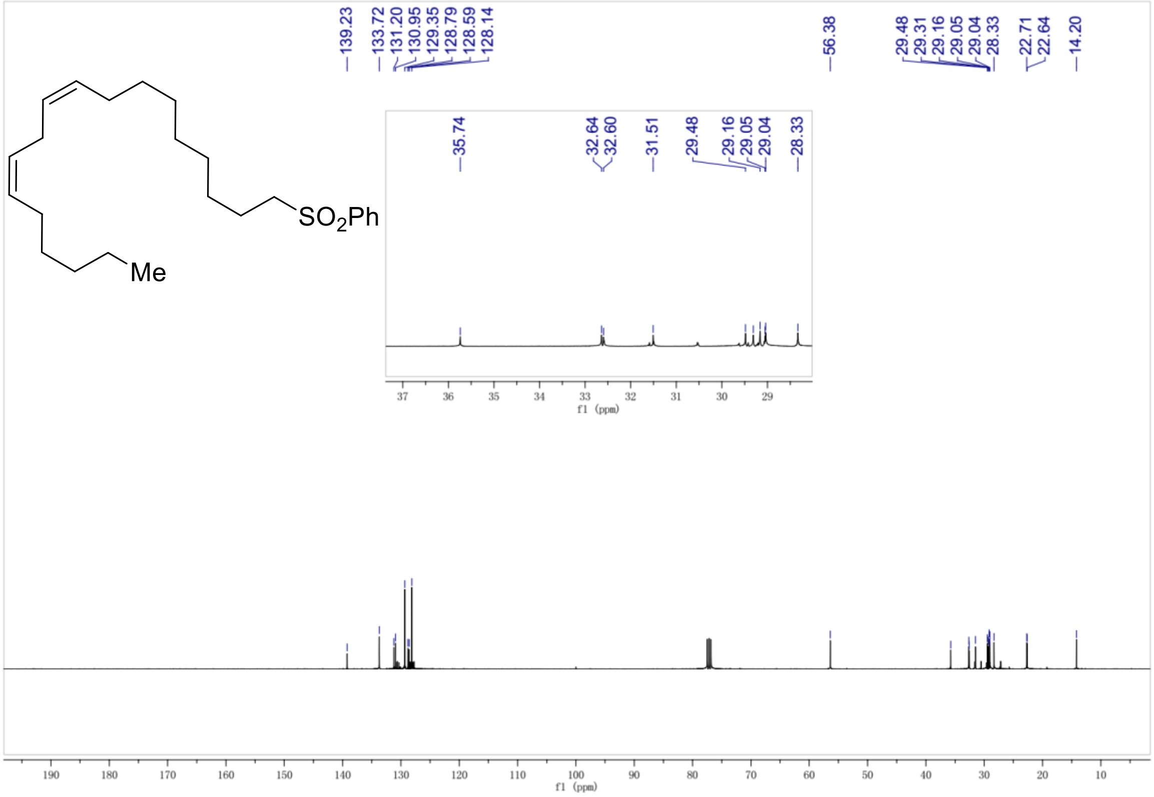

^{13}C{^{1}H} NMR spectrum of compound **32a** in $CDCl_3$ (100 MHz).

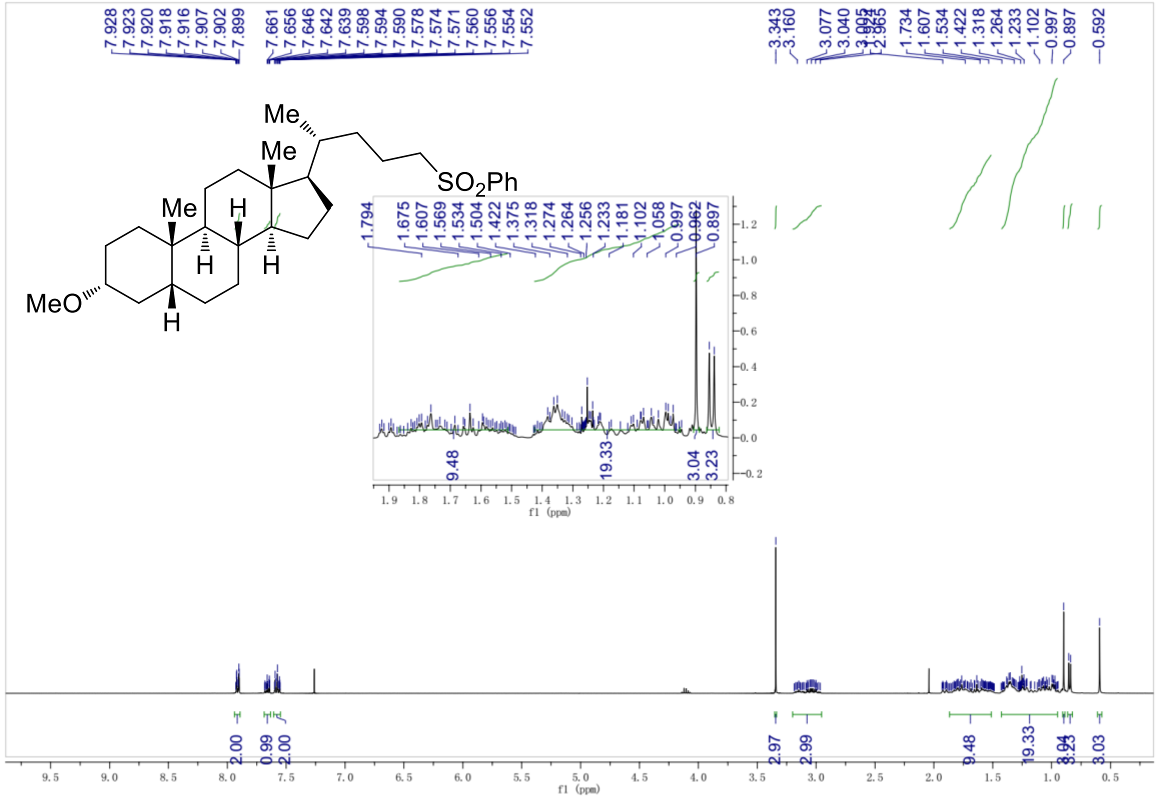

^{1}H NMR spectrum of compound **33a** in $CDCl_3$ (400 MHz).

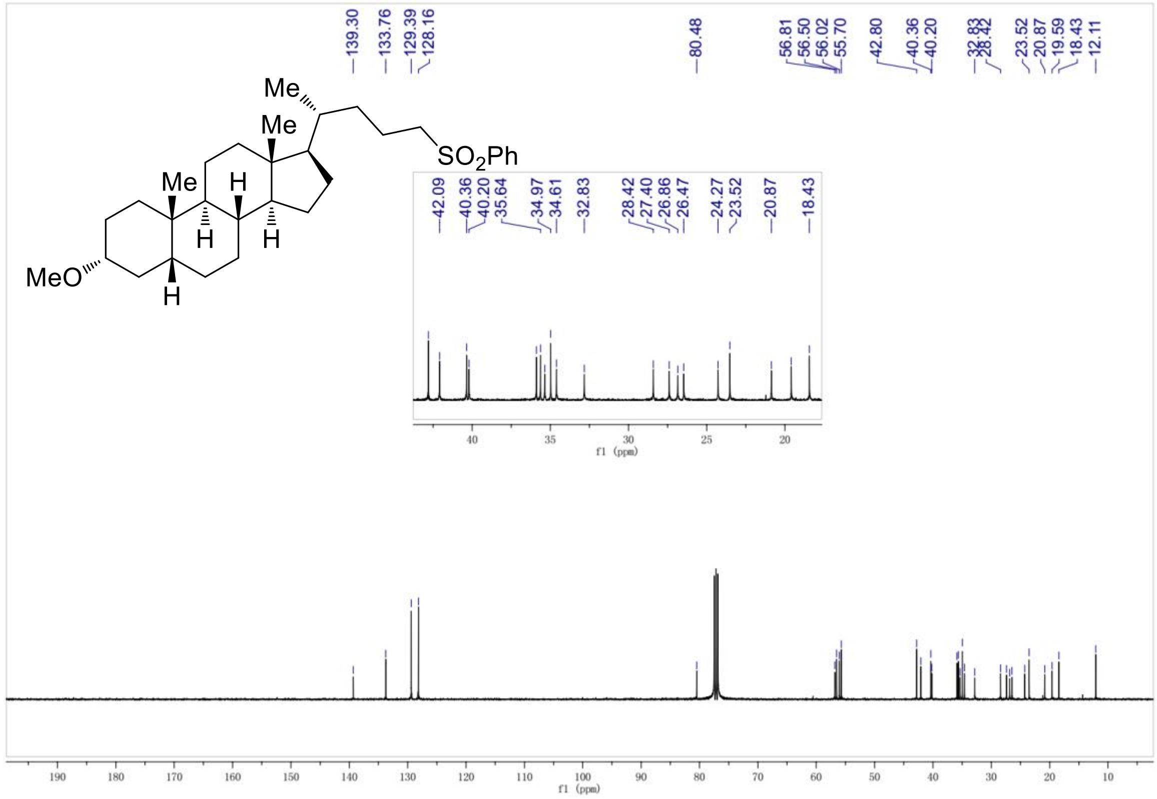

$^{13}C\{^{1}H\}$ NMR spectrum of compound **33a** in $CDCl_3$ (100 MHz).

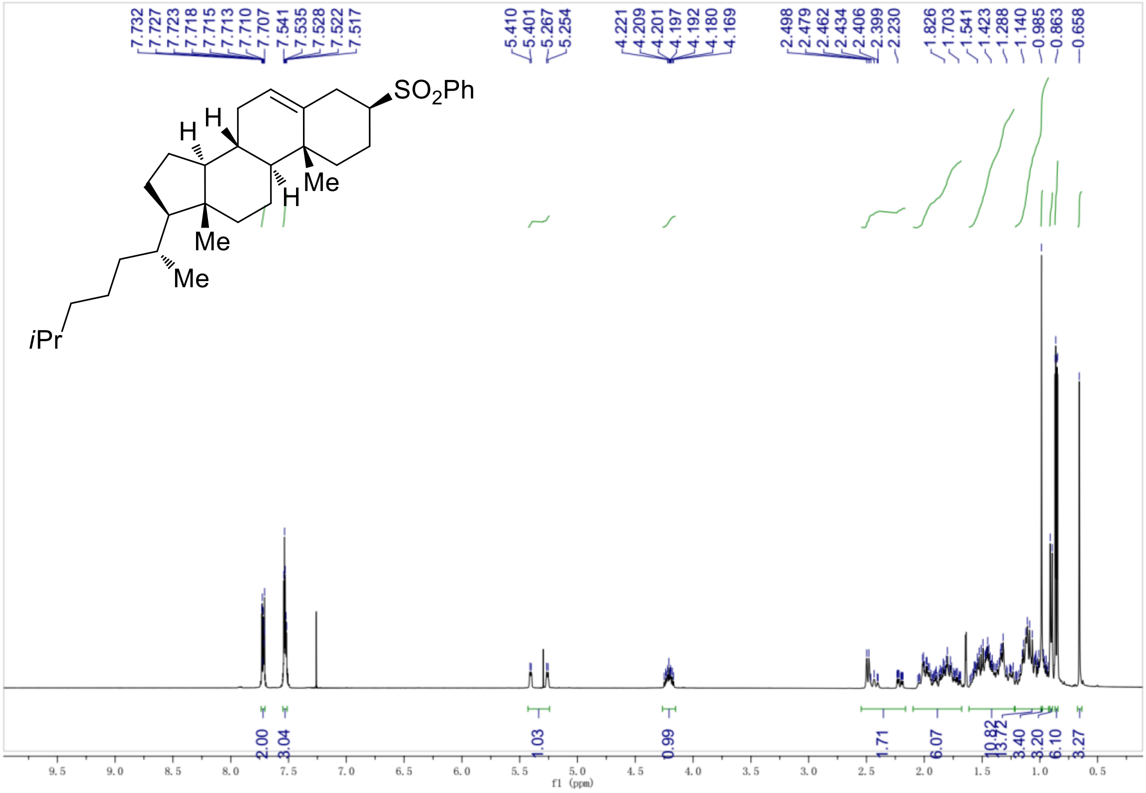

^{1}H NMR spectrum of compound **34a** in $CDCl_3$ (400 MHz).

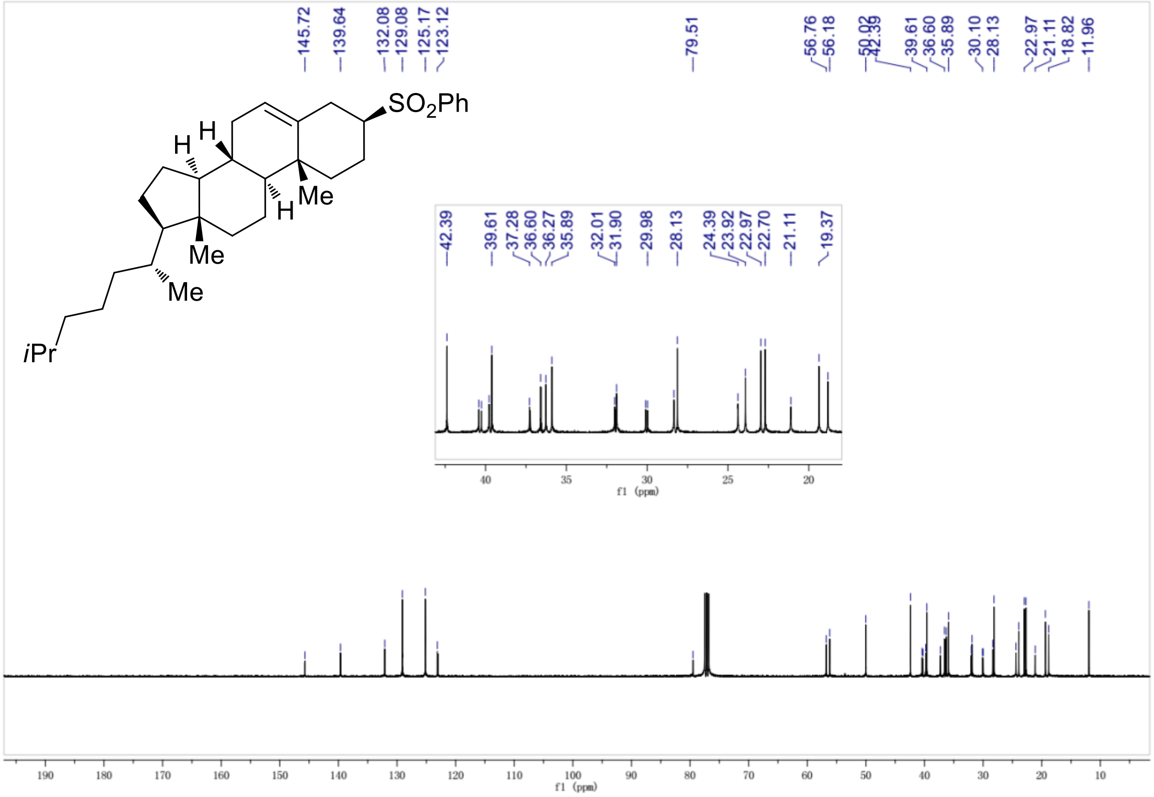

^{13}C{^{1}H} NMR spectrum of compound **34a** in $CDCl_3$ (100 MHz).

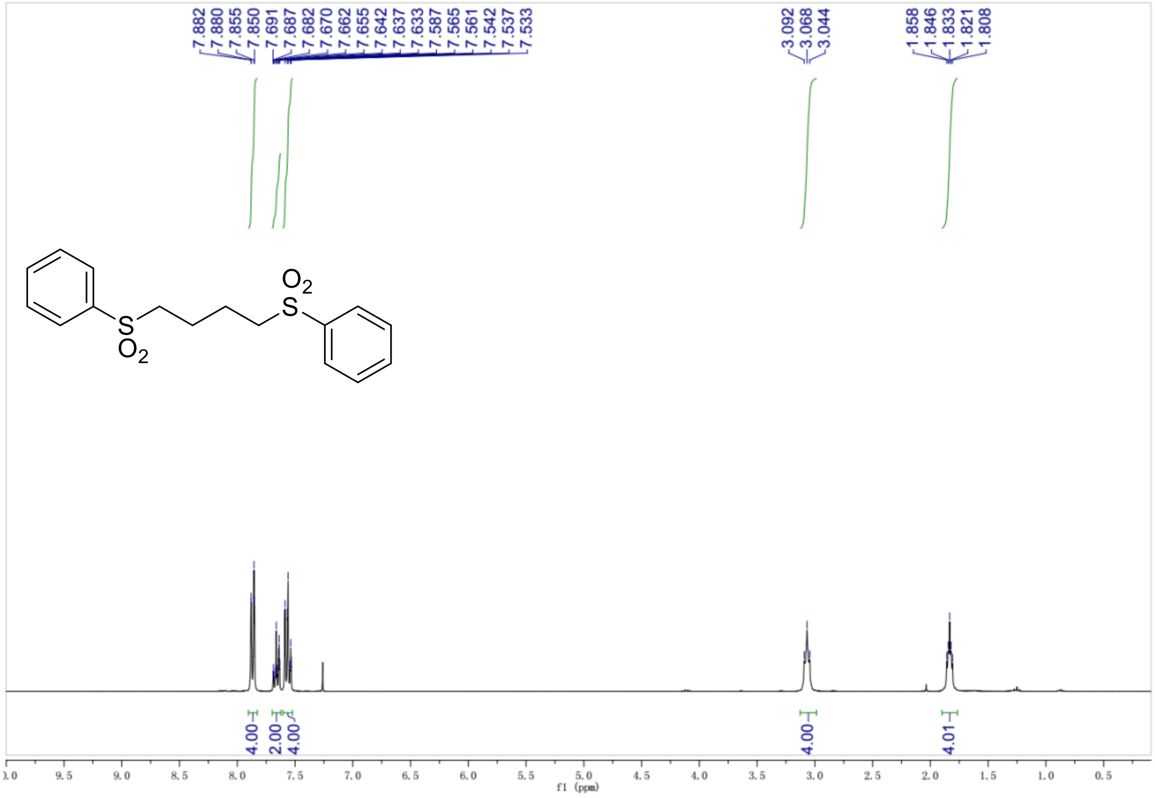

^{1}H NMR spectrum of compound **35a** in $CDCl_3$ (300 MHz).

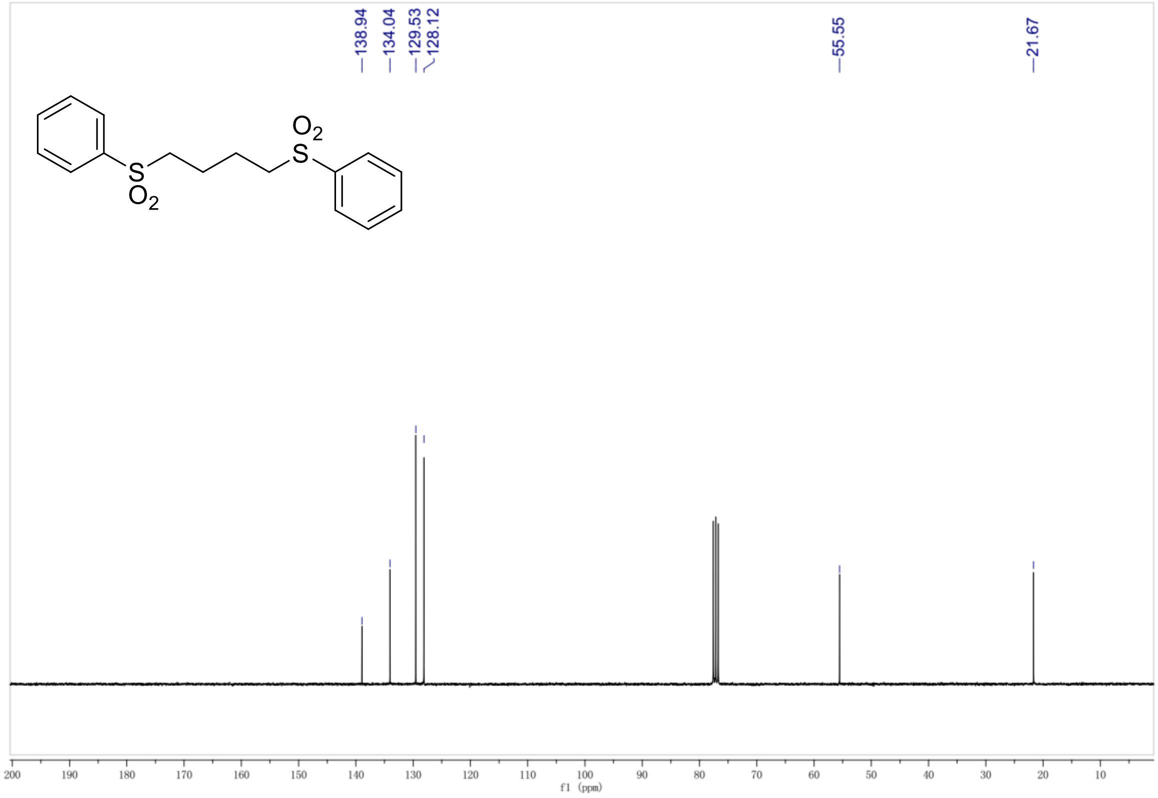

$^{13}C\{^{1}H\}$ NMR spectrum of compound **35a** in $CDCl_3$ (75 MHz).

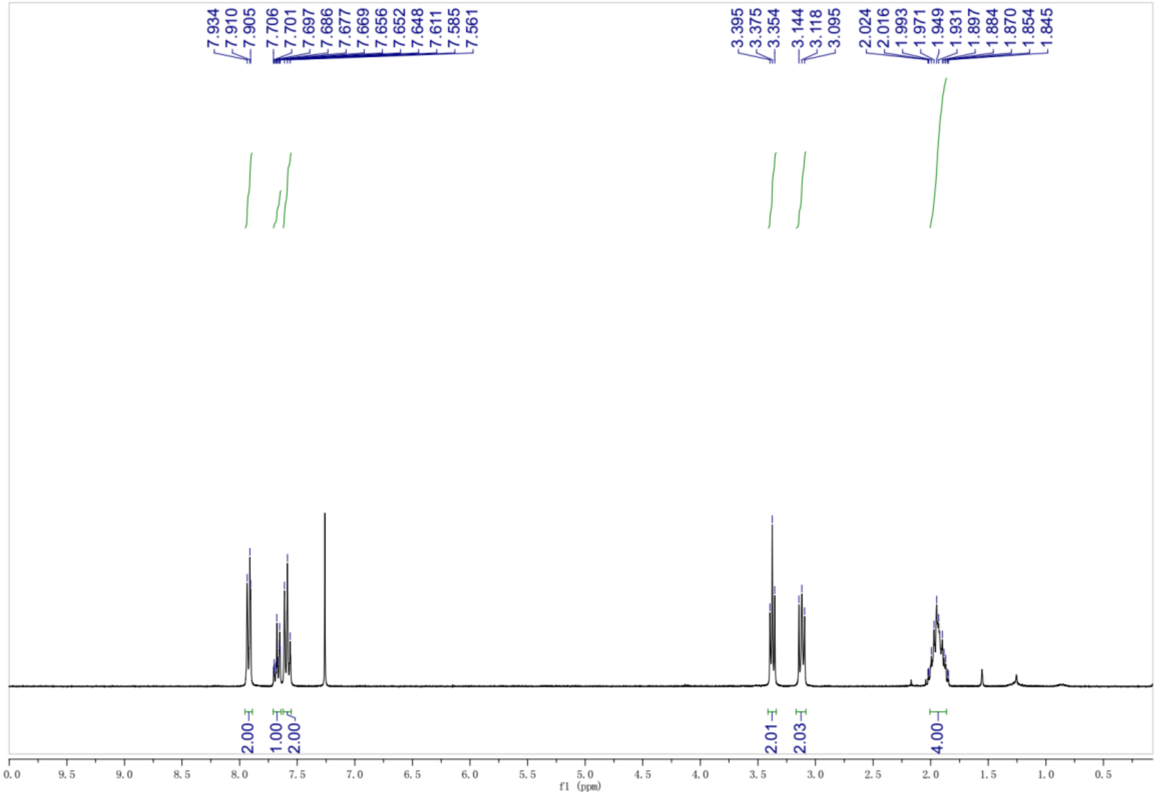

^{1}H NMR spectrum of compound **36a** in $CDCl_3$ (300 MHz).

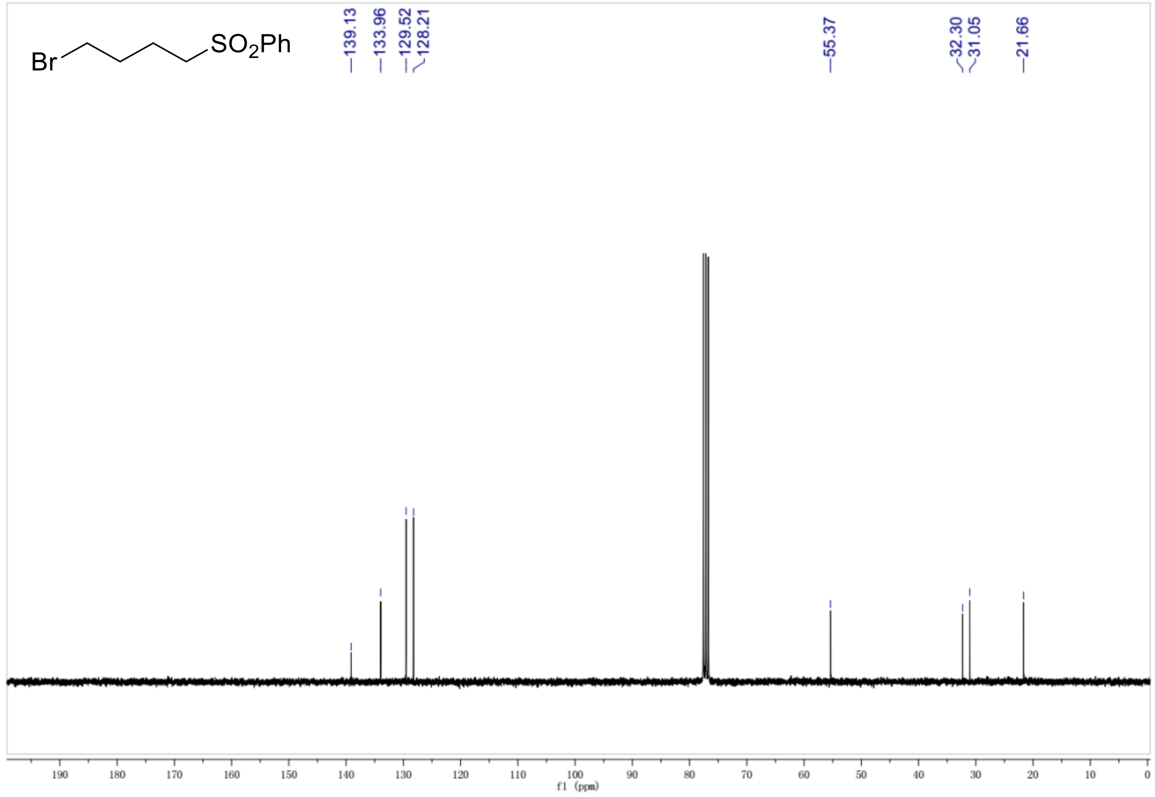

^{13}C{^{1}H} NMR spectrum of compound **36a** in $CDCl_3$ (75 MHz).

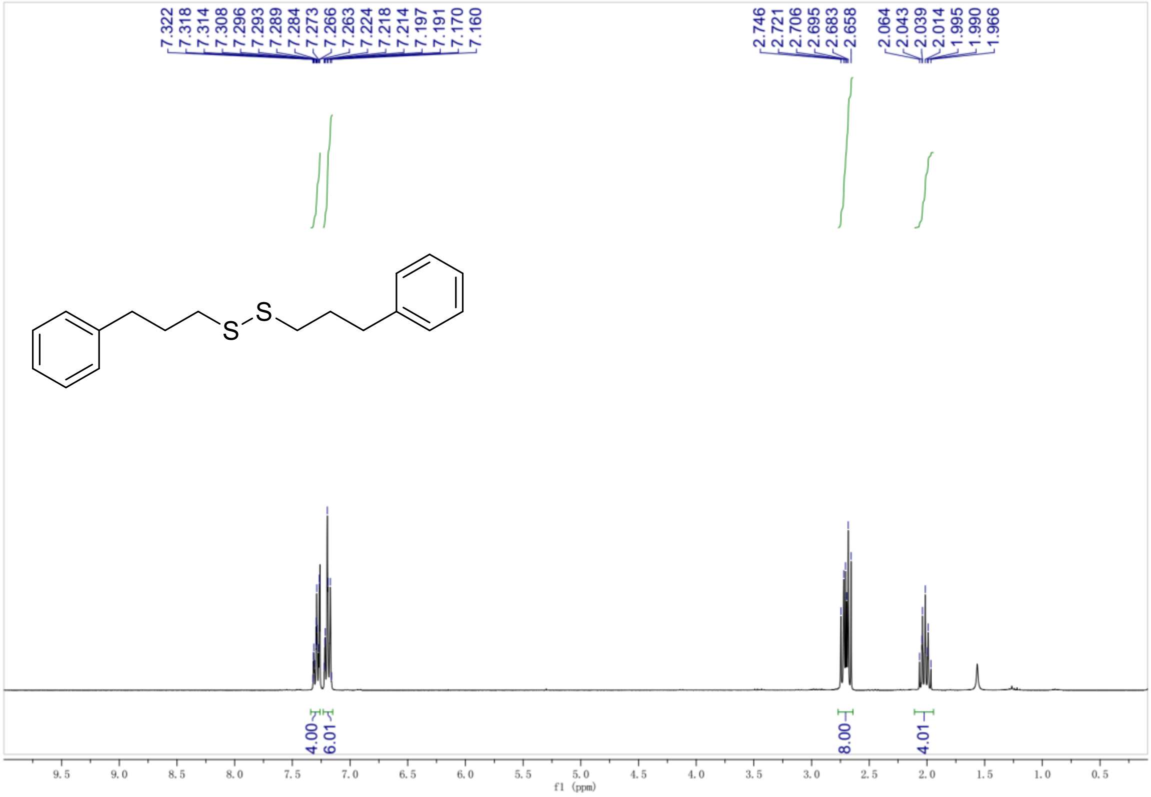

^{1}H NMR spectrum of **1,2-bis(3-phenylpropyl)disulfane** in $CDCl_3$ (300 MHz).

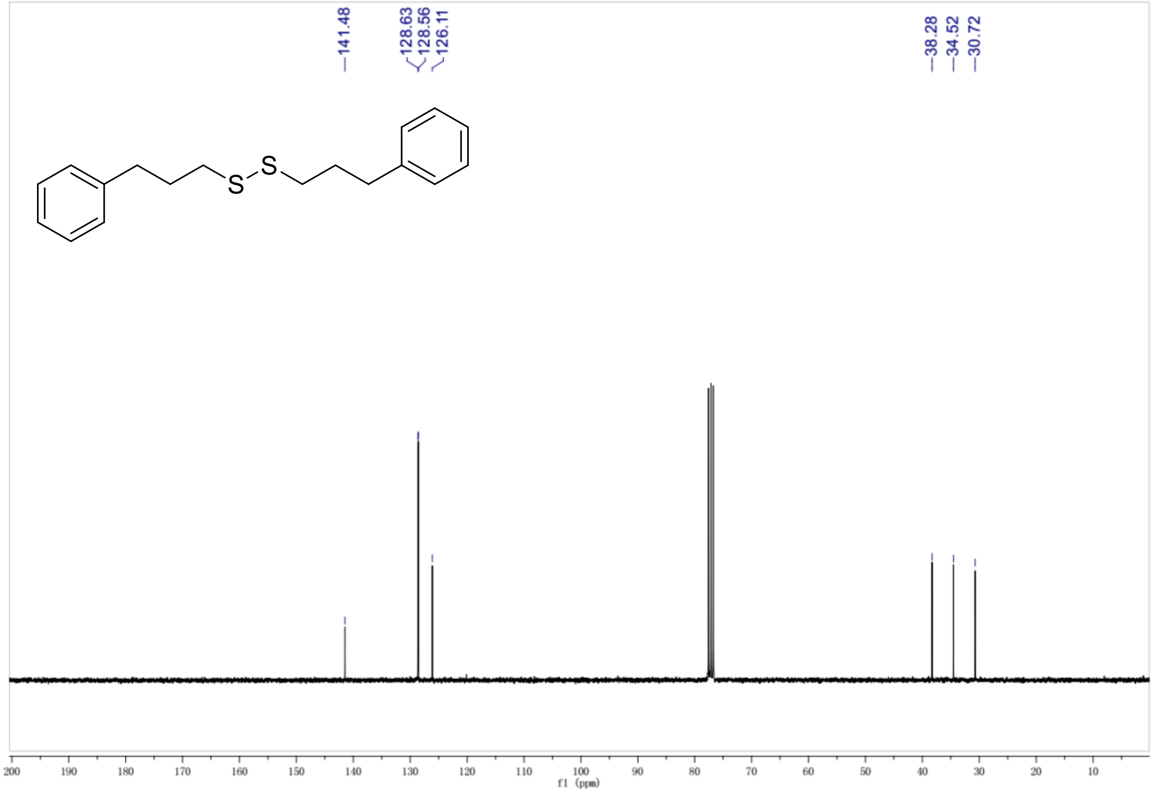

$^{13}C\{^{1}H\}$ NMR spectrum of **1,2-bis(3-phenylpropyl)disulfane** in $CDCl_3$ (75 MHz).

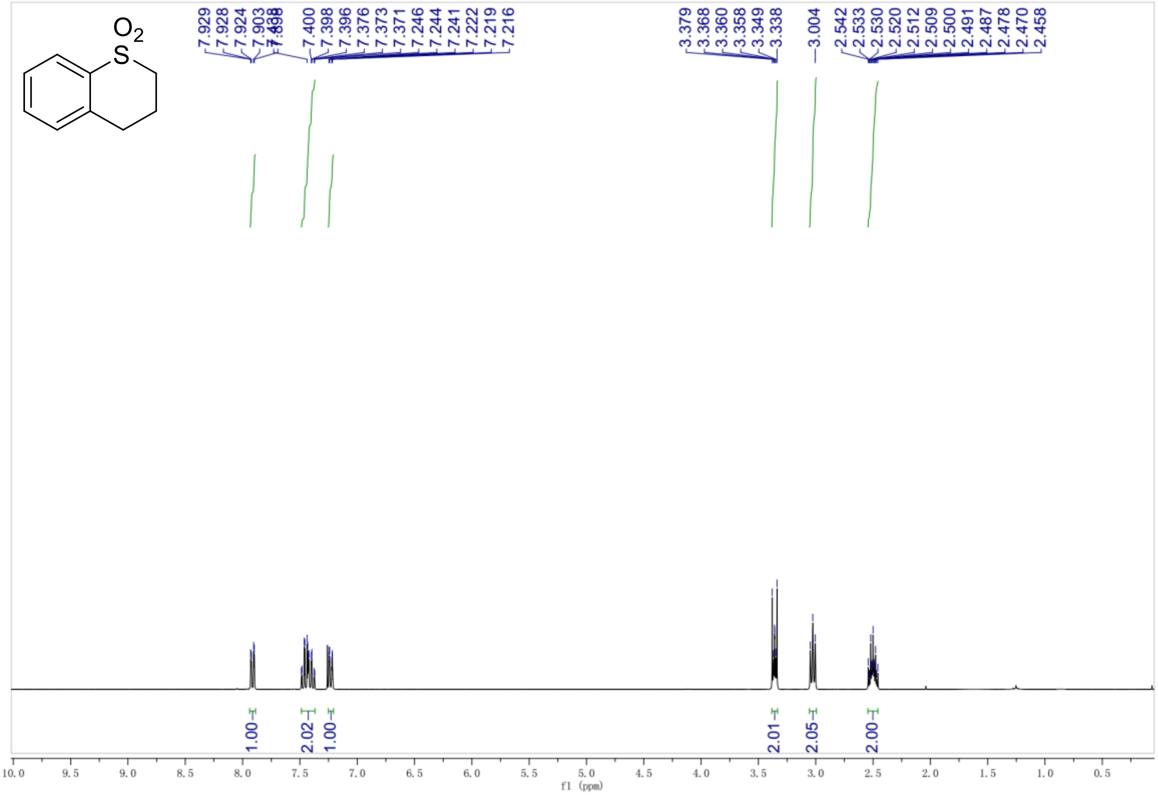

^{1}H NMR spectrum of compound **37a** in $CDCl_3$ (300 MHz).

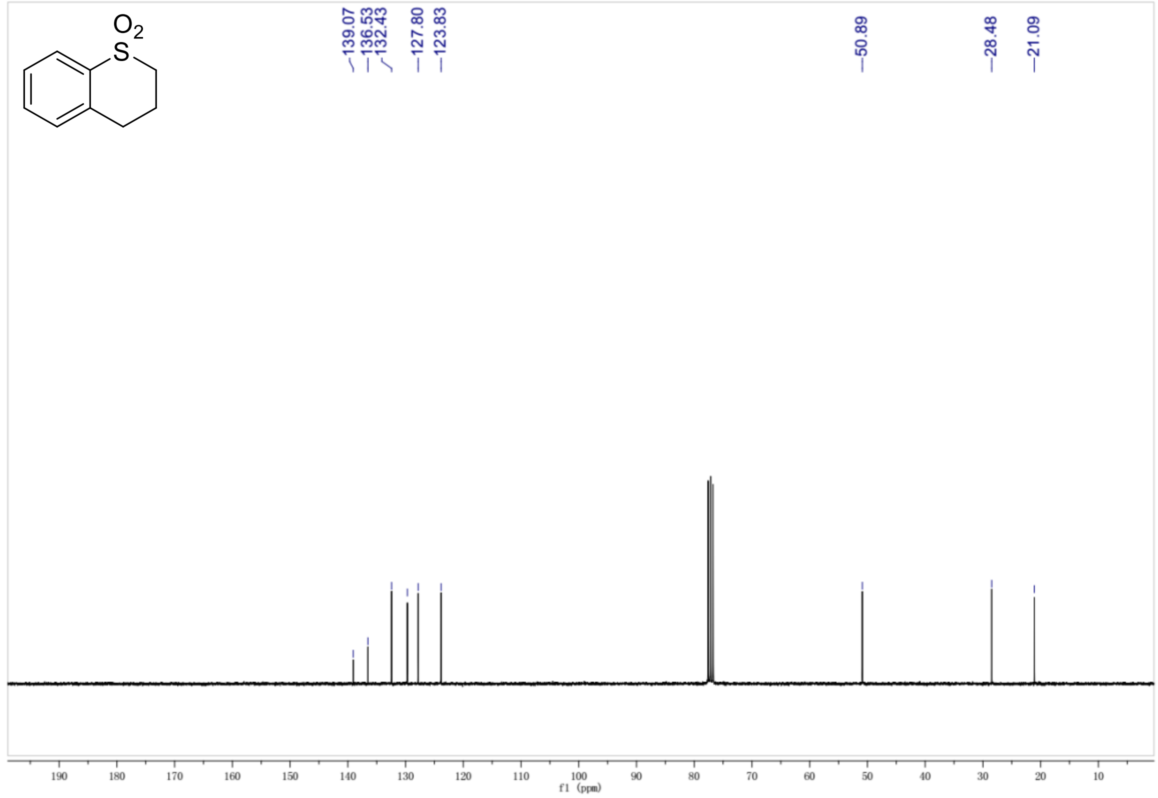

$^{13}C\{^{1}H\}$ NMR spectrum of compound **37a** in $CDCl_3$ (75 MHz).

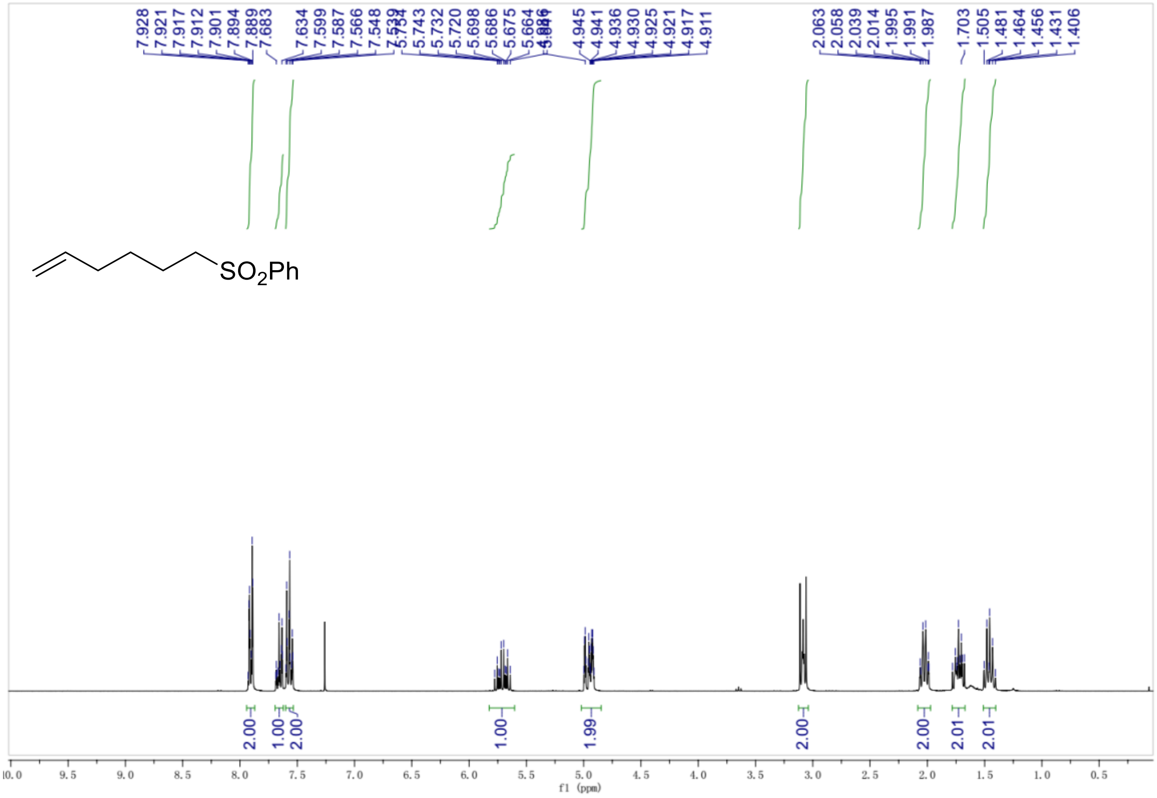

^{1}H NMR spectrum of compound **44a** in $CDCl_3$ (300 MHz).

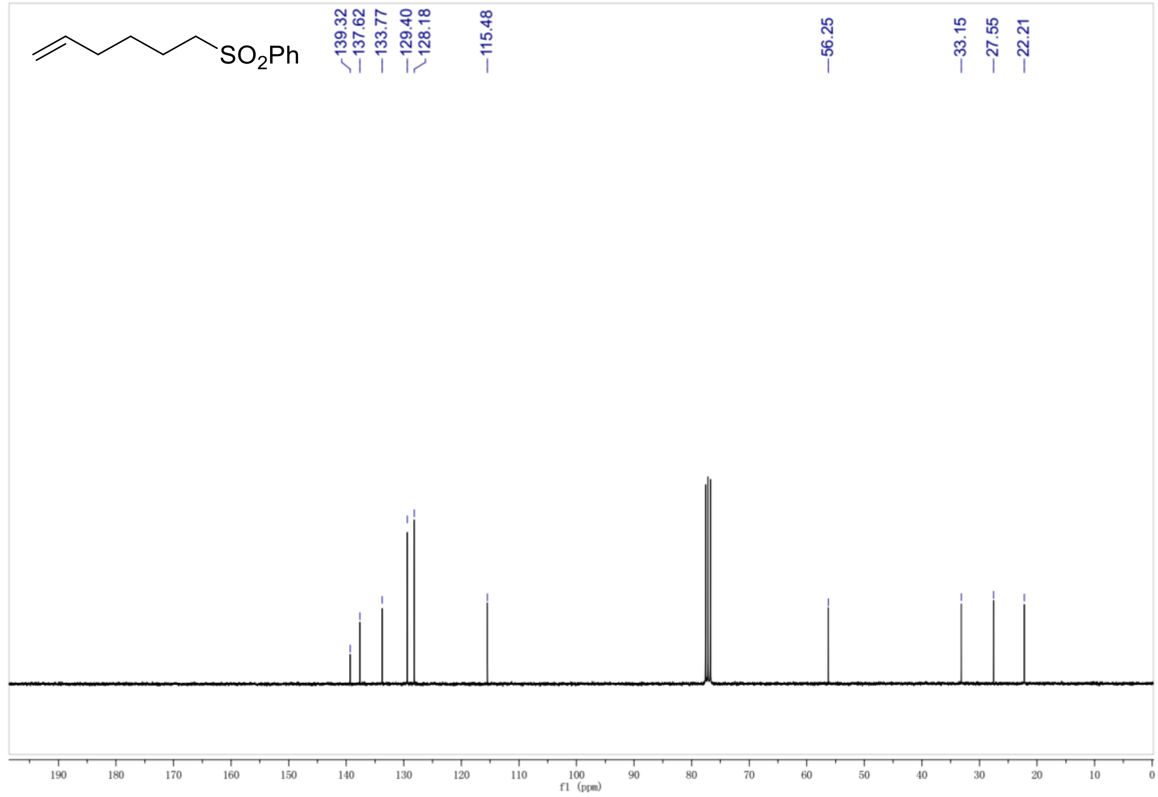

$^{13}C\{^{1}H\}$ NMR spectrum of compound **44a** in $CDCl_3$ (75 MHz).

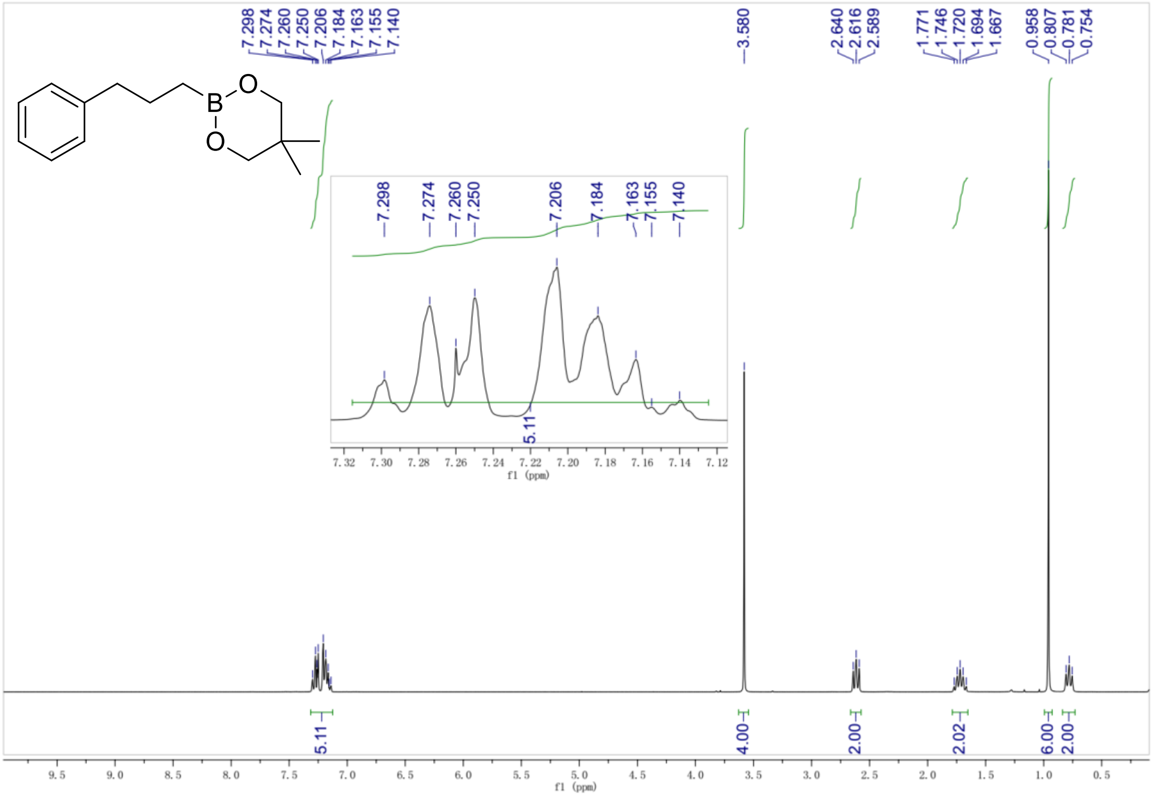

^{1}H NMR spectrum of compound **1b** in $CDCl_3$ (300 MHz).

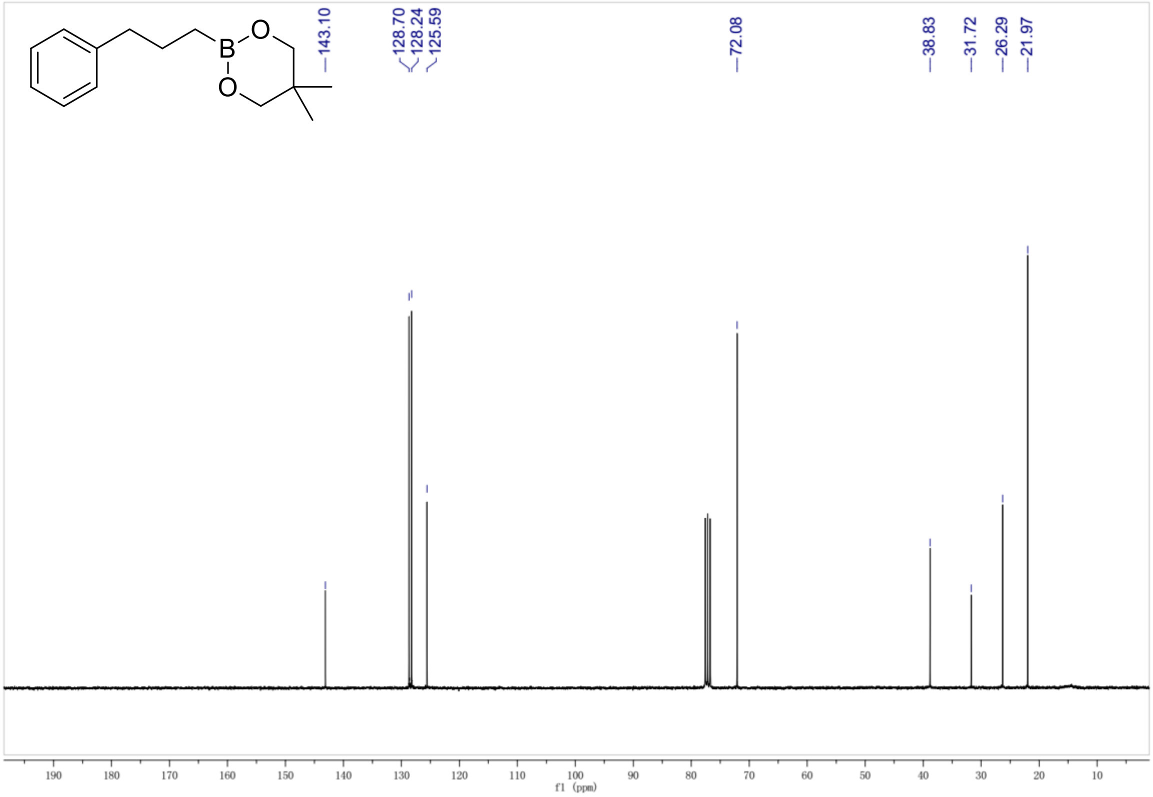

^{13}C{^{1}H} NMR spectrum of compound **1b** in $CDCl_3$ (75 MHz).

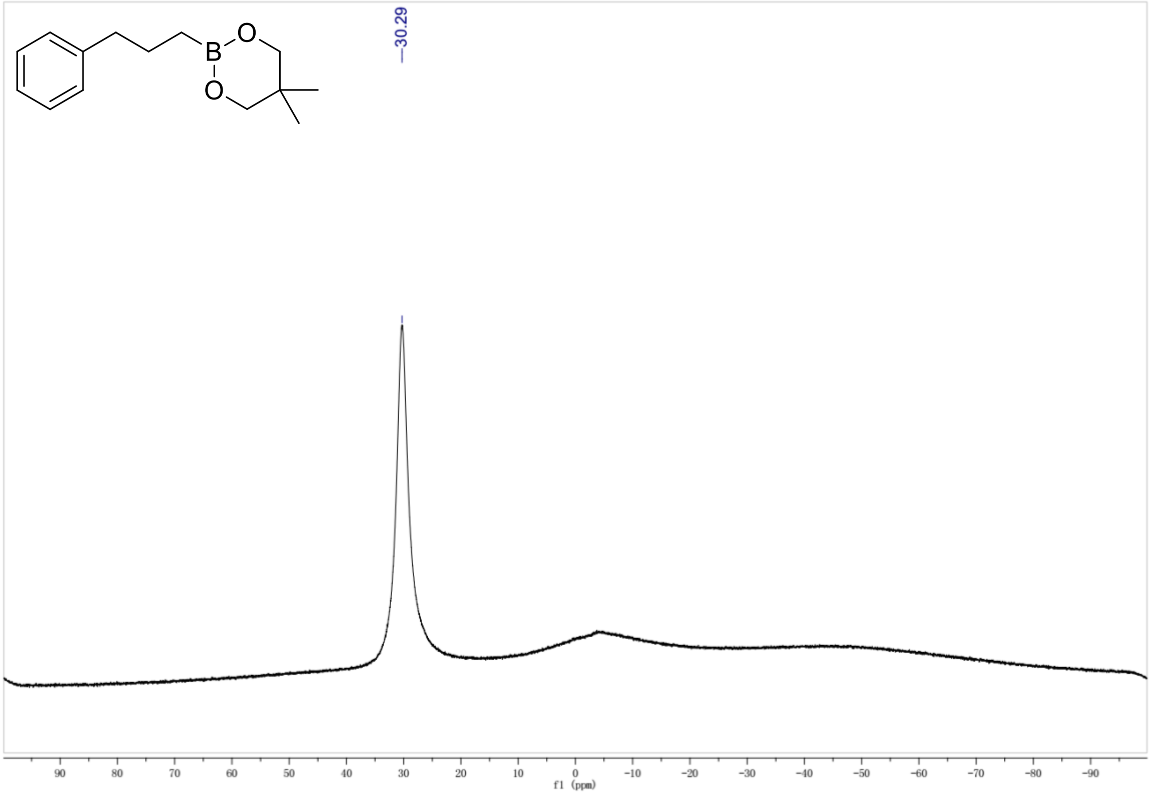

$^{11}B\{^{1}H\}$ NMR spectrum of compound **1b** in $CDCl_3$ (96 MHz).

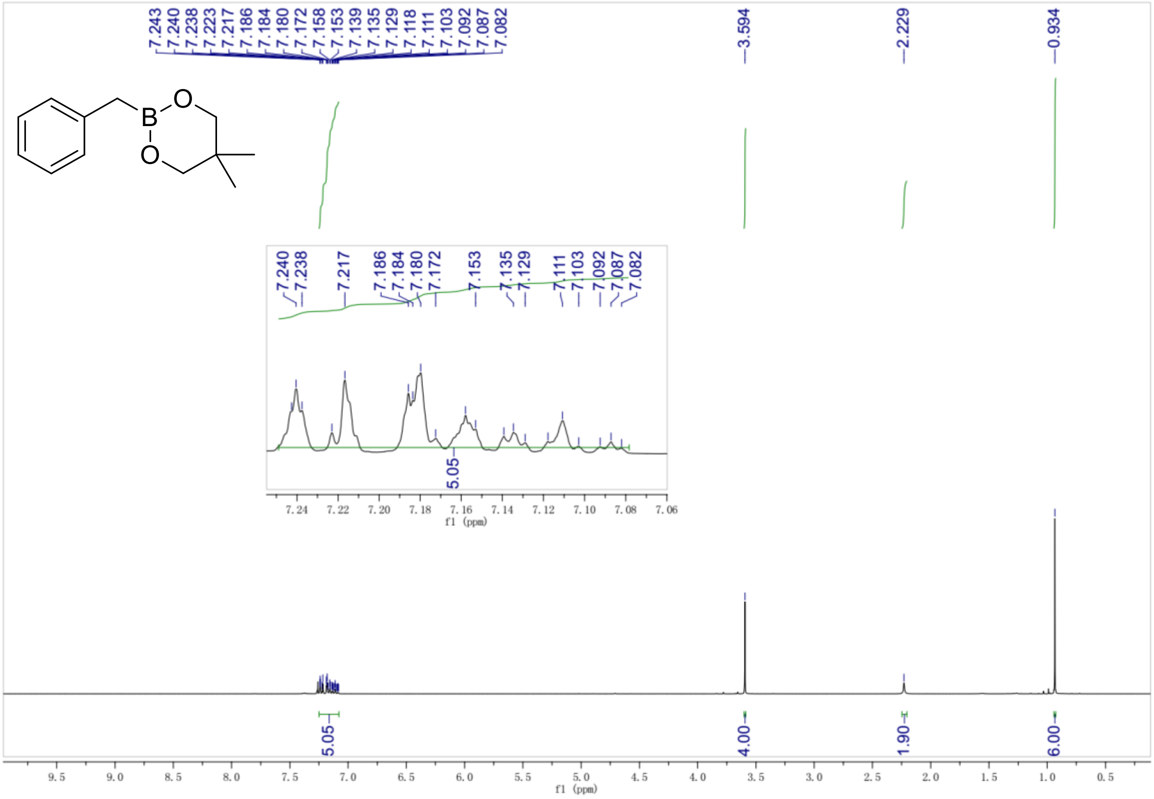

^{1}H NMR spectrum of compound **2b** in $CDCl_3$ (300 MHz).

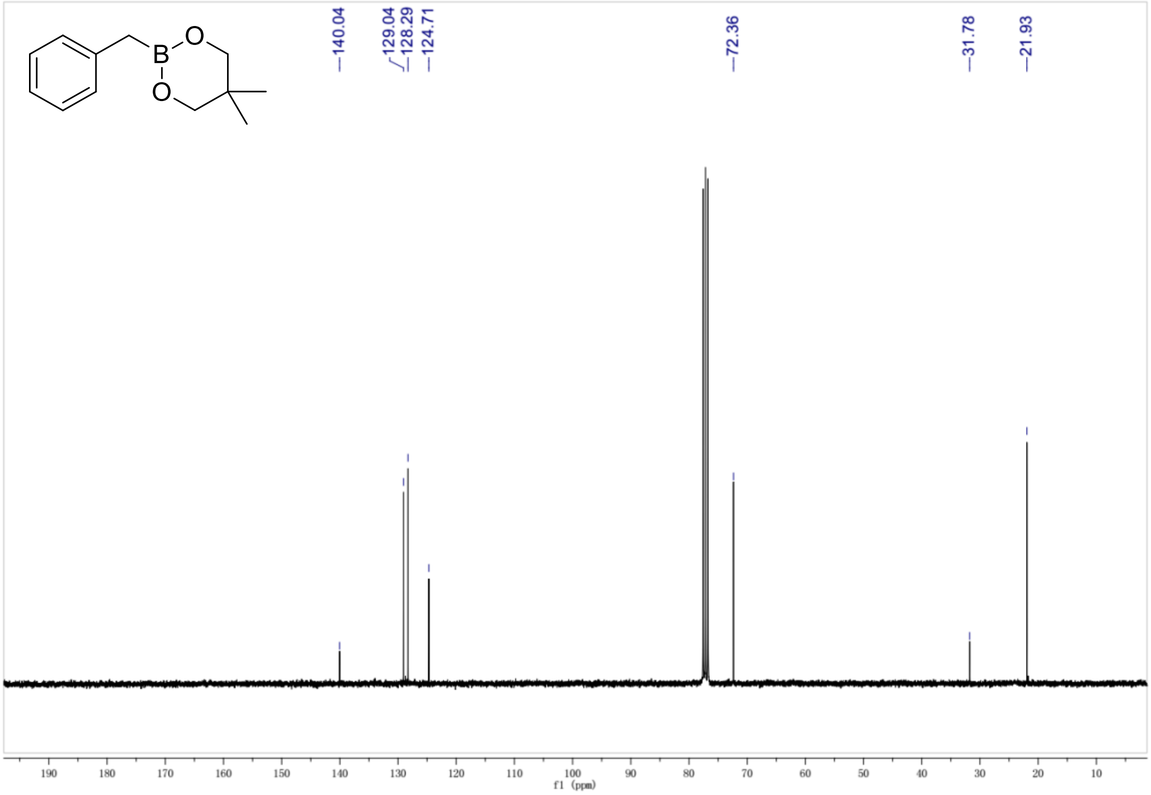

$^{13}C\{^{1}H\}$ NMR spectrum of compound **2b** in $CDCl_3$ (75 MHz).

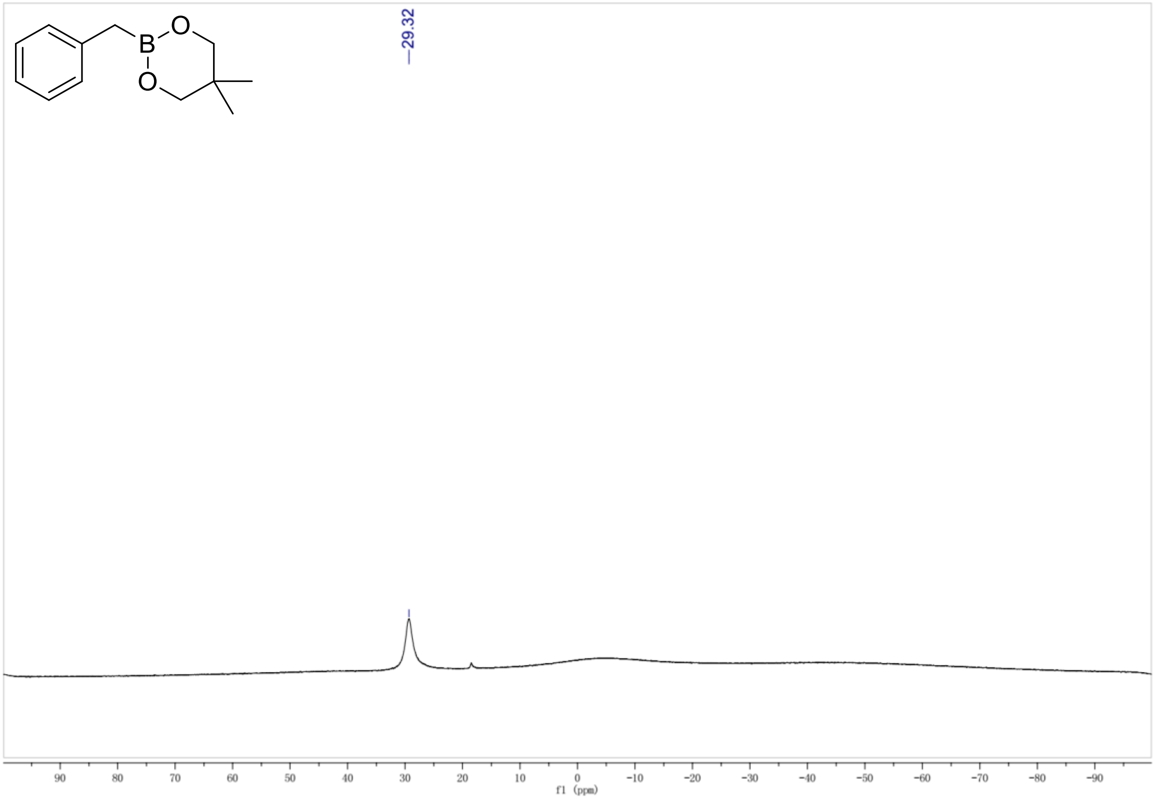

$^{11}B\{^{1}H\}$ NMR spectrum of compound **2b** in $CDCl_3$ (96 MHz).

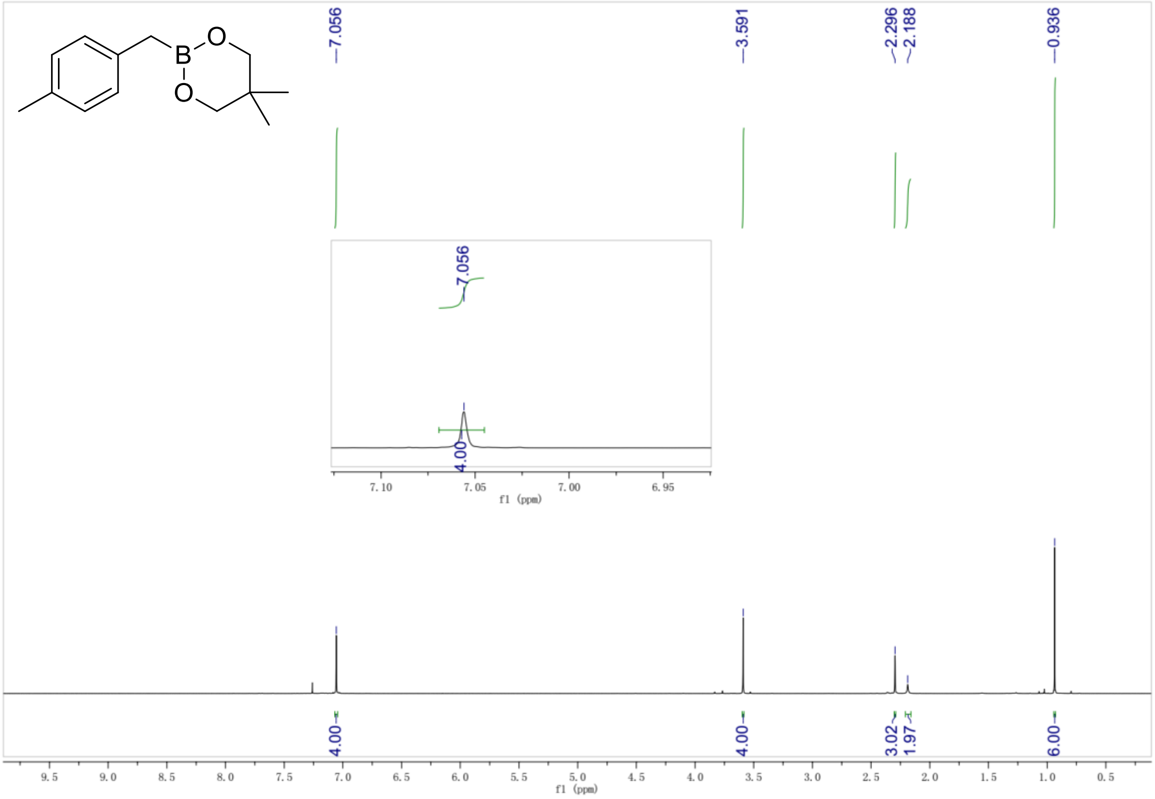

^{1}H NMR spectrum of compound **3b** in $CDCl_3$ (300 MHz).

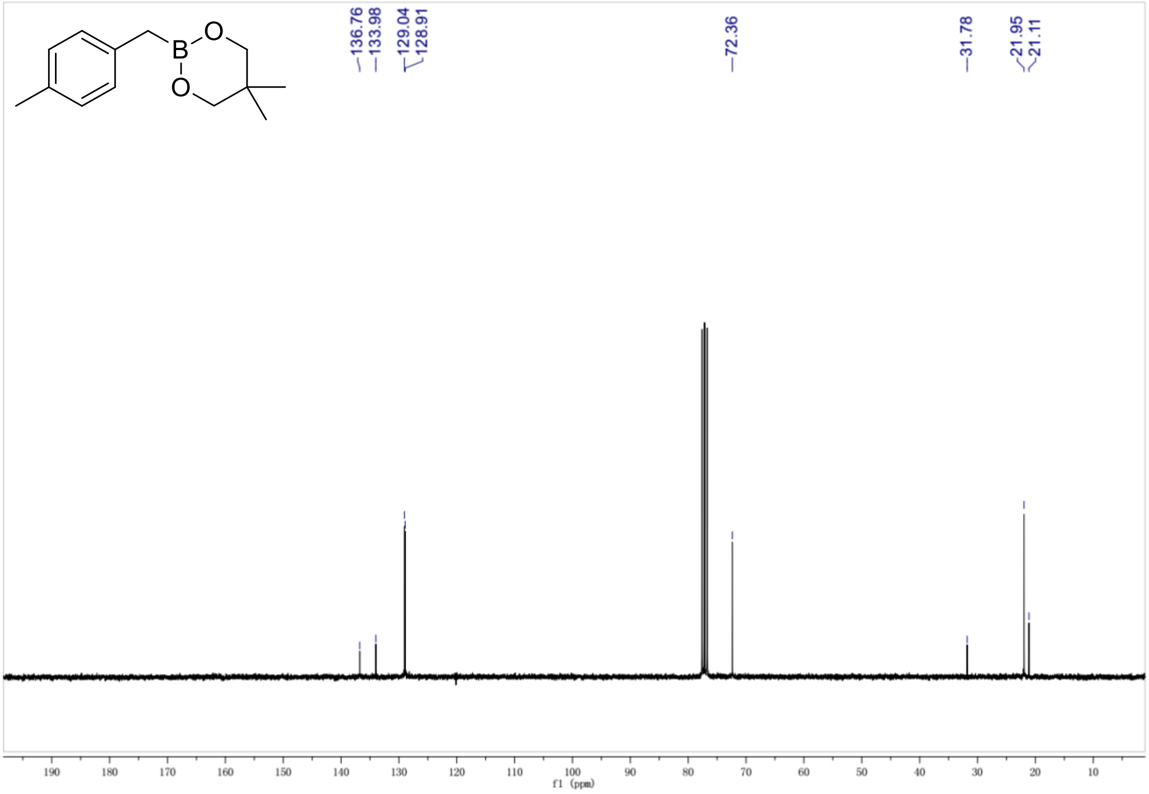

^{13}C{^{1}H} NMR spectrum of compound **3b** in $CDCl_3$ (75 MHz).

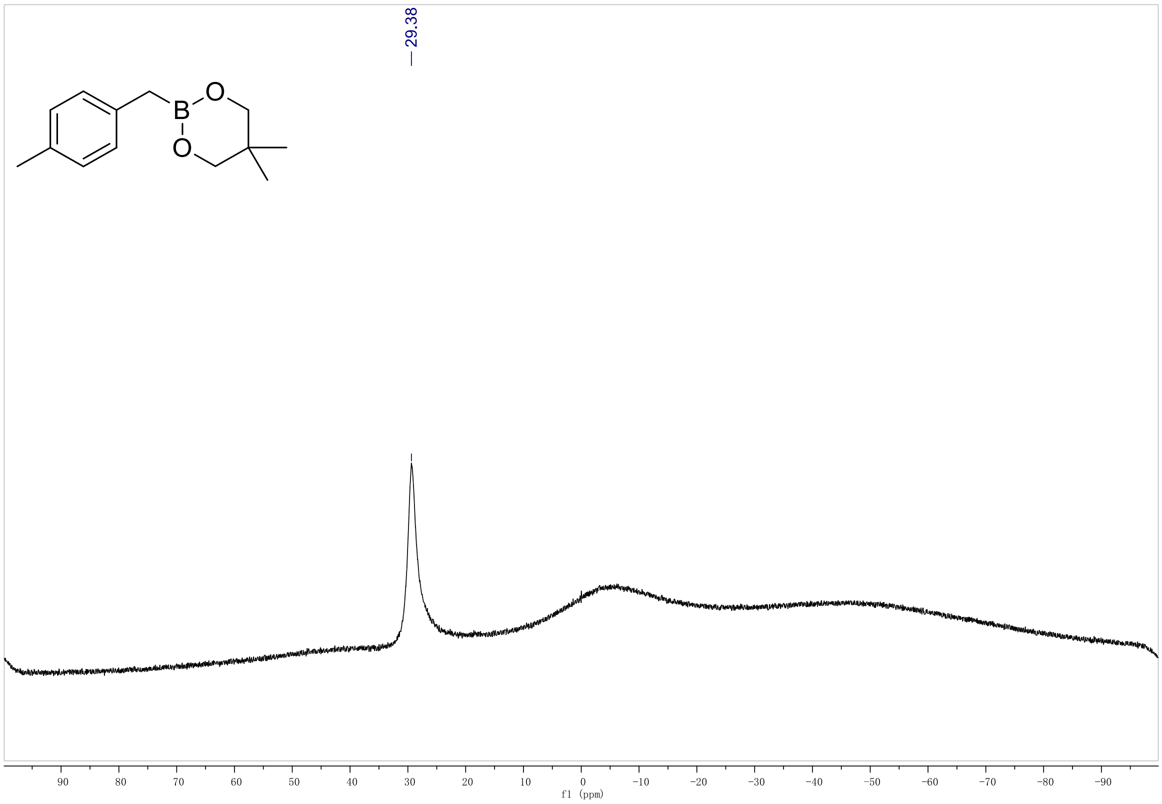

$^{11}B\{^{1}H\}$ NMR spectrum of compound **3b** in $CDCl_3$ (96 MHz).

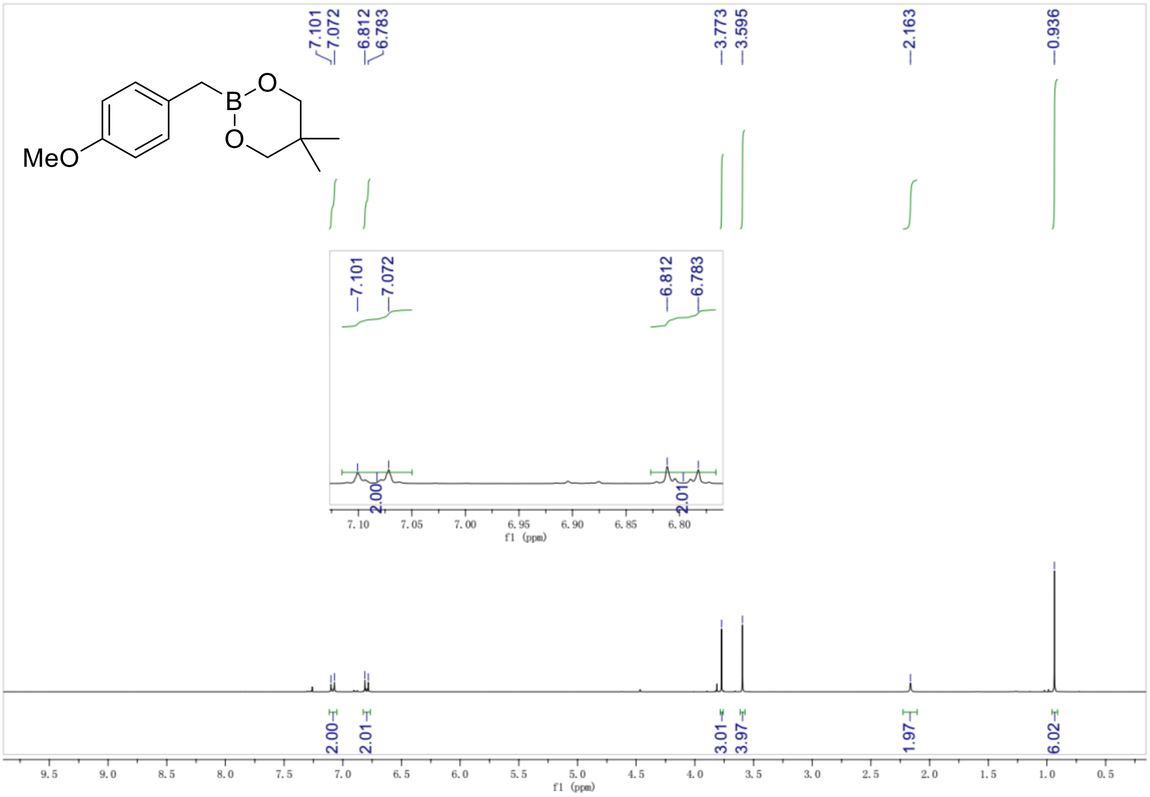

^{1}H NMR spectrum of compound **4b** in $CDCl_3$ (300 MHz).

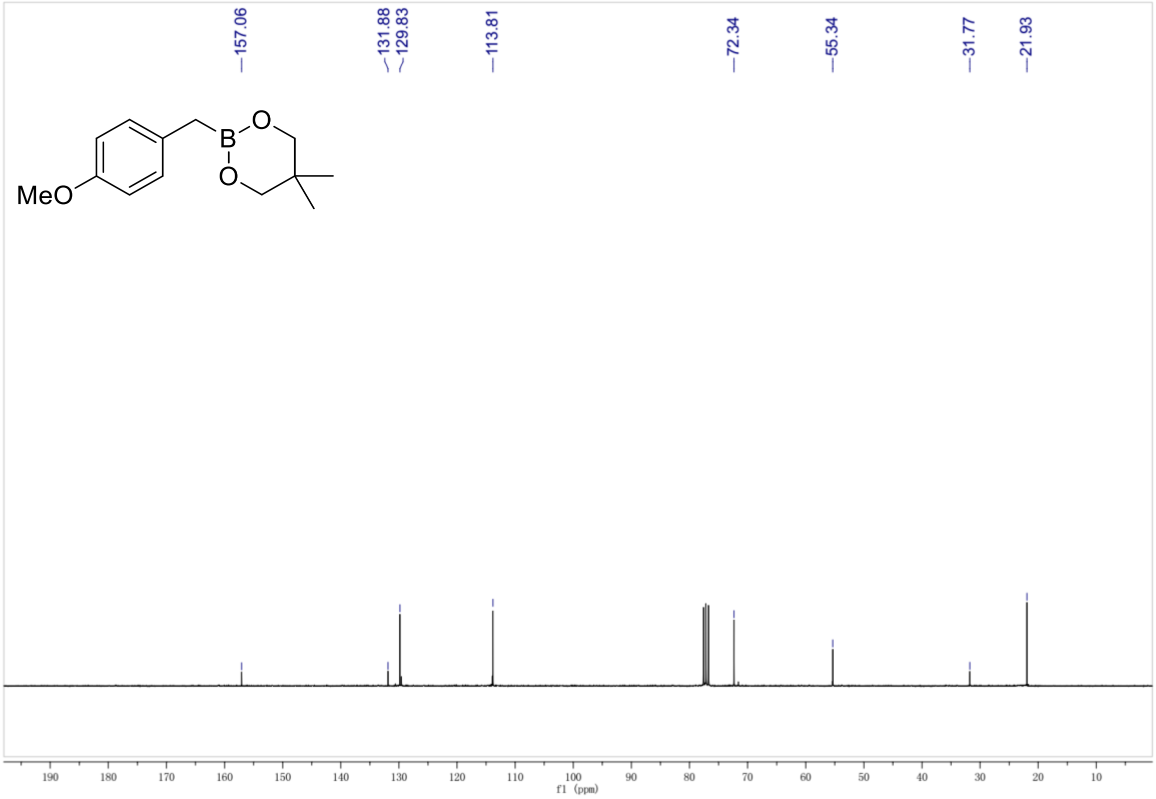

$^{13}C\{^{1}H\}$ NMR spectrum of compound **4b** in $CDCl_3$ (75 MHz).

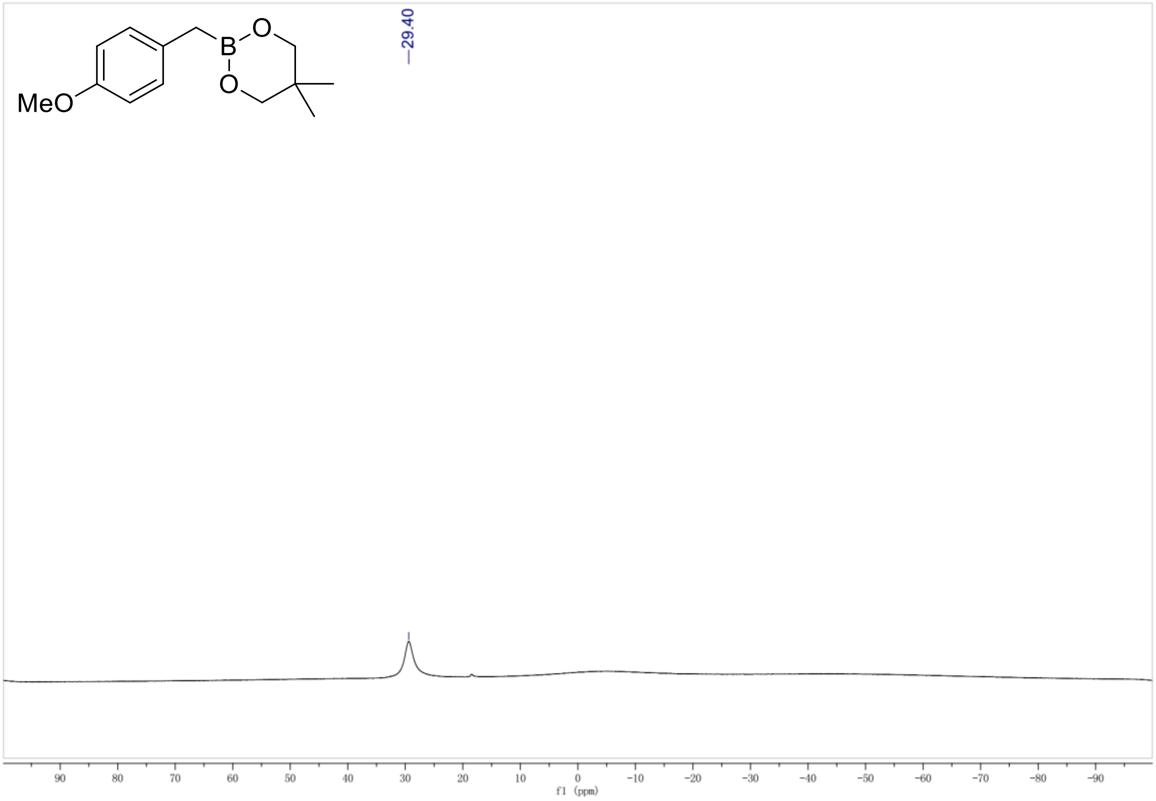

$^{11}B\{^{1}H\}$ NMR spectrum of compound **4b** in $CDCl_3$ (96 MHz).

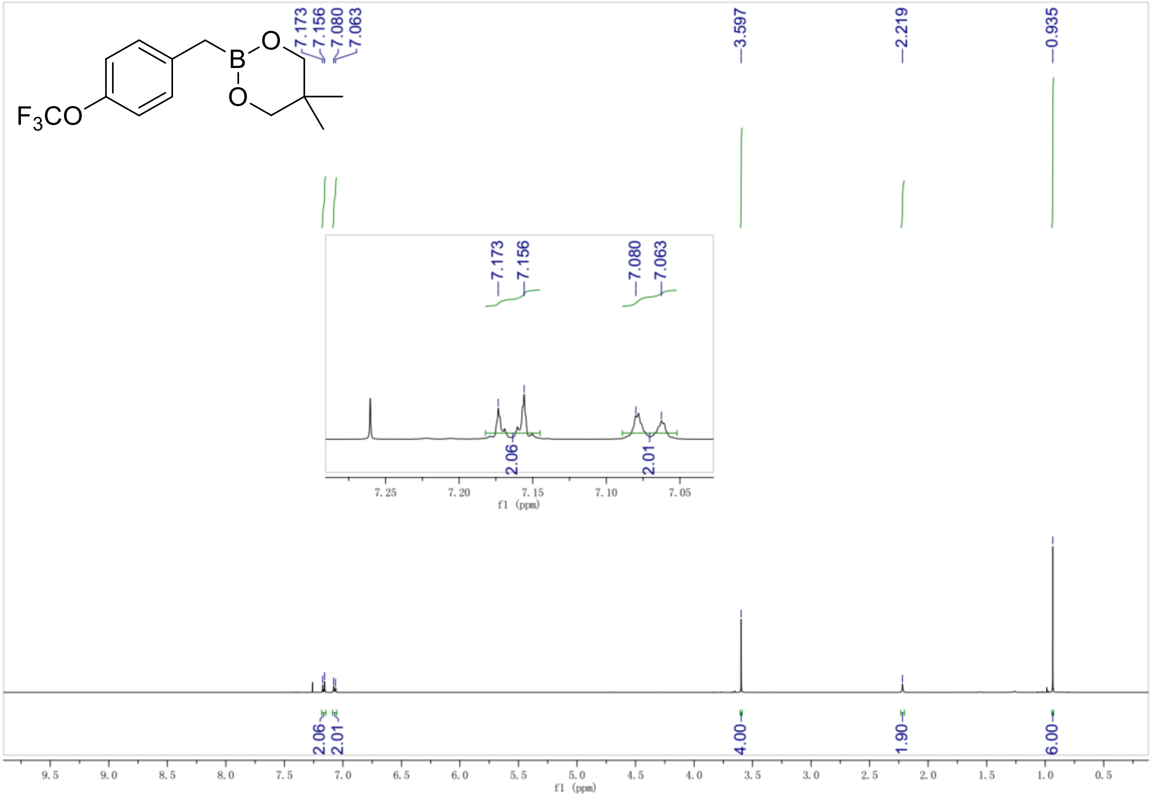

^{1}H NMR spectrum of compound **5b** in $CDCl_3$ (500 MHz).

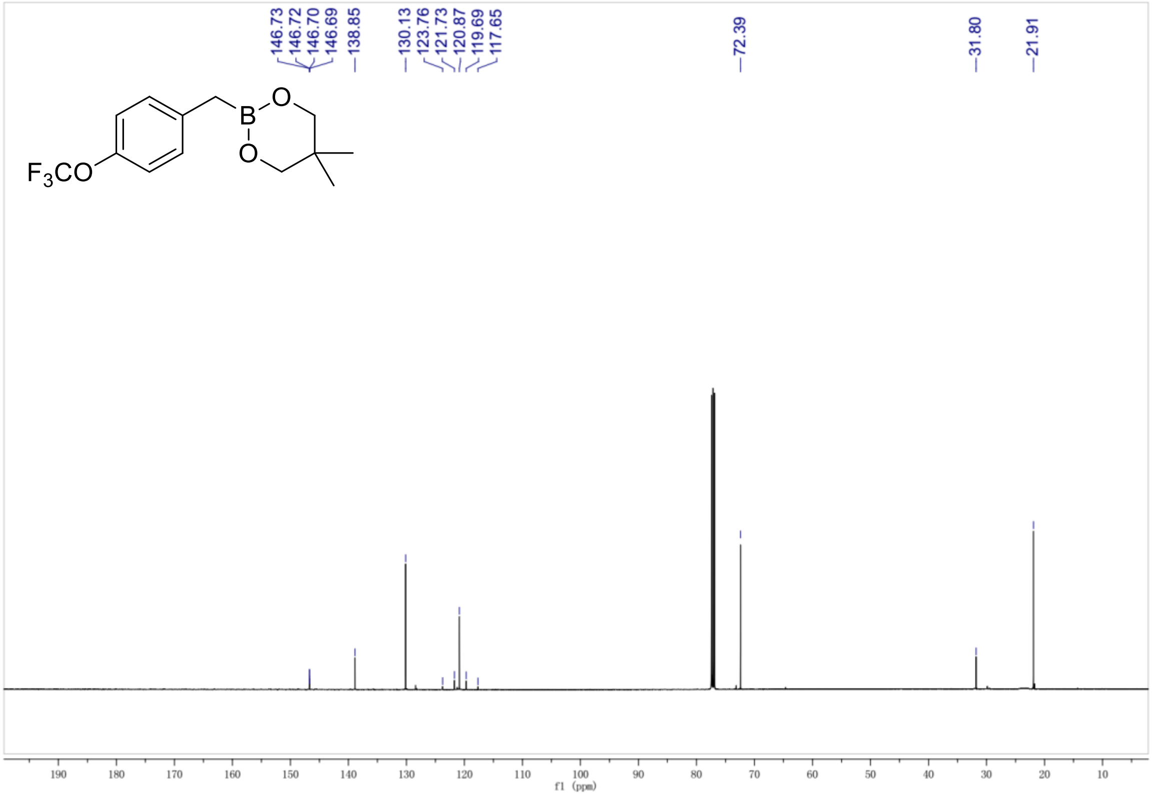

$^{13}C\{^{1}H\}$ NMR spectrum of compound **5b** in $CDCl_3$ (125 MHz).

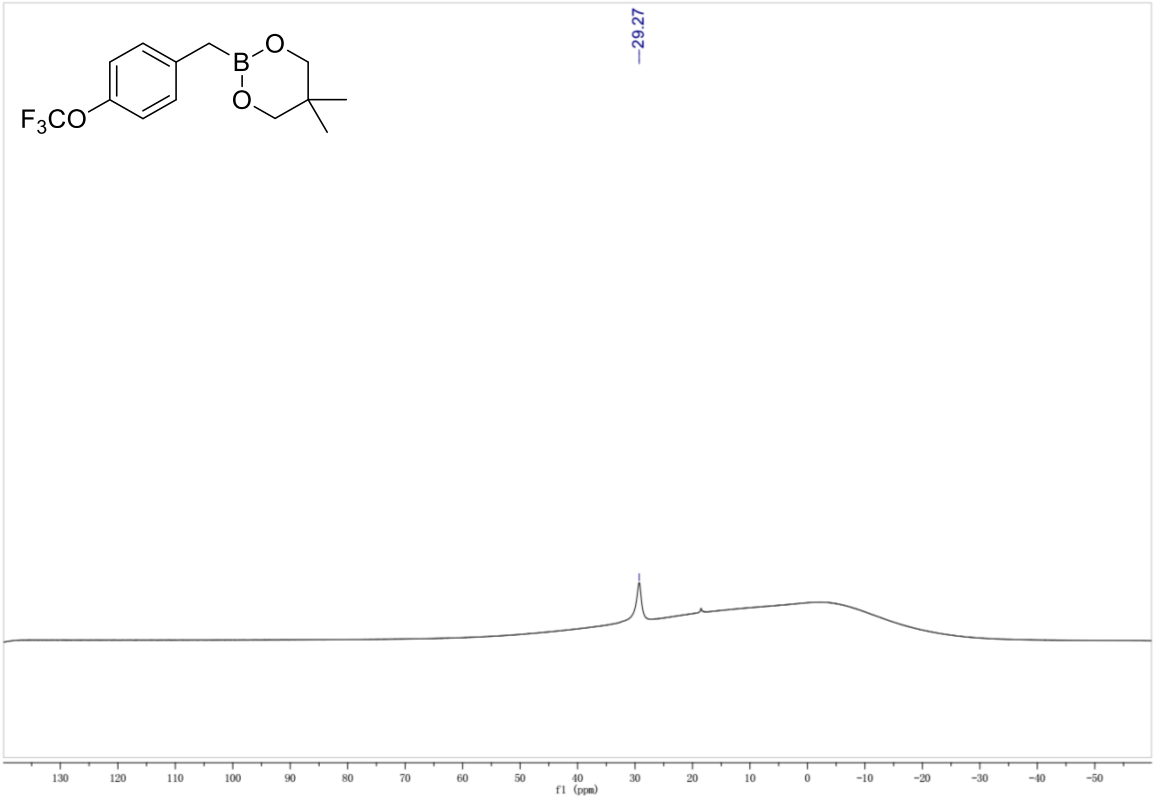

$^{11}B\{^1H\}$ NMR spectrum of compound **5b** in $CDCl_3$ (160 MHz).

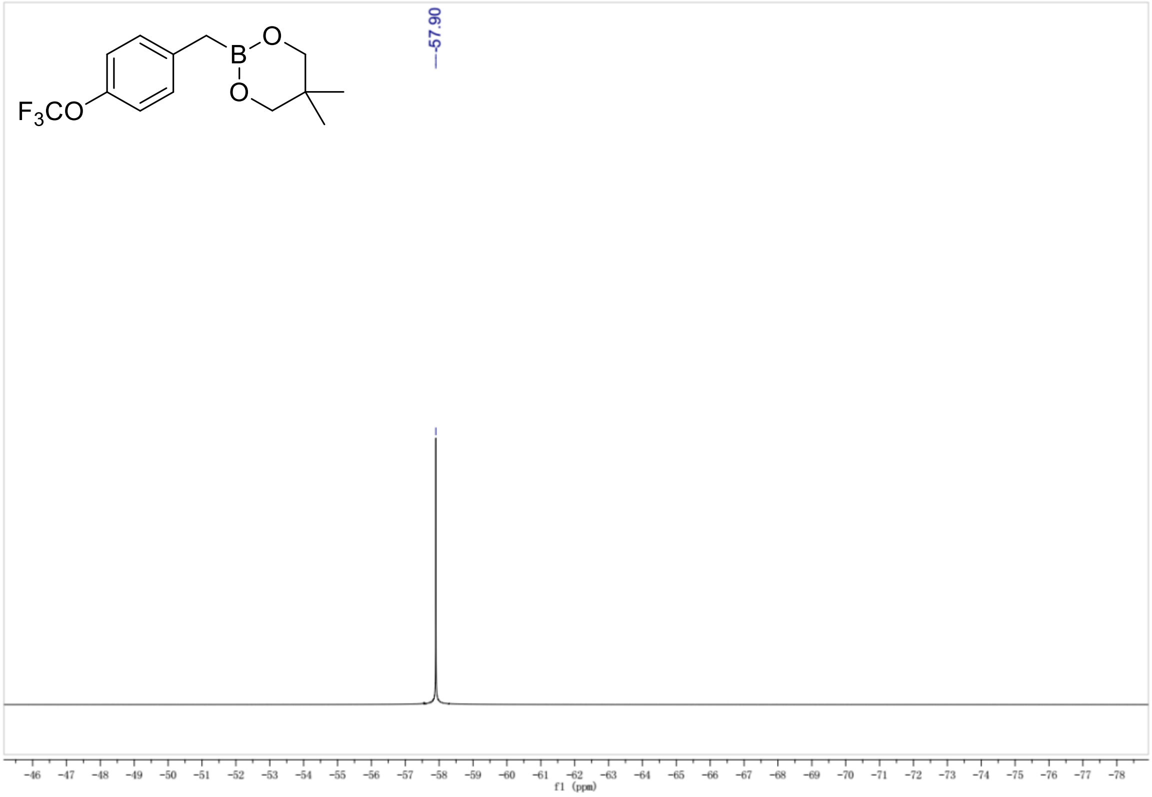

$^{19}F\{^1H\}$ NMR spectrum of compound **5b** in $CDCl_3$ (470 MHz).

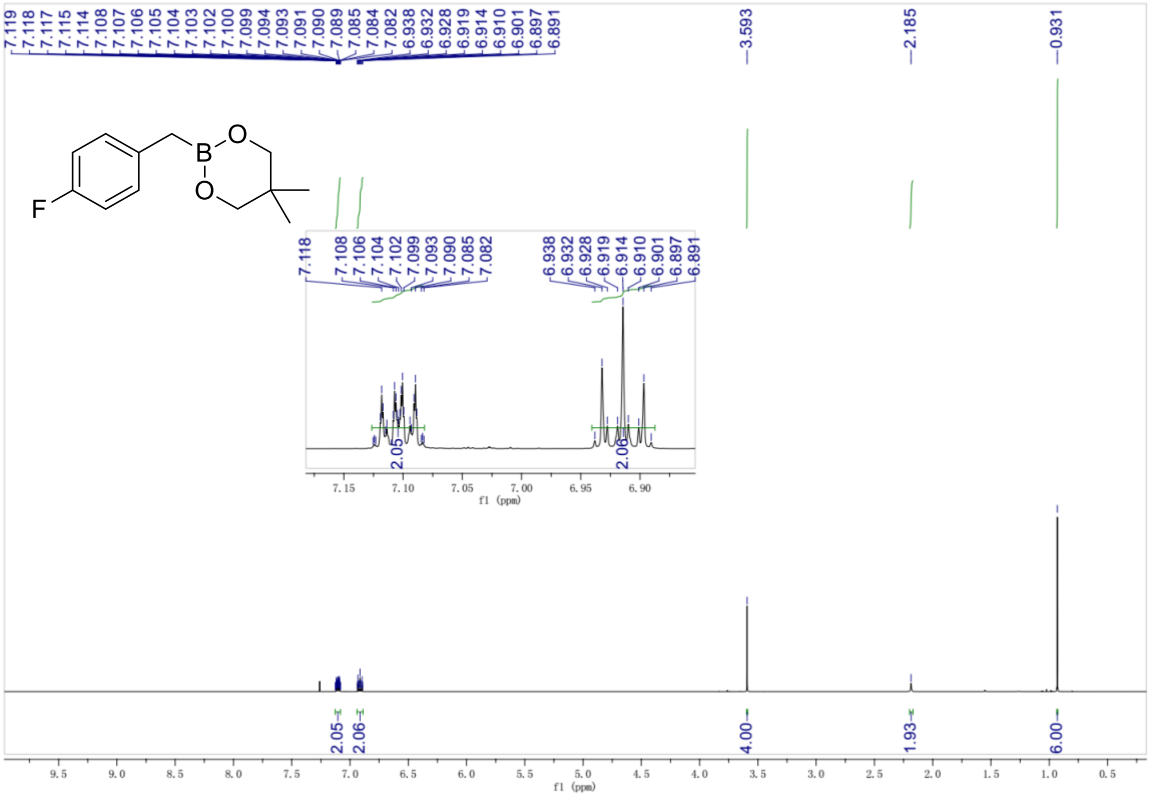

^{1}H NMR spectrum of compound **6b** in $CDCl_3$ (500 MHz).

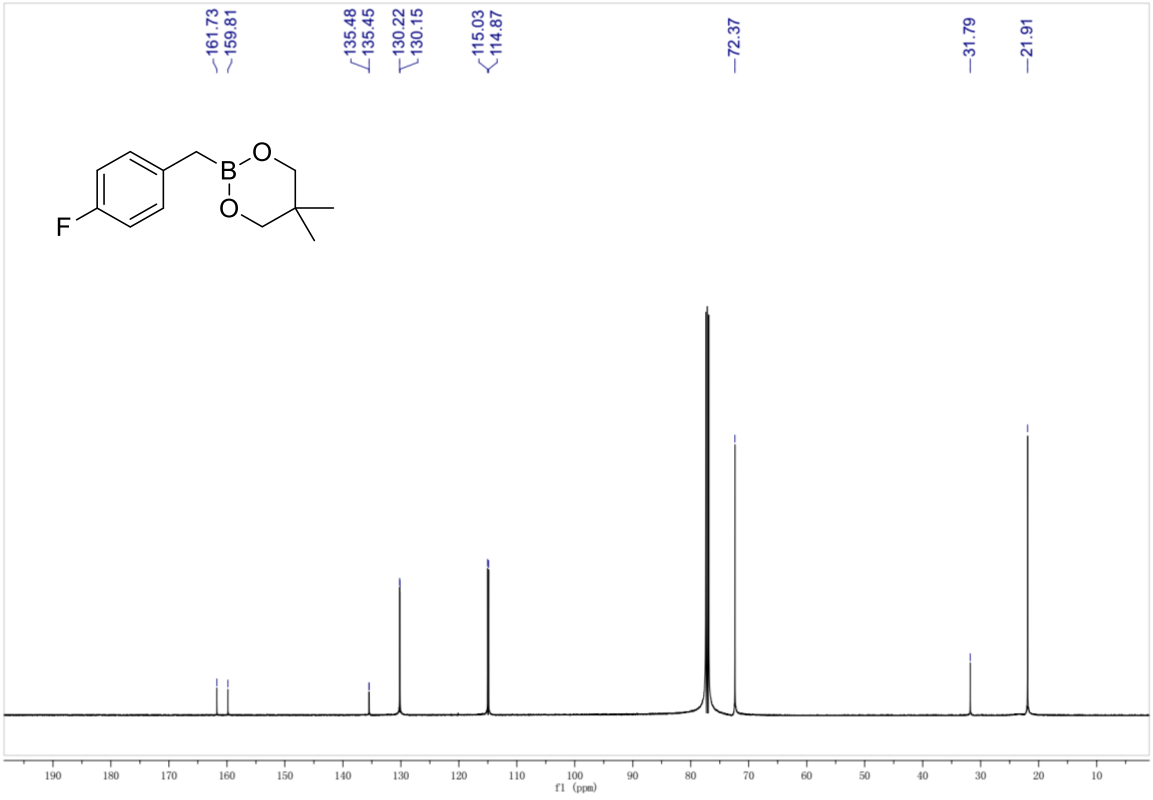

$^{13}C\{^{1}H\}$ NMR spectrum of compound **6b** in $CDCl_3$ (125 MHz).

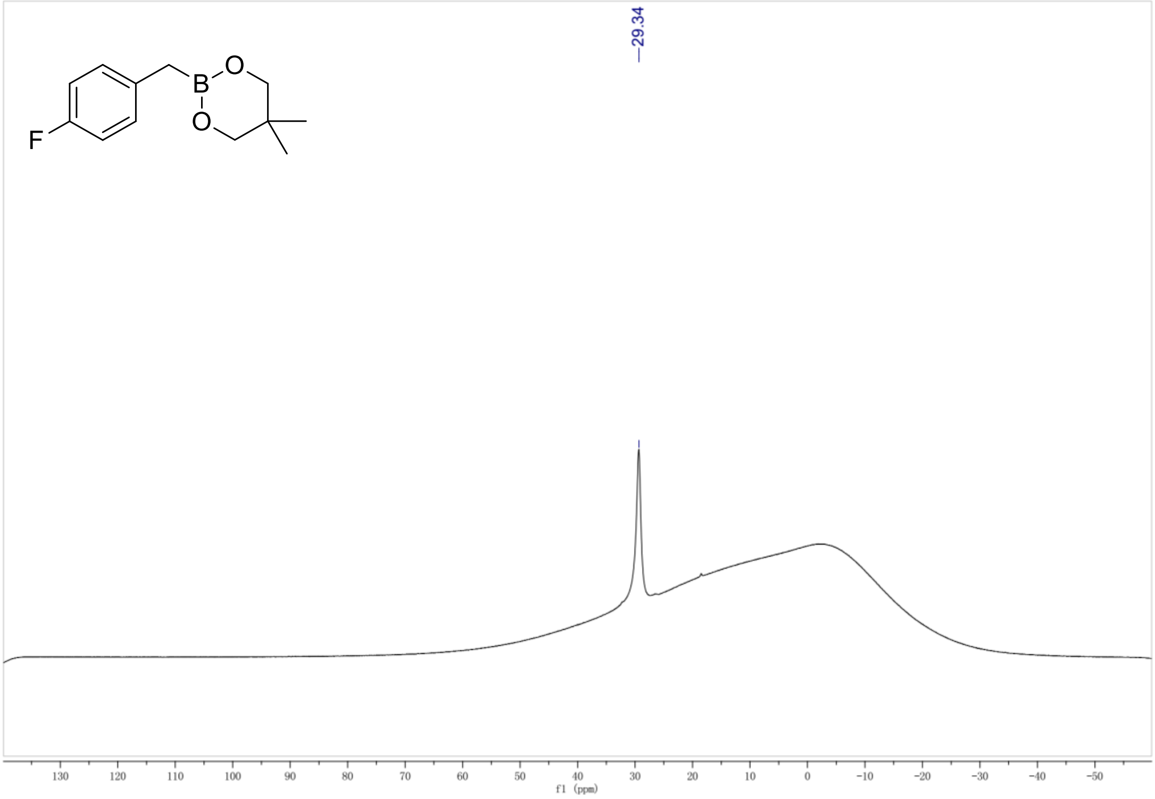

^{11}B{^{1}H} NMR spectrum of compound **6b** in $CDCl_3$ (160 MHz).

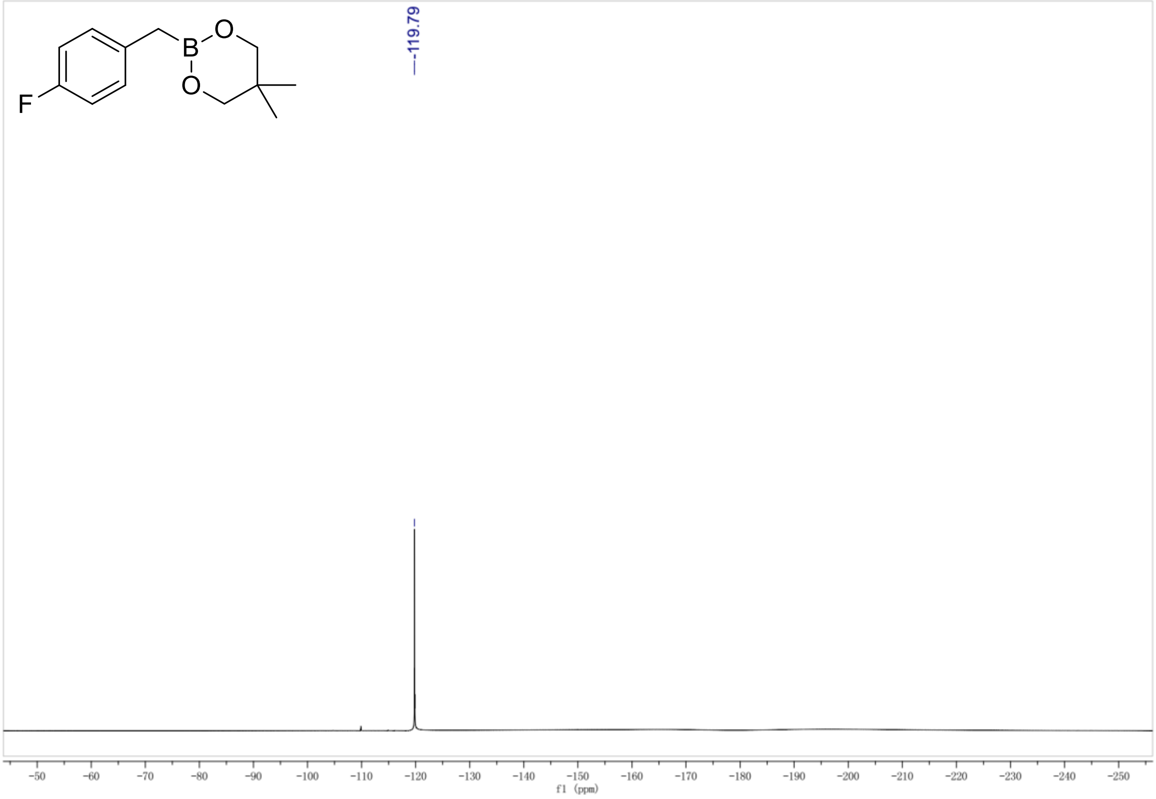

^{19}F{^{1}H} NMR spectrum of compound **6b** in $CDCl_3$ (470 MHz).

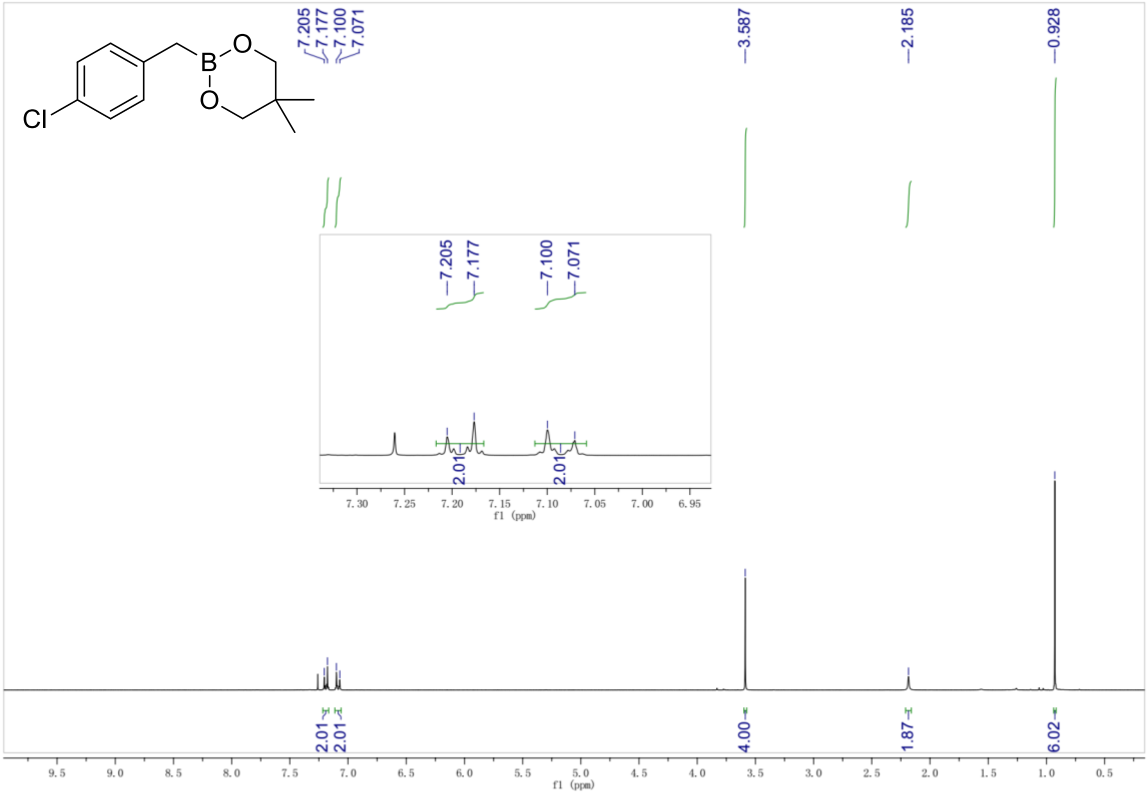

^{1}H NMR spectrum of compound **7b** in $CDCl_3$ (300 MHz).

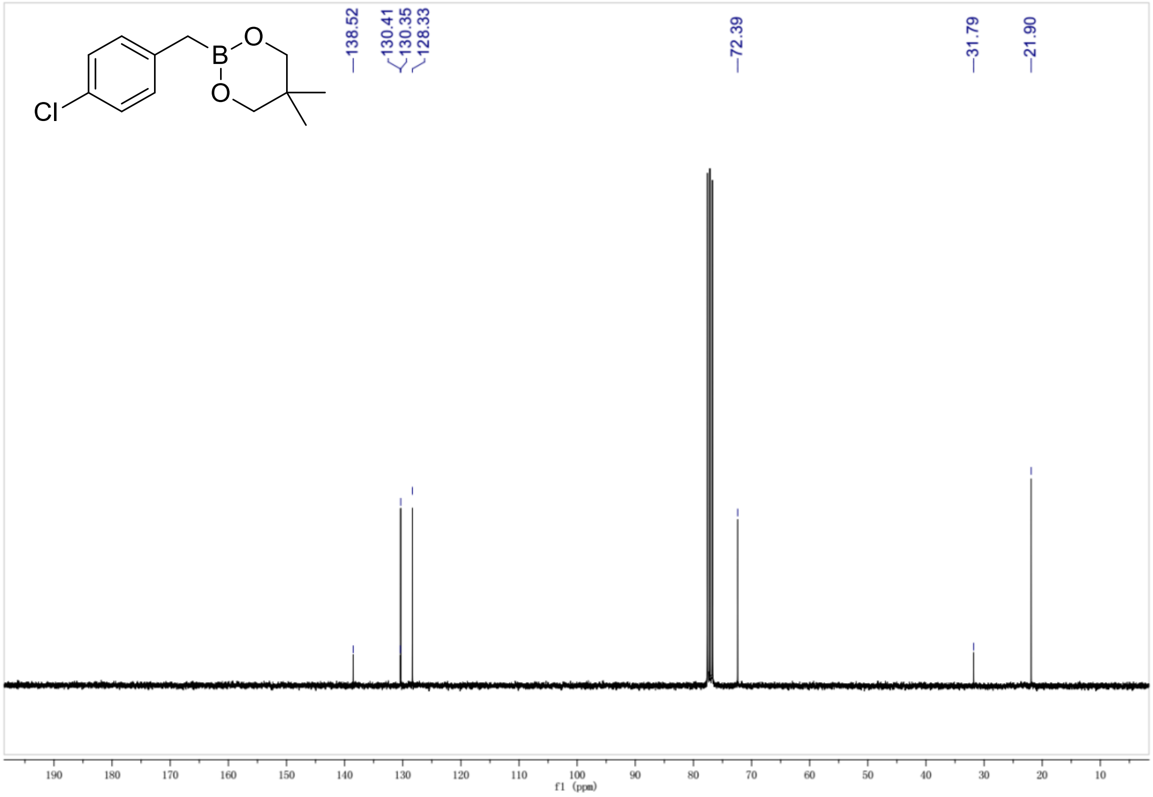

$^{13}C\{^{1}H\}$ NMR spectrum of compound **7b** in $CDCl_3$ (75 MHz).

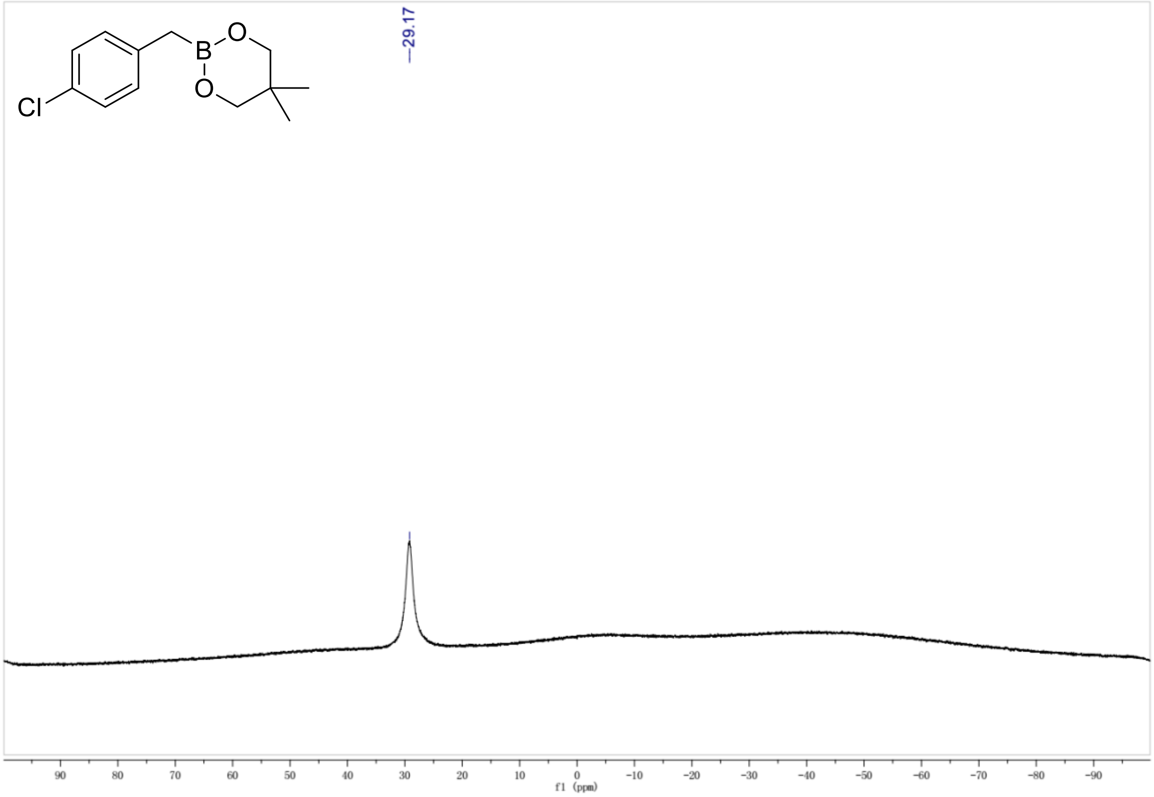

$^{11}B\{^{1}H\}$ NMR spectrum of compound **7b** in $CDCl_3$ (96 MHz).

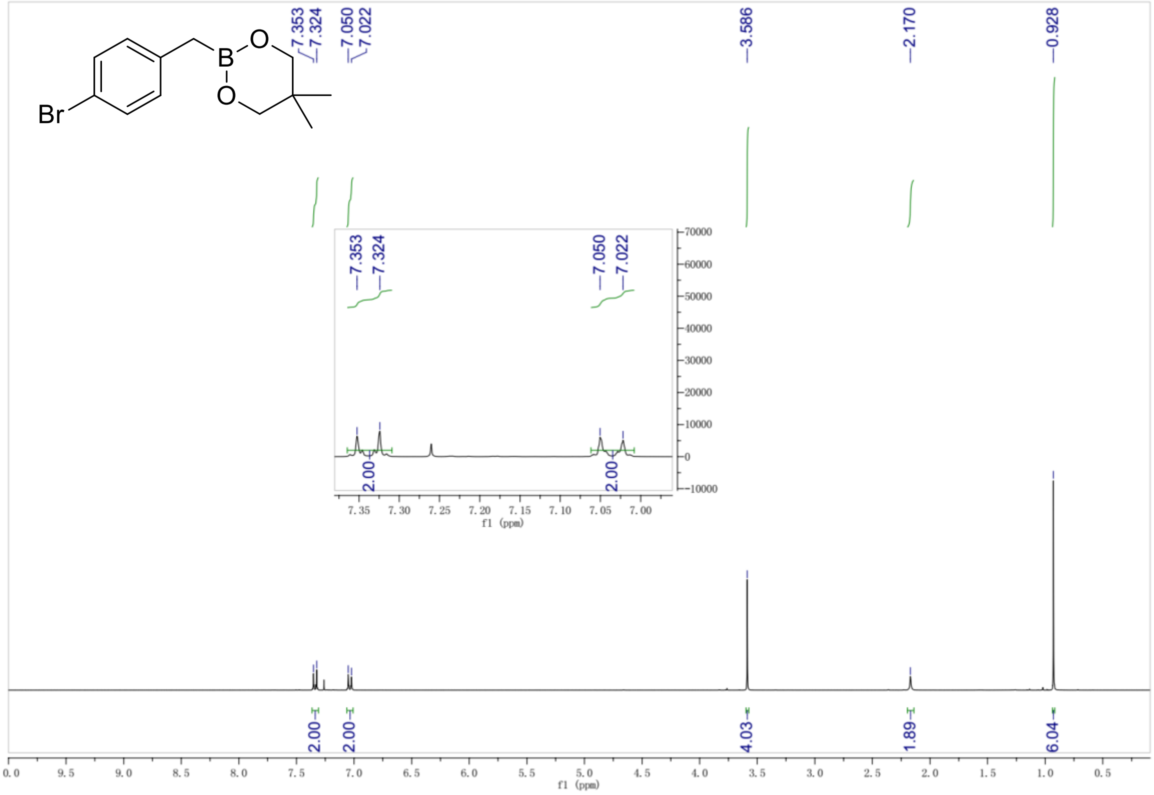

^{1}H NMR spectrum of compound **8b** in $CDCl_3$ (300 MHz).

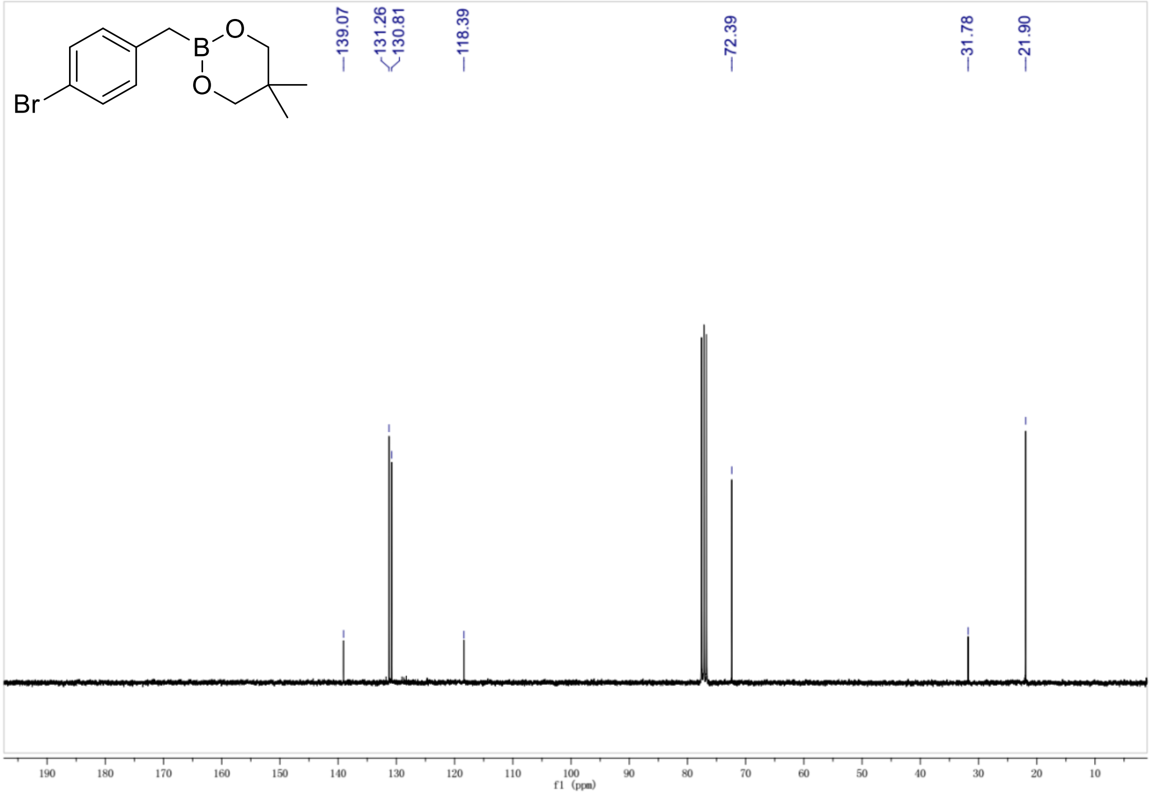

$^{13}C\{^1H\}$ NMR spectrum of compound **8b** in $CDCl_3$ (75 MHz).

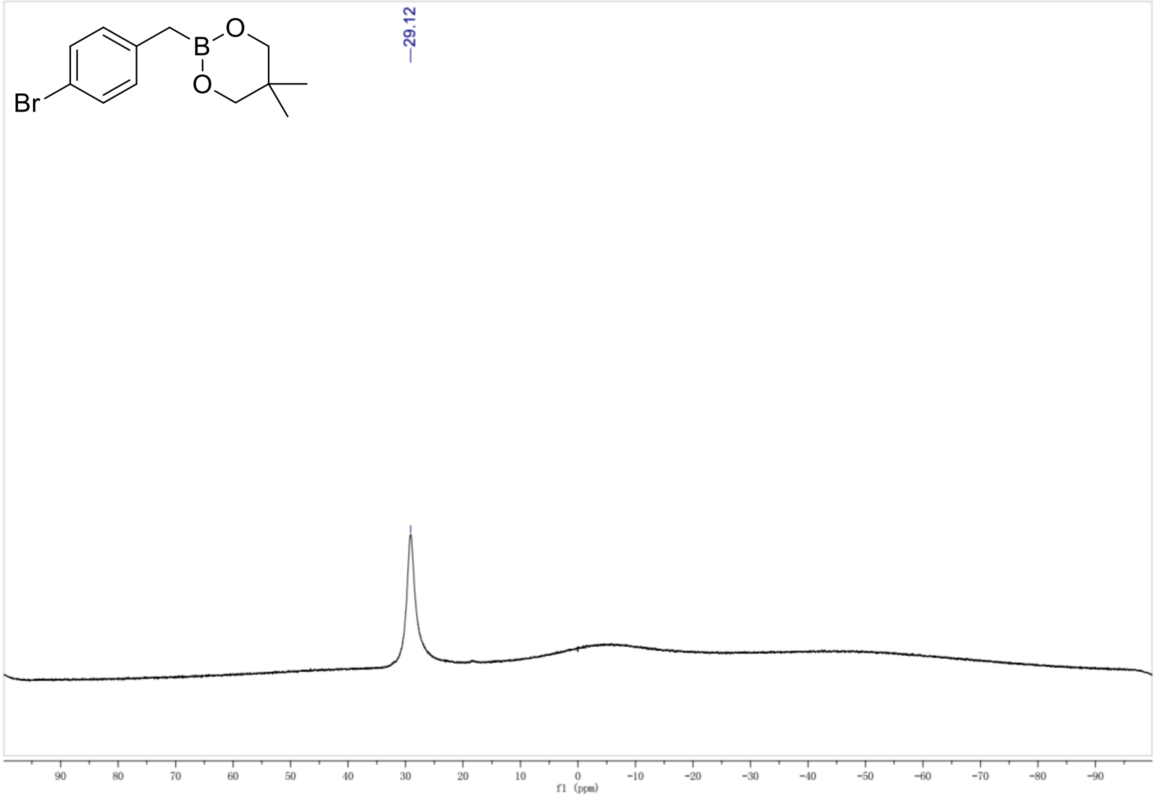

$^{11}B\{^1H\}$ NMR spectrum of compound **8b** in $CDCl_3$ (96 MHz).

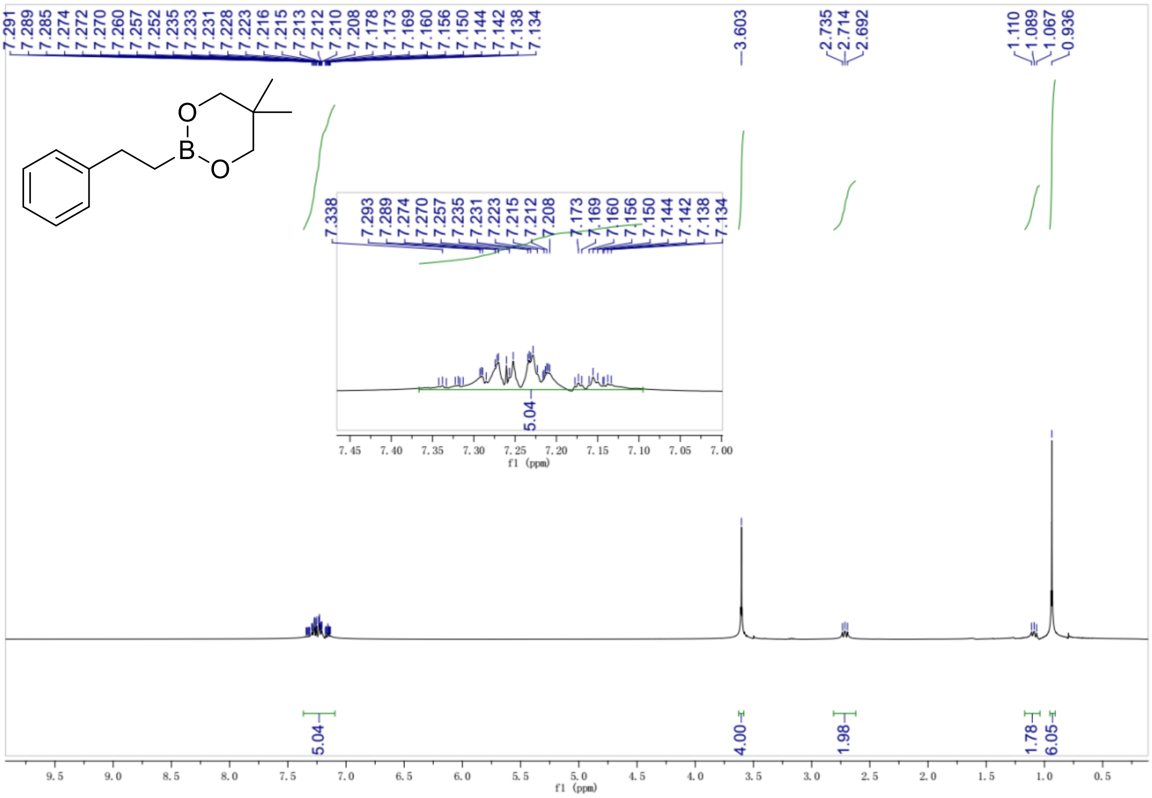

¹H NMR spectrum of compound **9b** in $CDCl_3$ (400 MHz).

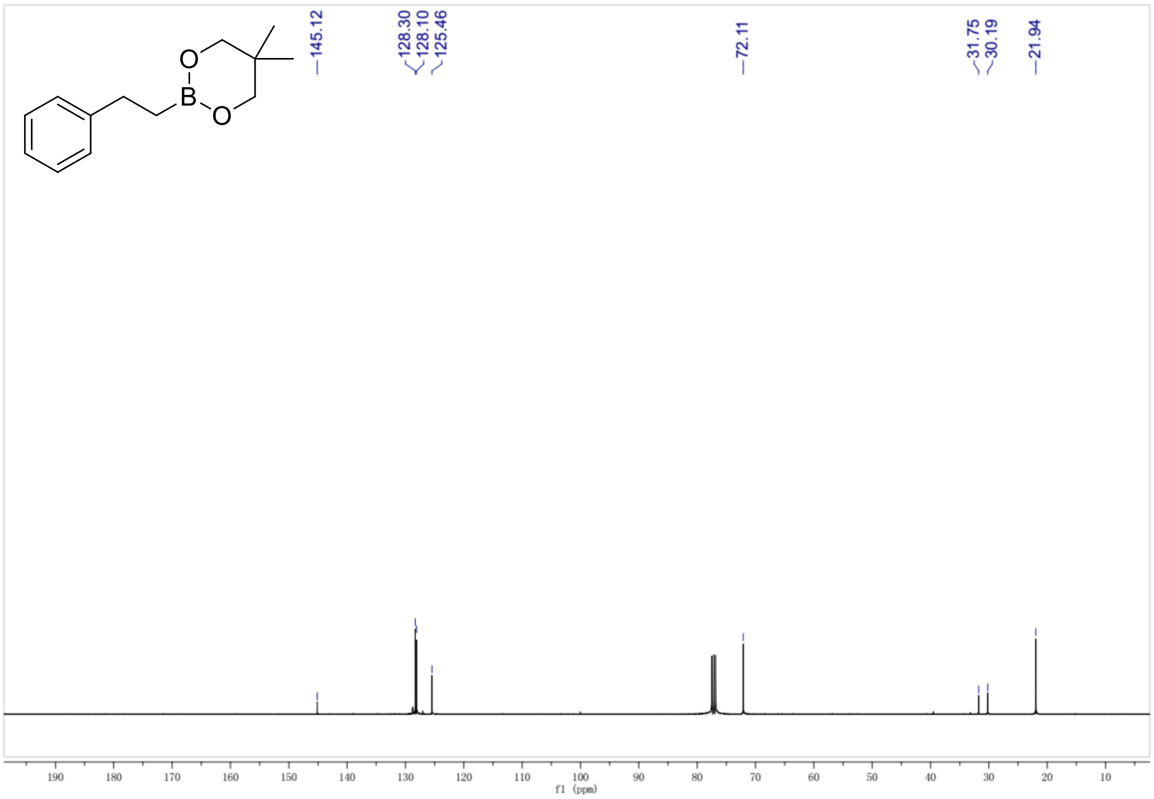

¹³C{¹H} NMR spectrum of compound **9b** in $CDCl_3$ (100 MHz).

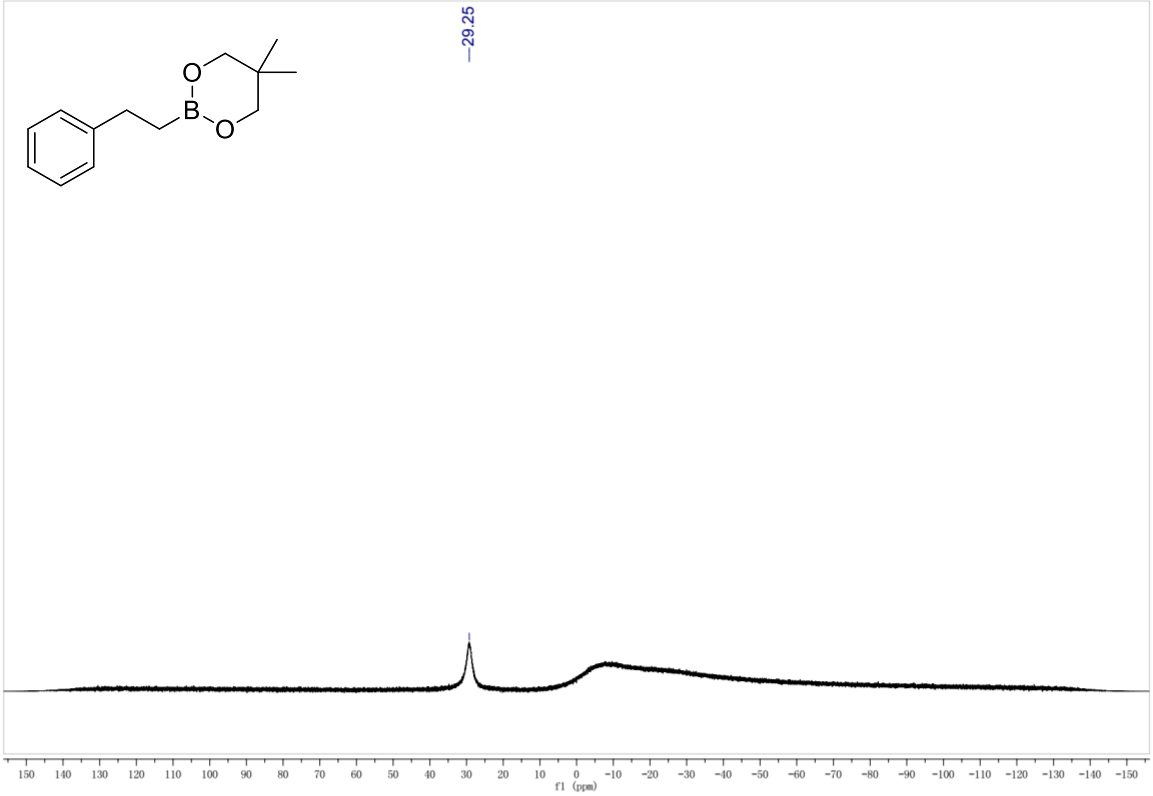

$^{11}B\{^{1}H\}$ NMR spectrum of compound **9b** in $CDCl_3$ (128 MHz).

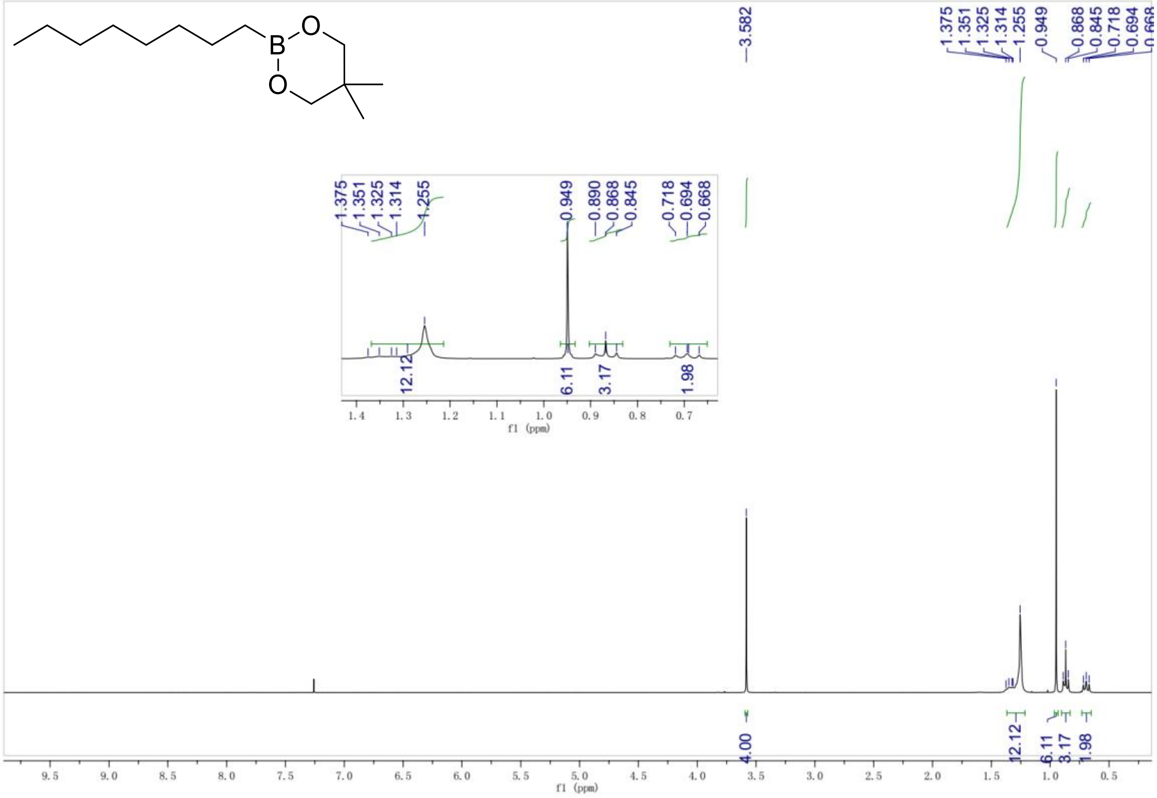

^{1}H NMR spectrum of compound **10b** in $CDCl_3$ (300 MHz).

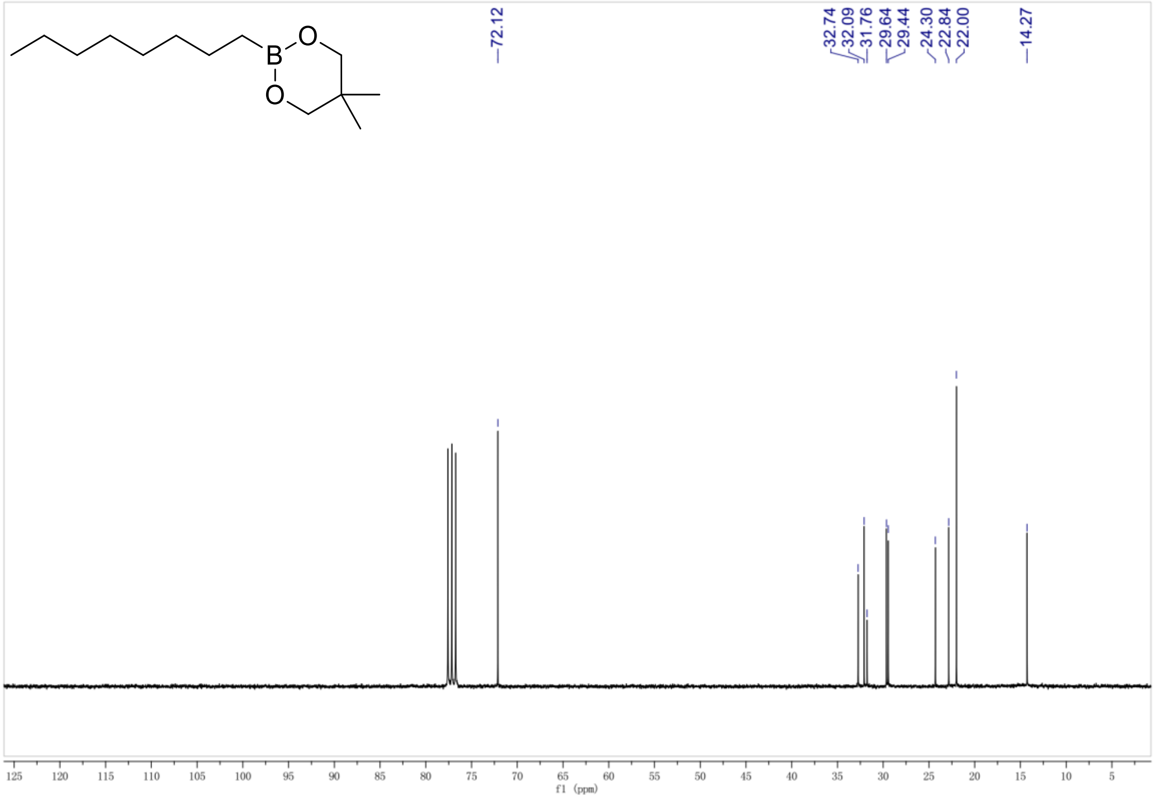

$^{13}C\{^{1}H\}$ NMR spectrum of compound **10b** in $CDCl_3$ (75 MHz).

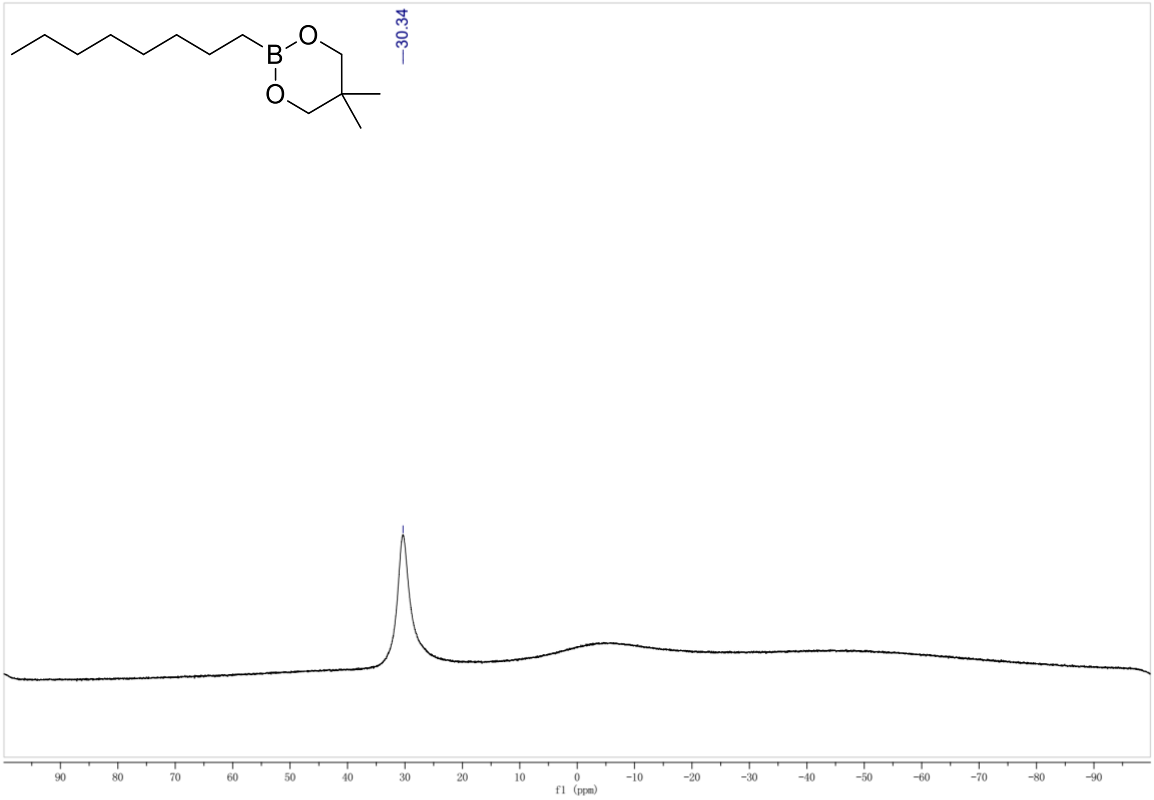

$^{11}B\{^{1}H\}$ NMR spectrum of compound **10b** in $CDCl_3$ (96 MHz).

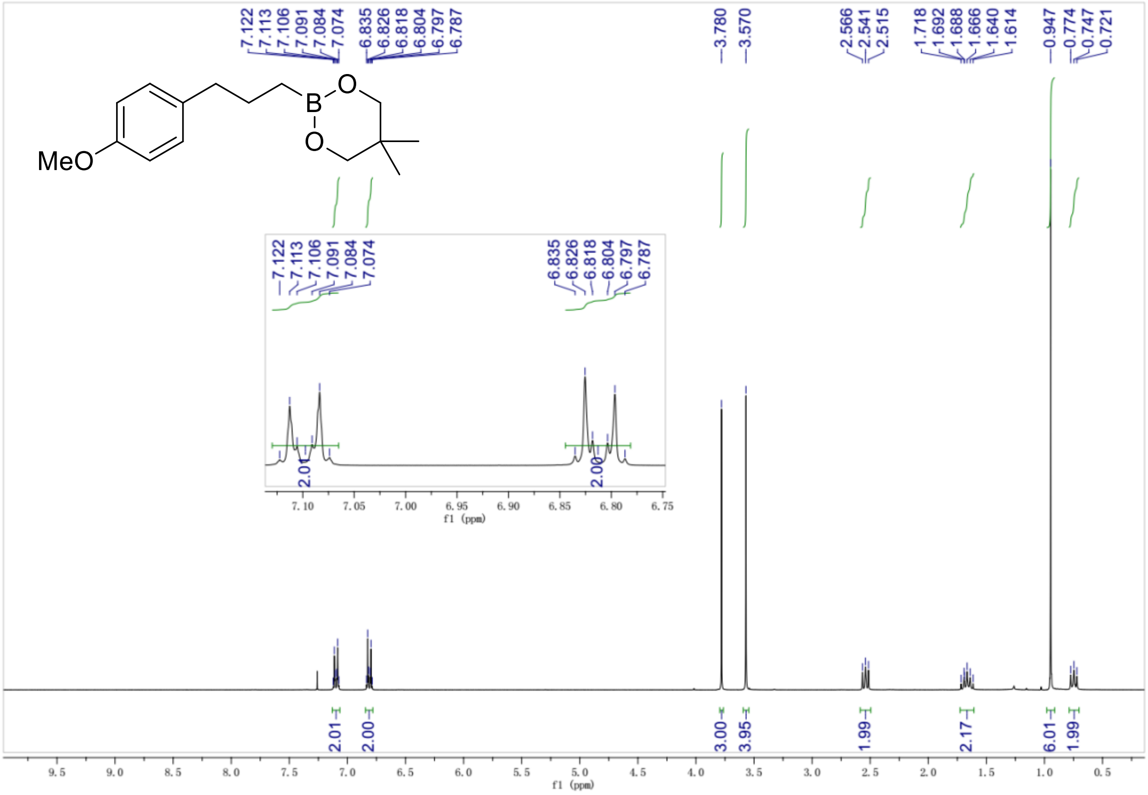

^{1}H NMR spectrum of compound **11b** in $CDCl_3$ (300 MHz).

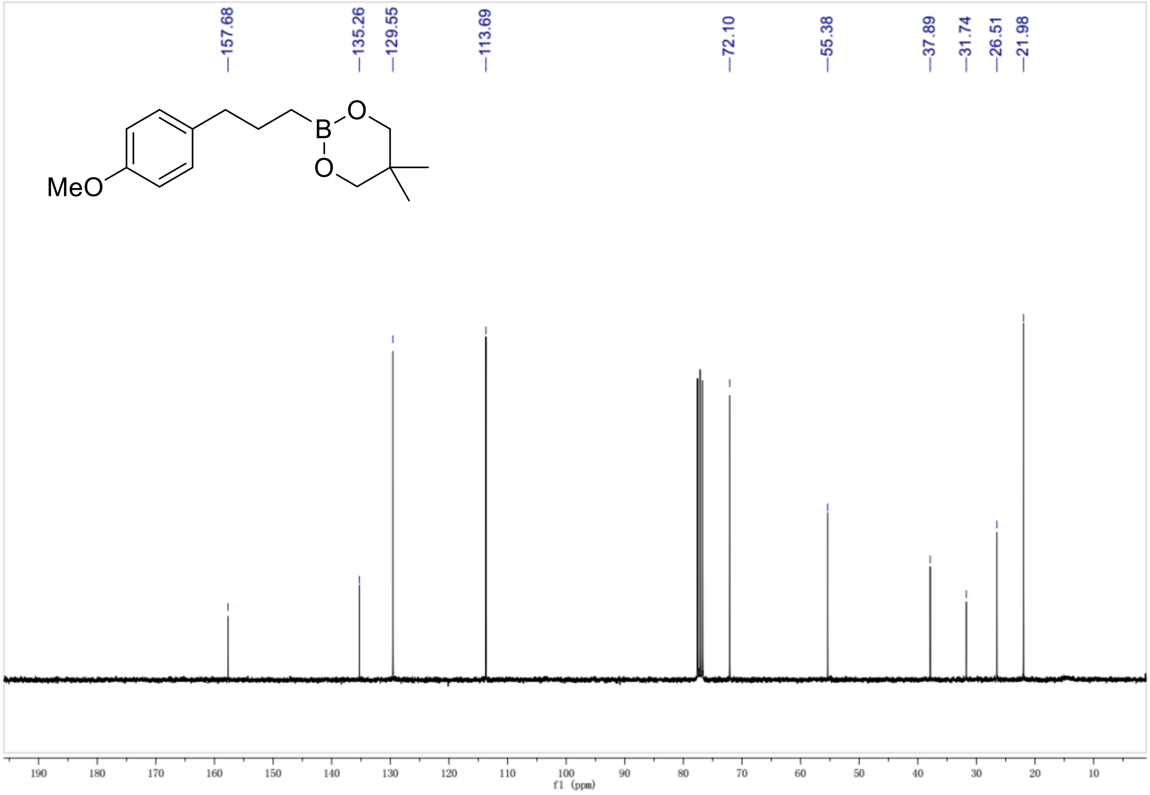

^{13}C{^{1}H} NMR spectrum of compound **11b** in $CDCl_3$ (75 MHz).

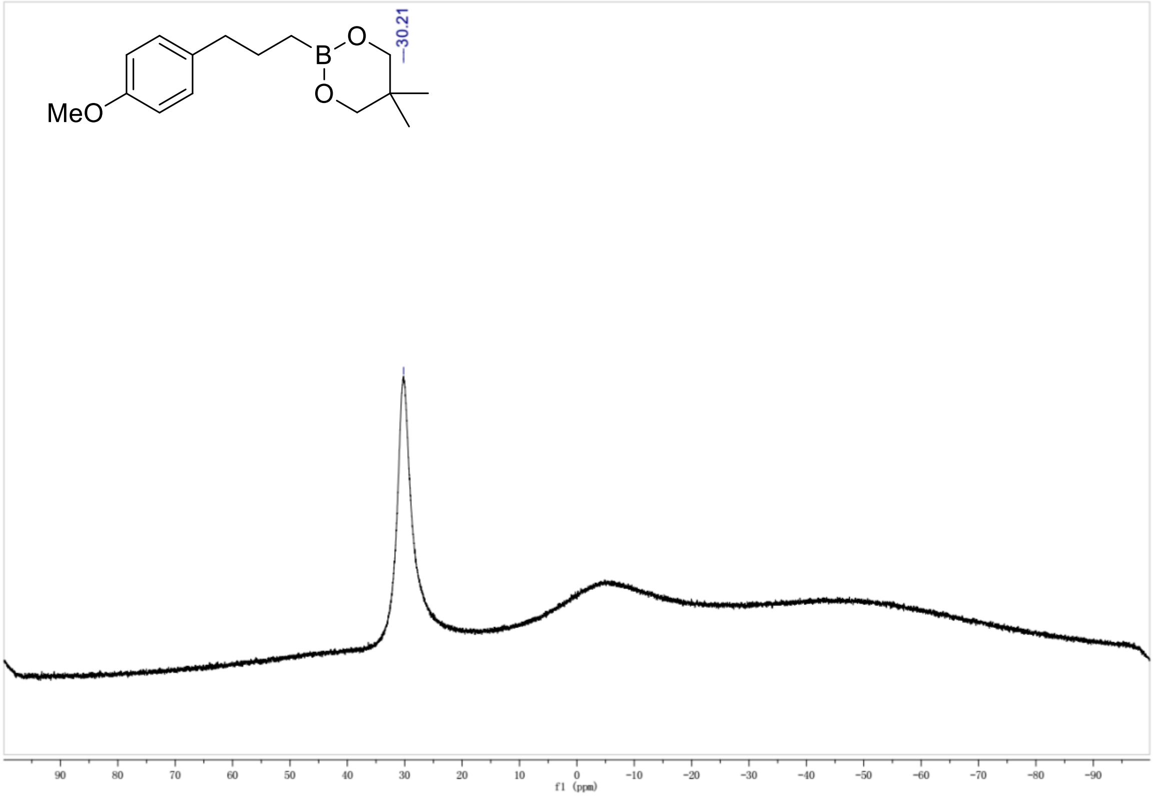

^{11}B{^{1}H} NMR spectrum of compound **11b** in $CDCl_3$ (96 MHz).

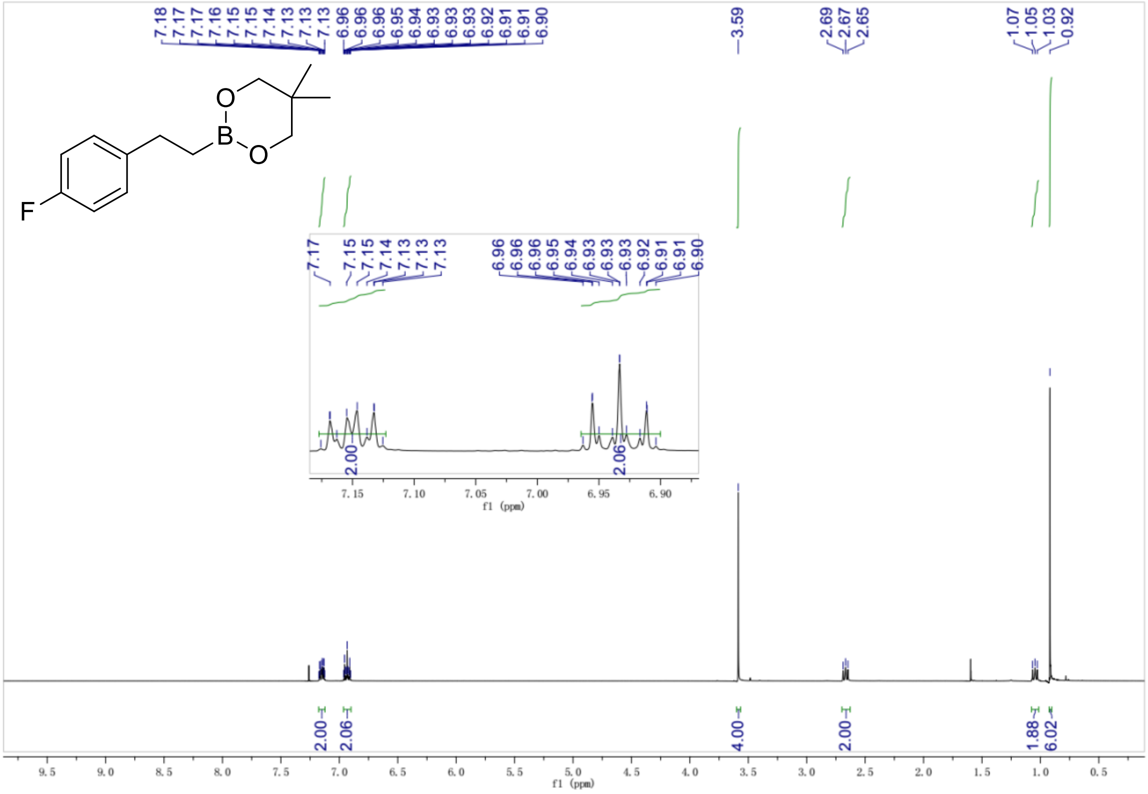

^{1}H NMR spectrum of compound **12b** in $CDCl_3$ (400 MHz).

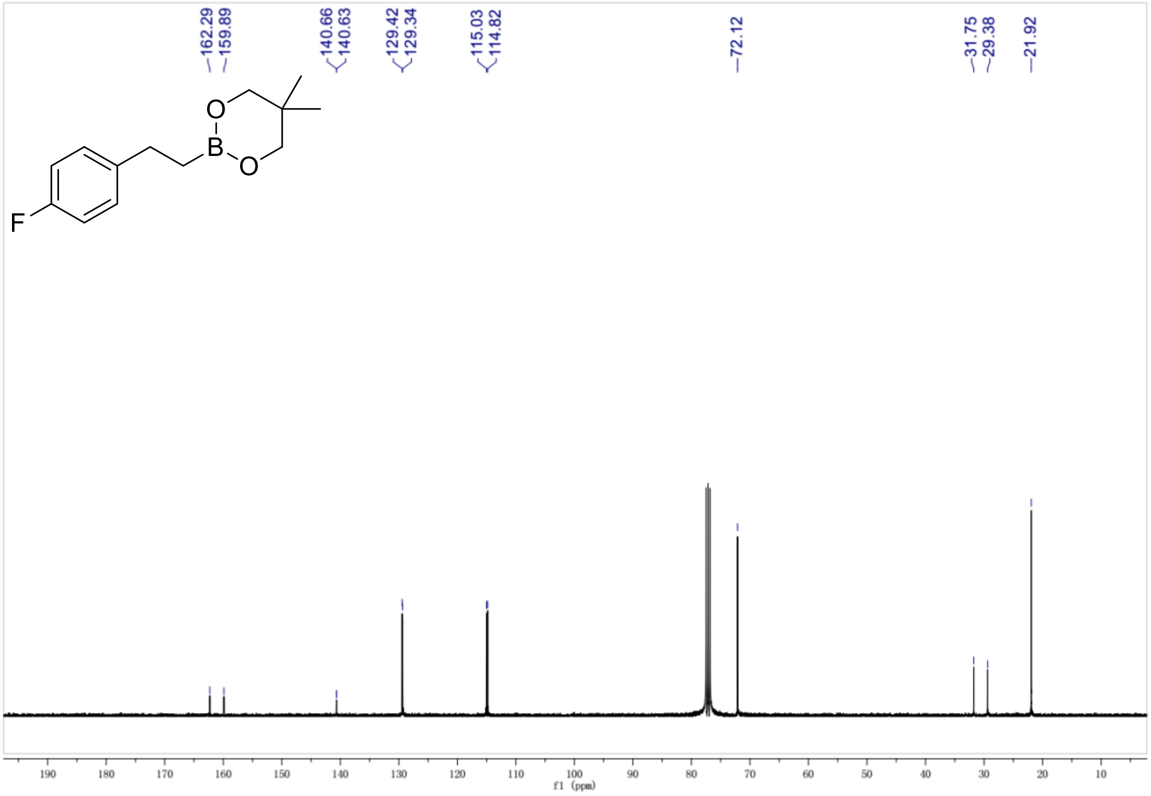

$^{13}C\{^1H\}$ NMR spectrum of compound **12b** in $CDCl_3$ (100 MHz).

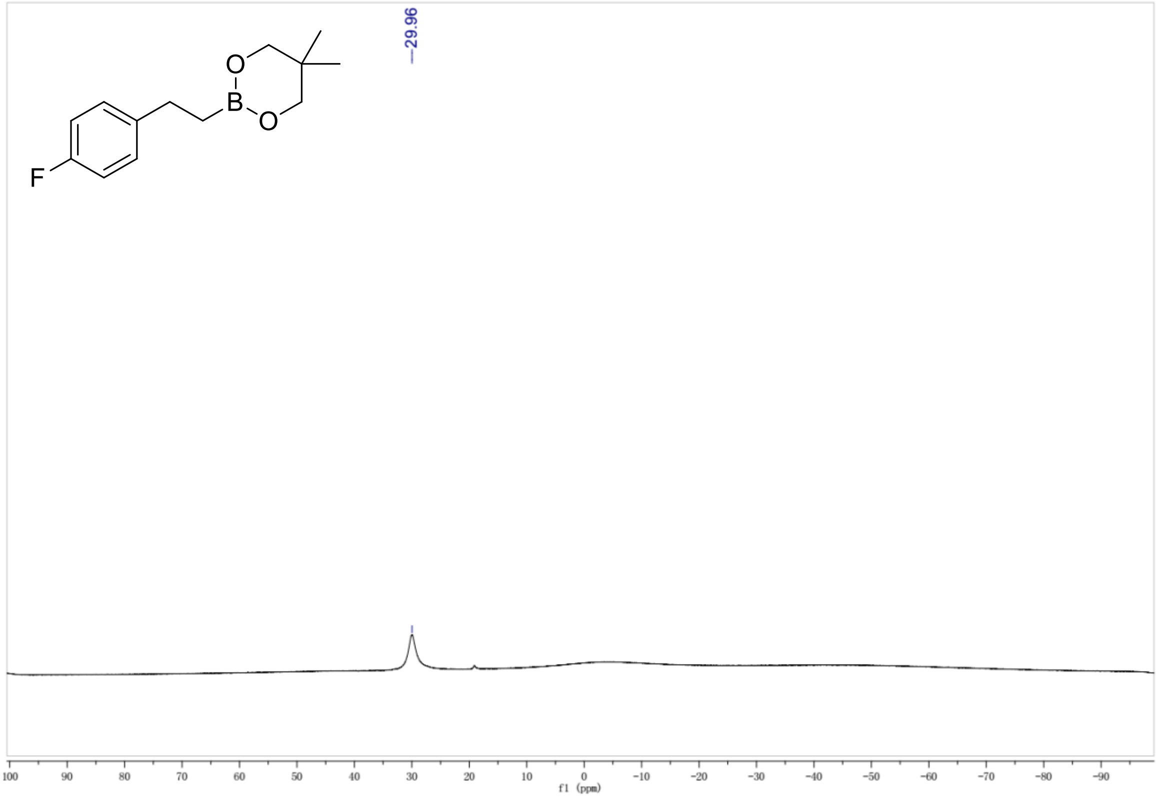

$^{11}B\{^1H\}$ NMR spectrum of compound **12b** in $CDCl_3$ (128 MHz).

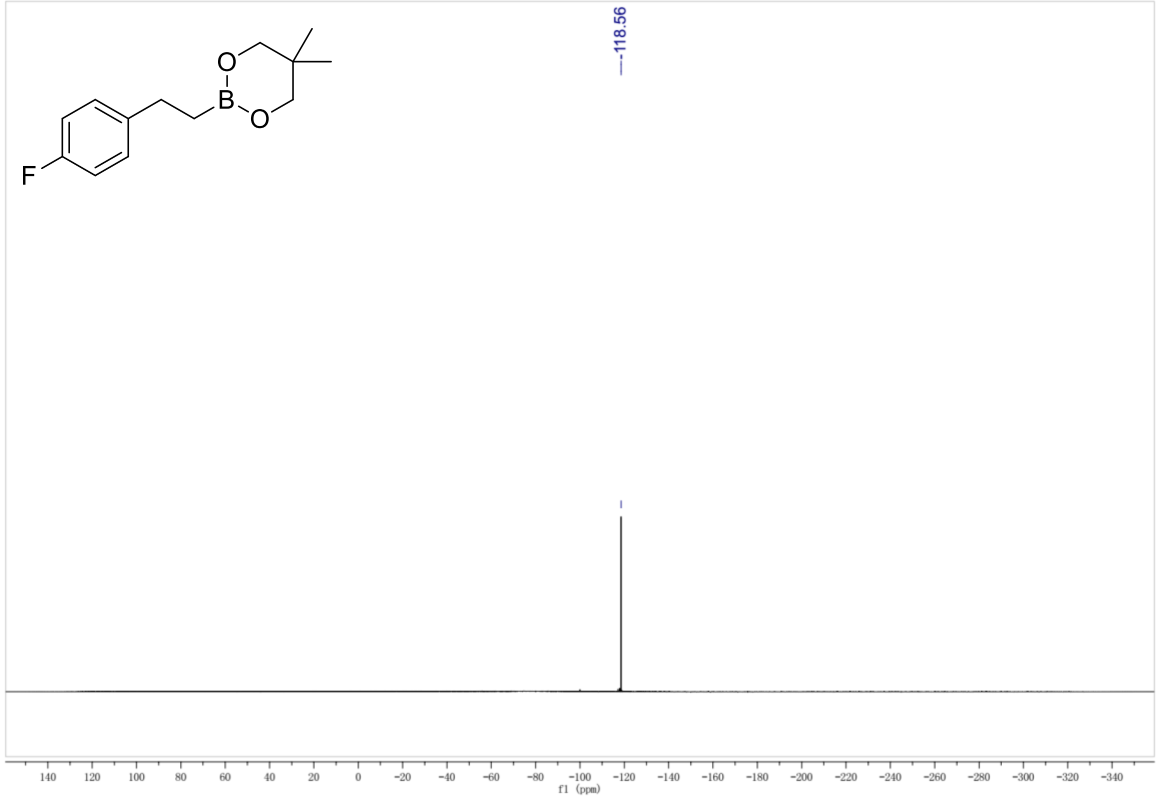

$^{19}F\{^{1}H\}$ NMR spectrum of compound **12b** in $CDCl_3$ (376 MHz).

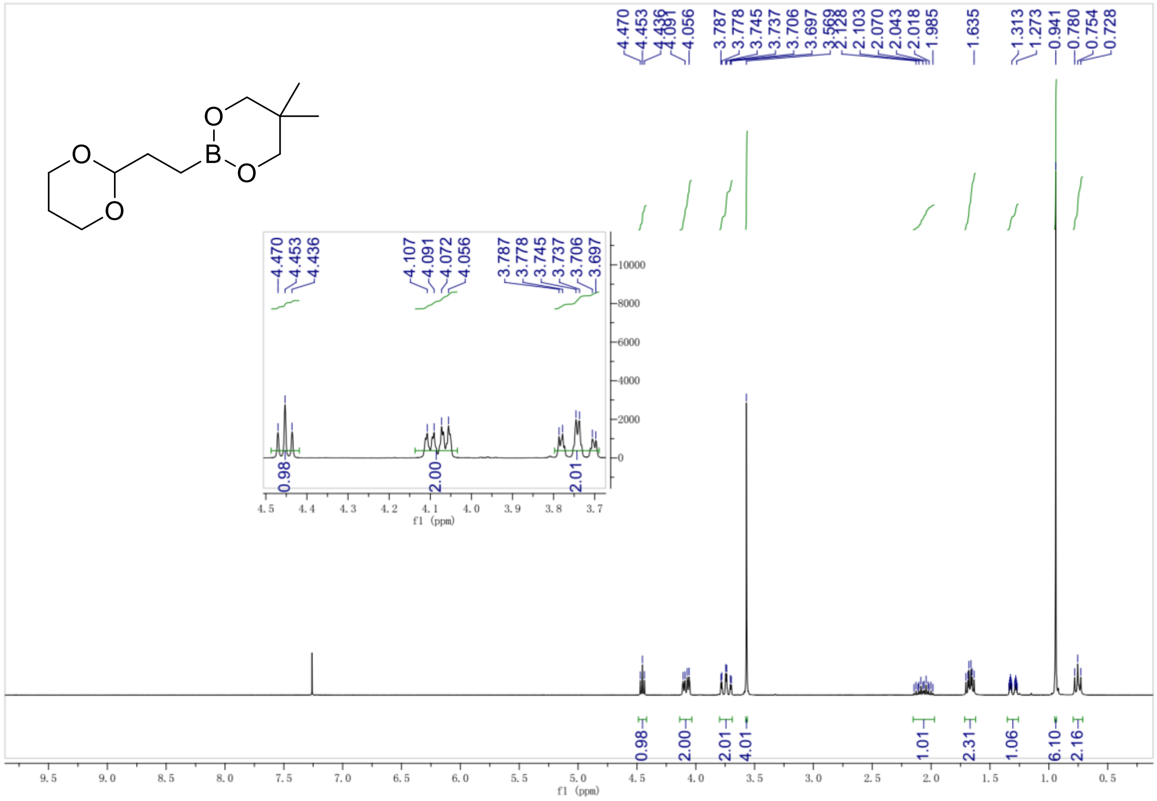

^{1}H NMR spectrum of compound **14b** in $CDCl_3$ (300 MHz).

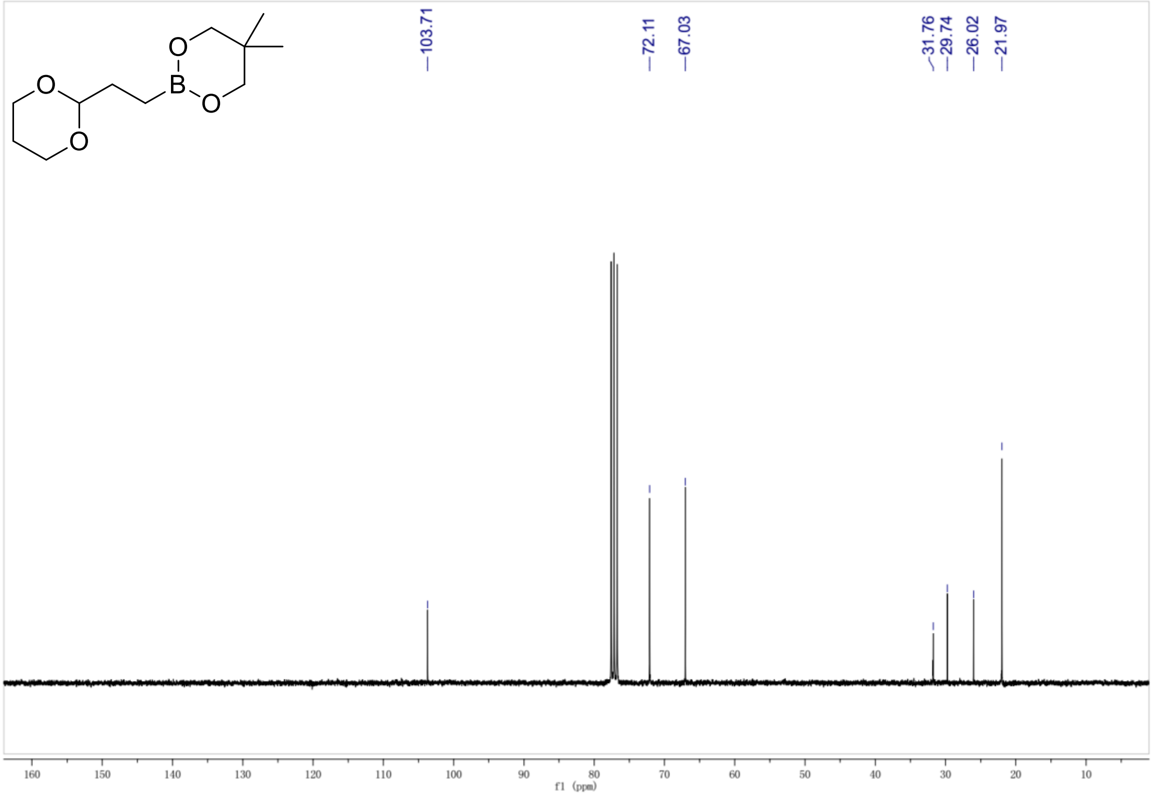

$^{13}C\{^{1}H\}$ NMR spectrum of compound **14b** in $CDCl_3$ (75 MHz).

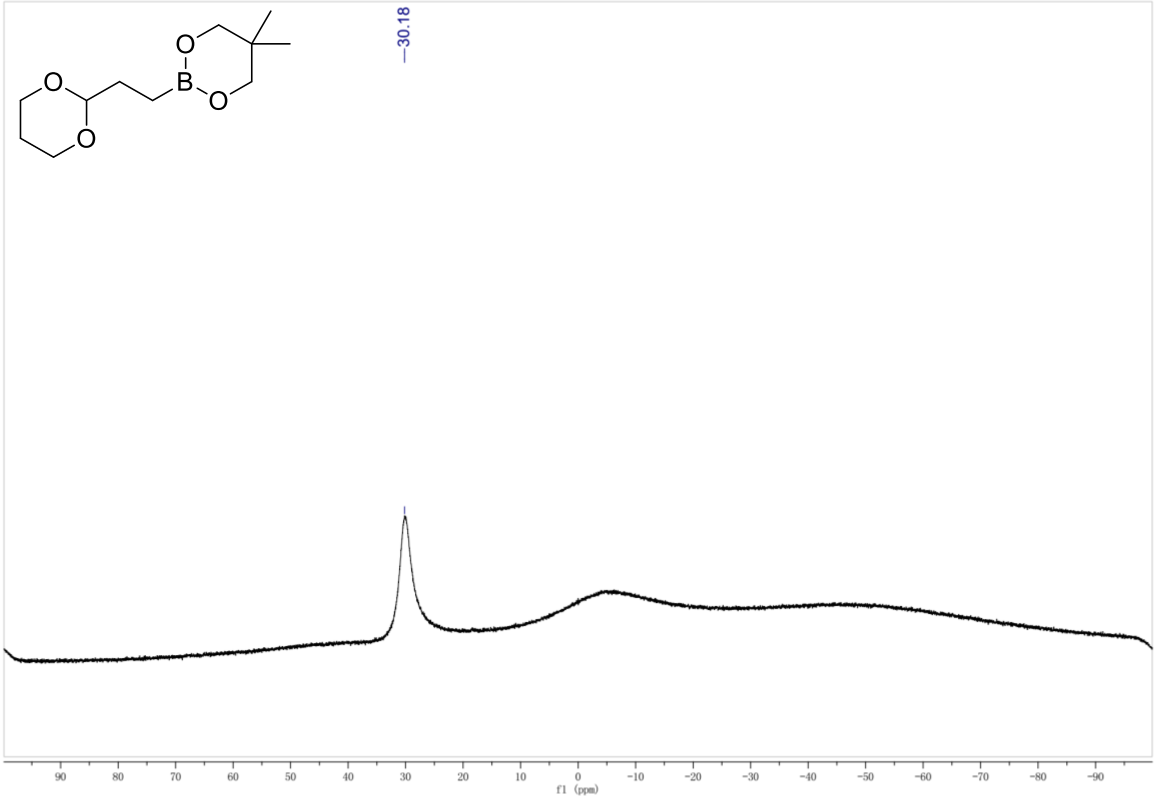

$^{11}B\{^{1}H\}$ NMR spectrum of compound **14b** in $CDCl_3$ (96 MHz).

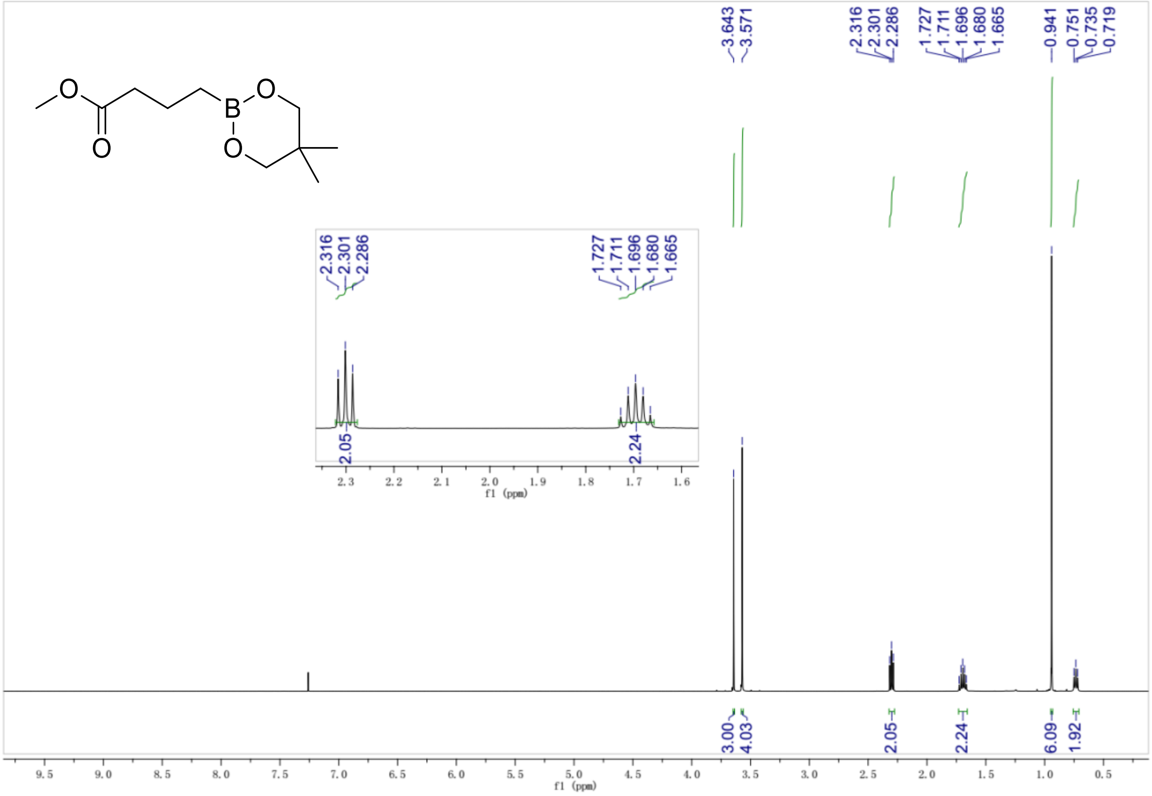

^{1}H NMR spectrum of compound **15b** in $CDCl_3$ (500 MHz).

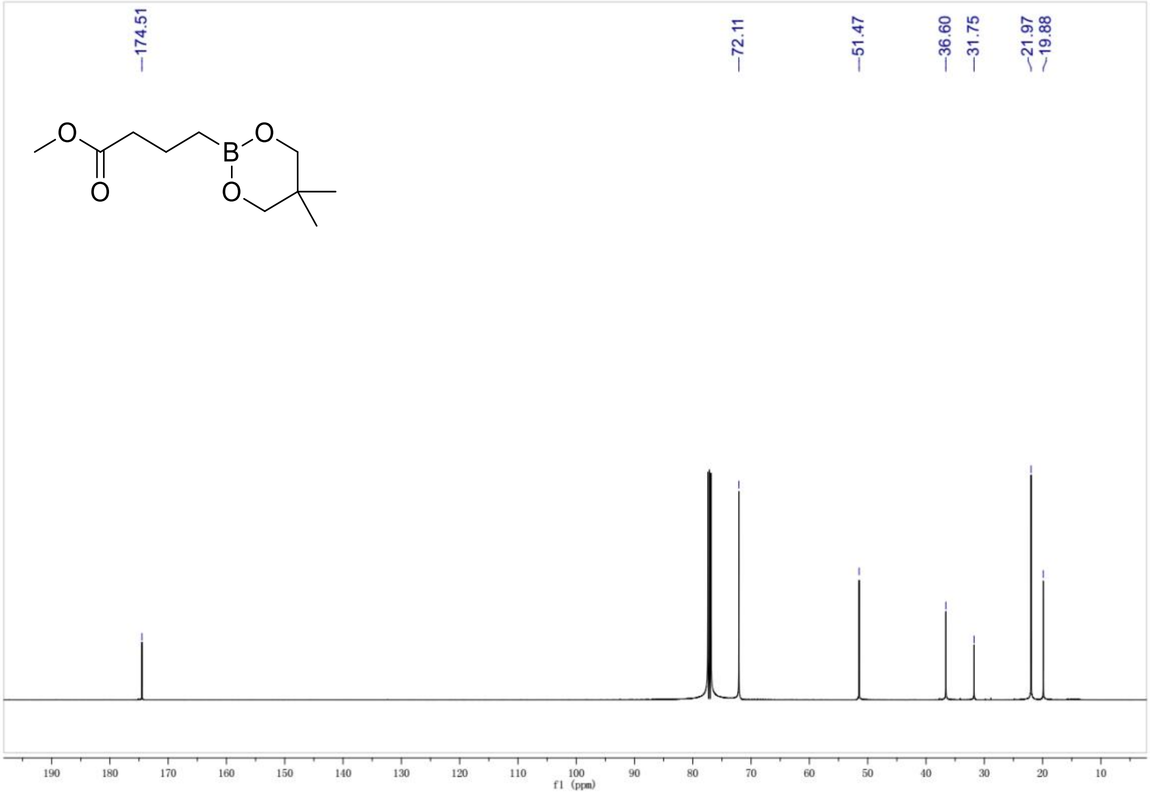

^{13}C{^{1}H} NMR spectrum of compound **15b** in $CDCl_3$ (125 MHz).

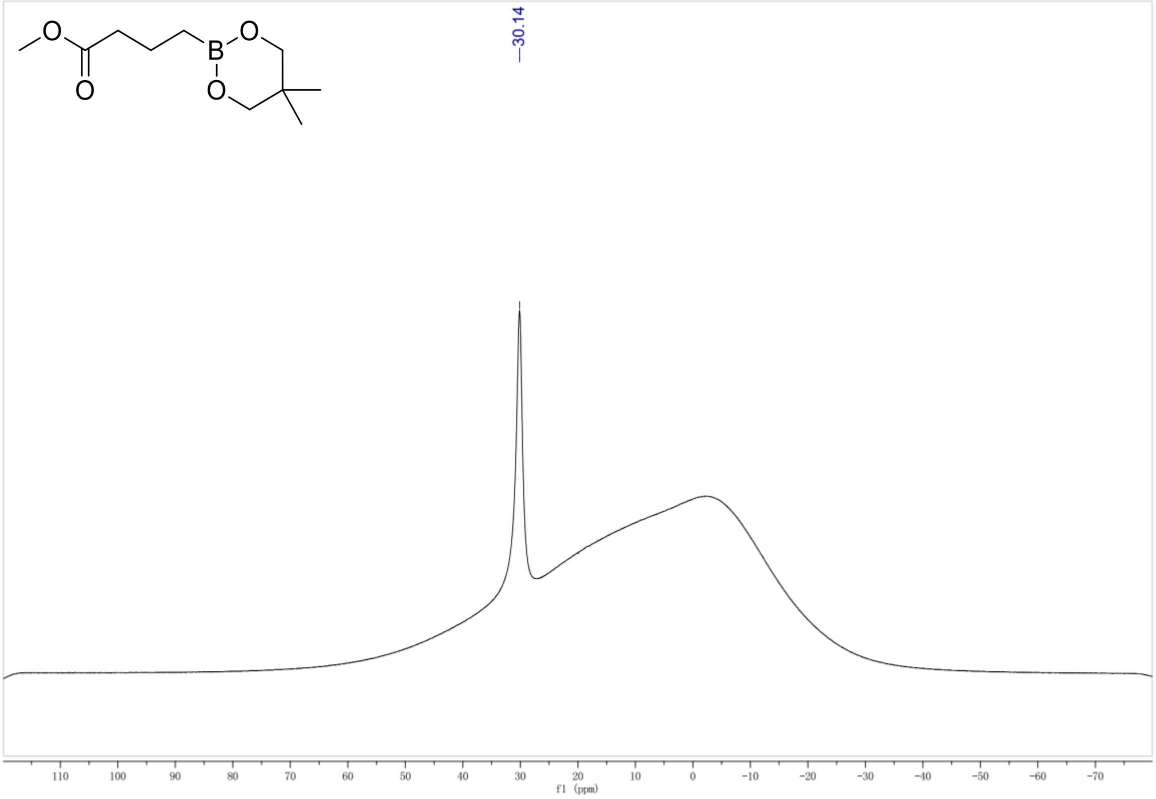

$^{11}B\{^{1}H\}$ NMR spectrum of compound **15b** in $CDCl_3$ (160 MHz).

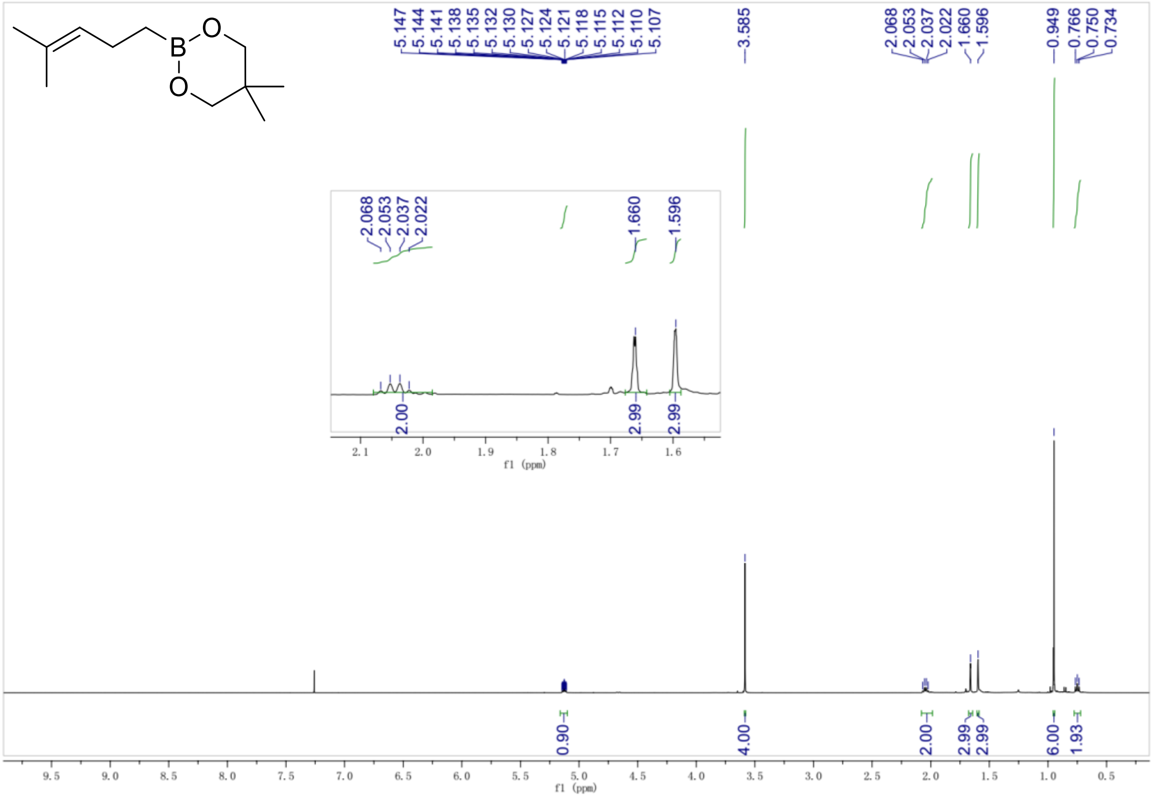

^{1}H NMR spectrum of compound **16b** in $CDCl_3$ (500 MHz).

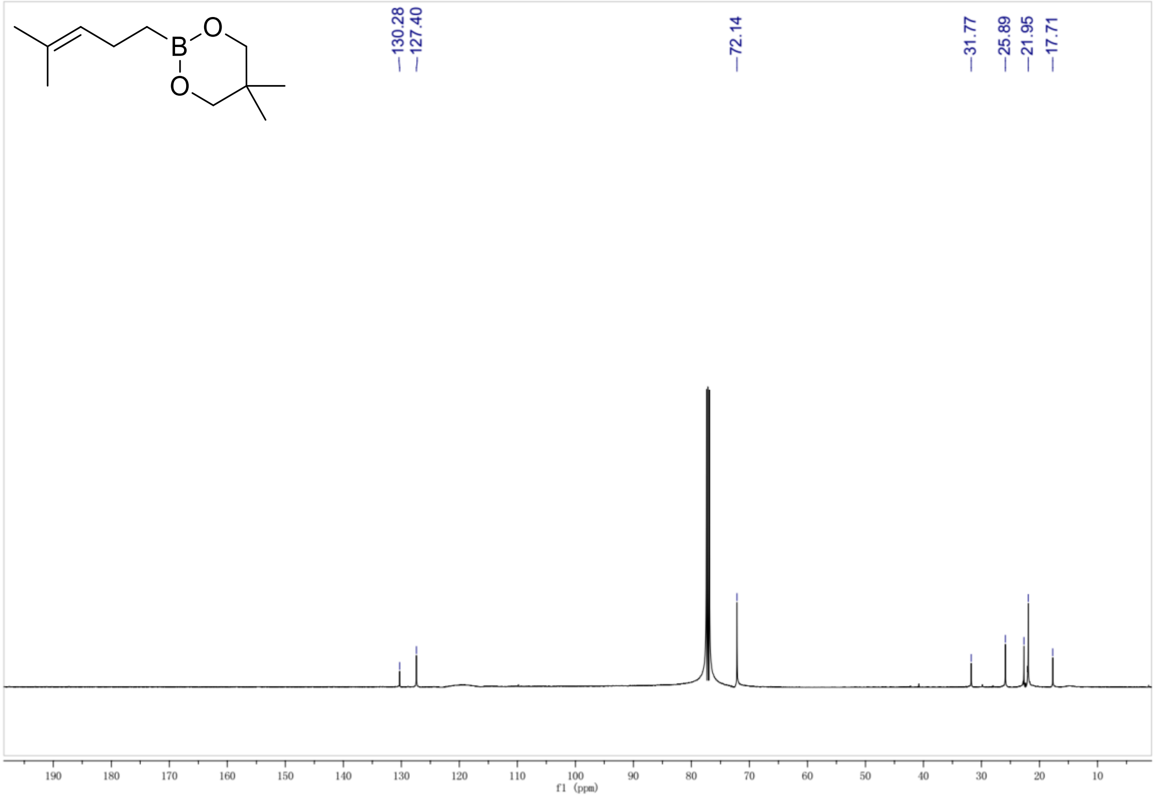

$^{13}C\{^{1}H\}$ NMR spectrum of compound **16b** in $CDCl_3$ (125 MHz).

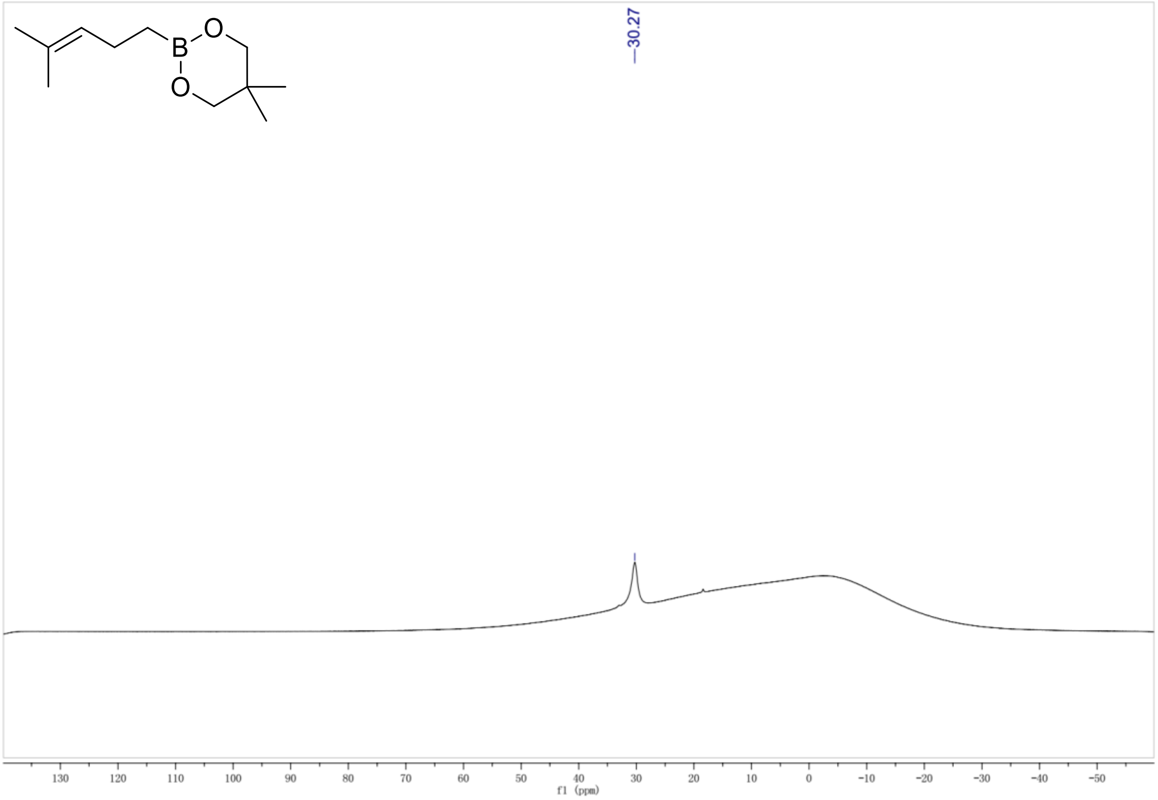

$^{11}B\{^{1}H\}$ NMR spectrum of compound **16b** in $CDCl_3$ (160 MHz).

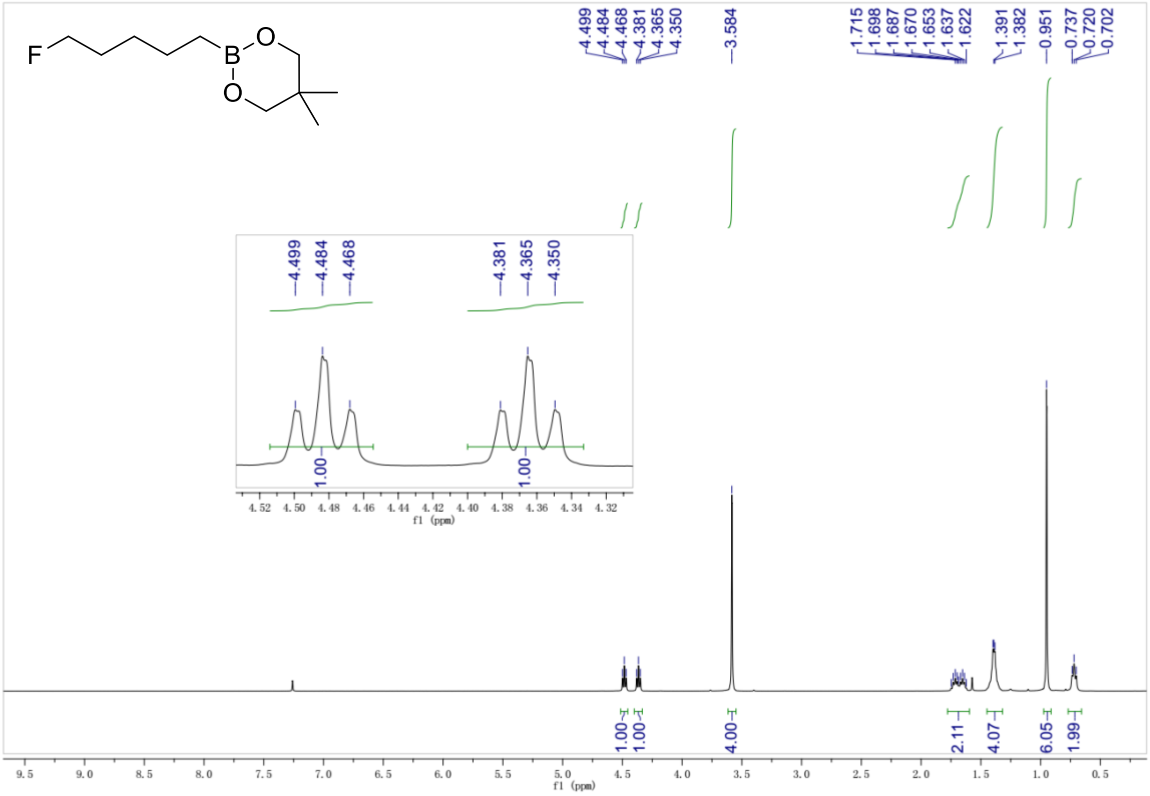

^{1}H NMR spectrum of compound **17b** in $CDCl_3$ (400 MHz).

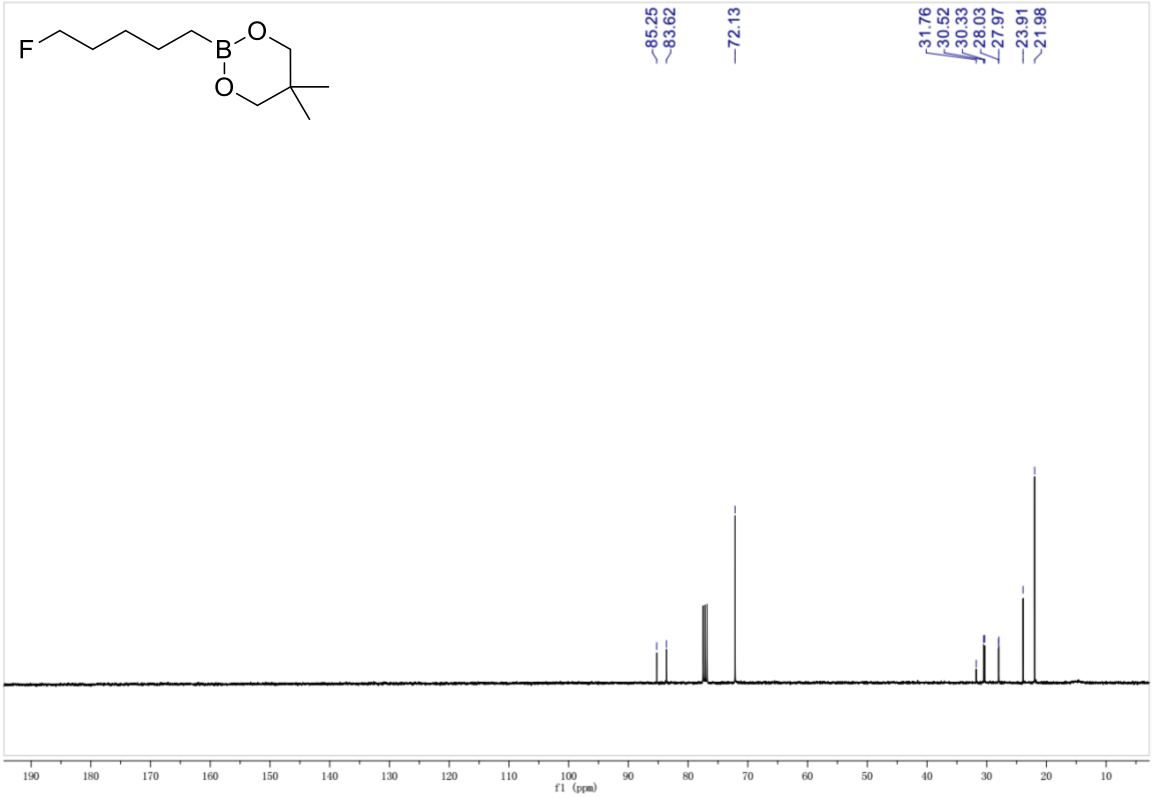

^{13}C{^{1}H} NMR spectrum of compound **17b** in $CDCl_3$ (100 MHz).

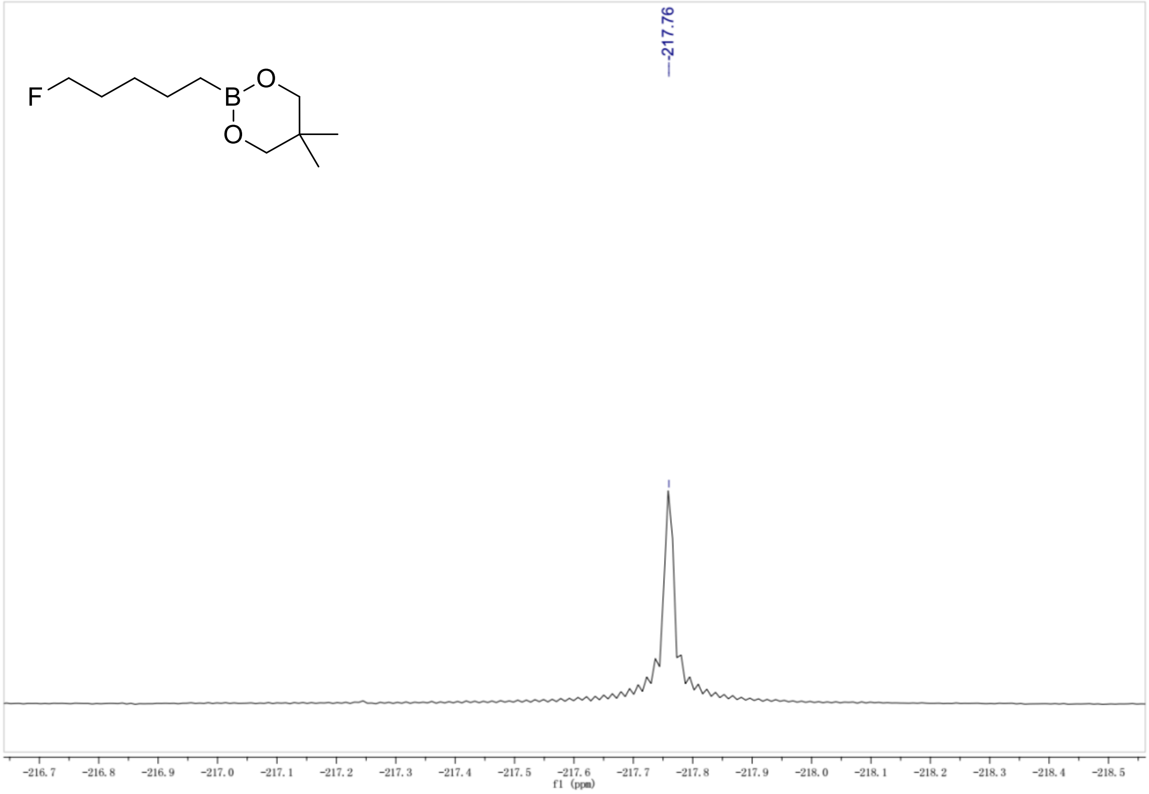

$^{19}F\{^1H\}$ NMR spectrum of compound **17b** in $CDCl_3$ (376 MHz).

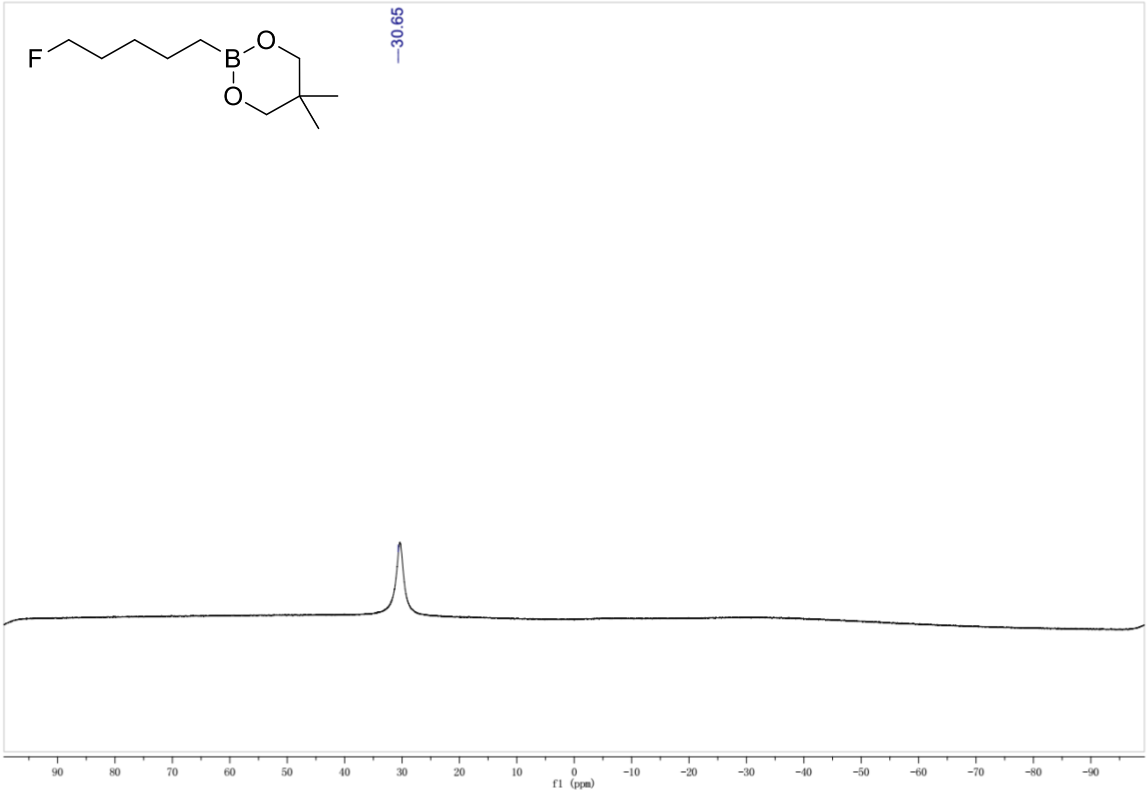

$^{11}B\{^1H\}$ NMR spectrum of compound **17b** in $CDCl_3$ (128 MHz).

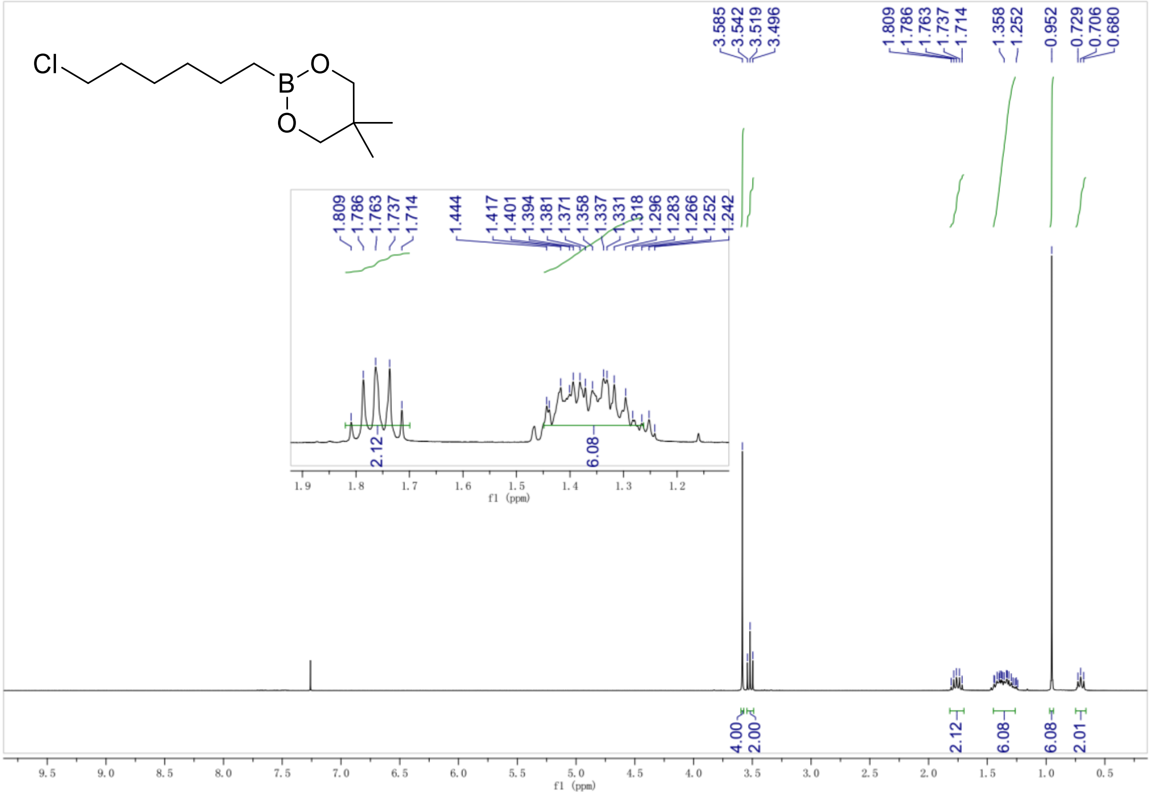

^{1}H NMR spectrum of compound **18b** in $CDCl_3$ (300 MHz).

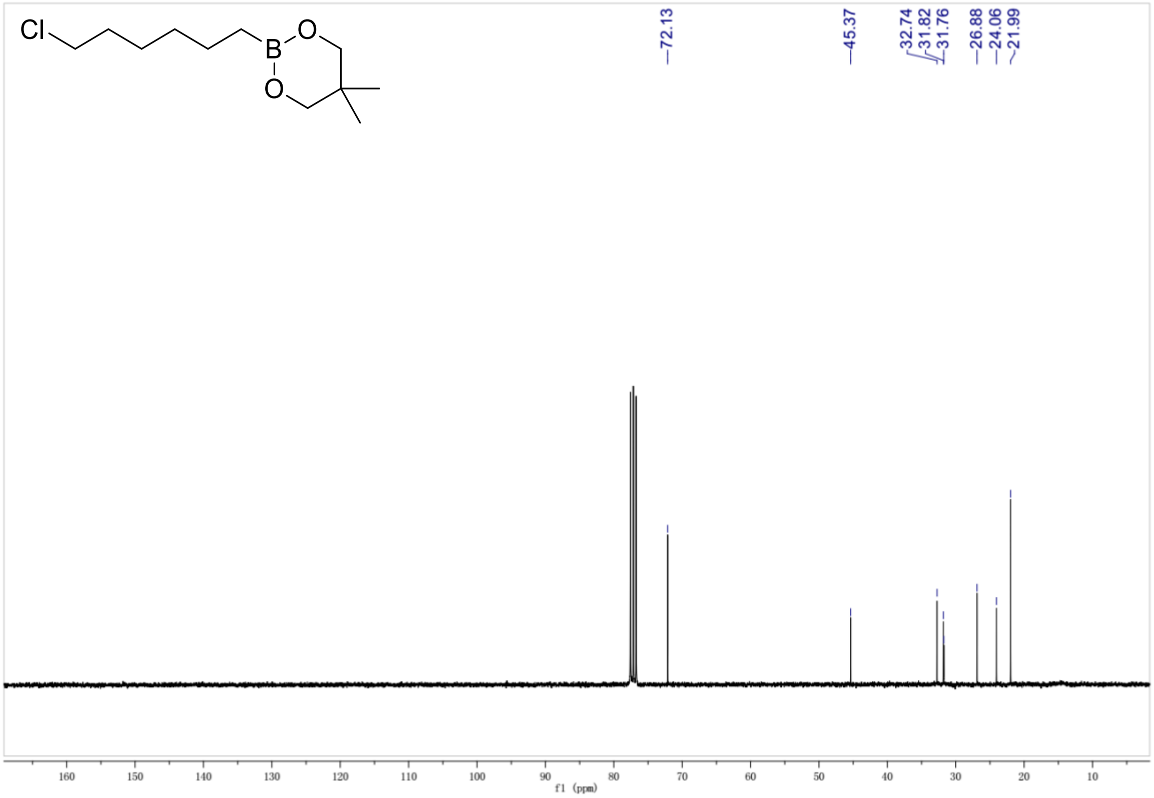

^{13}C{^{1}H} NMR spectrum of compound **18b** in $CDCl_3$ (75 MHz).

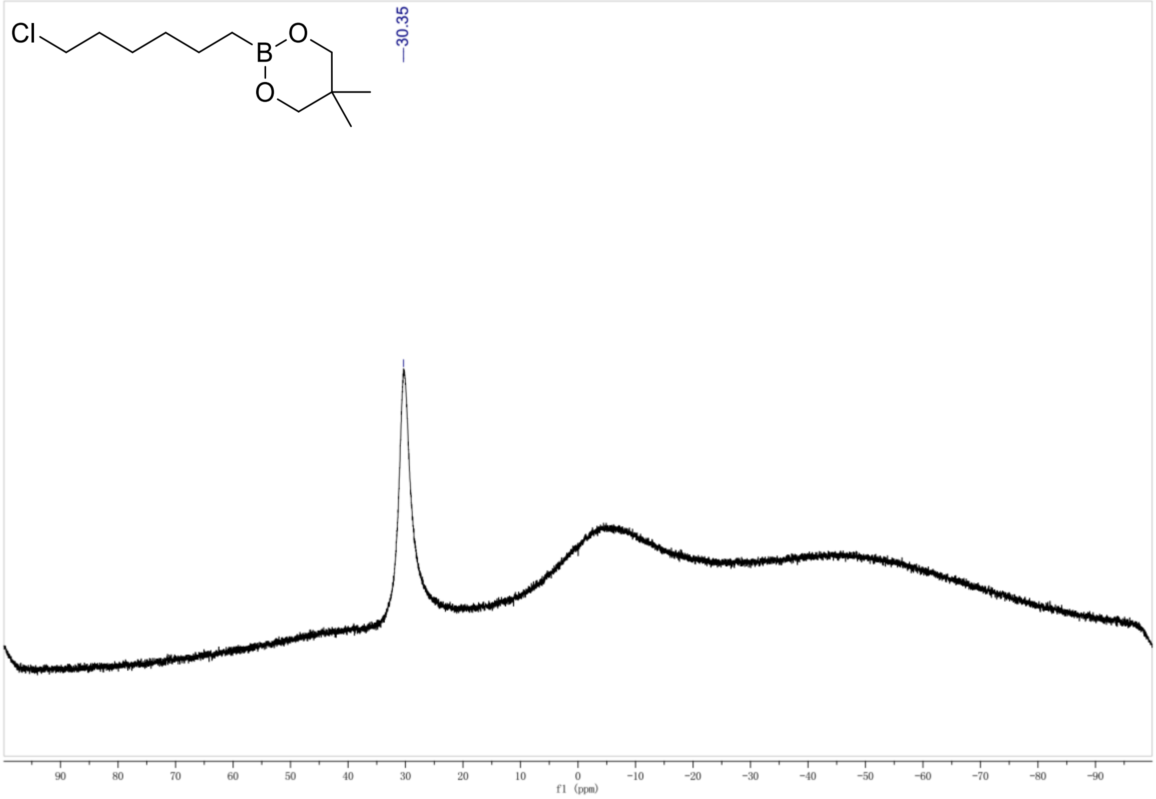

$^{11}B\{^{1}H\}$ NMR spectrum of compound **18b** in $CDCl_3$ (96 MHz).

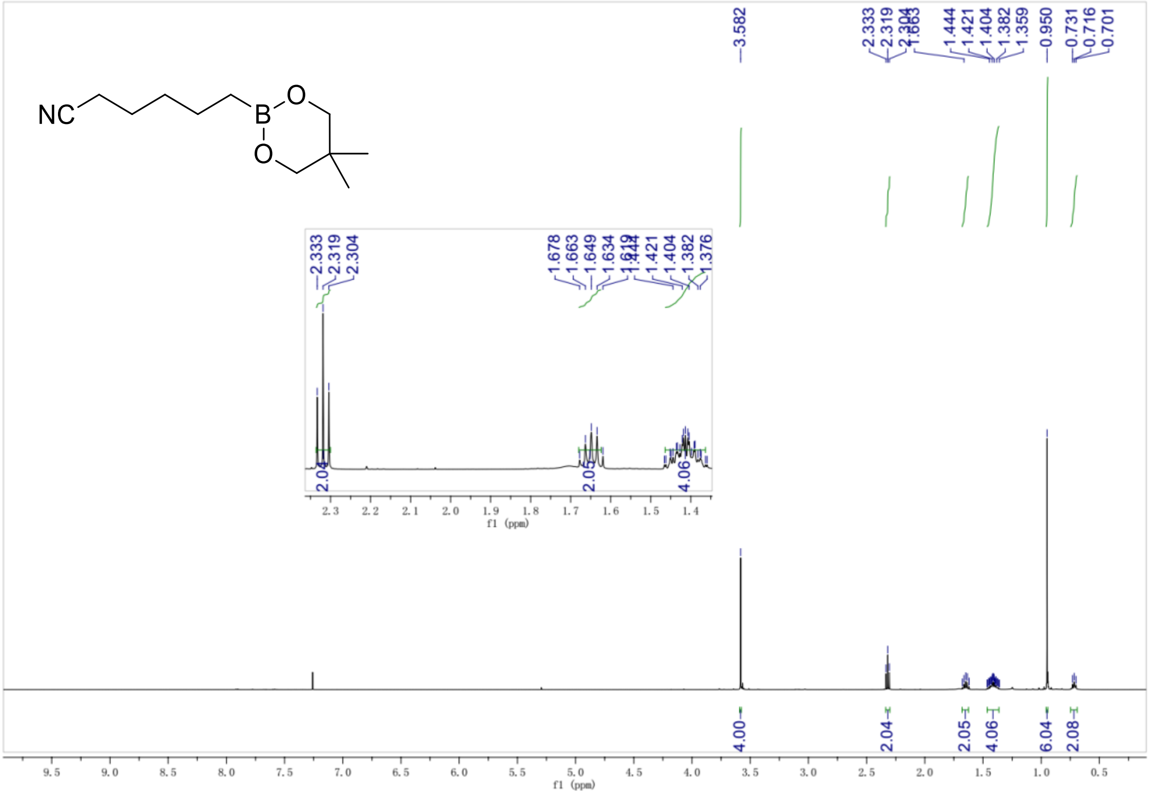

^{1}H NMR spectrum of compound **19b** in $CDCl_3$ (500 MHz).

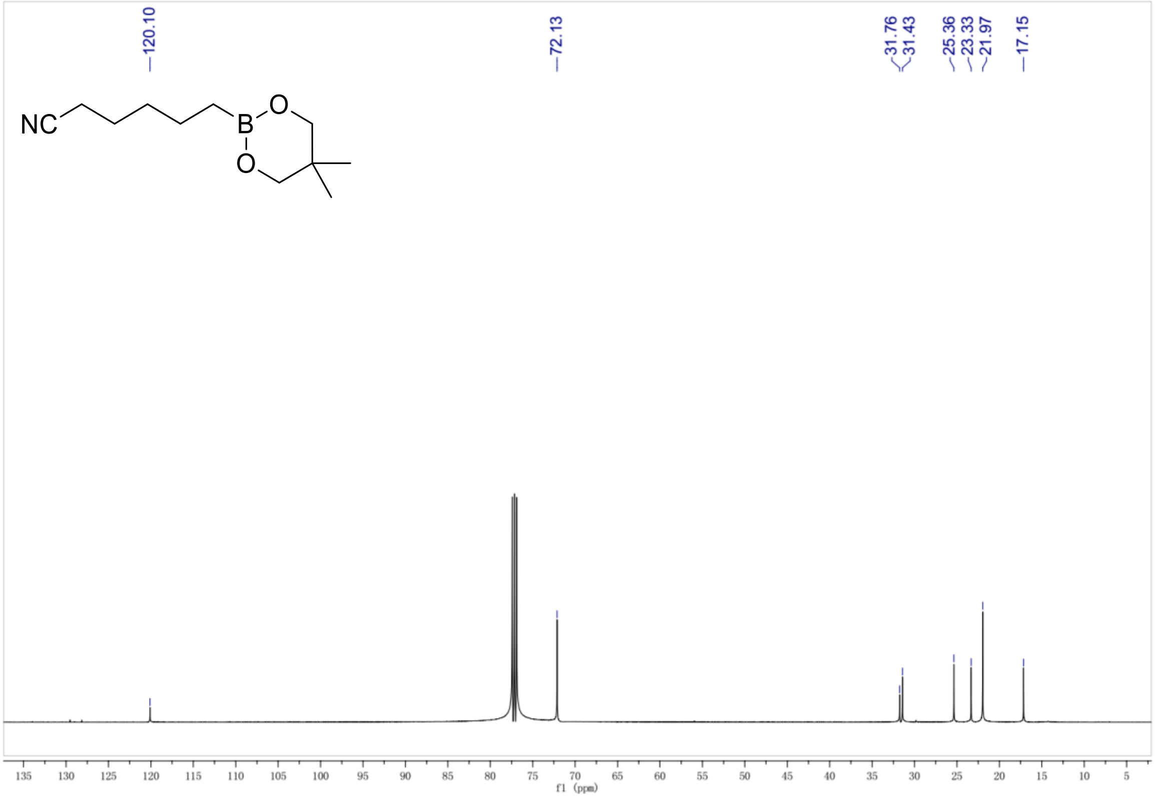

$^{13}C\{^1H\}$ NMR spectrum of compound **19b** in $CDCl_3$ (125 MHz).

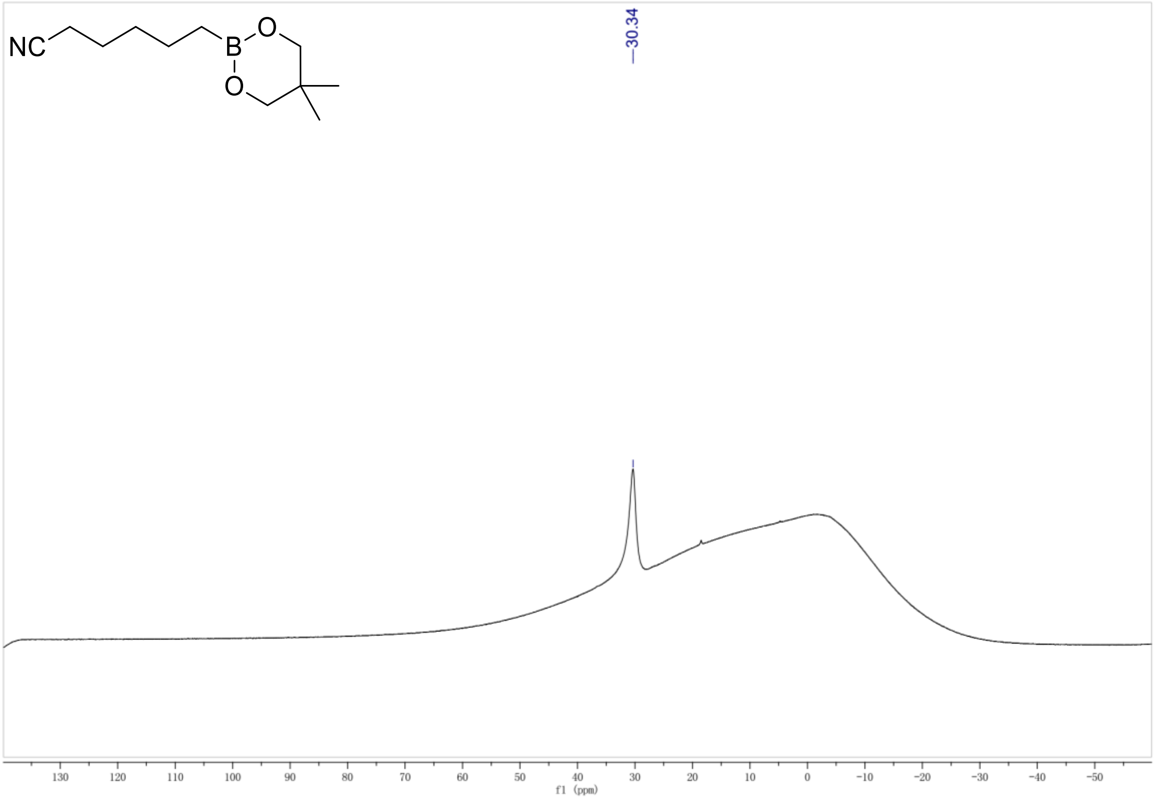

$^{11}B\{^1H\}$ NMR spectrum of compound **19b** in $CDCl_3$ (160 MHz).

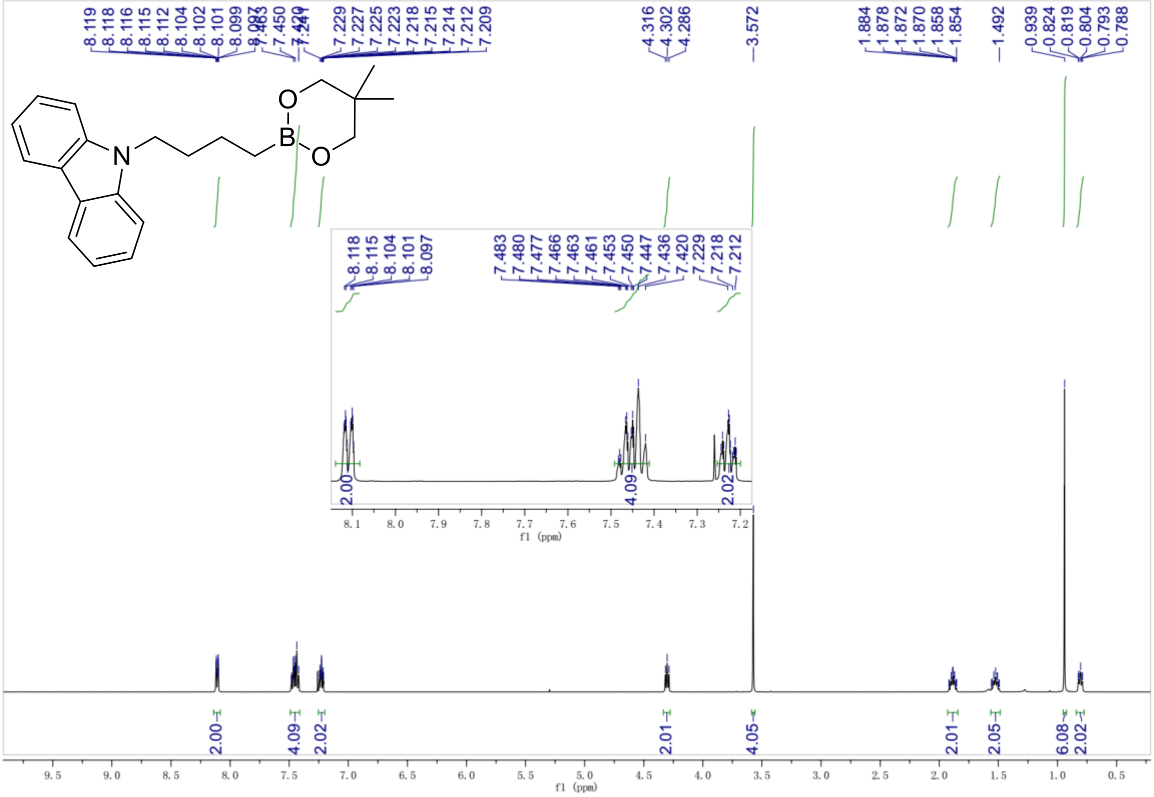

^{1}H NMR spectrum of compound **20b** in $CDCl_3$ (500 MHz).

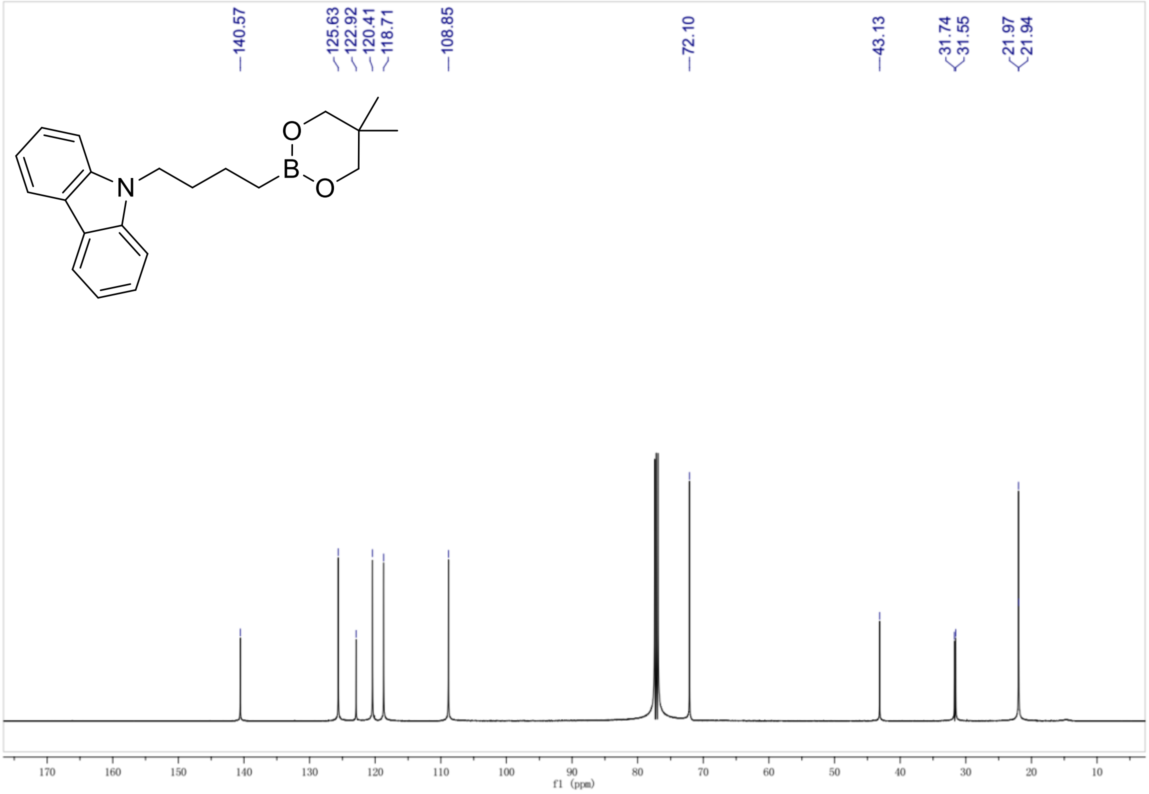

^{13}C{^{1}H} NMR spectrum of compound **20b** in $CDCl_3$ (125 MHz).

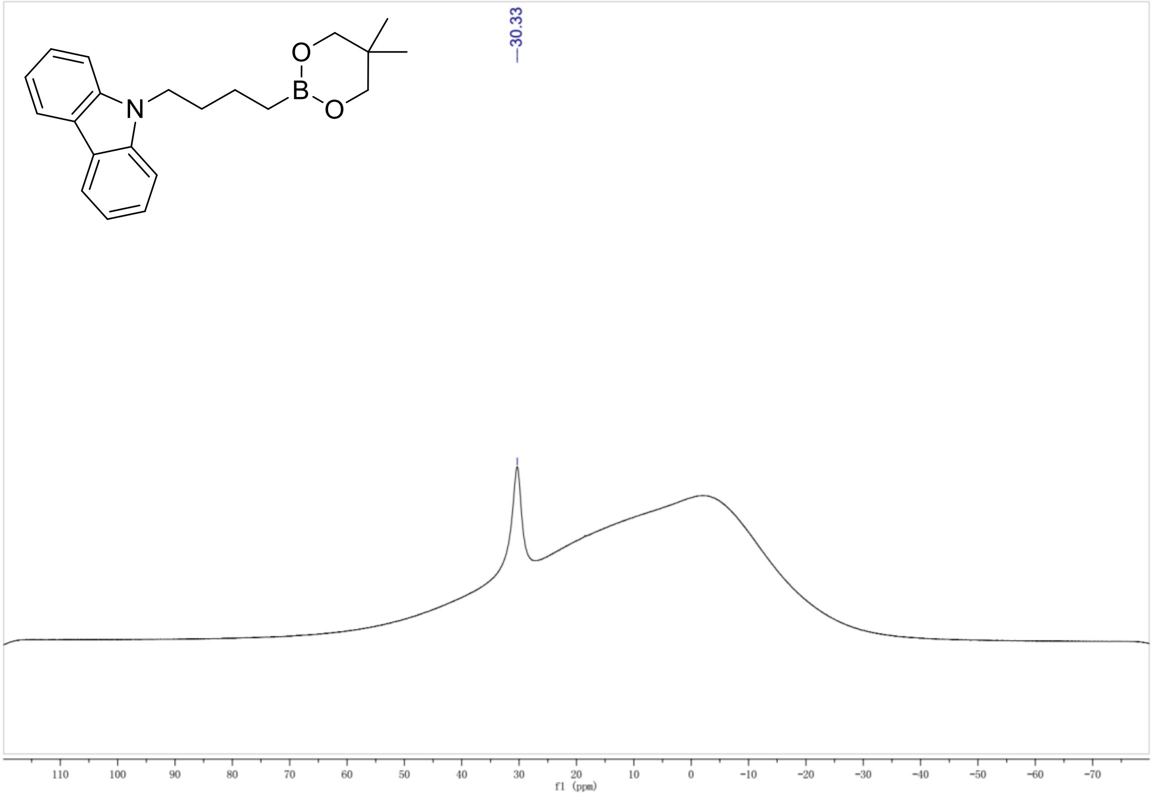

$^{11}B\{^{1}H\}$ NMR spectrum of compound **20b** in $CDCl_3$ (160 MHz).

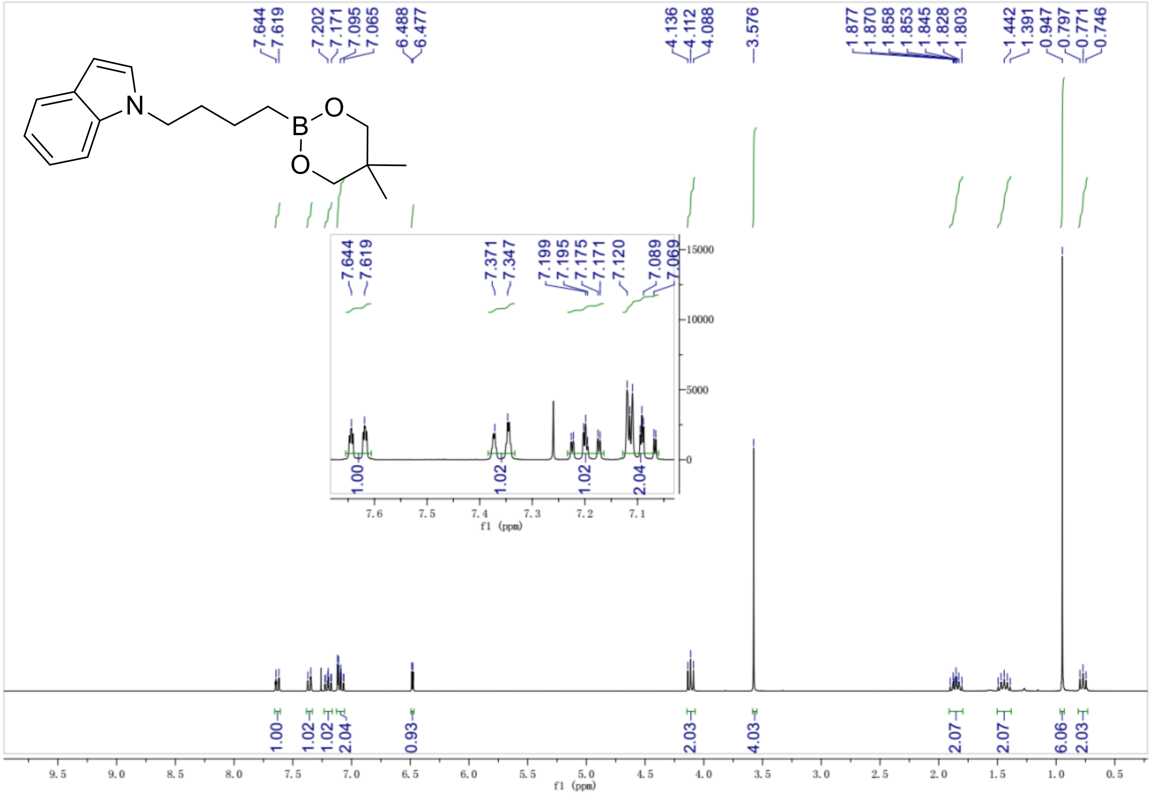

^{1}H NMR spectrum of compound **21b** in $CDCl_3$ (300 MHz).

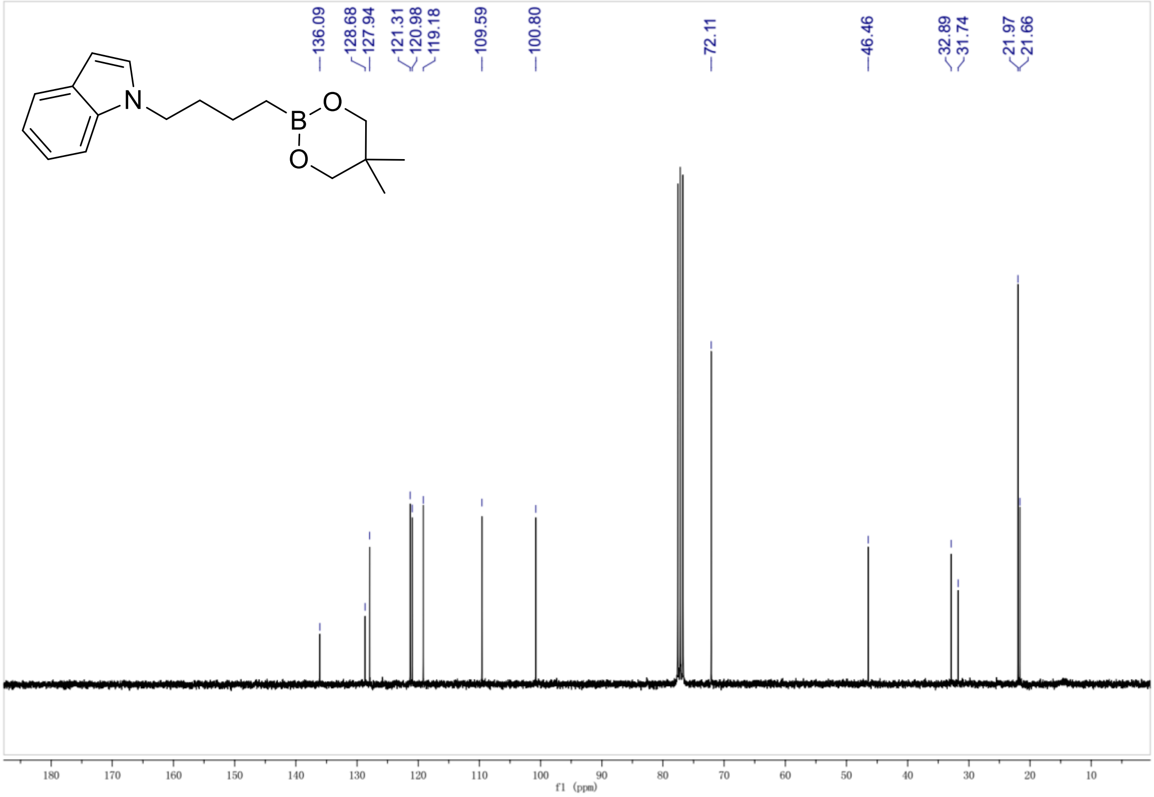

$^{13}C\{^1H\}$ NMR spectrum of compound **21b** in $CDCl_3$ (75 MHz).

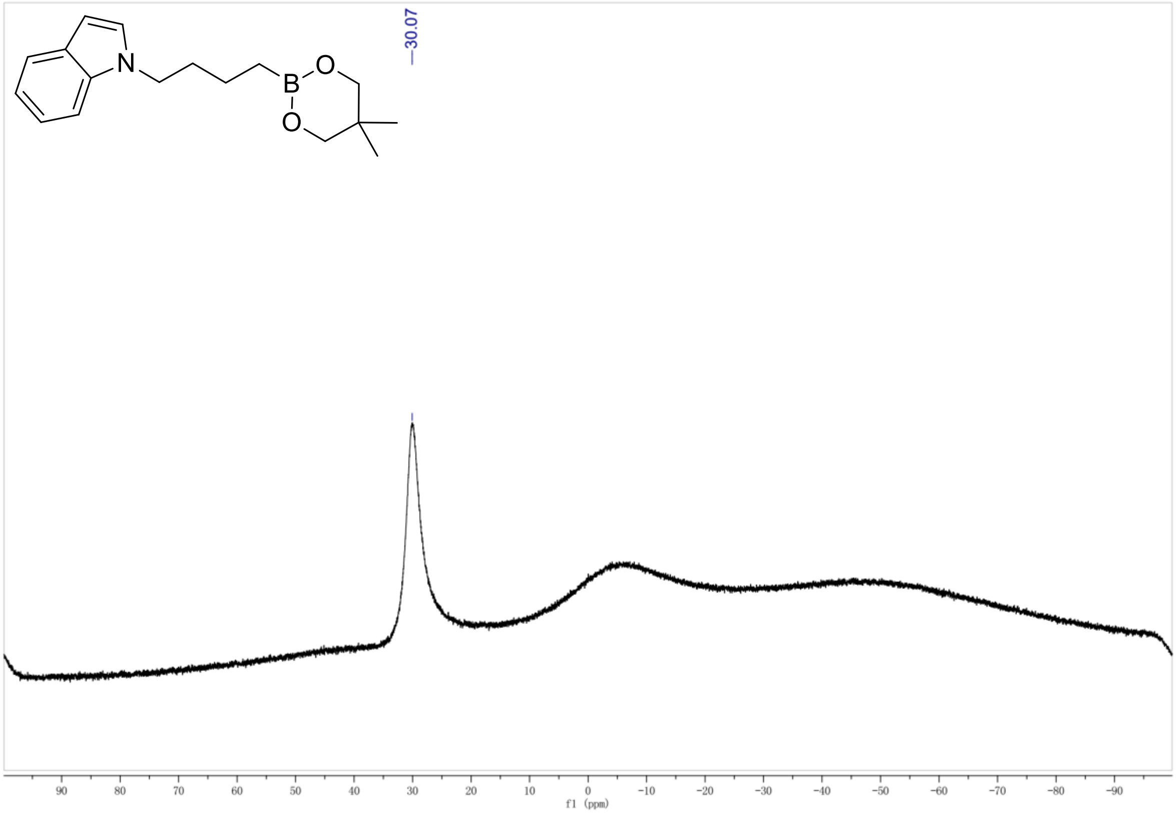

$^{11}B\{^1H\}$ NMR spectrum of compound **21b** in $CDCl_3$ (96 MHz).

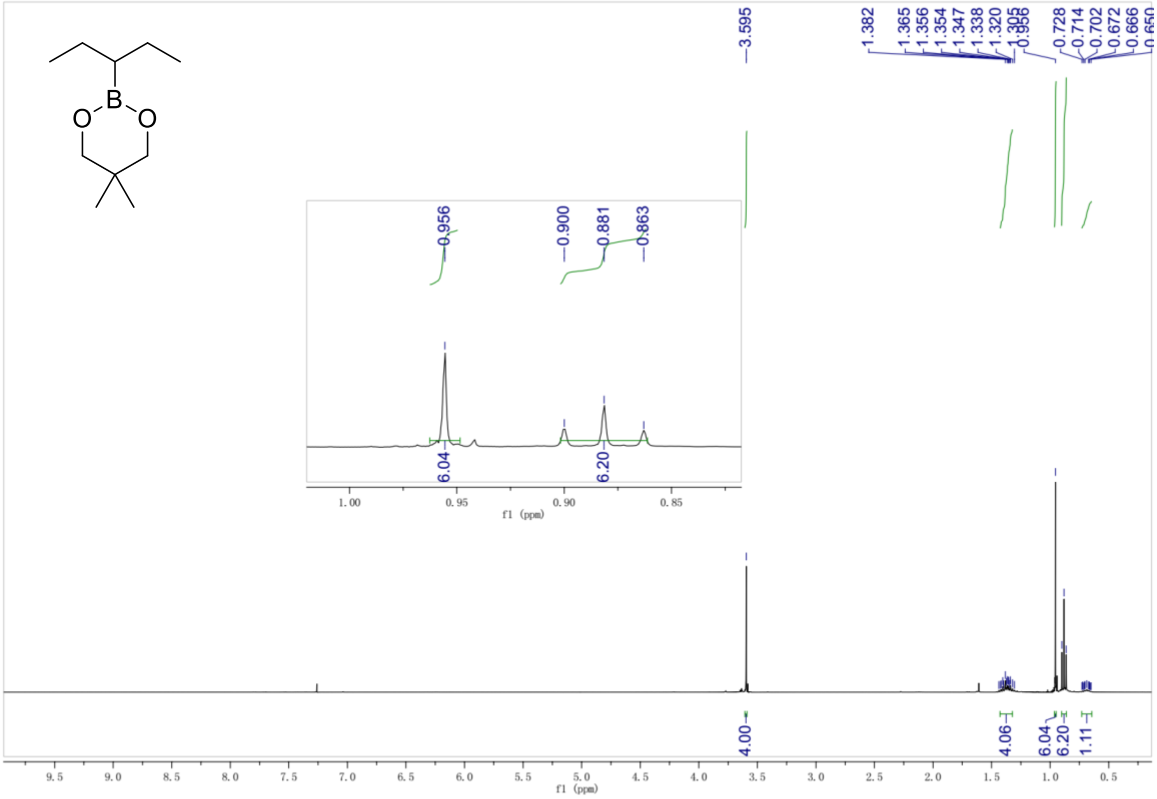

^{1}H NMR spectrum of compound **22b** in $CDCl_3$ (400 MHz).

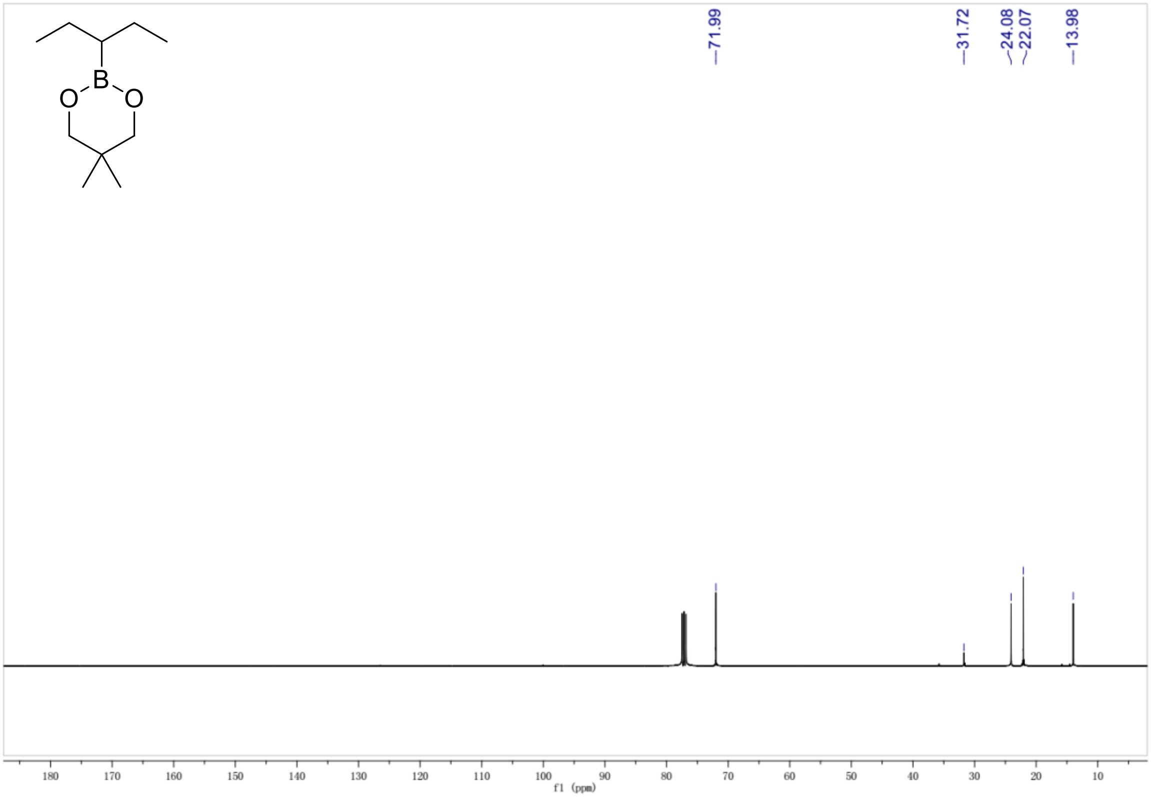

$^{13}C\{^1H\}$ NMR spectrum of compound **22b** in $CDCl_3$ (100 MHz).

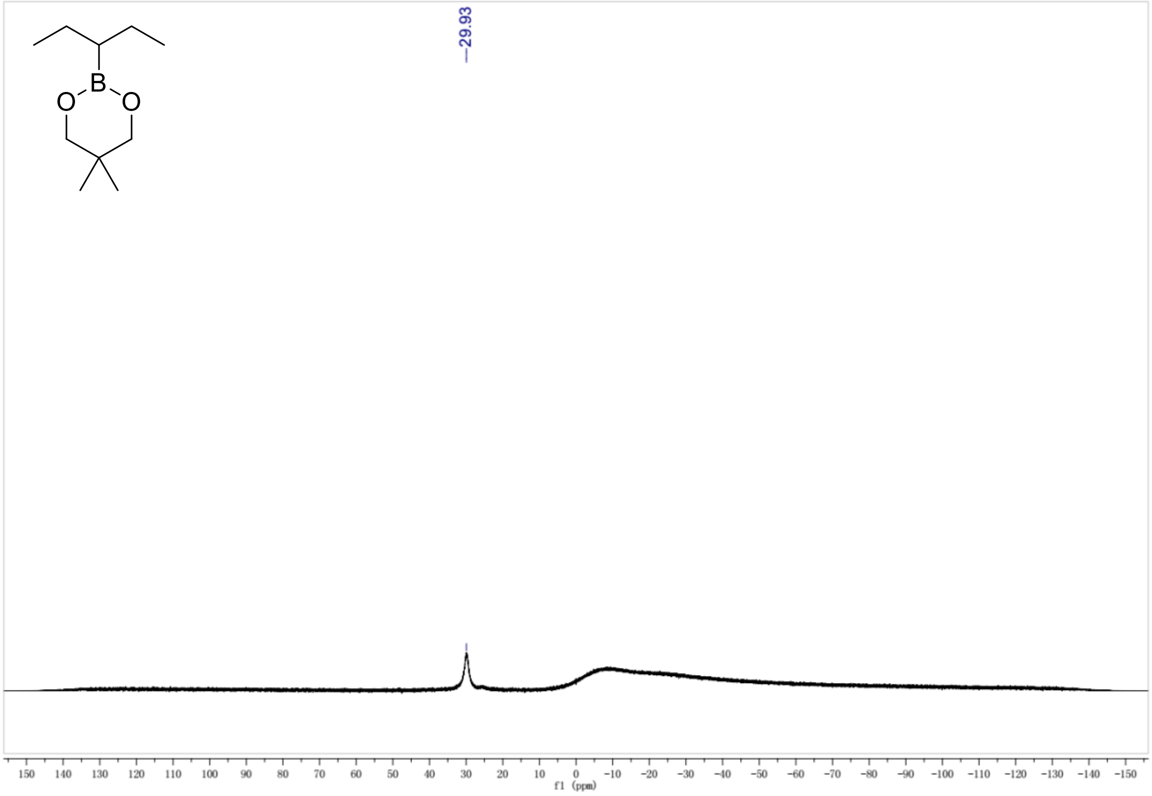

$^{11}B\{^{1}H\}$ NMR spectrum of compound **22b** in $CDCl_3$ (128 MHz).

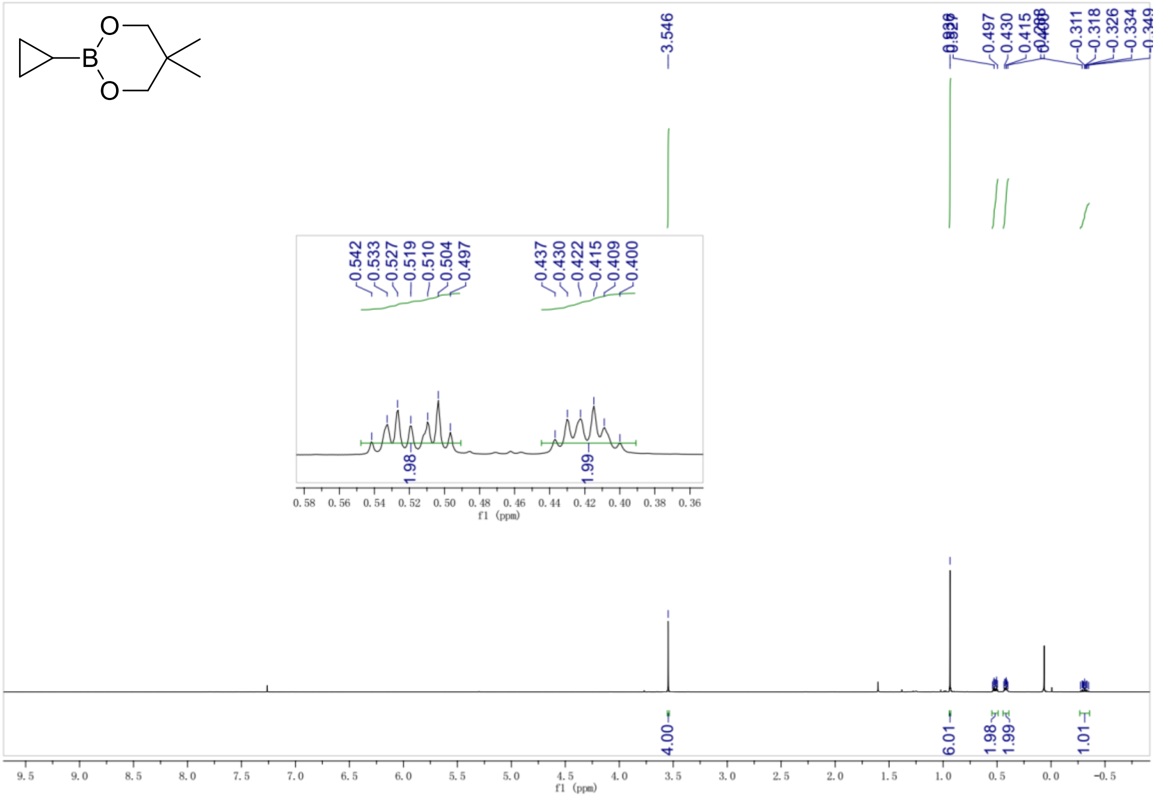

^{1}H NMR spectrum of compound **23b** in $CDCl_3$ (400 MHz).

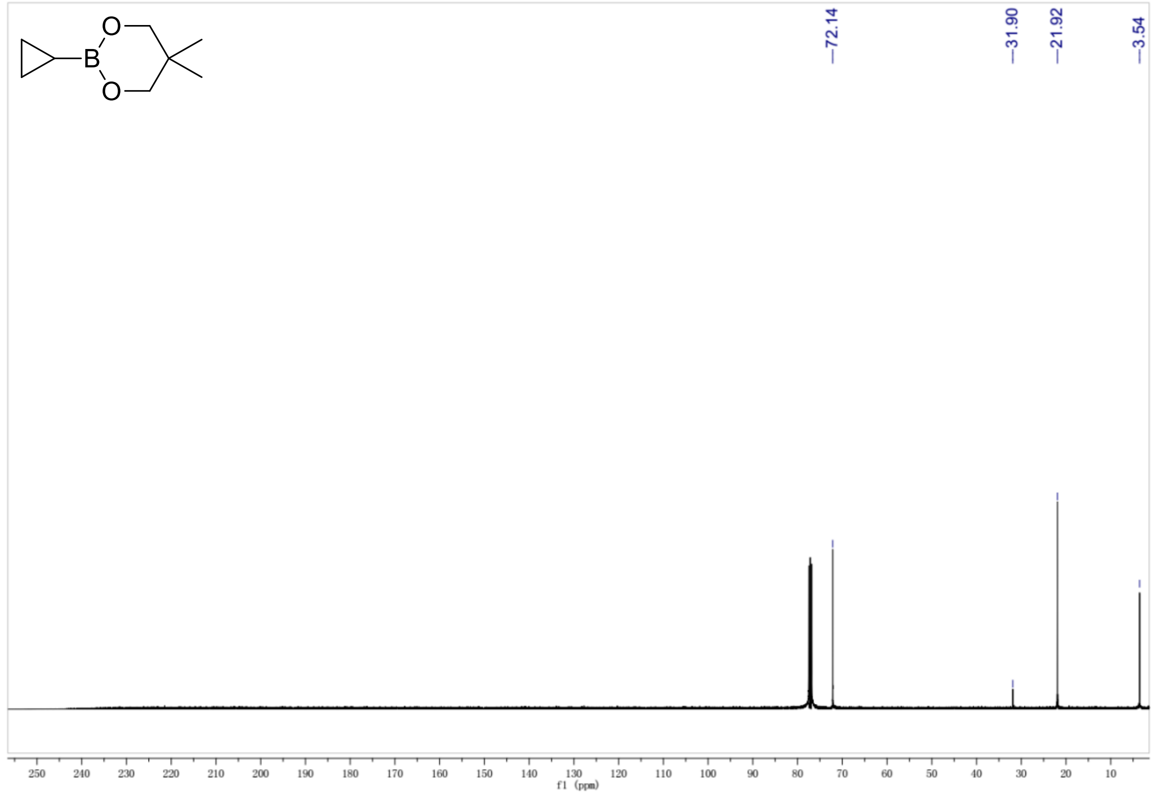

$^{13}C\{^{1}H\}$ NMR spectrum of compound **23b** in $CDCl_3$ (100 MHz).

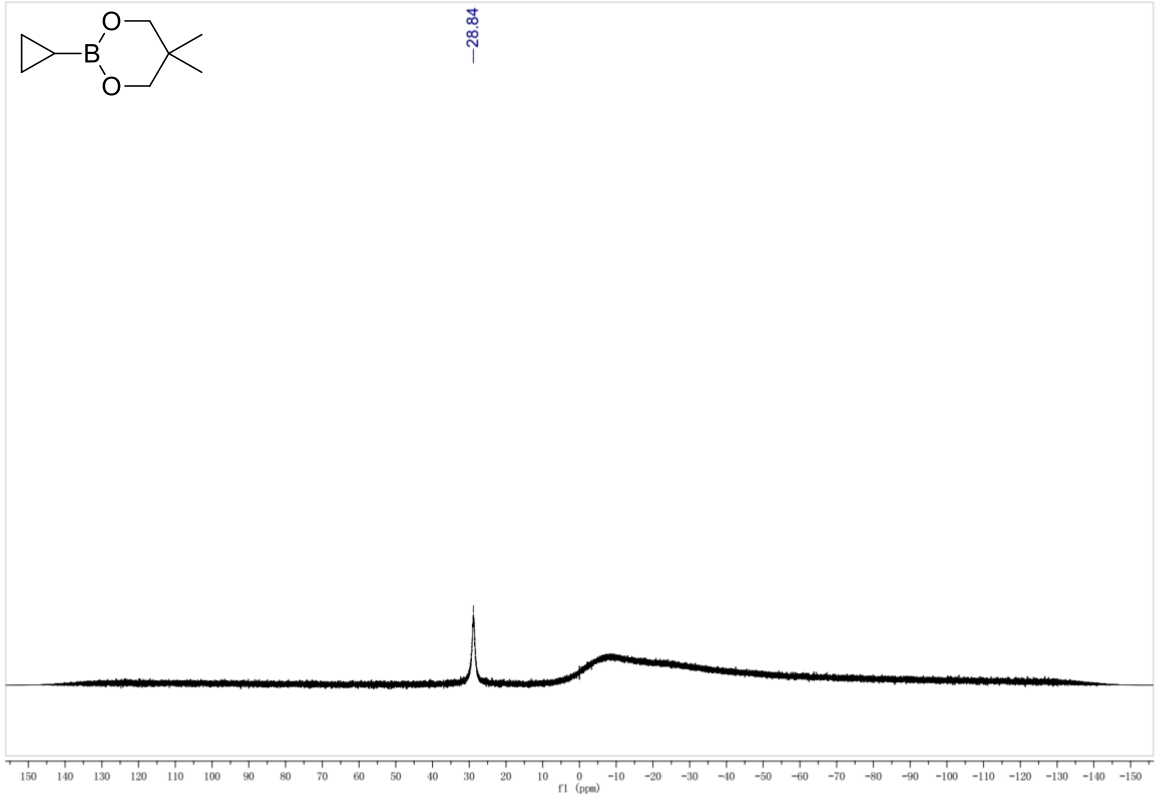

$^{11}B\{^{1}H\}$ NMR spectrum of compound **23b** in $CDCl_3$ (128 MHz).

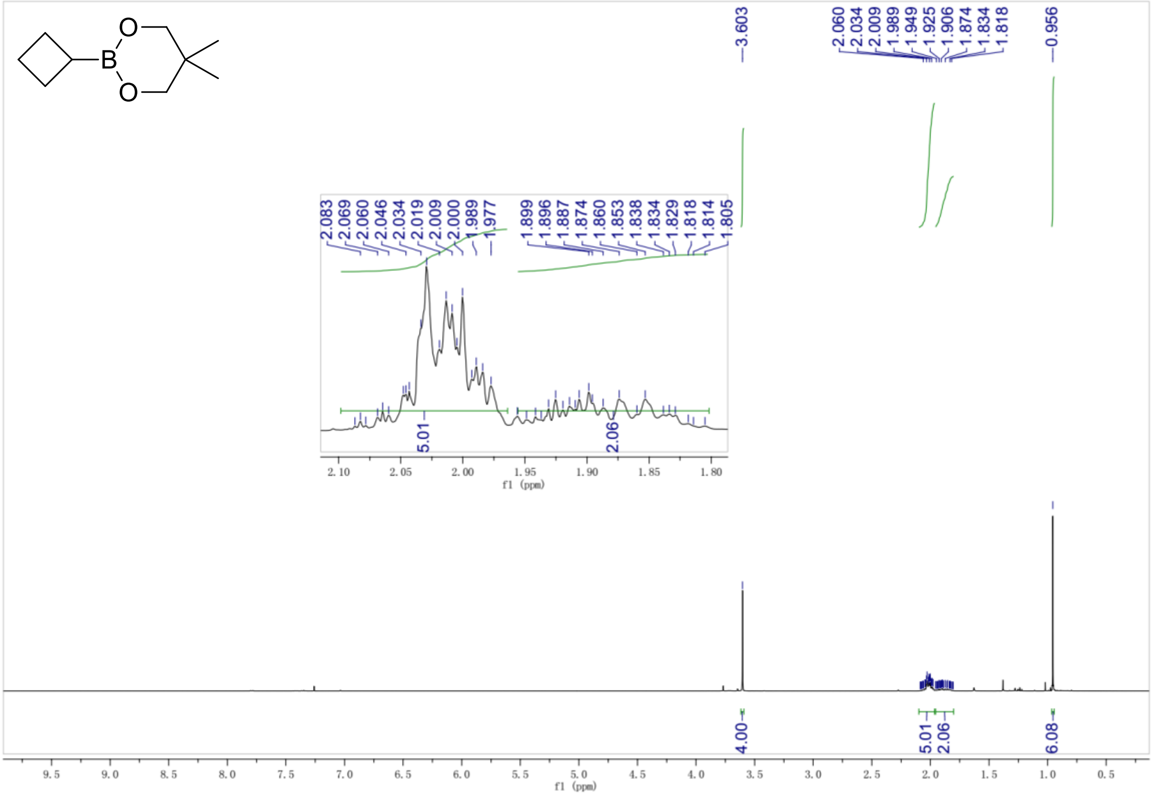

^{1}H NMR spectrum of compound **24b** in $CDCl_3$ (400 MHz).

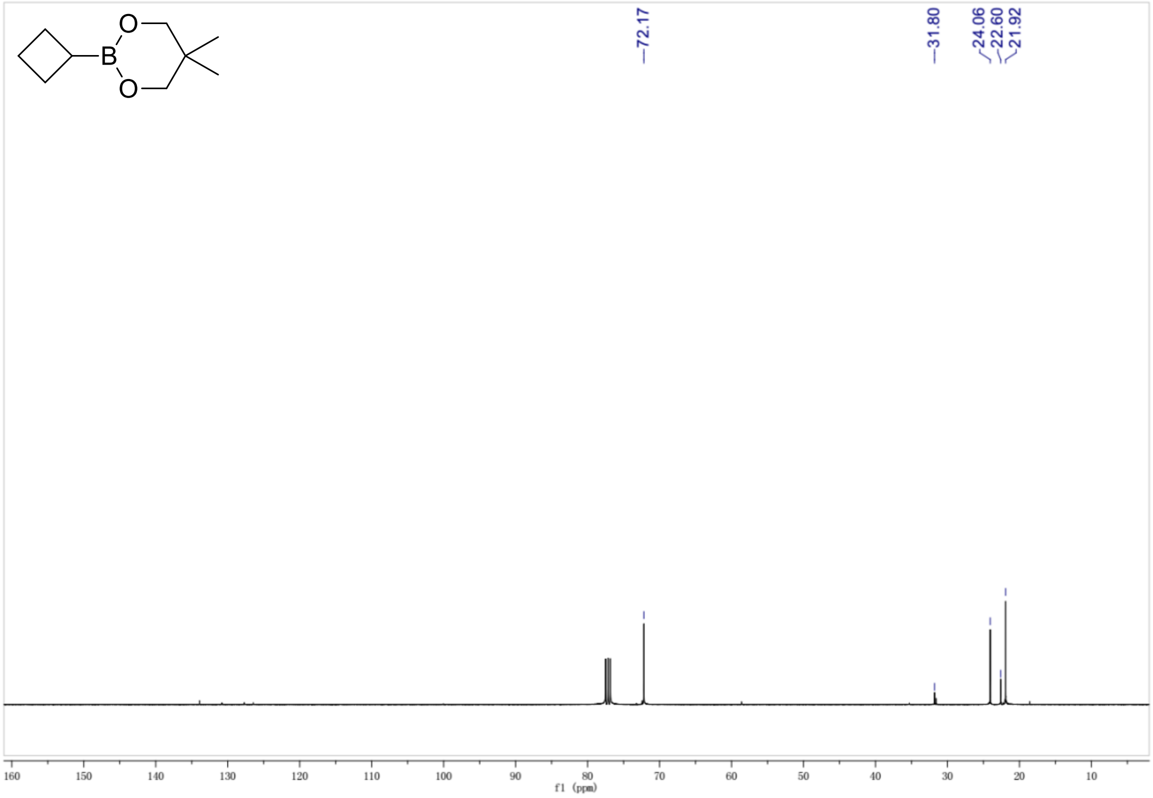

$^{13}C\{^{1}H\}$ NMR spectrum of compound **24b** in $CDCl_3$ (100 MHz).

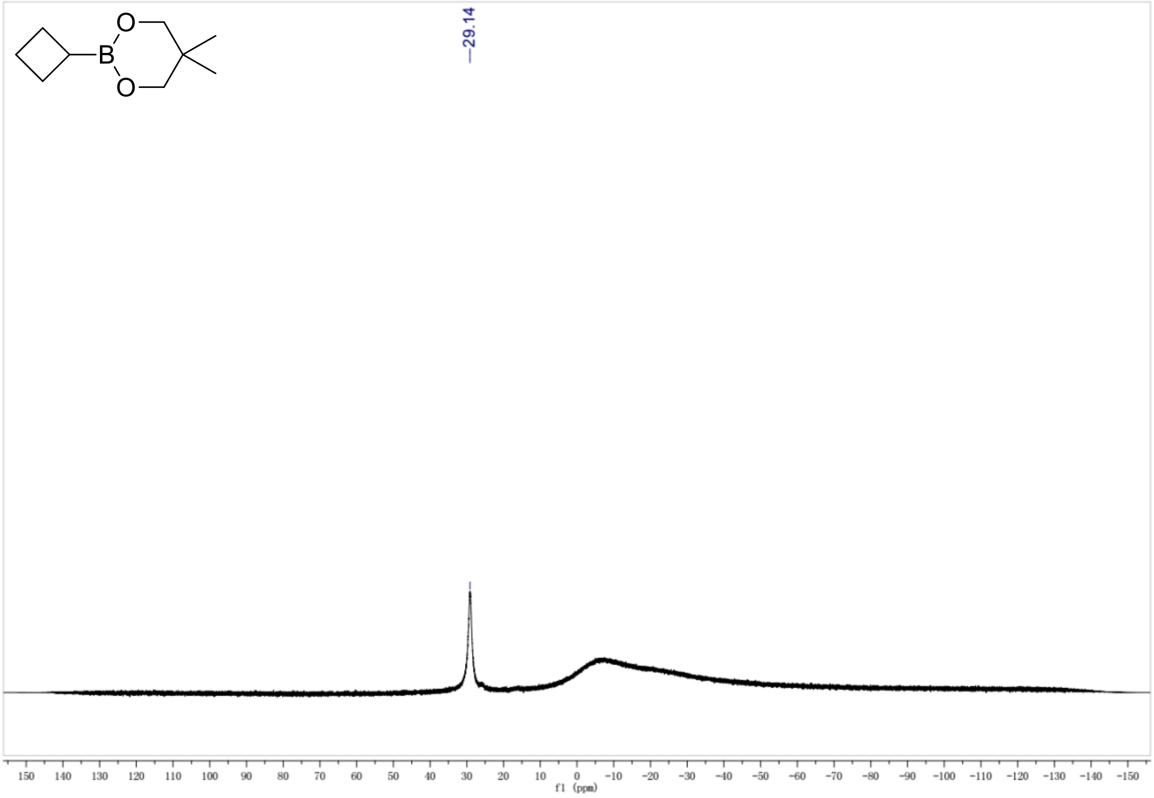

$^{11}B\{^{1}H\}$ NMR spectrum of compound **24b** in $CDCl_3$ (128 MHz).

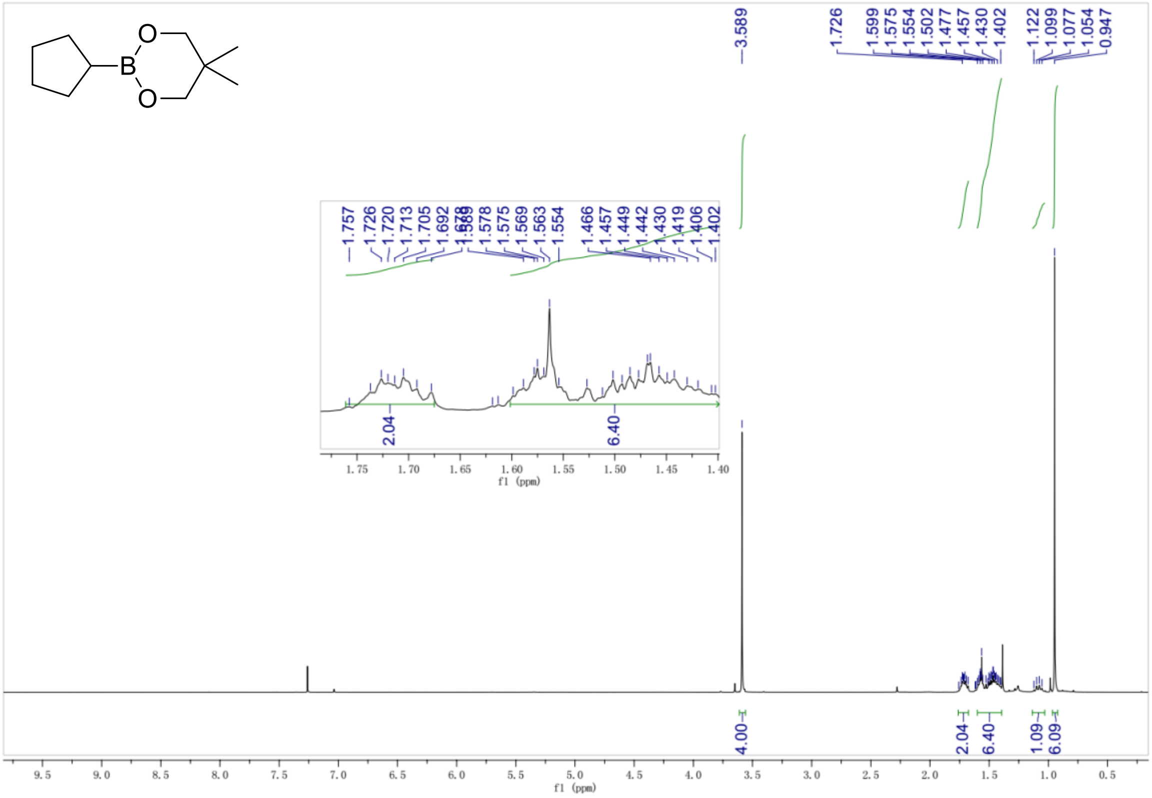

^{1}H NMR spectrum of compound **25b** in $CDCl_3$ (400 MHz).

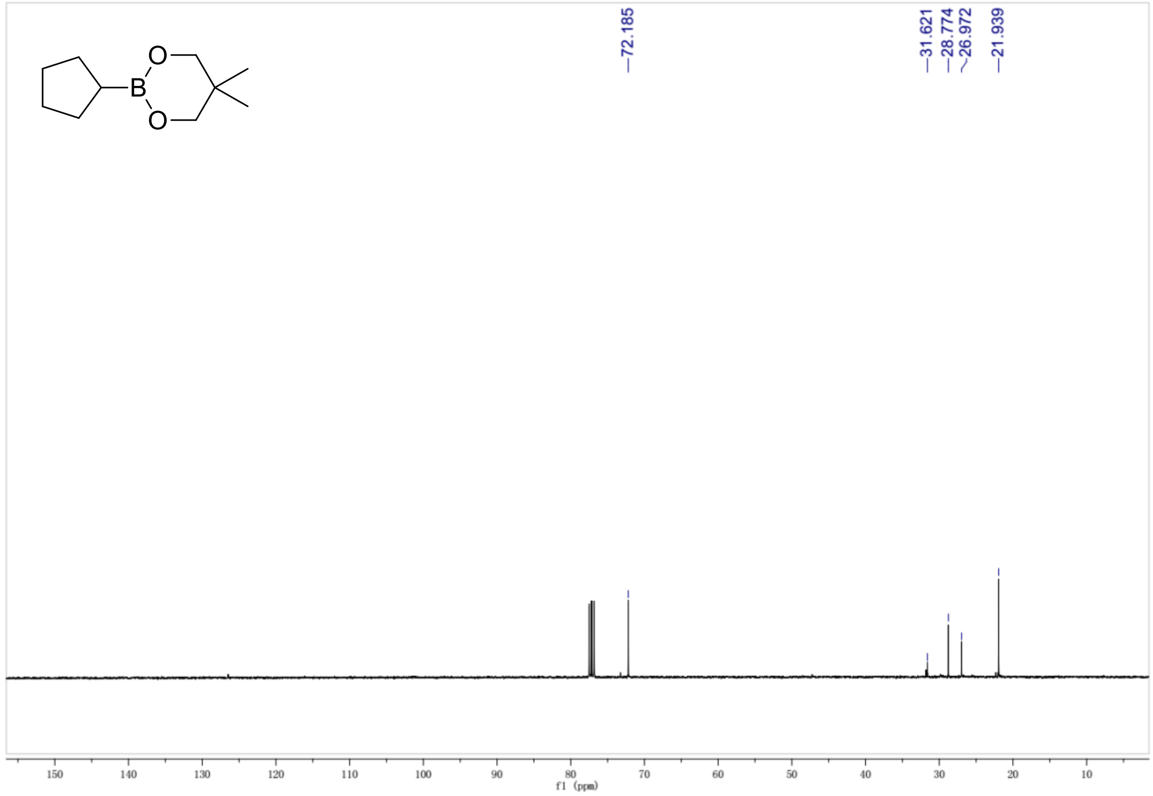

$^{13}C\{^1H\}$ NMR spectrum of compound **25b** in $CDCl_3$ (100 MHz).

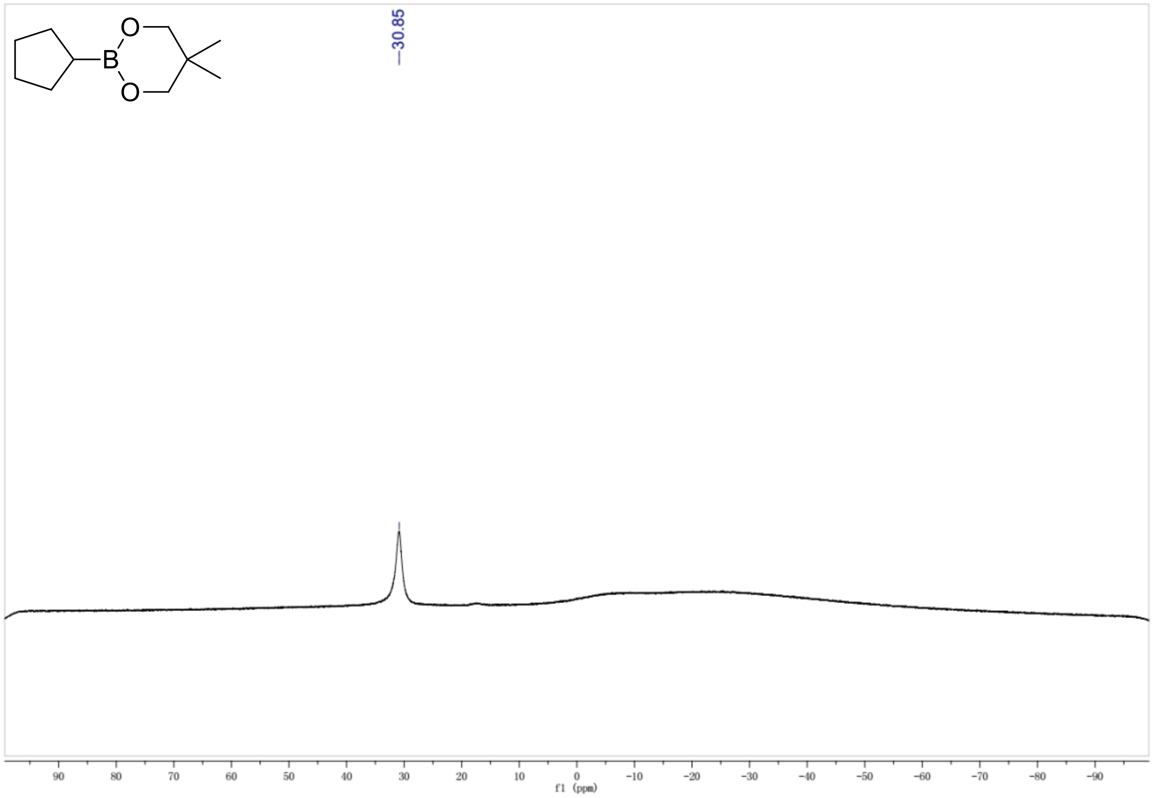

$^{11}B\{^1H\}$ NMR spectrum of compound **25b** in $CDCl_3$ (128 MHz).

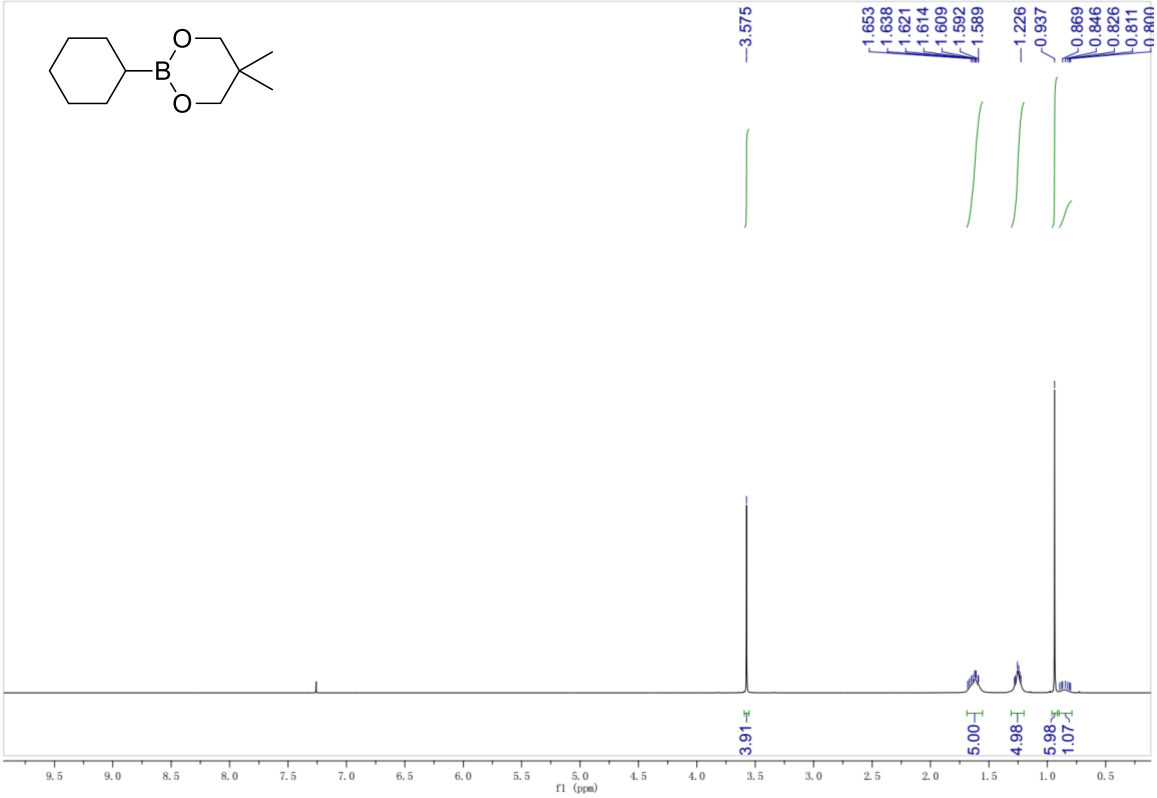

^{1}H NMR spectrum of compound **26b** in $CDCl_3$ (300 MHz).

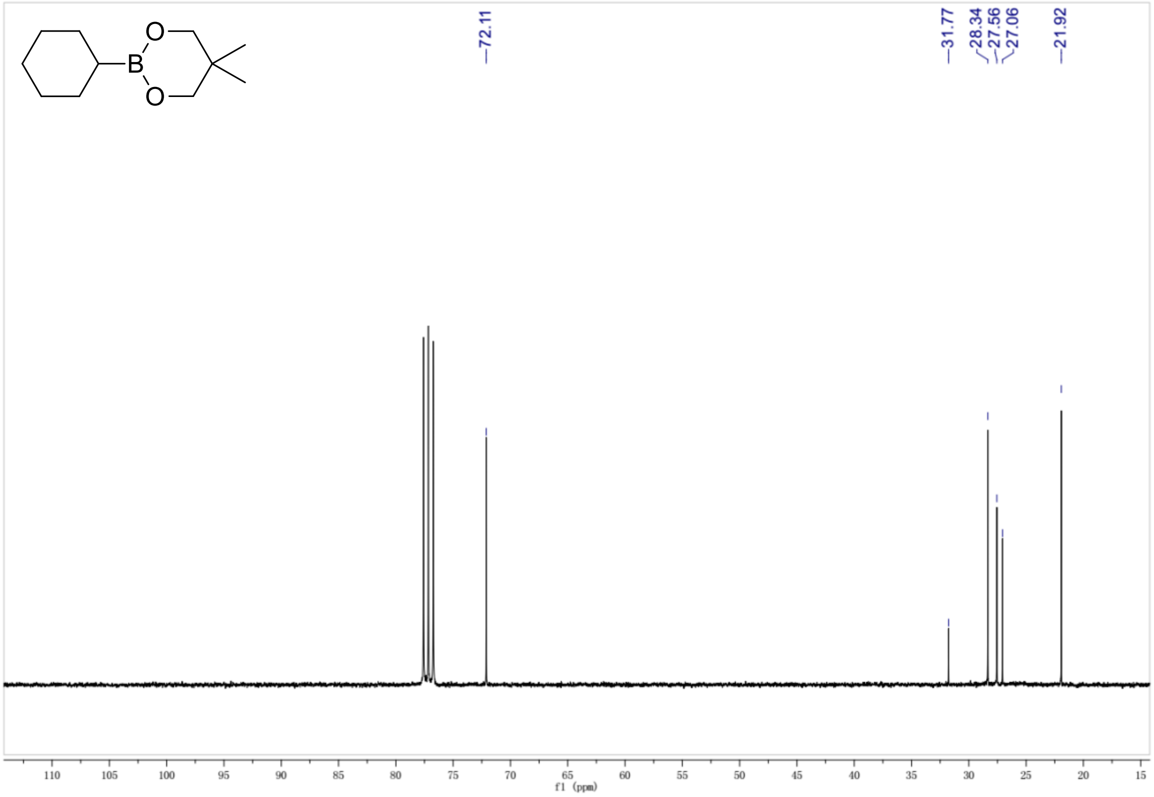

^{13}C{^{1}H} NMR spectrum of compound **26b** in $CDCl_3$ (75 MHz).

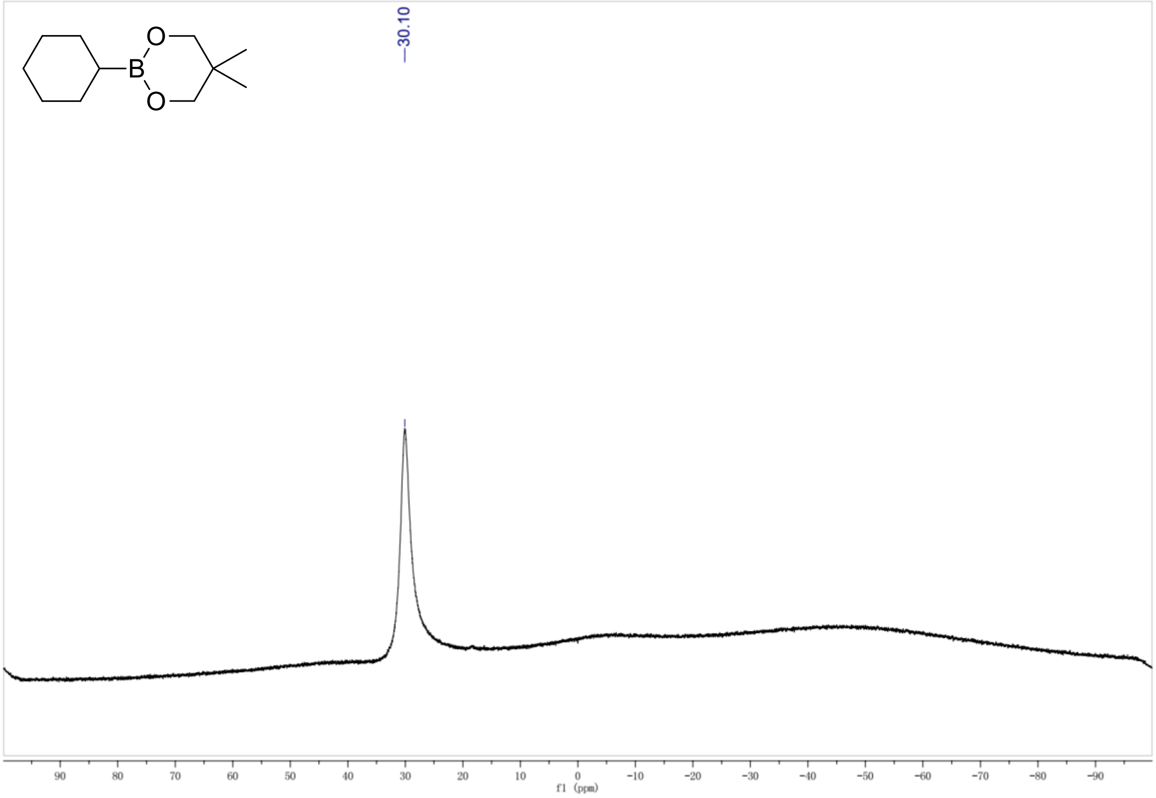

^{11}B{^{1}H} NMR spectrum of compound **26b** in $CDCl_3$ (96 MHz).

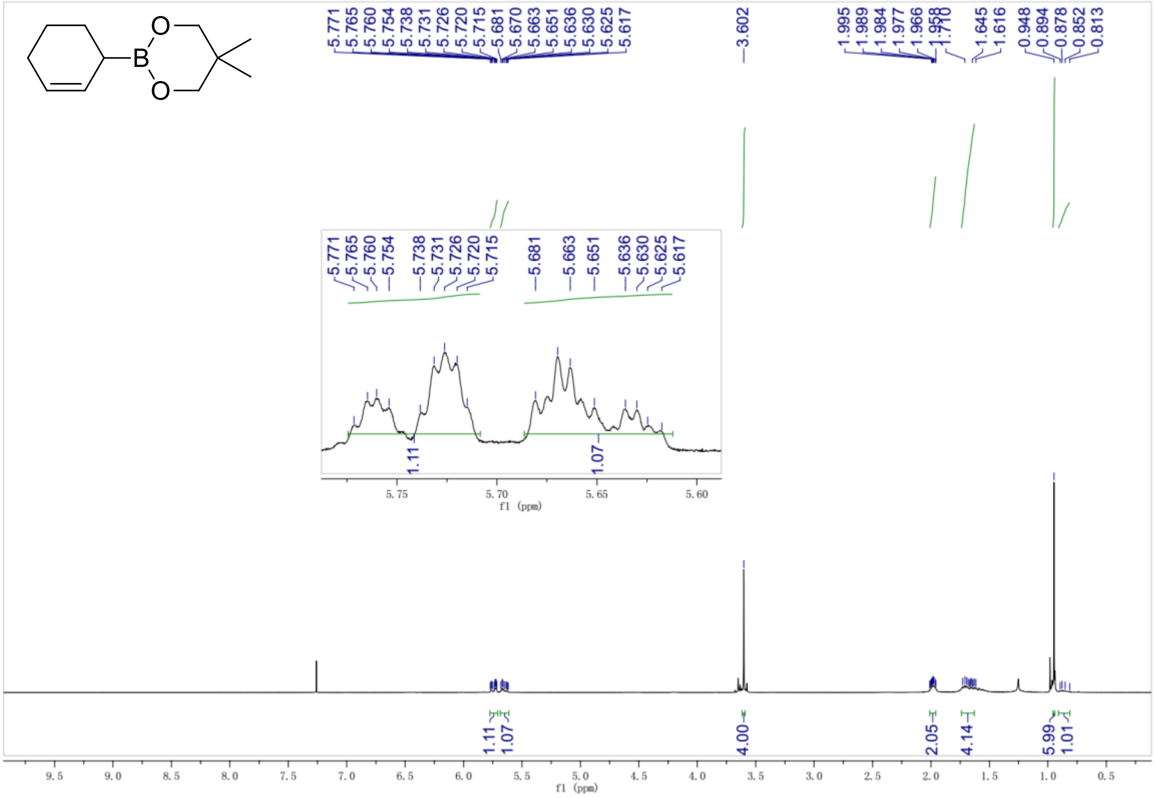

^{1}H NMR spectrum of compound **27b** in $CDCl_3$ (300 MHz).

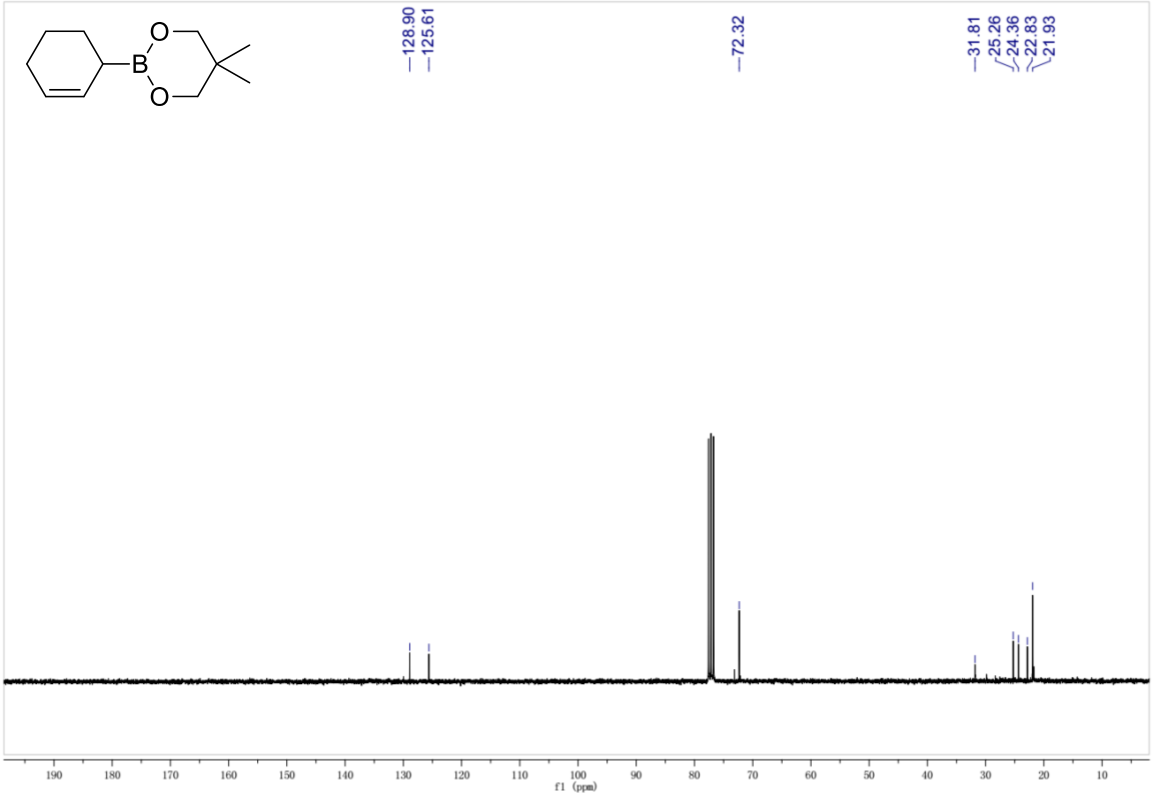

$^{13}C\{^{1}H\}$ NMR spectrum of compound **27b** in $CDCl_3$ (75 MHz).

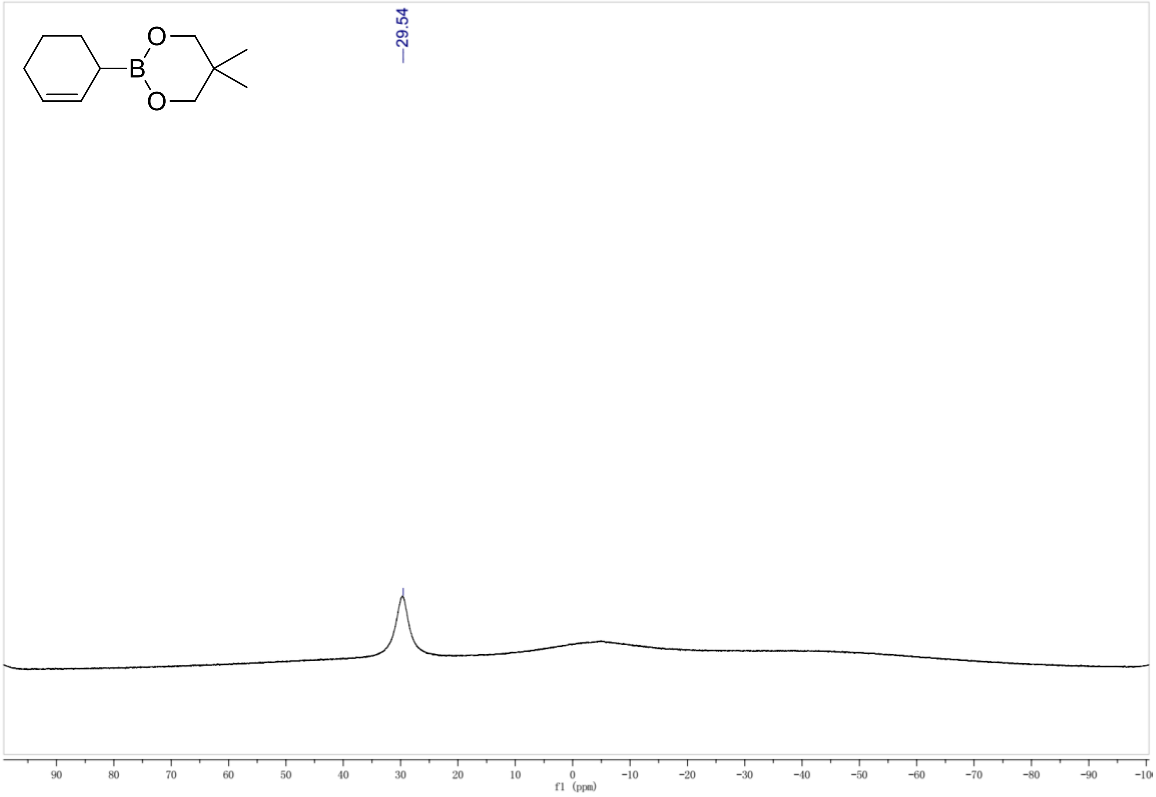

$^{11}B\{^{1}H\}$ NMR spectrum of compound **27b** in $CDCl_3$ (96 MHz).

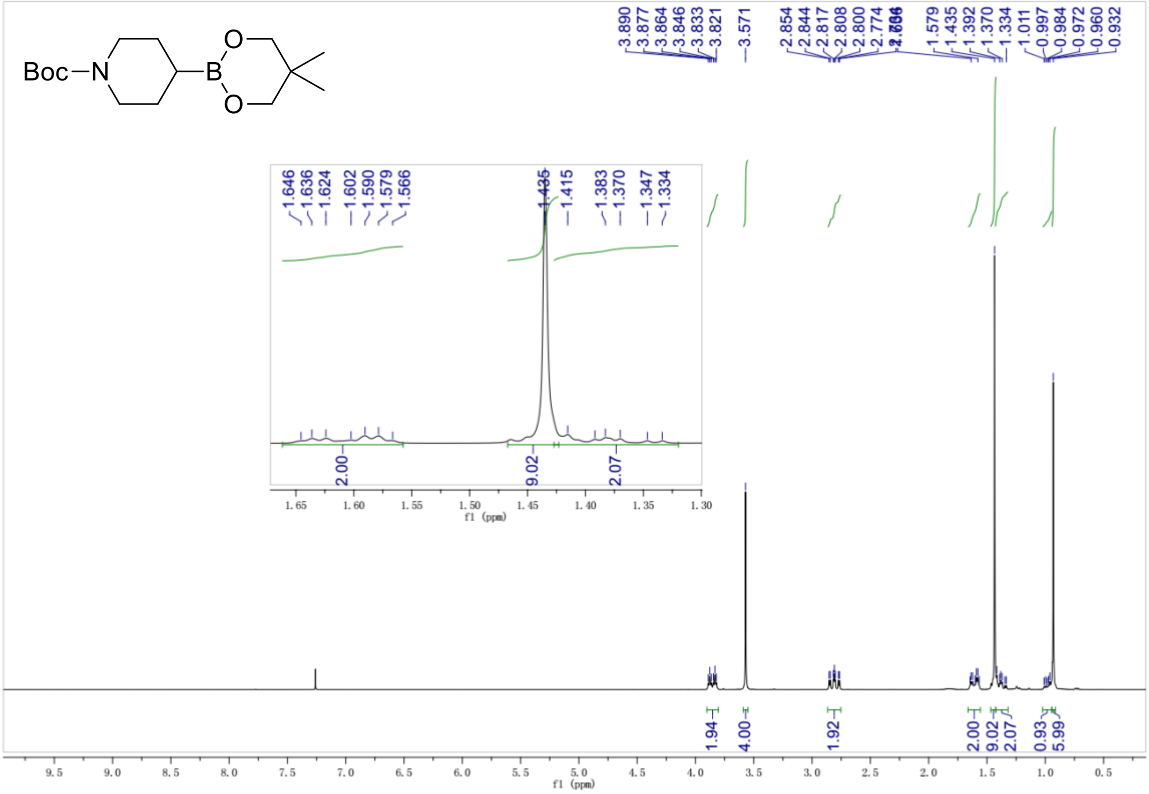

^{1}H NMR spectrum of compound **28b** in $CDCl_3$ (300 MHz).

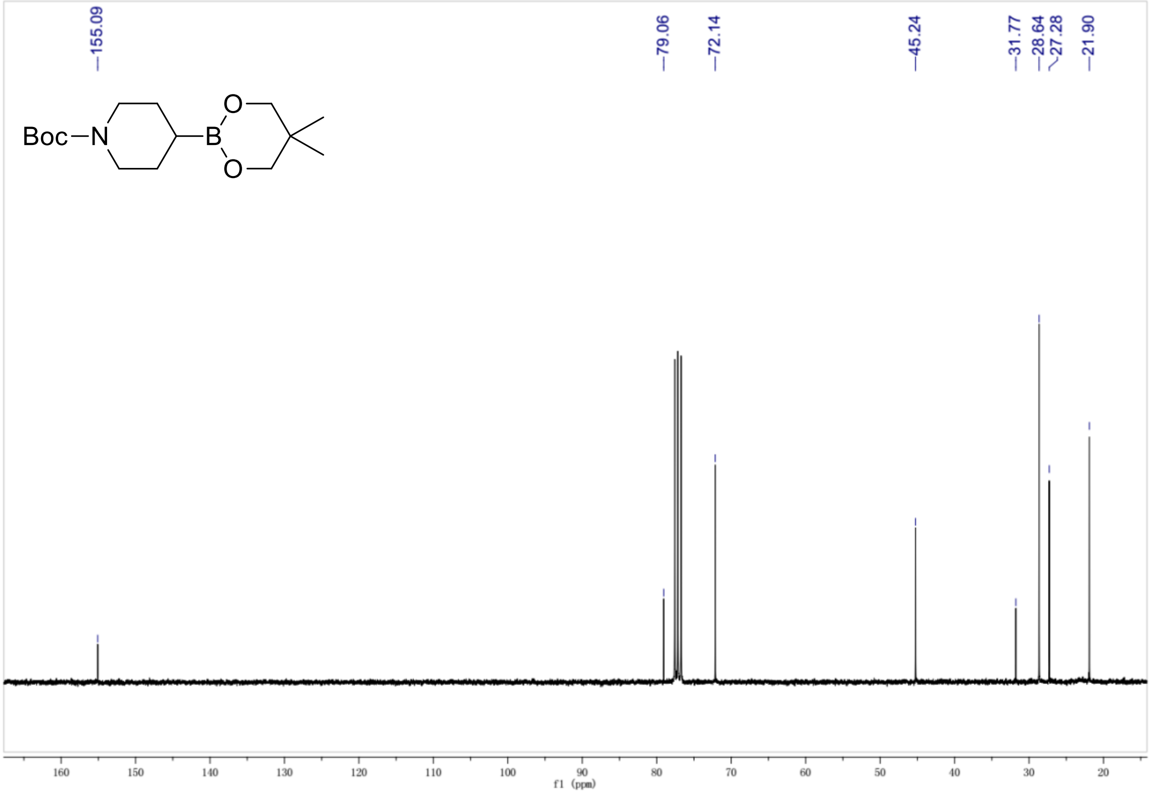

^{13}C{^{1}H} NMR spectrum of compound **28b** in $CDCl_3$ (75 MHz).

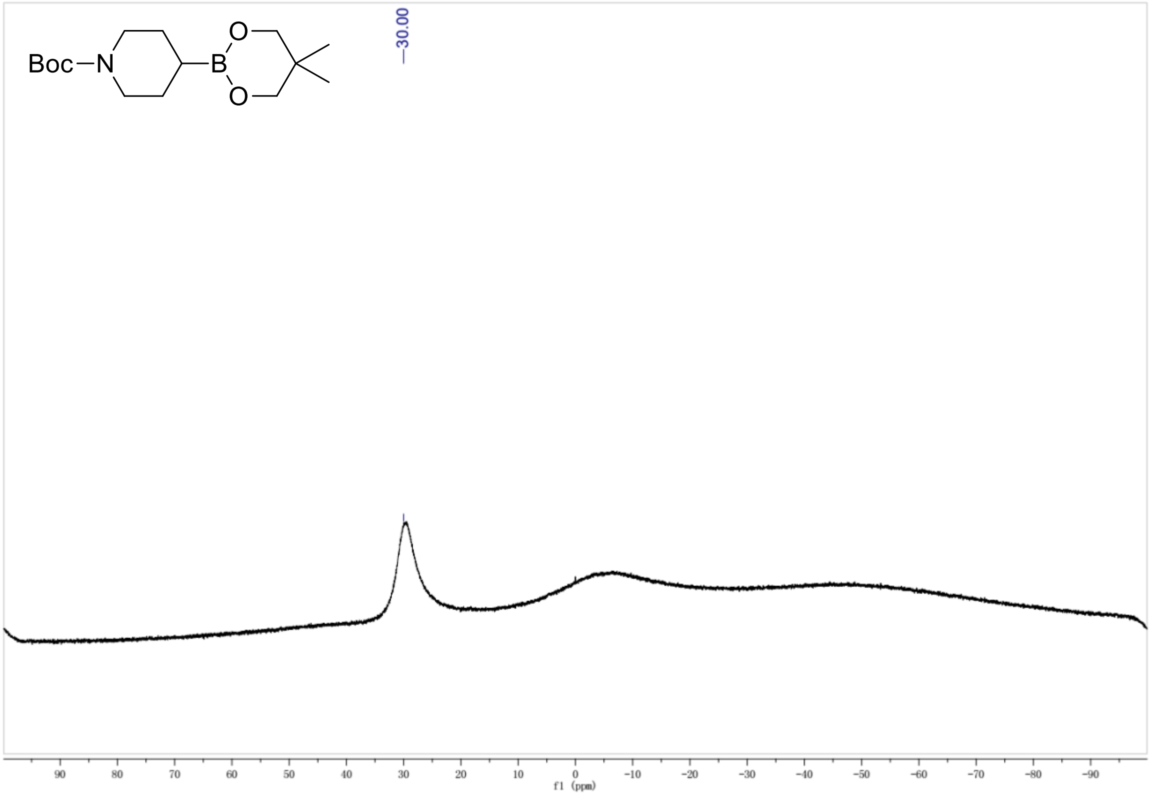

$^{11}B\{^{1}H\}$ NMR spectrum of compound **28b** in $CDCl_3$ (96 MHz).

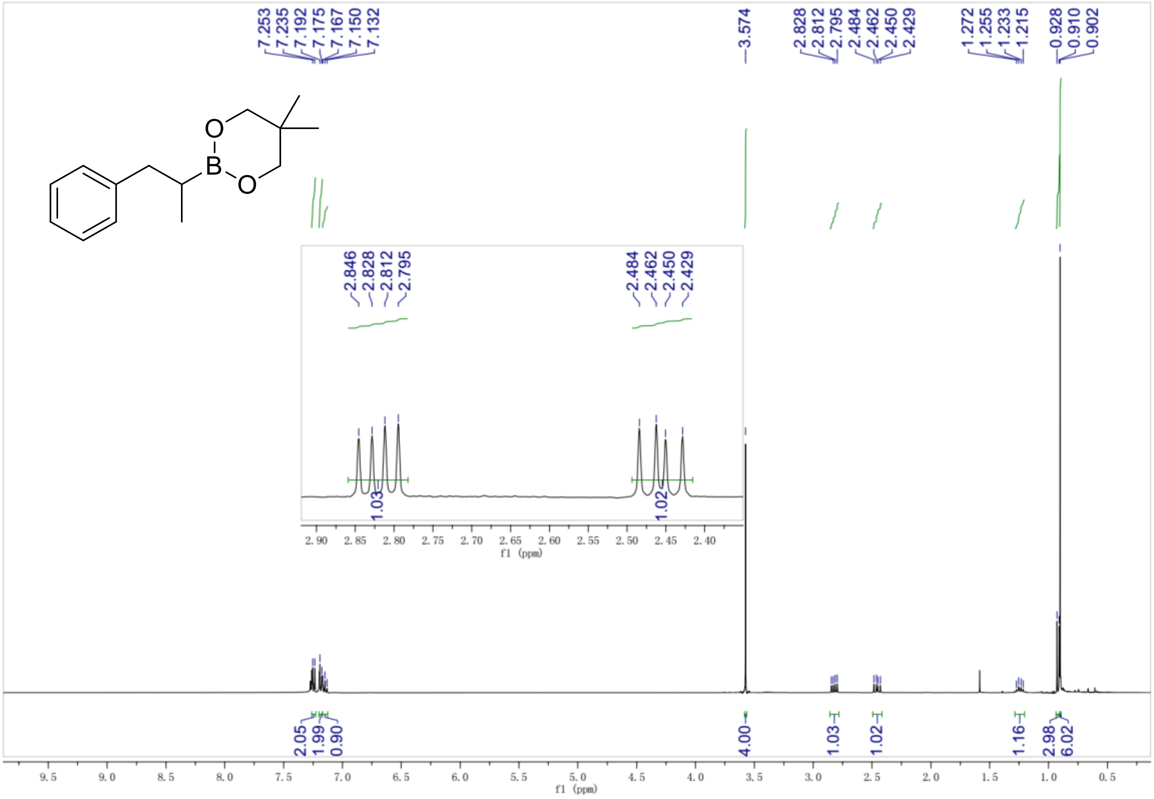

^{1}H NMR spectrum of compound **29b** in $CDCl_3$ (400 MHz).

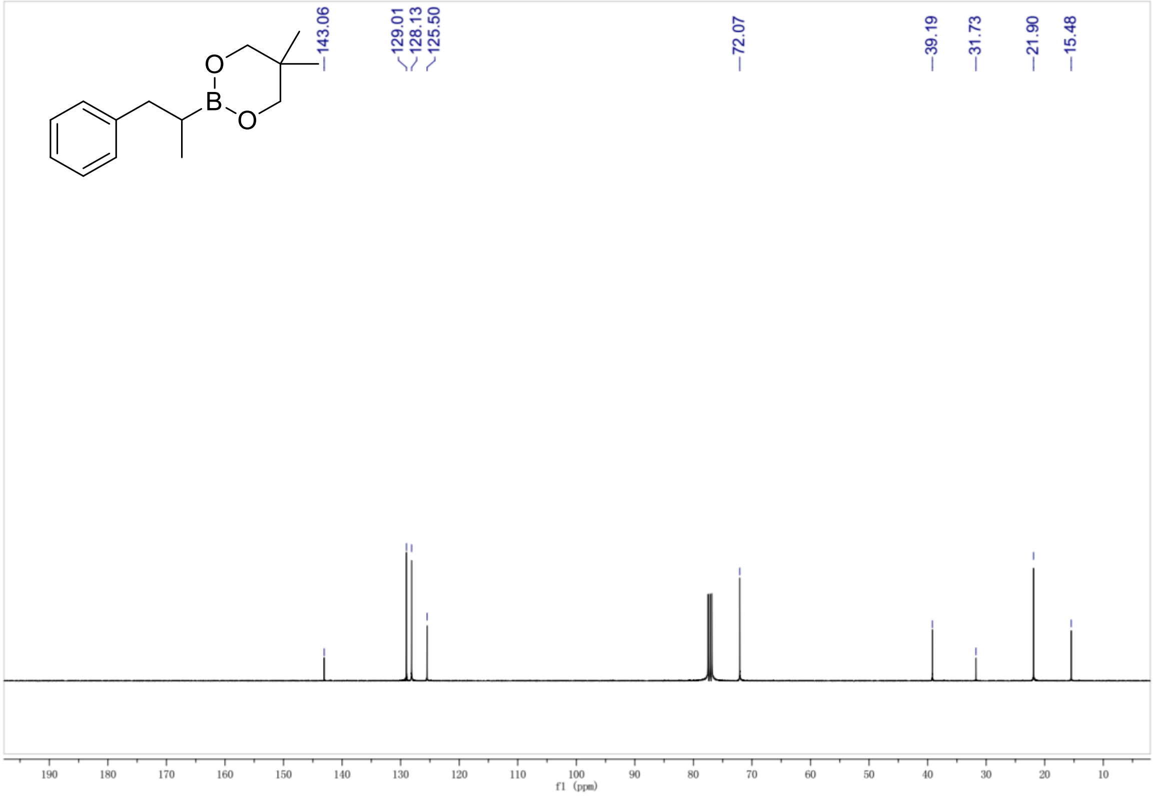

$^{13}C\{^{1}H\}$ NMR spectrum of compound **29b** in $CDCl_3$ (100 MHz).

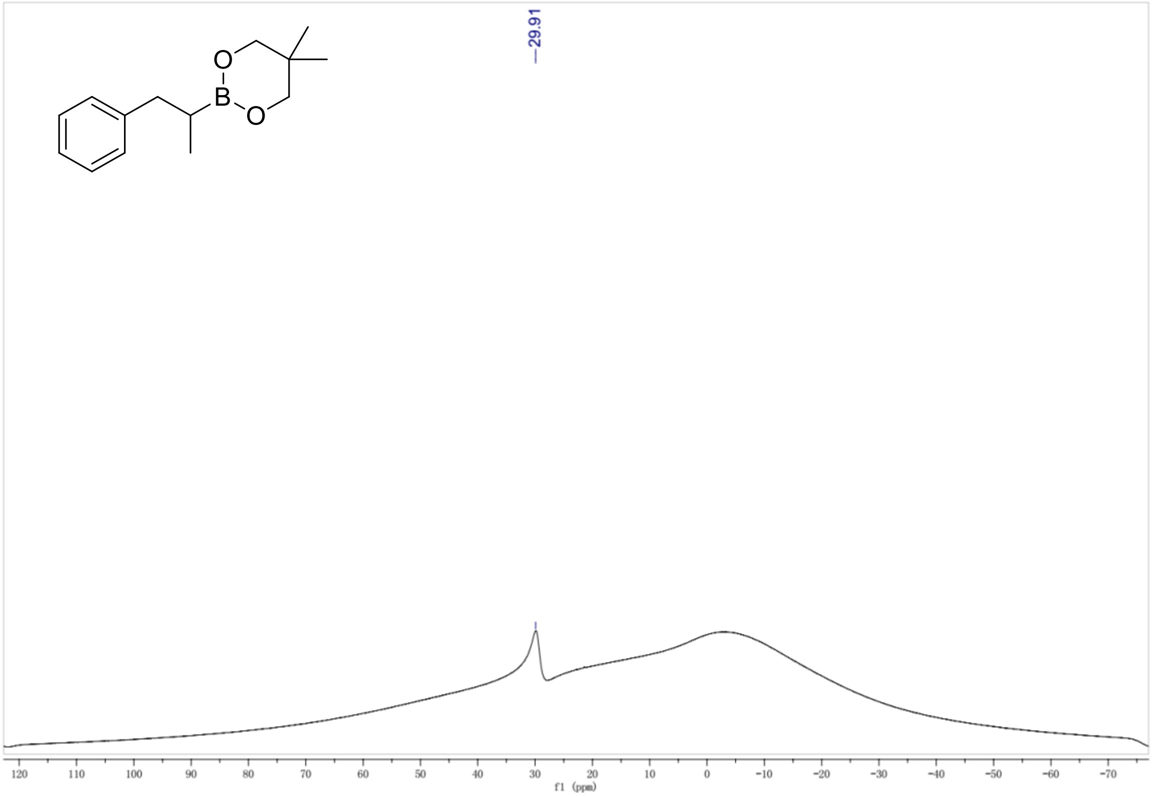

$^{11}B\{^{1}H\}$ NMR spectrum of compound **29b** in $CDCl_3$ (128 MHz).

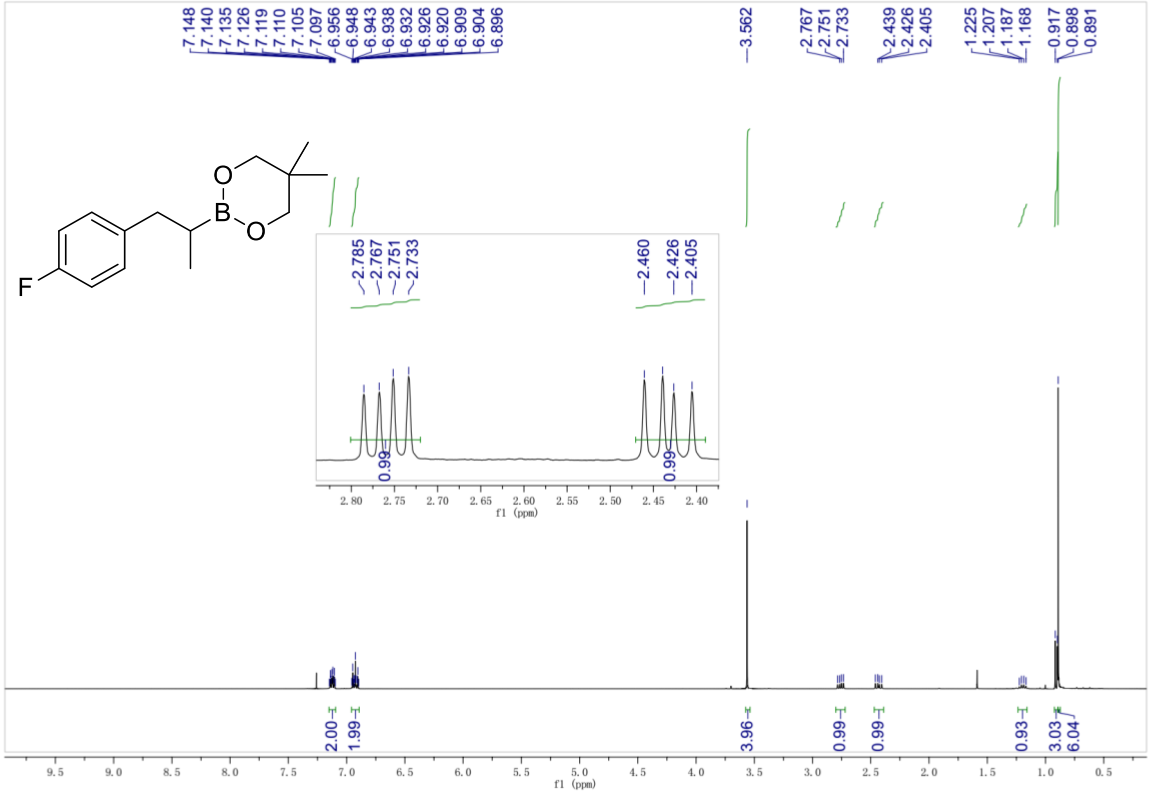

^{1}H NMR spectrum of compound **30b** in $CDCl_3$ (400 MHz).

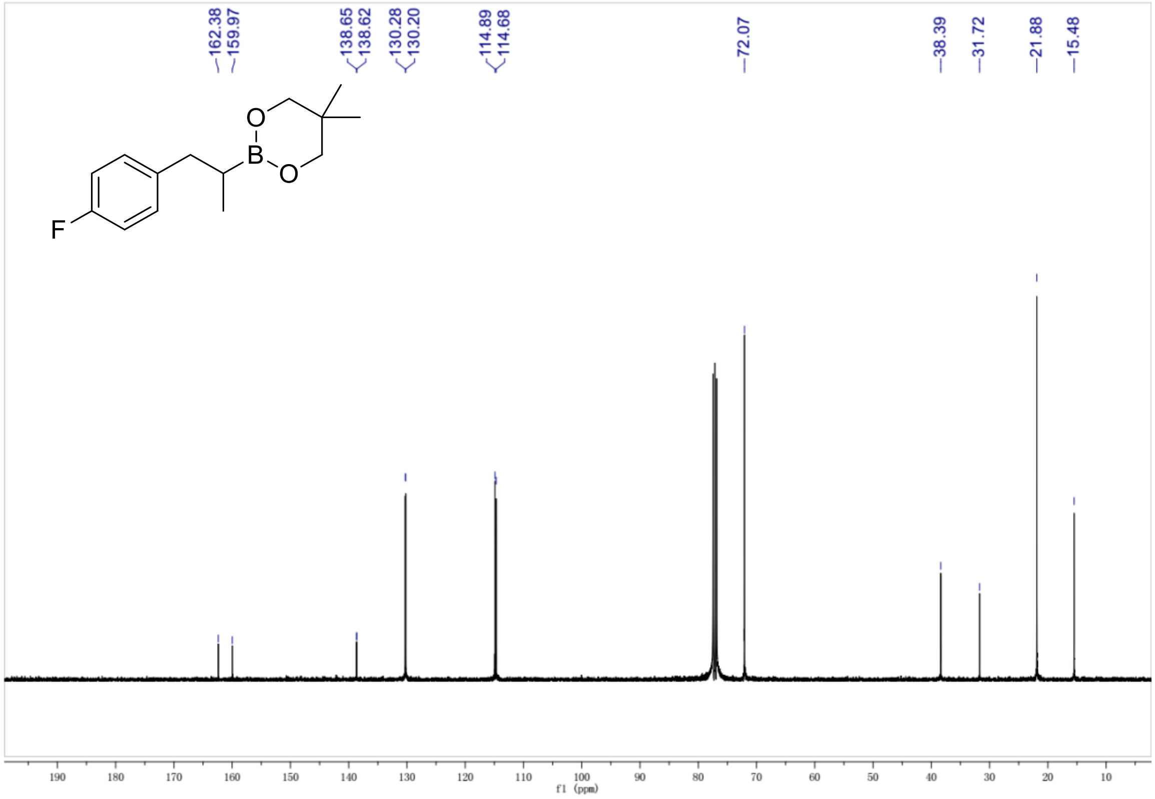

^{13}C{^{1}H} NMR spectrum of compound **30b** in $CDCl_3$ (100 MHz).

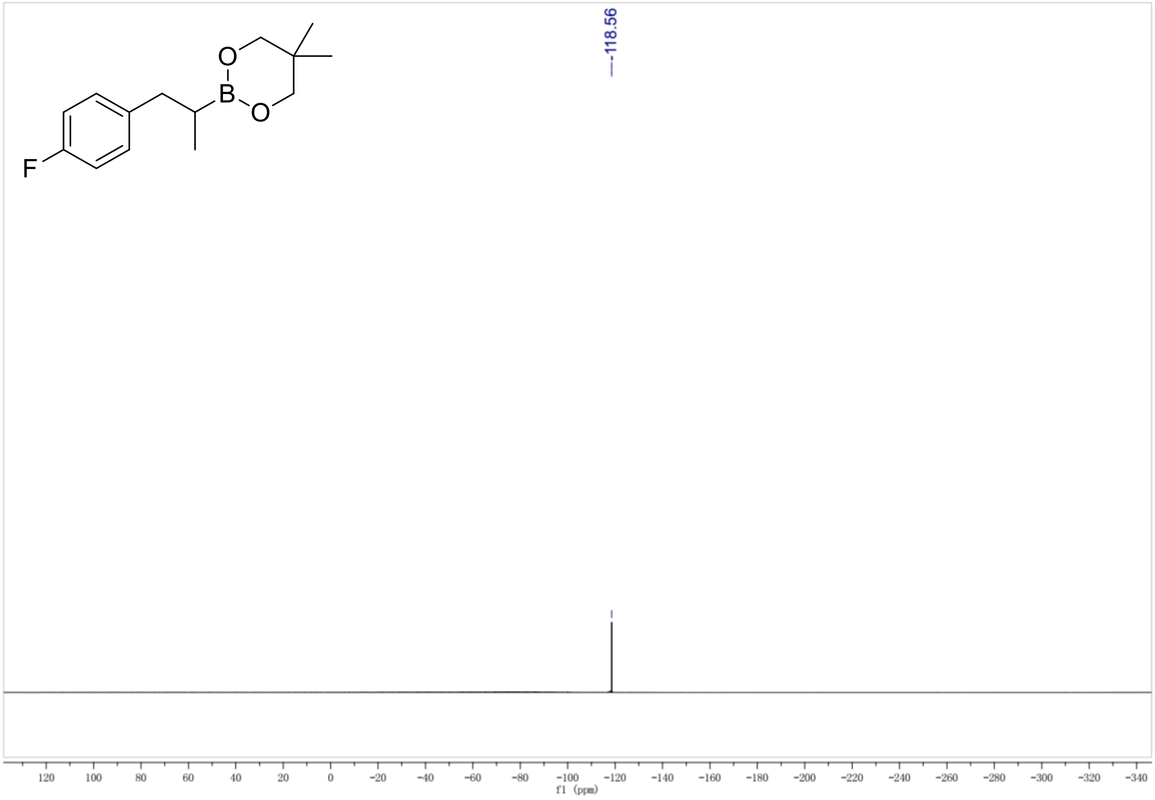

$^{19}F\{^1H\}$ NMR spectrum of compound **30b** in $CDCl_3$ (376 MHz).

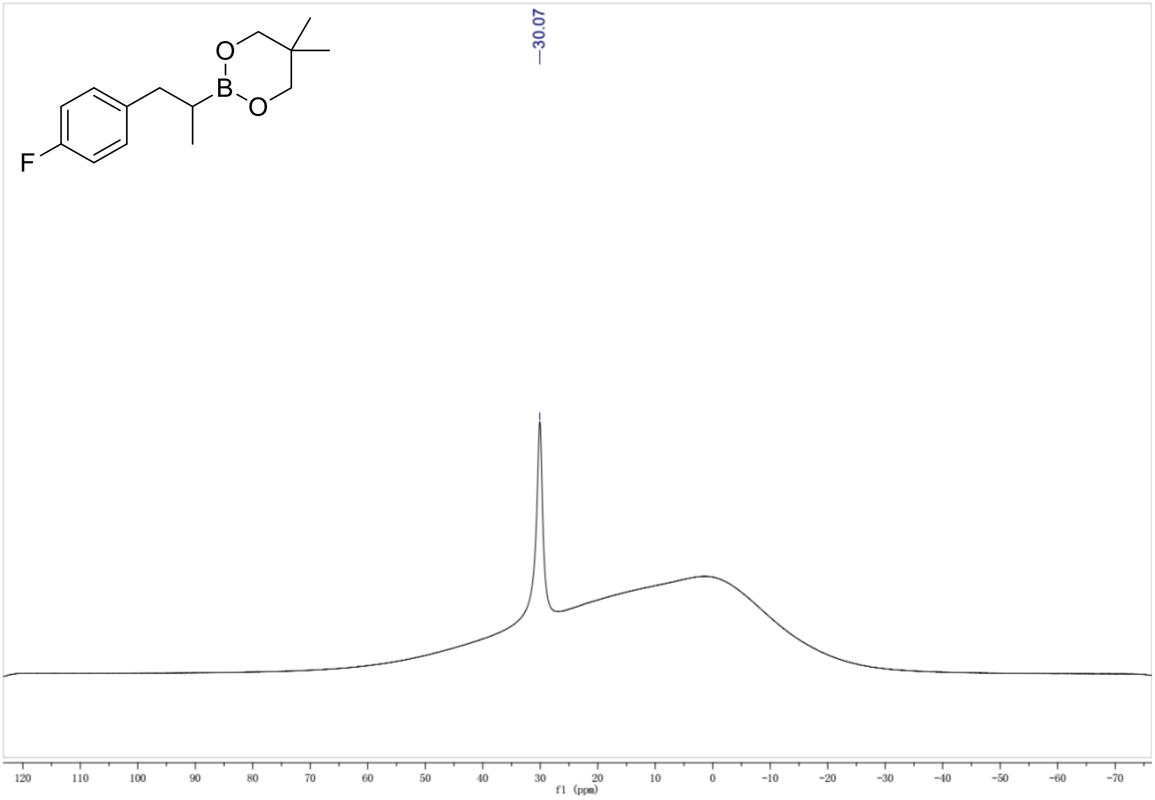

$^{11}B\{^1H\}$ NMR spectrum of compound **30b** in $CDCl_3$ (128 MHz).

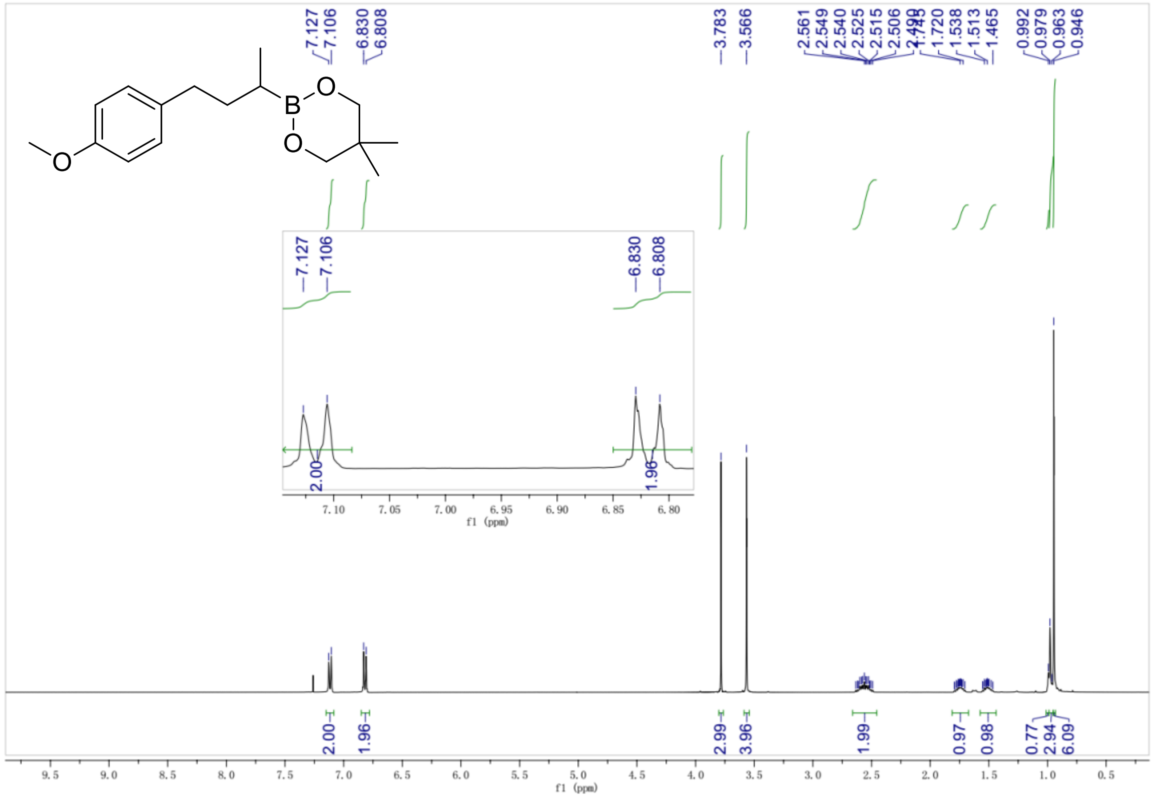

^{1}H NMR spectrum of compound **31b** in $CDCl_3$ (400 MHz).

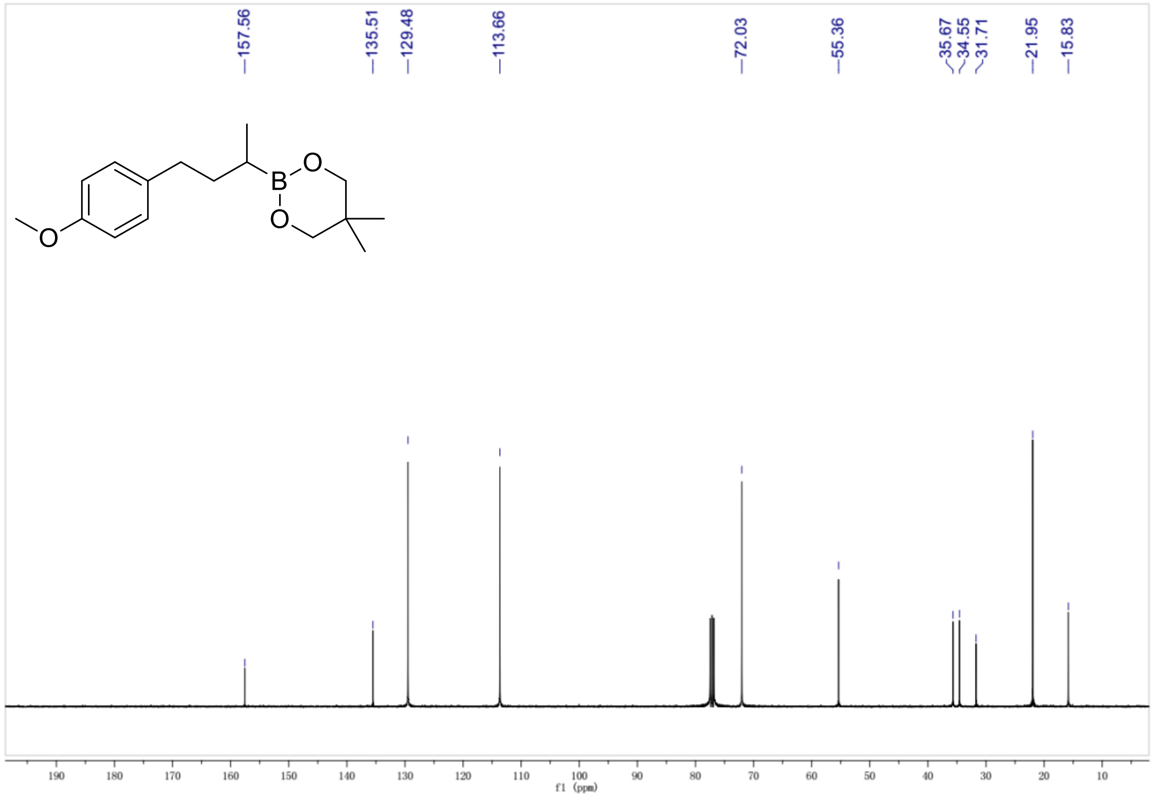

$^{13}C\{^{1}H\}$ NMR spectrum of compound **31b** in $CDCl_3$ (100 MHz).

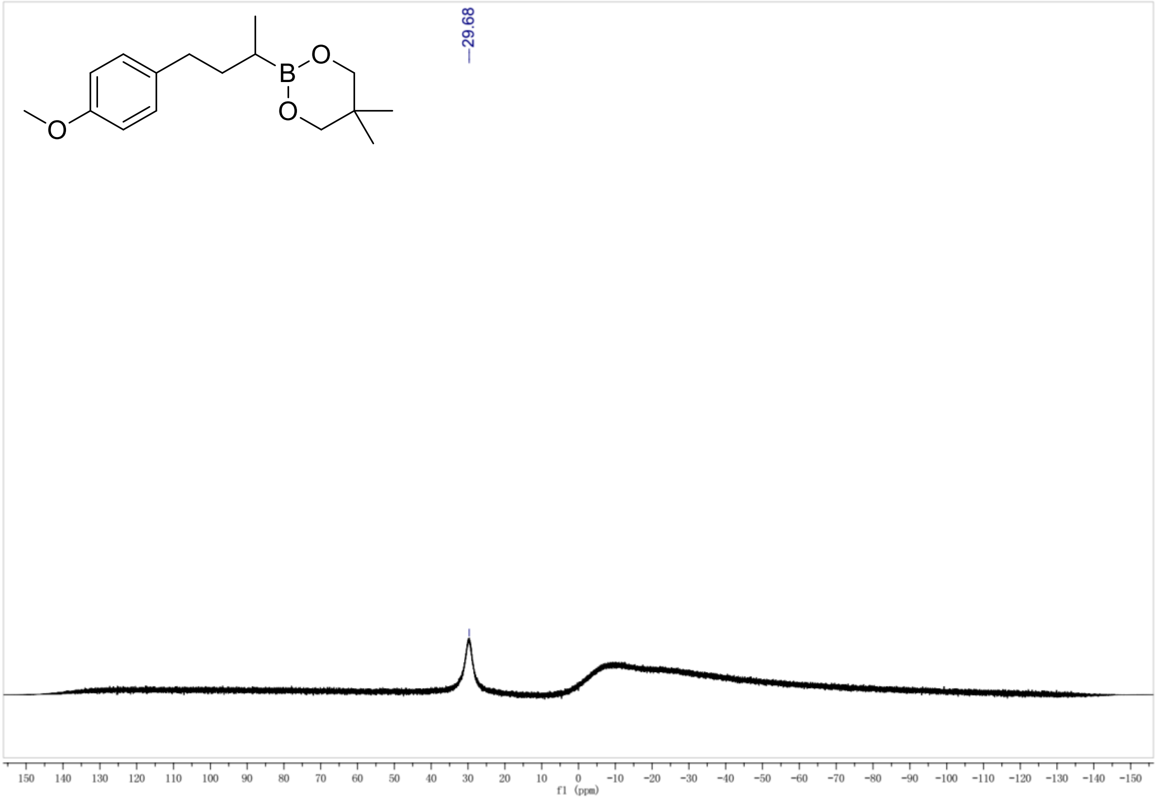

$^{11}B\{^{1}H\}$ NMR spectrum of compound **31b** in $CDCl_3$ (128 MHz).

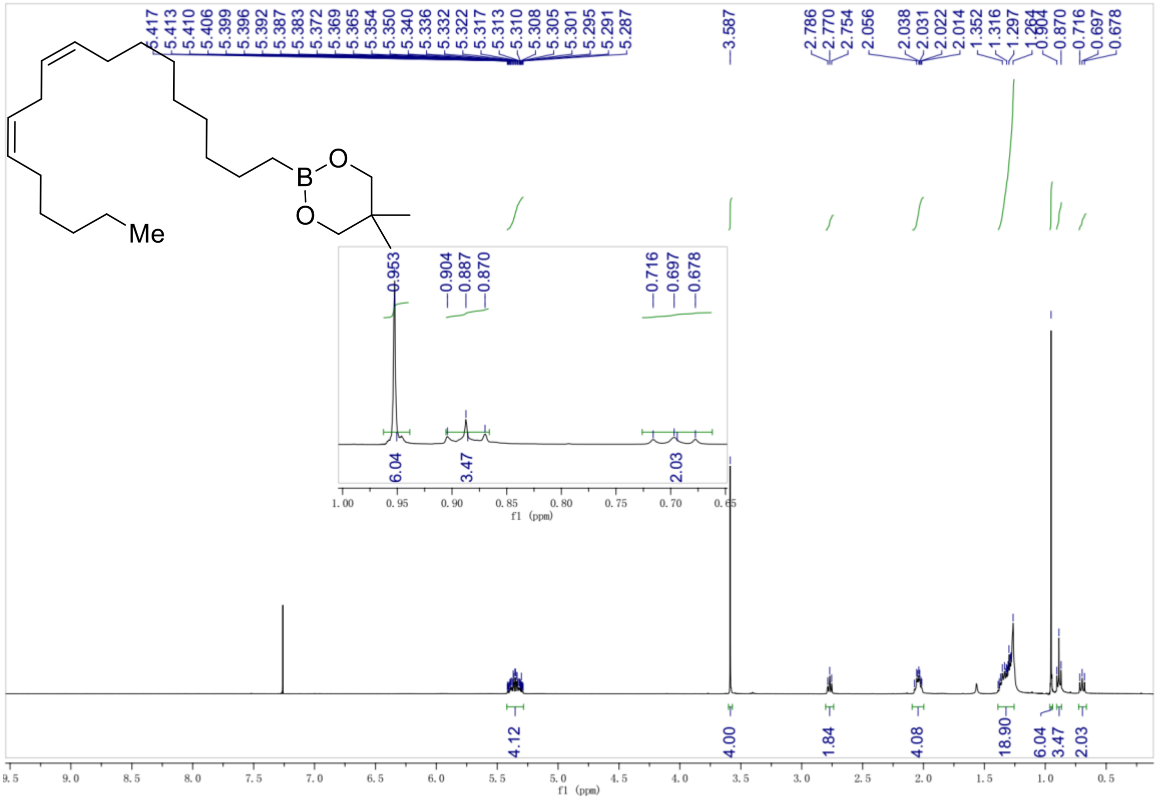

^{1}H NMR spectrum of compound **32b** in $CDCl_3$ (400 MHz).

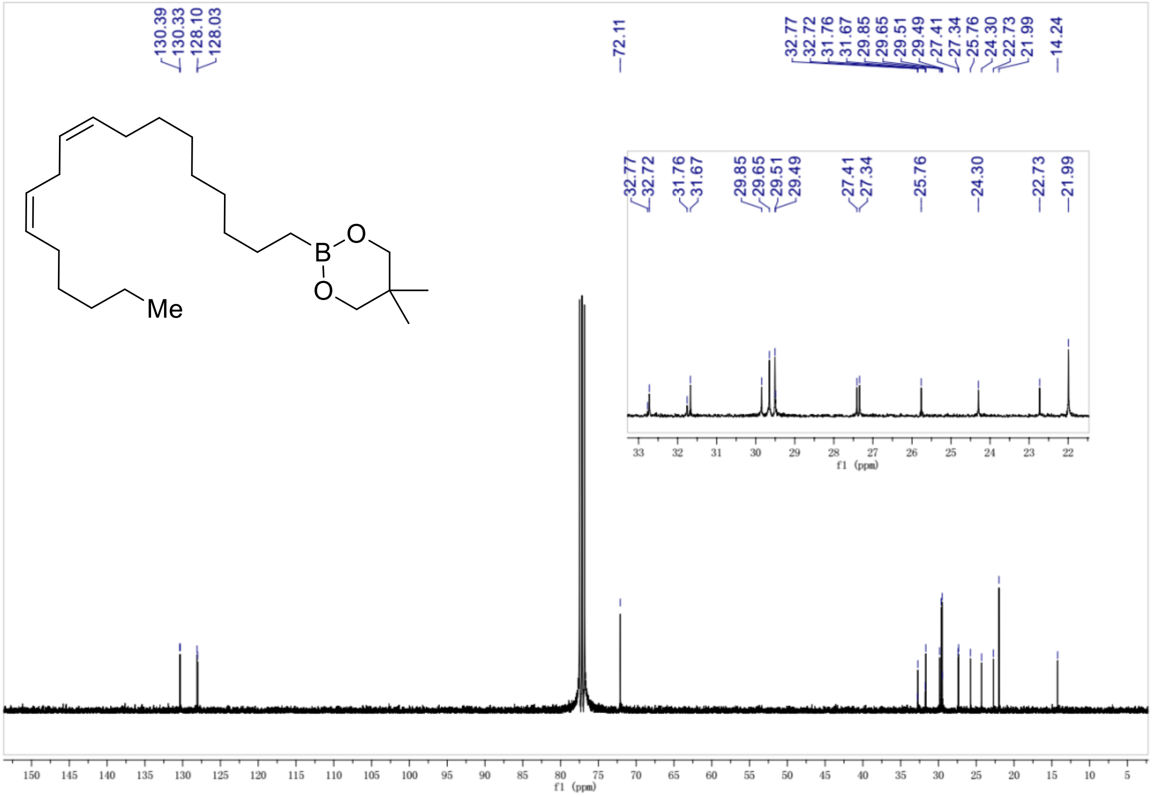

$^{13}C\{^{1}H\}$ NMR spectrum of compound **32b** in $CDCl_3$ (100 MHz).

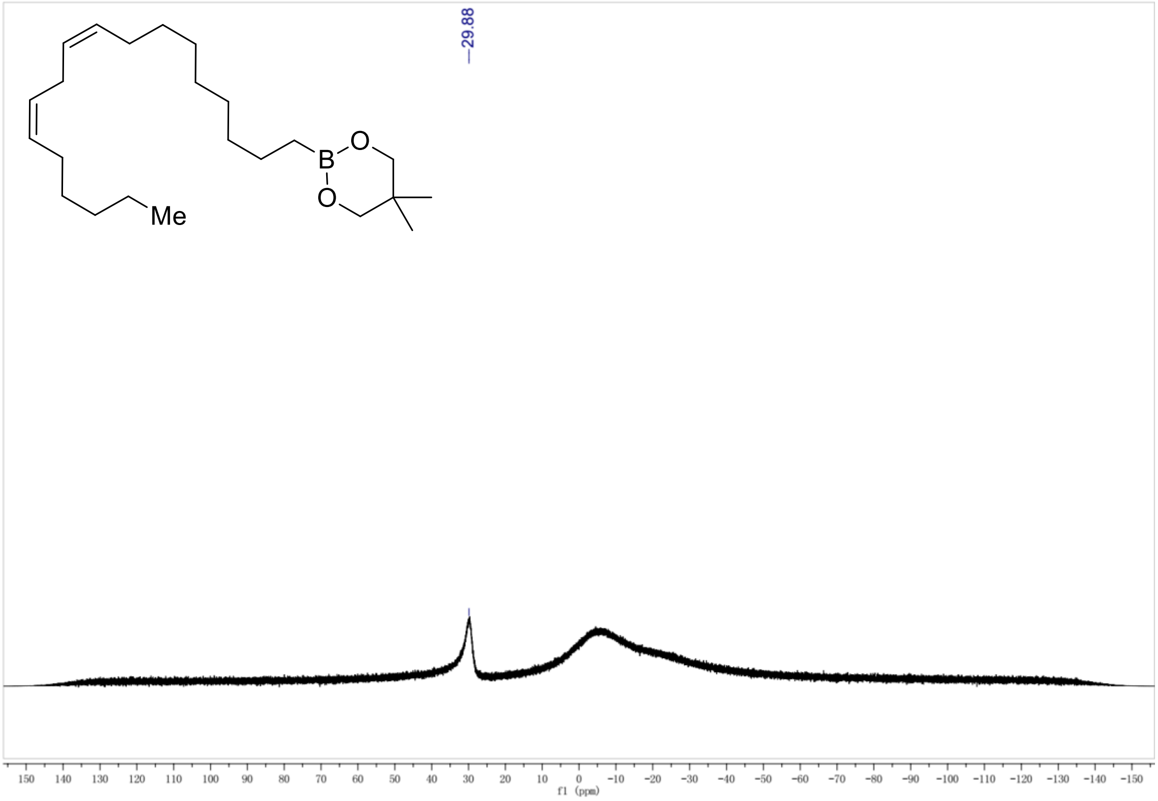

$^{11}B\{^{1}H\}$ NMR spectrum of compound **32b** in $CDCl_3$ (128 MHz).

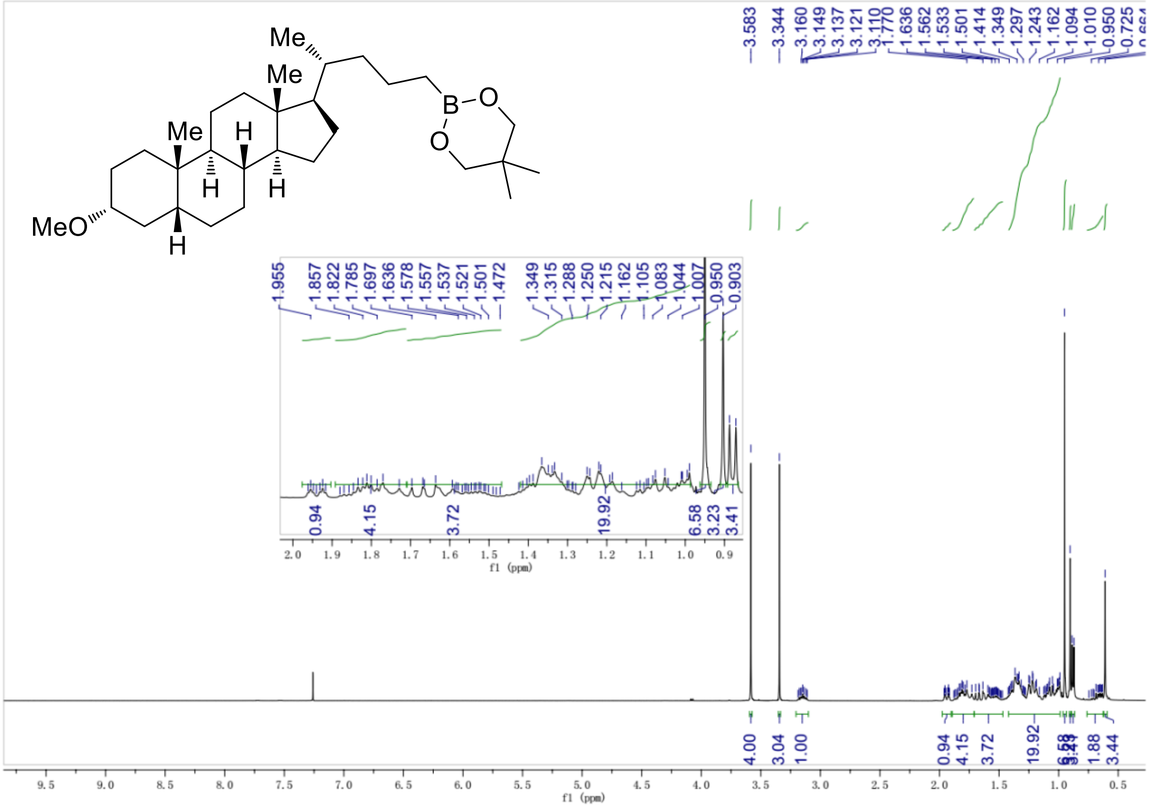

^{1}H NMR spectrum of compound **33b** in $CDCl_3$ (400 MHz).

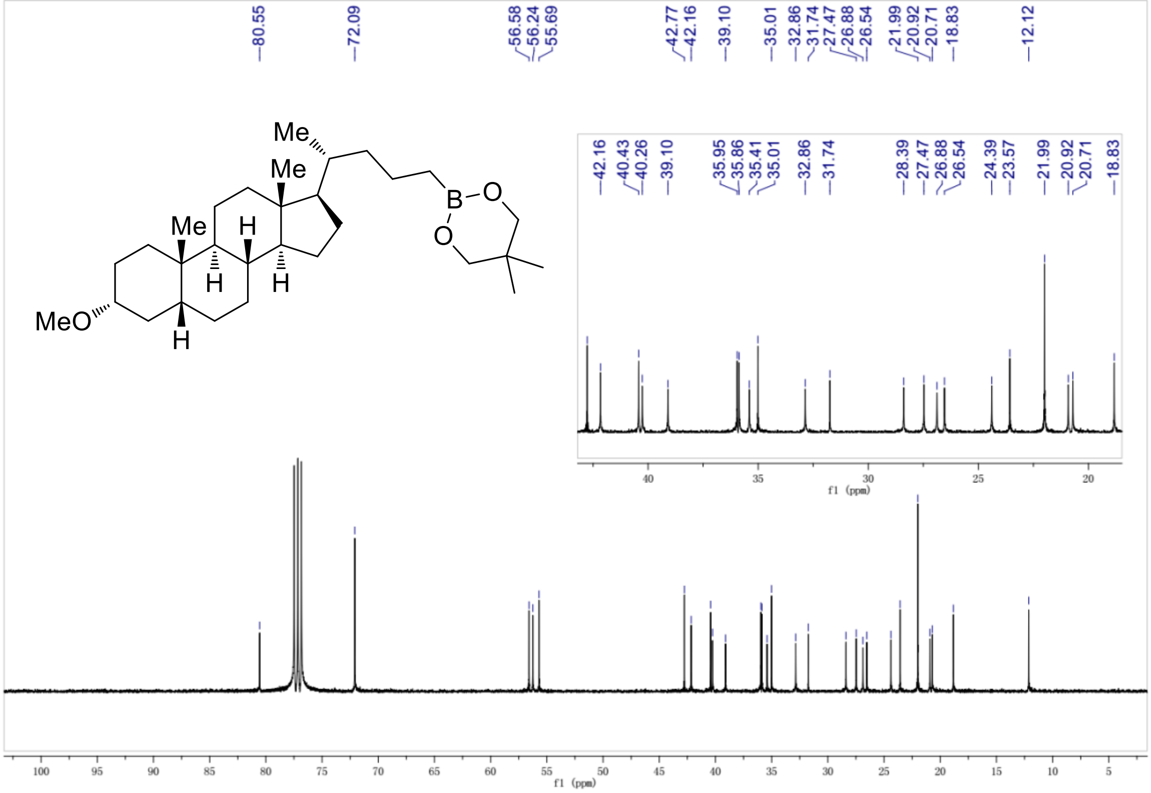

$^{13}C\{^{1}H\}$ NMR spectrum of compound **33b** in $CDCl_3$ (100 MHz).

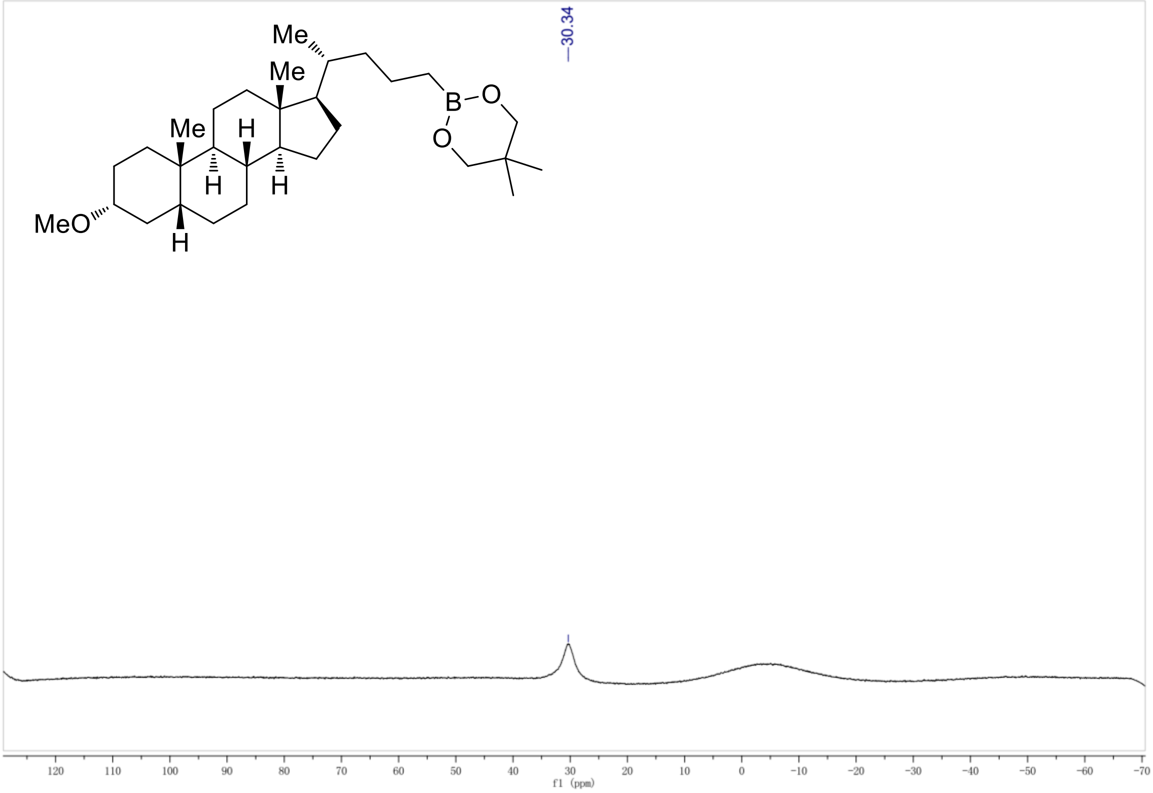

^{11}B{^{1}H} NMR spectrum of compound **33b** in $CDCl_3$ (128 MHz).

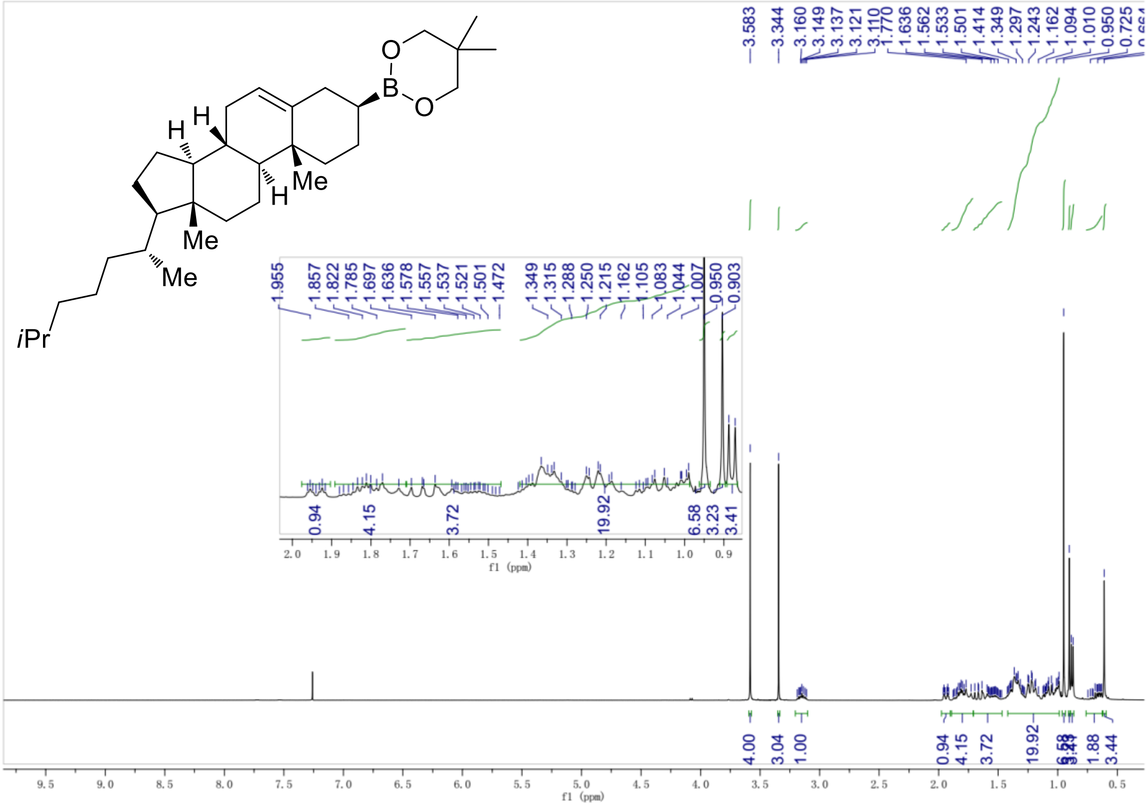

^{1}H NMR spectrum of compound **34b** in $CDCl_3$ (400 MHz).

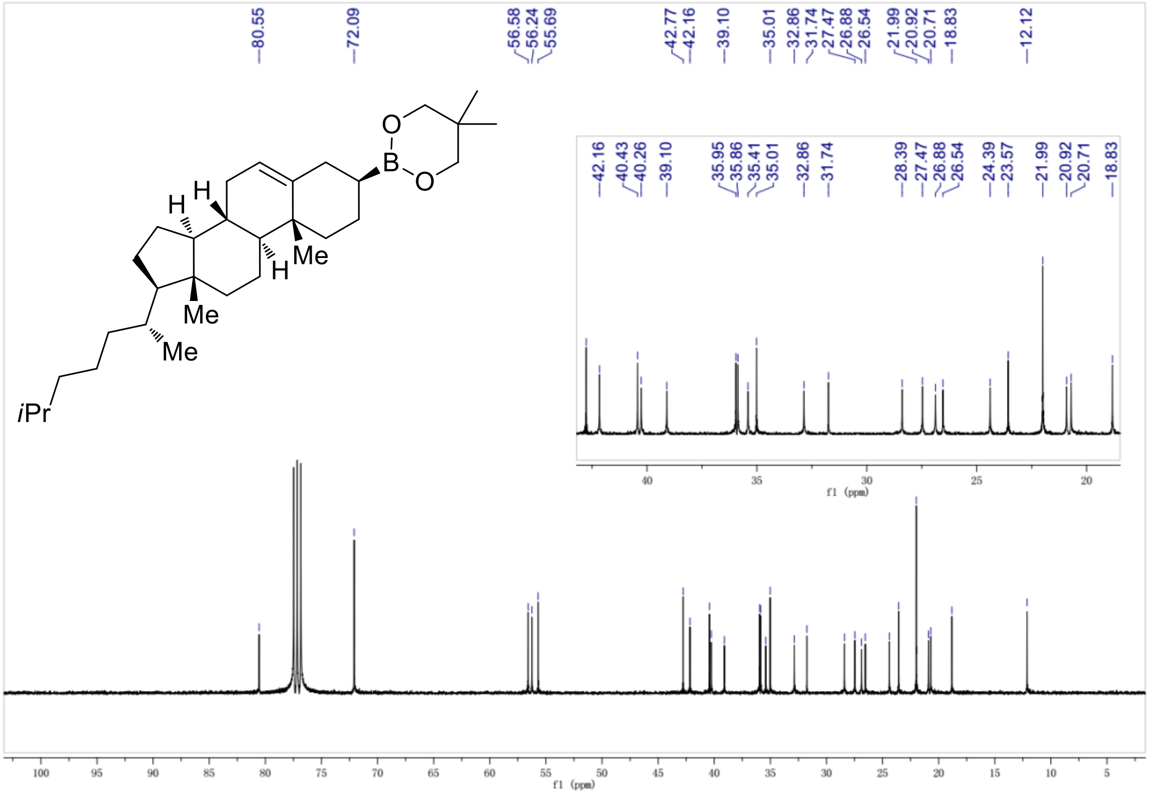

$^{13}C\{^{1}H\}$ NMR spectrum of compound **34b** in $CDCl_3$ (100 MHz).

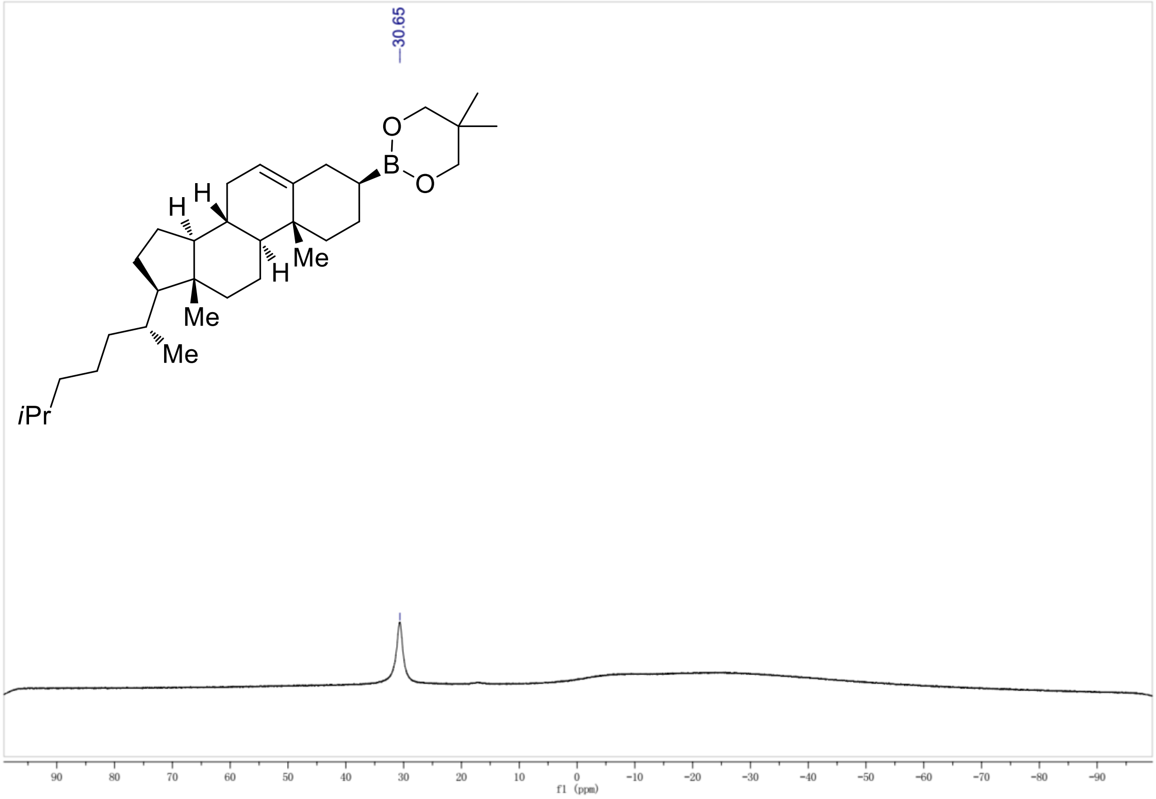

$^{11}B\{^{1}H\}$ NMR spectrum of compound **34b** in $CDCl_3$ (128 MHz).

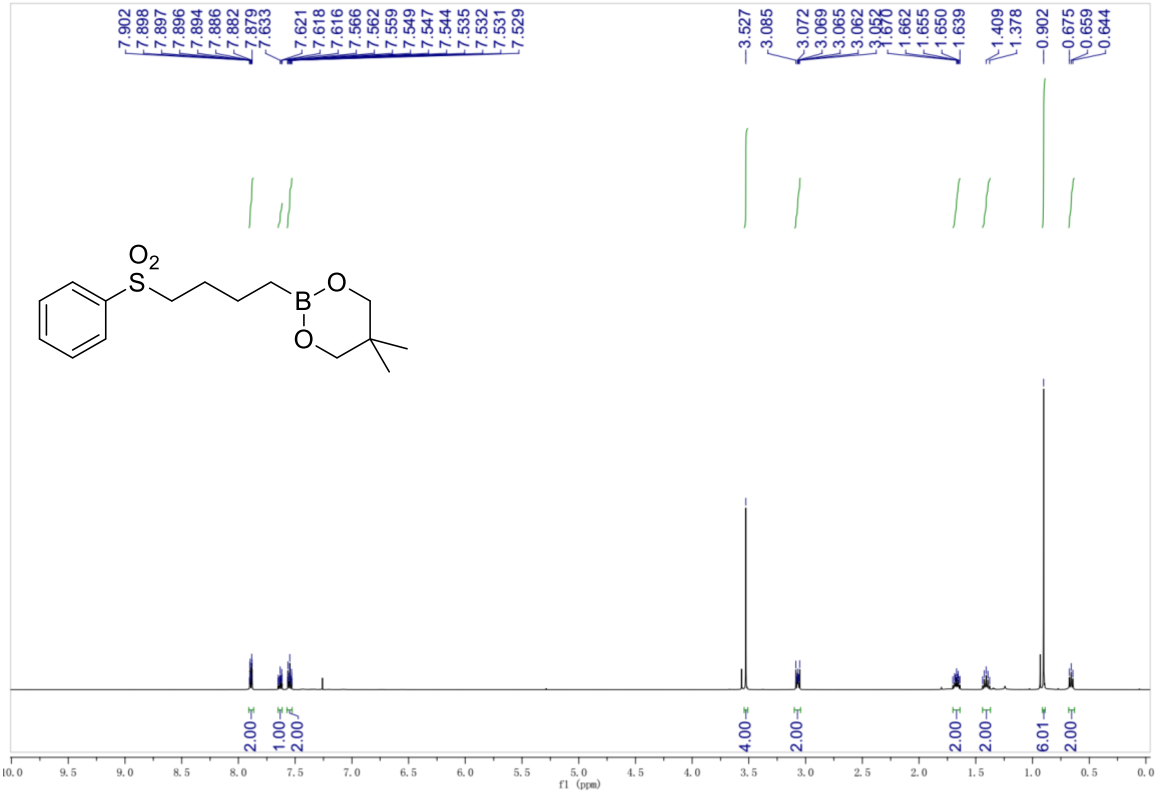

^{1}H NMR spectrum of compound **35b** in $CDCl_3$ (500 MHz).

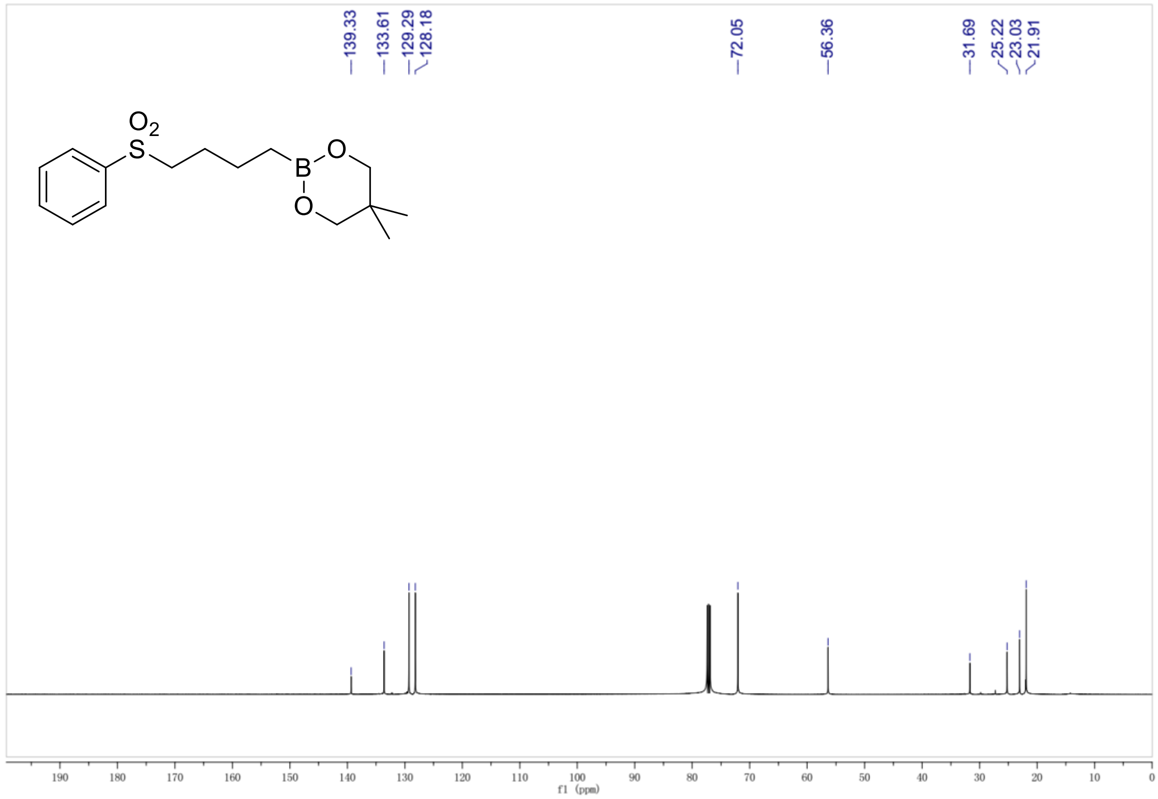

$^{13}C\{^{1}H\}$ NMR spectrum of compound **35b** in $CDCl_3$ (125 MHz).

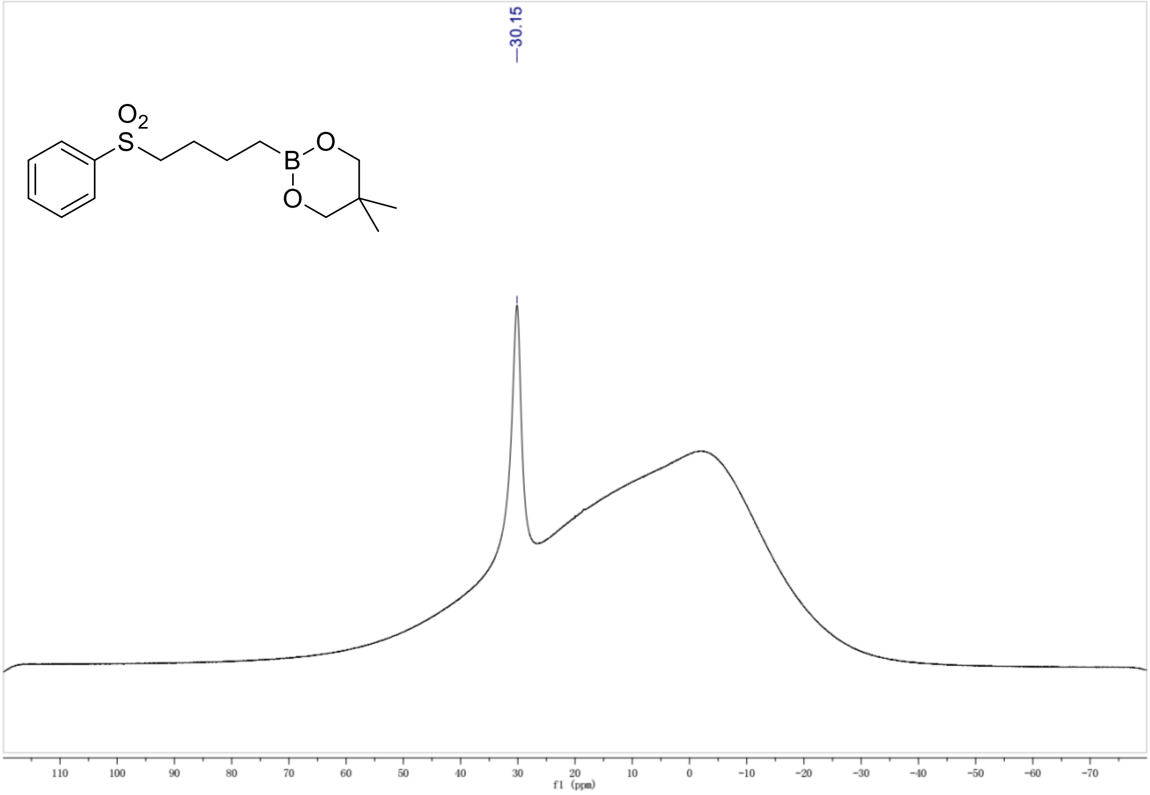

$^{11}B\{^{1}H\}$ NMR spectrum of compound **35b** in $CDCl_3$ (160 MHz).

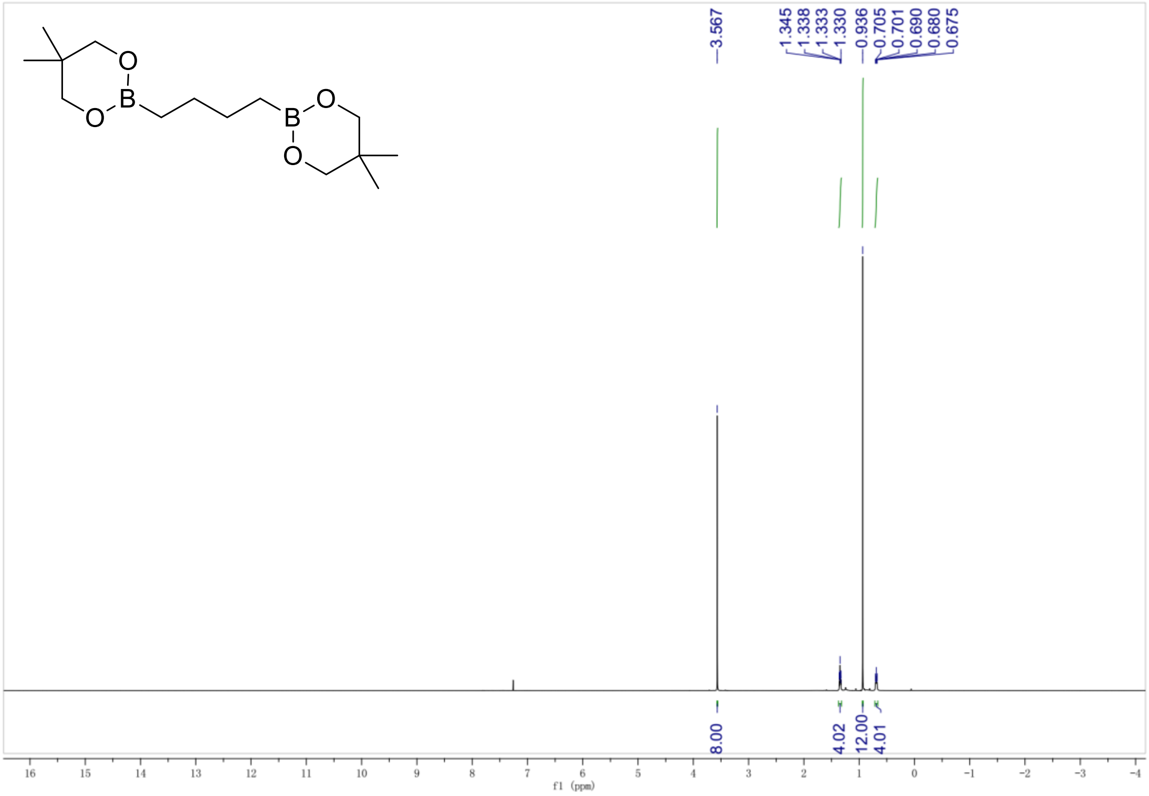

^{1}H NMR spectrum of compound **35b’** in $CDCl_3$ (500 MHz).

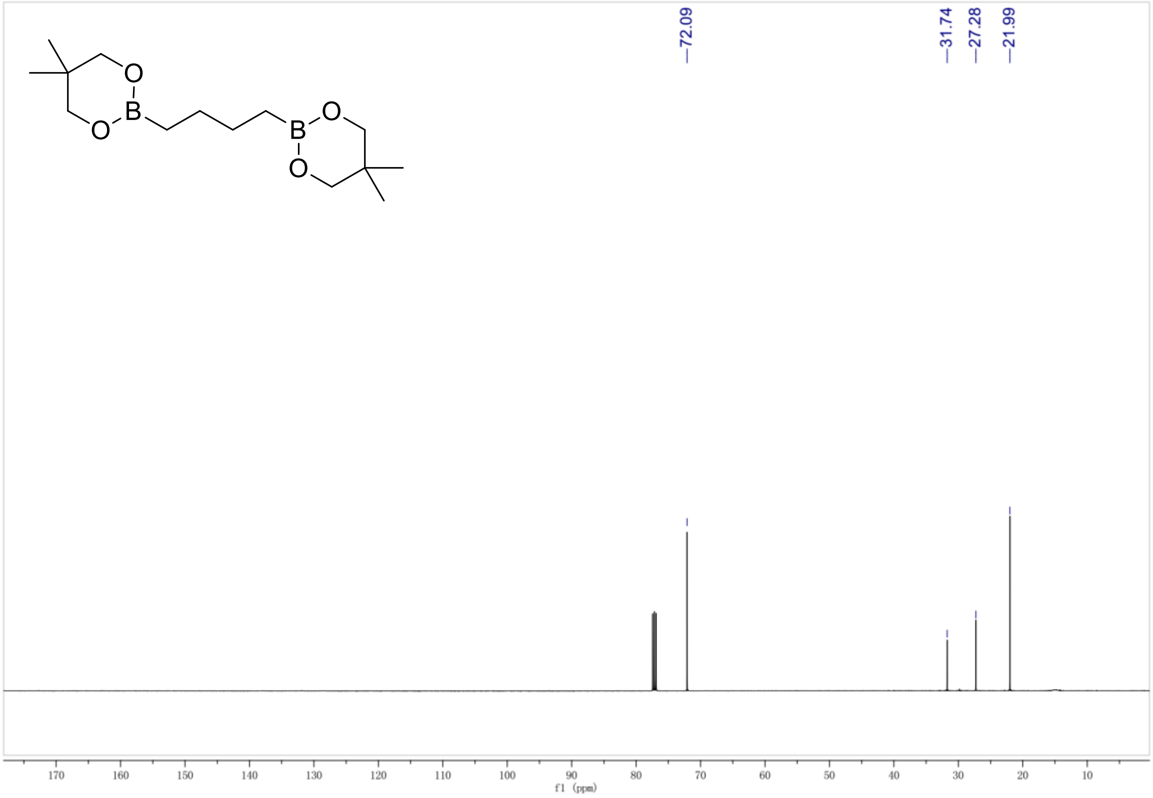

$^{13}C\{^{1}H\}$ NMR spectrum of compound **35b’** in $CDCl_3$ (125 MHz).

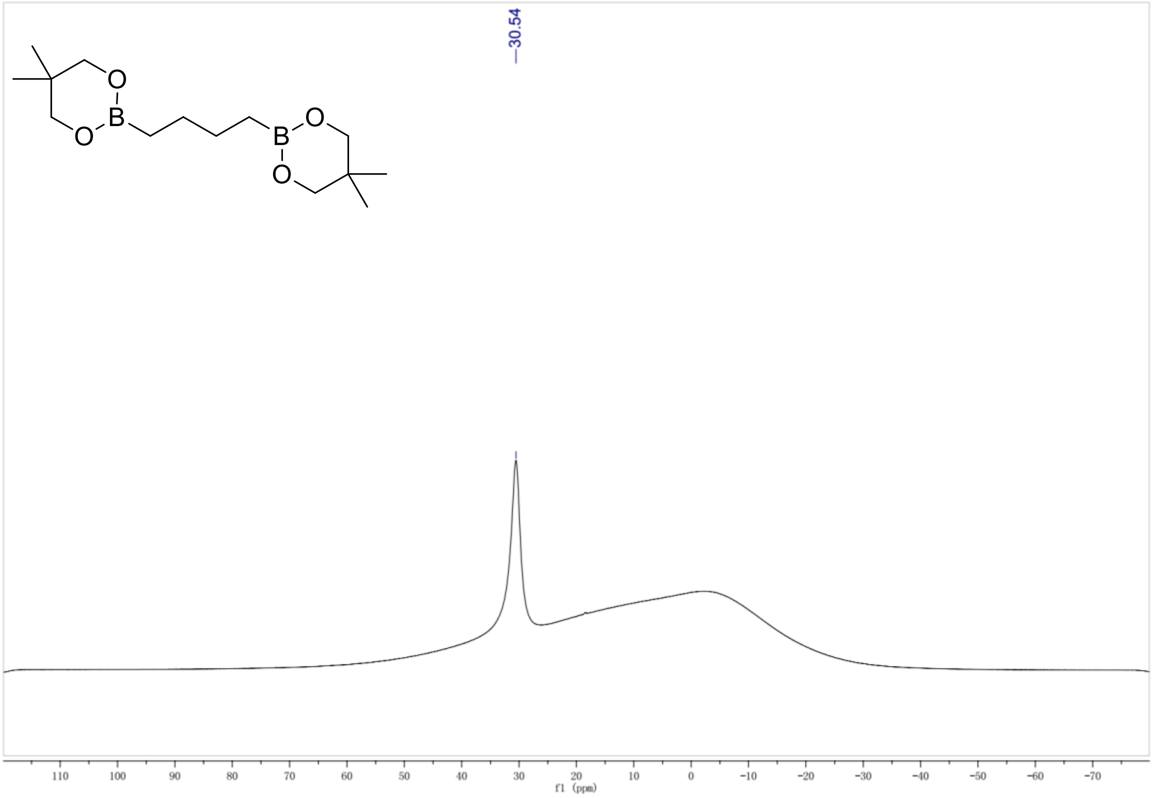

$^{11}B\{^{1}H\}$ NMR spectrum of compound **35b’** in $CDCl_3$ (160 MHz).

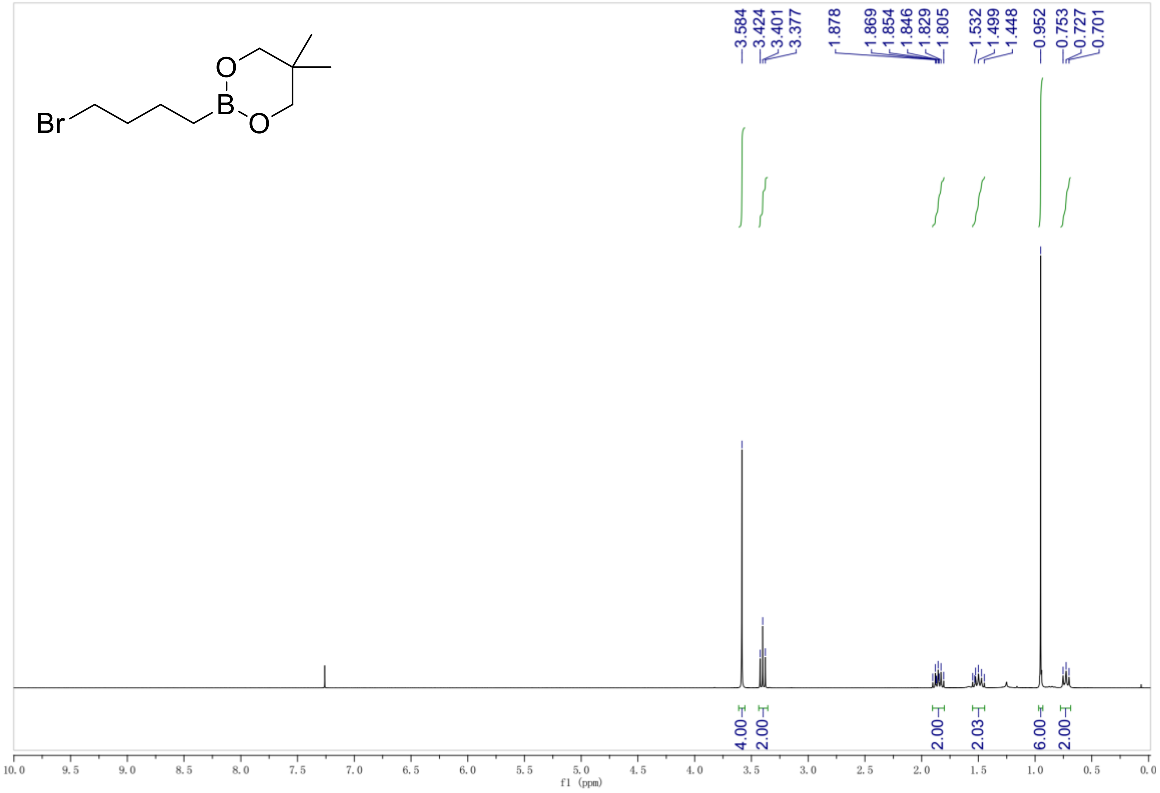

^{1}H NMR spectrum of compound **36b** in $CDCl_3$ (300 MHz).

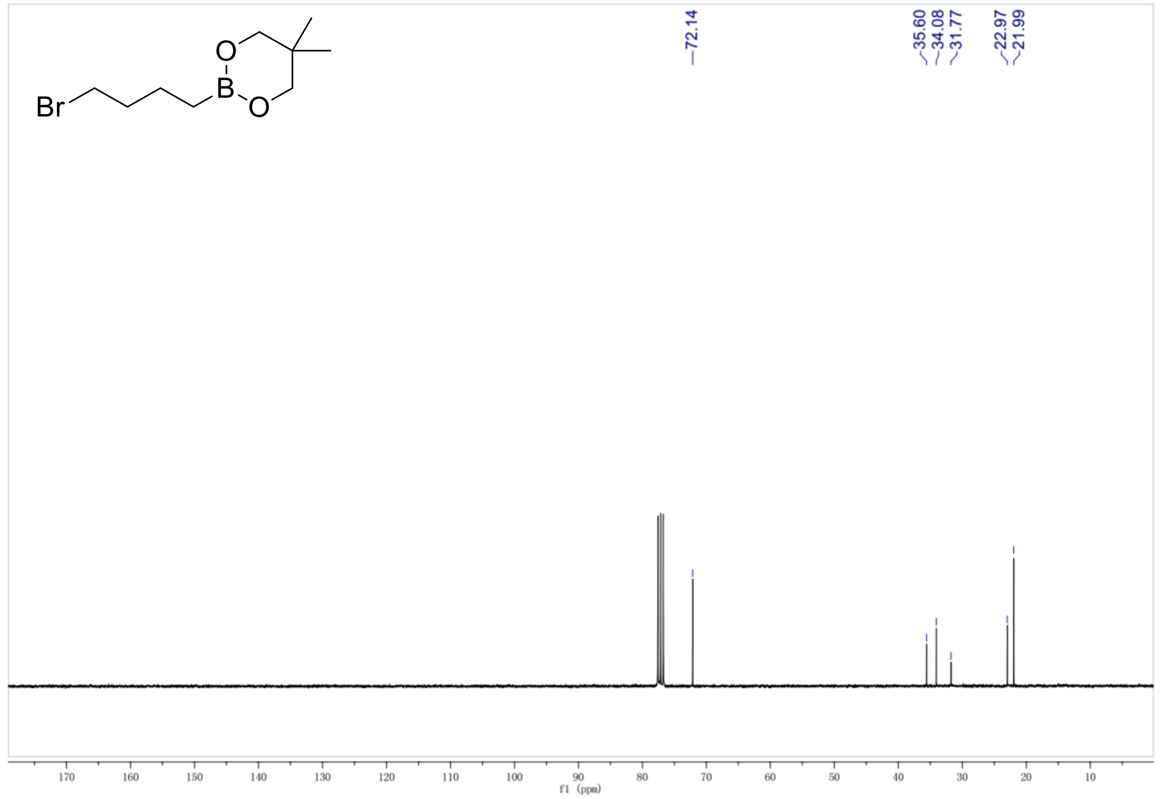

$^{13}C\{^{1}H\}$ NMR spectrum of compound **36b** in $CDCl_3$ (75 MHz).

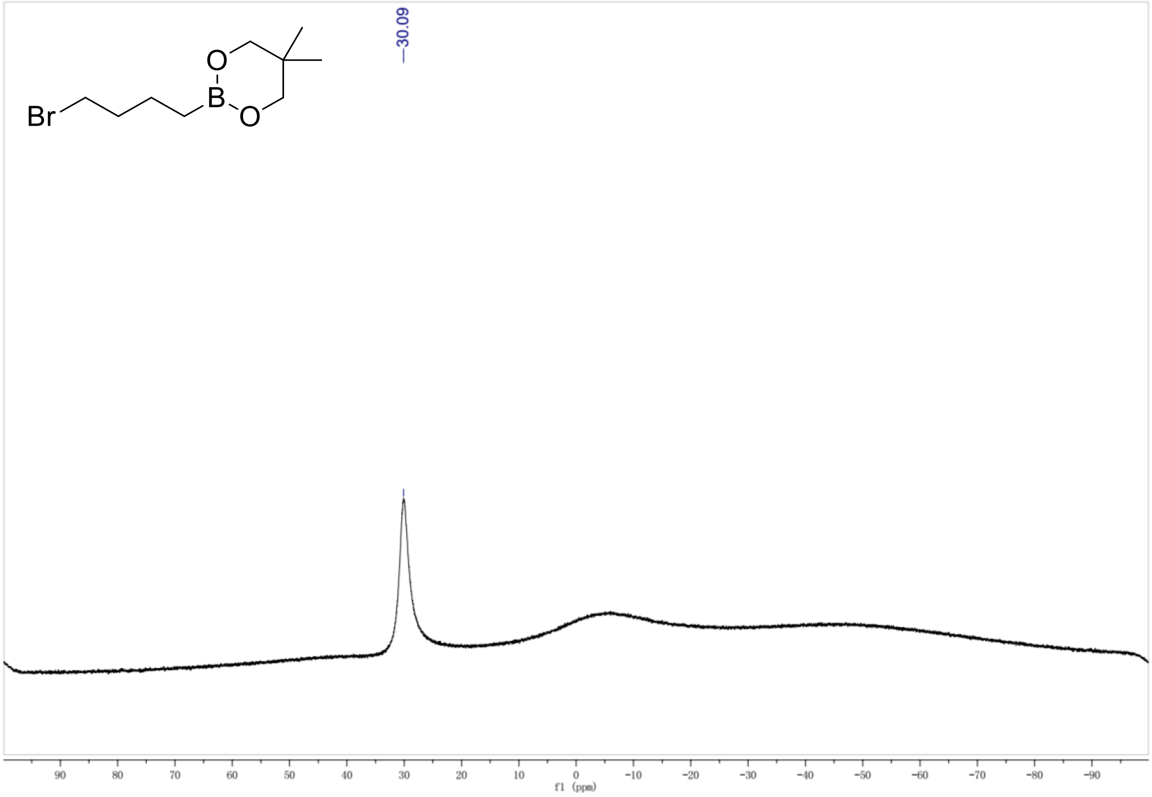

$^{11}B\{^{1}H\}$ NMR spectrum of compound **36b** in $CDCl_3$ (96 MHz).

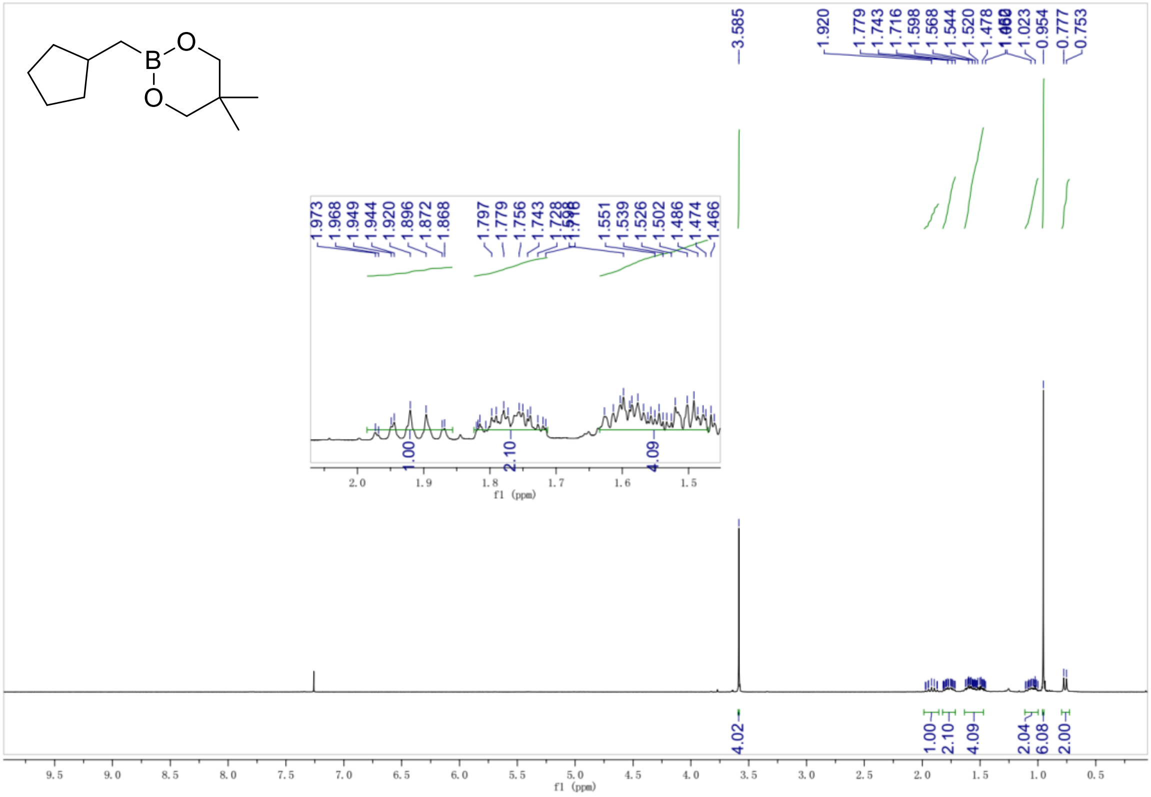

^{1}H NMR spectrum of compound **37b'** in $CDCl_3$ (300 MHz).

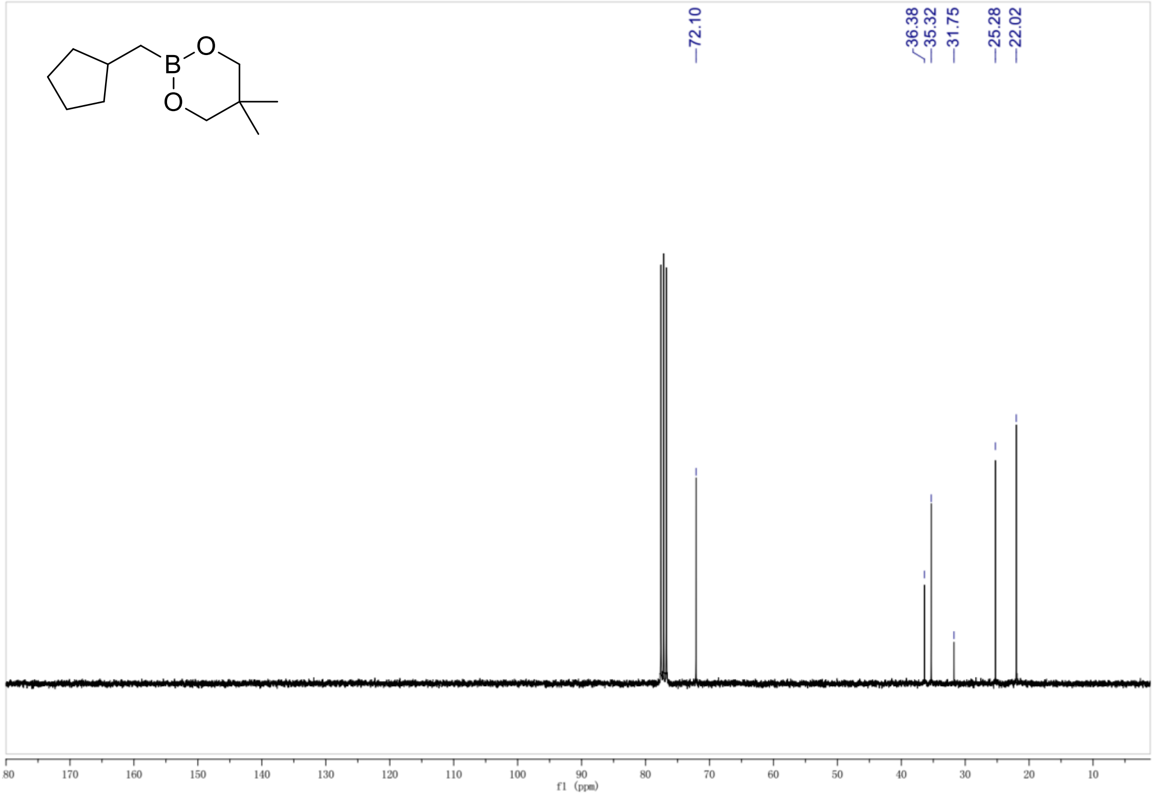

$^{13}C\{^{1}H\}$ NMR spectrum of compound **37b’** in $CDCl_3$ (75 MHz).

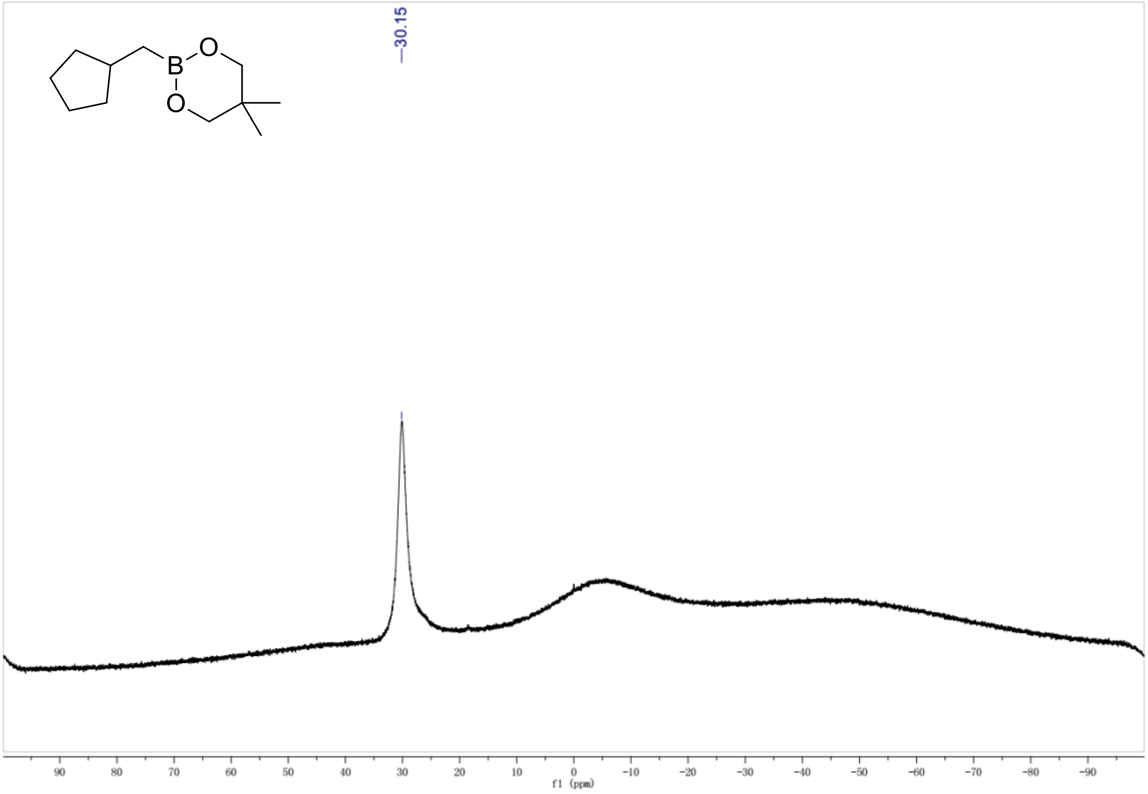

$^{11}B\{^{1}H\}$ NMR spectrum of compound **37b’** in $CDCl_3$ (96 MHz).
